# Ground-state dioxygen undergoes metal-free [3 + 2]-annulations with allenes and nitrosoarenes under ambient conditions†
†Electronic supplementary information (ESI) available. CCDC 1507477, 1507478, 1510902 and 1540299. For ESI and crystallographic data in CIF or other electronic format see DOI: 10.1039/c7sc01770g



**DOI:** 10.1039/c7sc01770g

**Published:** 2017-05-24

**Authors:** Jinxian Liu, Manisha Skaria, Pankaj Sharma, Yun-Wei Chiang, Rai-Shung Liu

**Affiliations:** a Department of Chemistry , National Tsing-Hua University , Hsinchu , Taiwan , Republic of China . Email: rsliu@mx.nthu.edu.tw; b College of Chemistry & Materials Science , Longyan University , Fujian , China

## Abstract

The cycloadditions of molecular dioxygen with neutral π-bond motifs rely heavily on singlet-state ^1^O_2_, whereas ground state ^3^O_2_ is chemically inactive. Here we report novel [3 + 2]-annulations among ground-state ^3^O_2_ (1 bar), allenes, and nitrosoarenes at low temperatures, efficiently yielding dioxygen-containing oxacycles. With less hindered 1-arylallene derivatives, these dioxygen species undergo skeletal rearrangement to 3-hydroxy-1-ketonyl-2-imine oxides. These cycloadditions represent valuable one-pot *O*,*N*,*O*-trifunctionalizations of allenes. Our EPR experiments confirm the presence of 1,4-diradical intermediates from an allene/nitrosoarene mixture, which manifest the hidden diradical properties of nitrosoarenes.

## Abstract 2

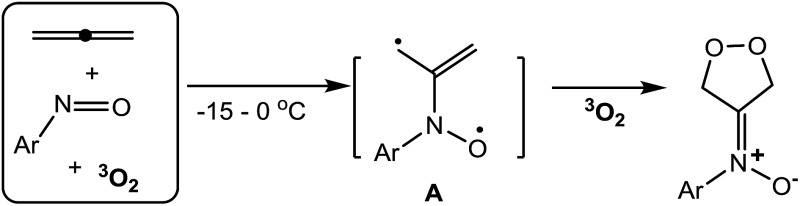
We report novel [3 + 2]-annulations among ground-state ^3^O_2_ (1 bar), allenes, and nitrosoarenes at low temperatures, efficiently yielding dioxygen-containing oxacycles.

## Introduction

**Scheme 1:**
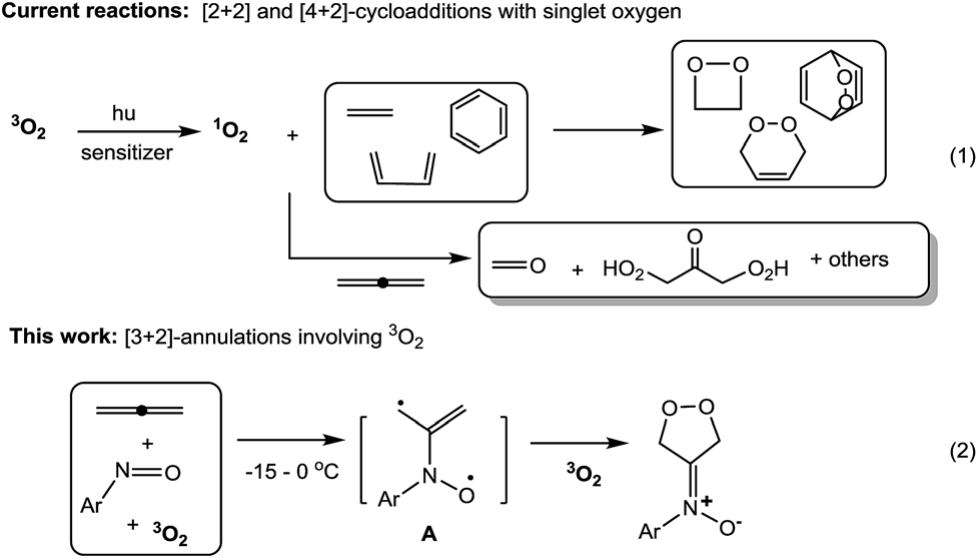
Cycloadditions of unsaturated hydrocarbons with ^1^O_2_ and ^3^O_2_.

Cycloadditions of two or three π-bond molecules are powerful tools to access carbo- or heterocycles. Ground-state ^3^O_2_ has low-lying LUMO orbitals, but its triplet state greatly reduces its chemical reactivity toward neutral molecules^1^ unless a metal catalyst is present. The cycloadditions of ^3^O_2_ dioxygen rely nearly exclusively on prior photo-activation to form singlet-state ^1^O_2_ (ref. 1) that reacts with dienes,^2^ olefins^3^ or even arenes^4^ in [*n* + 2]-cycloadditions (*n* = 2 and 4, Scheme 1, eqn (1)). This photolytic process requires a sensitizer in a cold bath (–40 °C) over a protracted period (>12 h) because highly energetic ^1^O_2_ might produce byproducts from the oxygen-ene reactions^5^ and oxidative C=C cleavages.^6^ In the case of allenes, singlet dioxygen afforded a complicated mixture of undesired compounds.^7a,b^

As ground-state ^3^O_2_ is a free π-molecule and is available everywhere; its metal-free [*n* + 2]-cycloadditions with commonly used unsaturated hydrocarbons would provide a clean and cheap synthesis of valuable 1,*n*-diols, although there is no literature precedence. As far as we are aware, only 1,4-diradical precursors such as *o*-benzocyclobutanes,^8^ 1,2,6,7-octatetraenes,^9^ 2,3-dimethylenebicyclo[2.2.0]hexane^10^ and other 1,4-diazo species^11^ reacted with ground-state ^3^O_2_ in thermal [4 + 2]-cycloadditions; these precursors are too uncommon to show general utility. We recently achieved metal-catalyzed annulations of *N*-hydroxy allenylamines with nitrosoarenes *via* a single radical process.^7*d*^ In search of a breakthrough in dioxygen chemistry, we developed facile [3 + 2]-cycloadditions among nitrosoarenes, allenes and ground-state ^3^O_2_ to efficiently afford *N*-(1,2-dioxolan-4-ylidene)aniline oxides (eqn (2)). Particularly notable are the ambient conditions: –15 to 0 °C, ^3^O_2_ (1 bar), no light, no catalyst and no additive. Importantly, these facile spin-forbidden dioxygen annulations reveal a new role of nitrosoarenes as effective diradical precursors that is synthetically significant in nitroso chemistry.^12^ In the context of nitroso/alkene and nitroso/alkyne reactions,^13^ theoretical calculations by Houk^12e,f^ suggested the intermediacy of the diradical species, but these transient species could not be trapped with dioxygen or other small molecules.

**Fig. 1:**
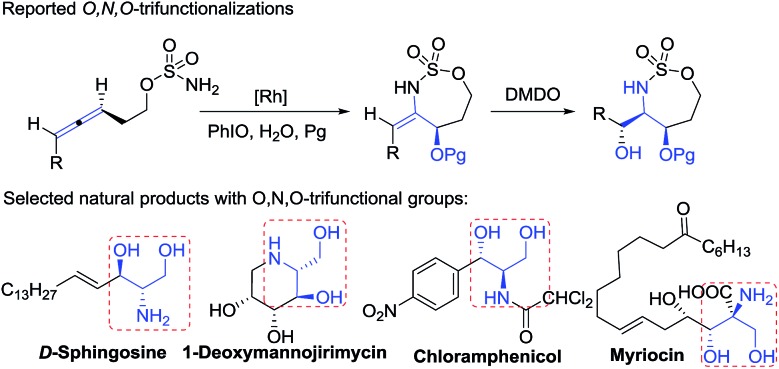
*O*,*N*,*O*-Trifunctionalizations of allenes and selected natural products.

2-Amino-1,3-diols are present in numerous natural products with diverse biological activity (Fig. 1).^14^ Catalytic *O*,*N*,*O*-trifunctionalization of allenes is a new appealing tool to assess these motifs, as noted by the work of Schomaker, who reported Rh-catalyzed intramolecular cyclizations of homoallenylsulfamate esters *via* a two-step sequence.^15*a*^ In contrast, our one-pot intermolecular *O*,*N*,*O*-functionalizations employ common and cheap nitrosoarenes, allenes and oxygen.

## Results and discussion

Table 1 presents the optimized yields of a *O*,*N*,*O*-trifunctionalized molecule **3a** from a mixture of allene **1a**, nitrosobenzene **2a** (*n* equiv.) and O_2_ (1 bar). When 1.5 equiv. of nitrosobenzene **2a** was used in cold THF (–15 °C), the yield was 43% (entry 1). The yield of **3a** increased to 63% with nitrosobenzene in three fold proportions (entry 2). In other solvents, the yields of **3a** were 50% in toluene, 54% in CH_3_CN, and 58% in DCM (entries 3–5). The yield of **3a** decreased substantially to 10% in THF at 25 °C (entry 6). The reaction under N_2_ failed to yield the desired product **3a** in a traceable amount (entry 7).^16^ Compound **3a** assumes an E-configuration with its hydroxyl *cis* to the nitrone oxygen to form a hydrogen bond. This structure was inferred from X-ray diffraction measurements of its relative **3b** ^17^ (Table 2 entry 1).

**Table 1:** Optimization of reaction conditions

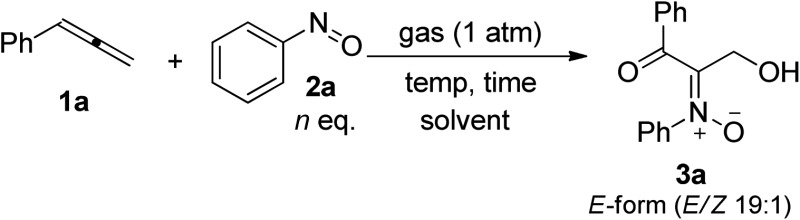						
Entry	Solvent^*a*^	Gas	*n*	*T* (°C)	*t* (h)	Yield^*b*^ (%)
1	THF	O_2_	1.5	–15	2	43
2	THF	O_2_	3	–15	2	63
3	Toluene	O_2_	3	–15	2	50
4	MeCN	O_2_	3	–15	2	54
5	DCM	O_2_	3	–15	2	58
6	THF	O_2_	3	25	2	10
7	THF	N_2_	3	–15	10	—

^*a*^[**1a**] = 0.1 M.

^*b*^Product yields are reported after purification using a silica column.

**Table 2:** *O*,*N*,*O*-Trifunctionalizations of allenes with O_2_ and ArNO^*a*^ ^,^ ^*b*^

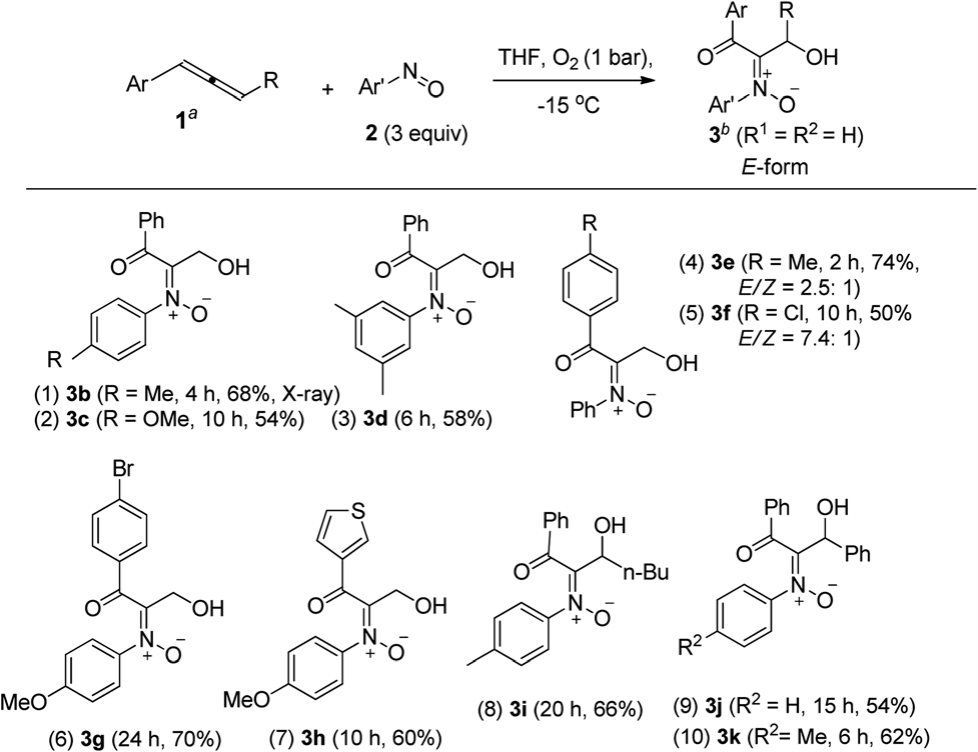

^*a*^[**1**] = 0.1 M.

^*b*^Product yields are reported after purification using a silica column.

To assess the reaction scope, we applied these optimized conditions to additional mono- and 1,3-disubstituted allenes **1b–1g**; Table 2 summarizes the results. For phenylallene **1a**, its corresponding reactions with 4-methyl-, 4-methoxy- and 3,5-dimethylphenylnitroso species afforded 3-hydroxy-1-ketonyl-2-imine oxides **3b–3d** in 54–68% yields (entries 1–3). Varied arylallenes **1b–1e** (Ar = 4-MeC_6_H_4_, 4-ClC_6_H_4_, 4-BrC_6_H_4_ and 3-thienyl) yielded desired compounds **3e–3h** in satisfactory yields (50–74%, entries 4–6). 3-Substituted phenylallenes **1f** and **1g** (R = *n*-Bu and Ph) were also effective substrates for these cycloadditions (entries 8–10).

Notably, the reaction of sterically hindered 3-cyclohexyl-1-phenylallene **1i** with 4-methoxyphenylnitroso **2c** and O_2_ (1 bar) afforded dioxygen-containing oxacycle **4a** together with desired product **3l**; the yields were 45% and 28%, respectively. Species **4a** assumes an anti-configuration (dr > 20 : 1) according to its ^1^H NOE spectra; this new compound was efficiently converted to compound **3l** in hot THF (eqn (3)), *via* a Kornblum–DeLaMare rearrangement.^22^

3

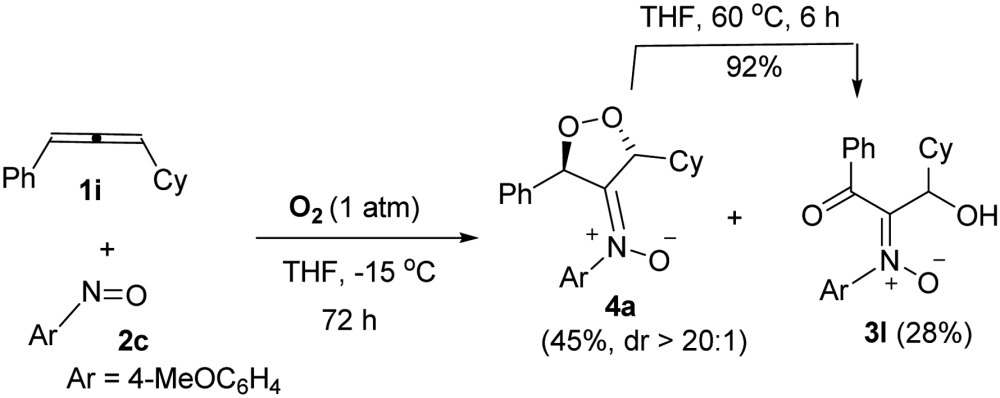




The kinetic stability of dioxygen-containing oxacycle **4a** is enhanced with a suitable steric environment. We further tested the reactions on various 1-aryl-1-methylallenes **1j–1m** with 4-methoxyphenylnitroso **2c** and O_2_ (1 bar) in THF (0 °C), generating dioxygen-containing compounds **4b–4e** (Ar = 4-RC_6_H_4_, R = H, Me, MeO, Br) in satisfactory yields (Table 3, entries 1–4). The molecular structure of compound **4b** was confirmed by its X-ray diffraction pattern.^17^ Various 1-aryl-3,3-dimethylallenes **1n–1q** (Ar = 4-RC_6_H_4_, R = H, Me, MeO, Br), electron-rich nitrosoarenes and O_2_ were also amenable to such cycloadditions, yielding desired compounds **4f–4m** in satisfactory yields (60–72%, entries 5–12) except **4k** in only 38% yield. This dioxygen cycloaddition was applicable to cyclohexylidene-derived phenylallene **1r**, affording compound **4n** in 66% yield (entry 13). Compounds **4** serve as the first examples of the cycloadditions of ground-state ^3^O_2_ with unsaturated hydrocarbons at low temperatures.

**Table 3:** [3 + 2]-Cycloadditions among O_2_, allenes and nitrosoarenes^*a*^ ^,^ ^*b*^

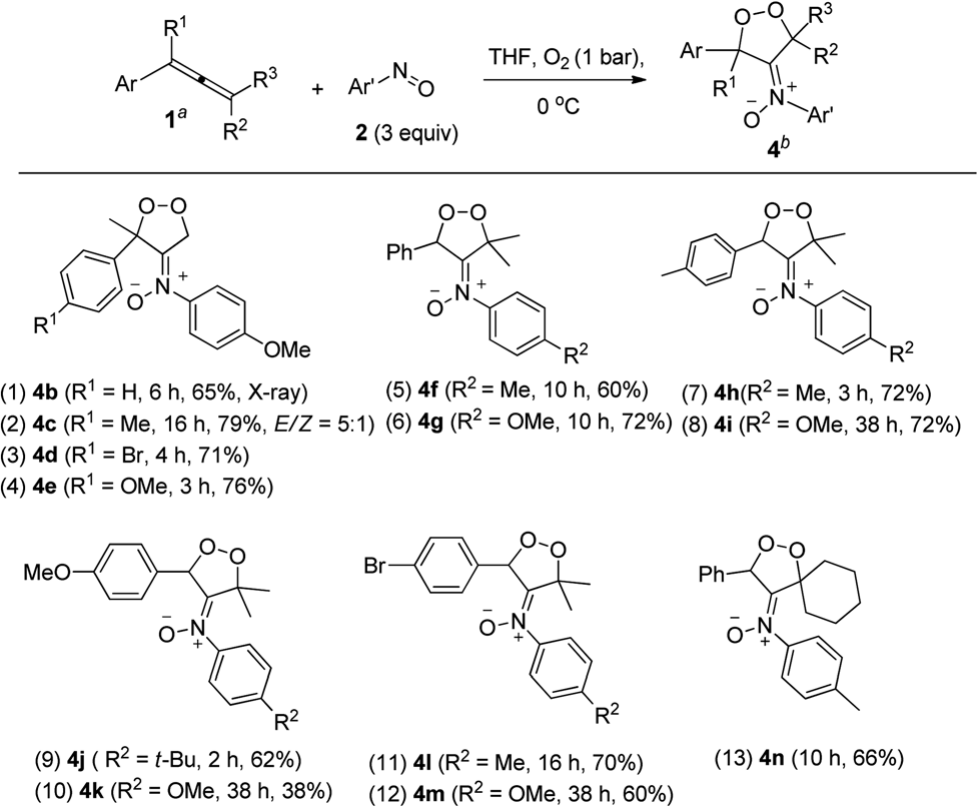

^*a*^[**1**] = 0.1 M.

^*b*^Product yields are reported after purification using a silica column.

An electron-deficient nitrosoarene is an inapplicable substrate, as shown by eqn (4). Under O_2_, the reaction of trisubstituted allene **1p** with 4-chlorophenylnitroso species **2f** in cold THF (0 °C) afforded nitroso-containing cycloadduct **5a** in 53% yield; the dioxygen-containing product, *ca.* 5%, was unstable for isolation (eqn (4)). In contrast, the same allene **1p** could deliver dioxygen-containing species **4j** and **4k** using electron-rich nitrosoarenes under the same conditions (entries 9–10, Table 3).
4

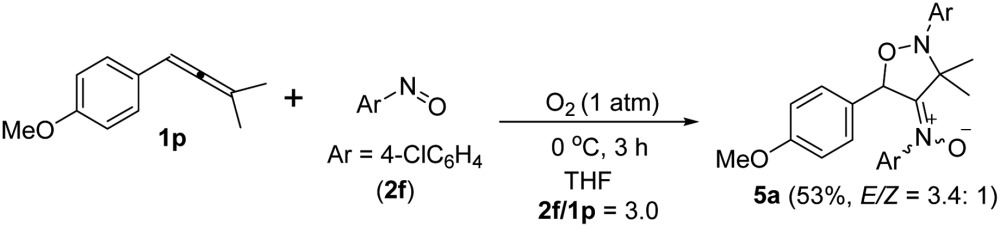



5

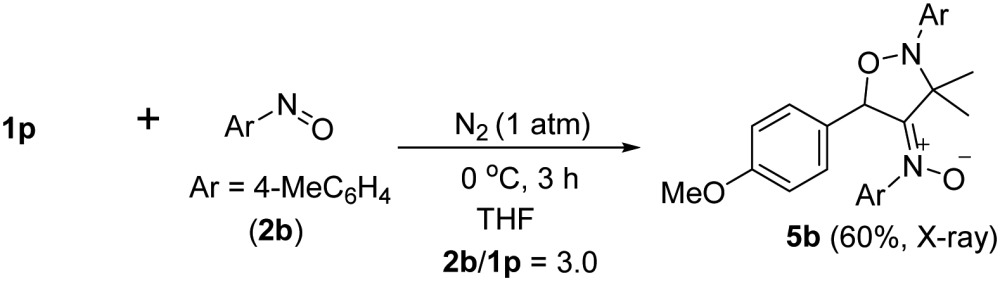




Under nitrogen, trisubstituted allene **1p** reacted with 4-methylphenylnitroso **2b** in cold THF to form nitroso-containing cycloadduct **5b** in 60% yield (eqn (5)). The stereochemistry and its E-configuration of this new compound was confirmed by its X-ray diffraction pattern.^17^ Such a new reaction represents a new and useful *O*,*N*,*N*-functionalization of allenes. A preliminary survey of the reaction scope is summarized in Table 4. We tested the reactions on 1,3-di- and 1,1,3-trisubstituted allenes **1g** and **1t** that reacted with nitroso-arenes (R = H, Cl, CO_2_Et) to afford nitroso-containing cycloadducts **5c–5g** in reasonable yields (58–83%). Furthermore, the anti-configuration of compound **5c** was determined by X-ray diffraction.^17^

**Table 4:** [3 + 2]-Cycloadditions among allenes and nitrosoarenes under N_2_ ^*a*^ ^,^ ^*b*^

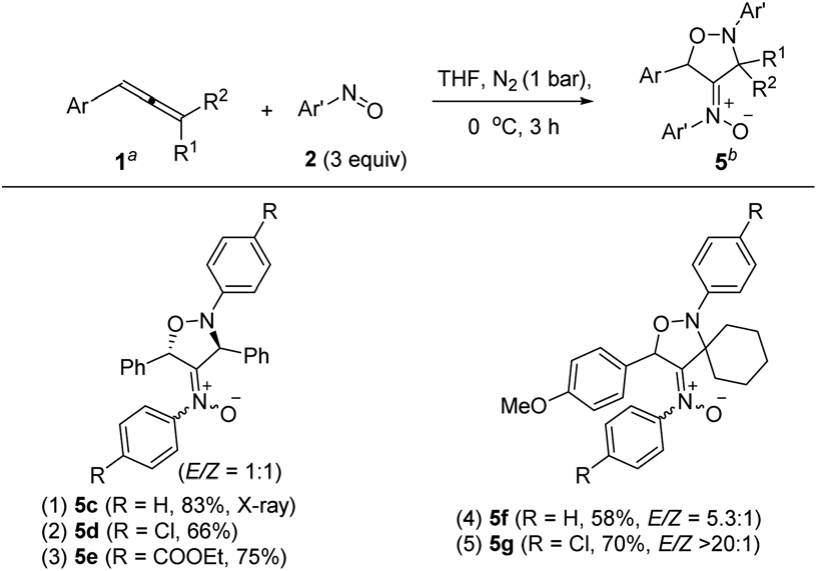

^*a*^[**1**] = 0.1 M.

^*b*^Product yields are reported after purification using a silica column.

Dioxygen-containing heterocycles **4** are readily reduced with Pd/C, H_2_ (1 atm) in MeOH (23 °C)^18^ to cleave their O–O bonds, satisfactorily yielding desired 1,3-dihydroxy-2-imine oxides **6**. These reductions highlight the utility of molecular oxygen to afford 1,3-dihydroxy-2-amino derivatives. Several instances of affording tertiary 1,3-alcohol derivatives are illustrated in eqn (6) and (7); their chemical yields exceed 65%. Under these reductions, the valuable nitrone functionalities of these acyclic 1,3-diols remain intact as indicated by their HRMS and ^13^C-NMR spectra.
6

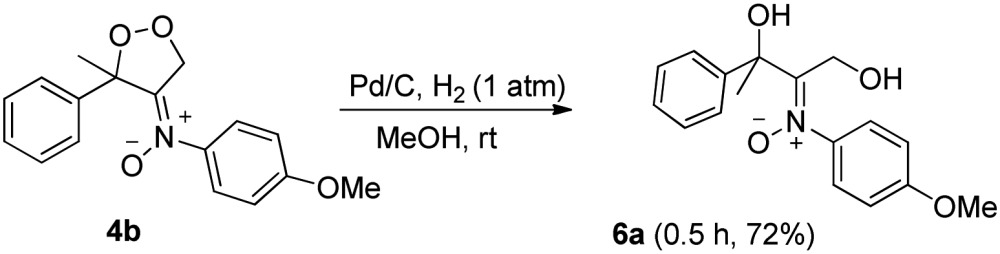



7

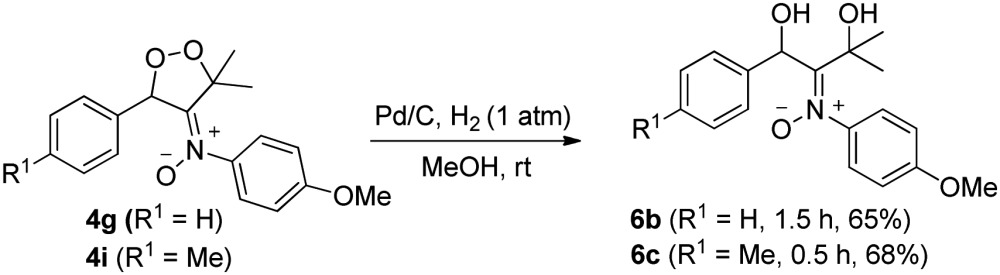




**Fig. 2:**
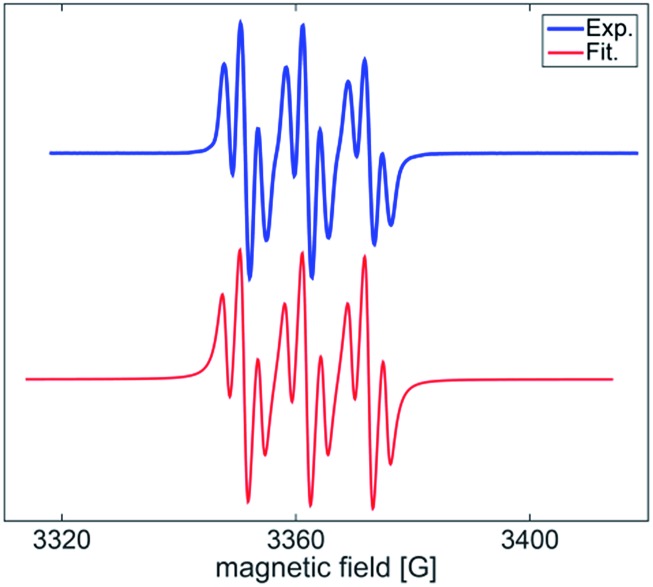
Observed and simulated EPR spectra.

The facile cycloadditions among allenes, nitrones and ground-state O_2_ are very astonishing because an intersystem crossing (ISC) must be involved for one key intermediate. To investigate the mechanism, we examined the reaction of 1-phenyl-3-cyclopropylallene **1s** with 4-methylphenylnitroso species **2b** under O_2_, yielding compound **3m** in 71% yield; this transformation did not induce cyclopropane cleavage because of the stability of the phenylallylic radical **A** (eqn (8)).^19^ We thus exclude the intermediacy of the dicarbon radical **A′**, although analogous carbon radicals were postulated for the *o*-quinodimethine species.^8^ We isolated compound **7** in 13% yield from the reaction of 1-phenylallene **1a** with PhNO (1.2 equiv.) and TEMPO (2 equiv.) under N_2_, indicating the formation of diradical intermediates (eqn (9)). We employed EPR to characterize the diradical species from a mixture of 3,3-dimethyl-1-phenylallene **1n** and nitrosobenzene **2a** in THF at 0 °C (0.5 h). Fig. 2 (top) shows the EPR signal of the diradical species; the intensity of this signal remains unchanged for 5 h under N_2_. The simulation analysis was performed using the EasySpin program.^20^ The satisfactory fit was achieved with a two-component simulation (bottom). The abundant component (70%) corresponds to nitrogen-centered diradicals (*g* = 2.00616, *a*_N_ = 10.7 G and 3.0 G).^21^ The minor component corresponds to a monoradical nitroxide with *a*_N_ = 10.7 G. Notably, when recorded at *T* < 130 K, the spectrum exhibits a well-known nitroxide rigid-limit lineshape in accordance with the above simulation result; the coupling of unpaired electrons with the nitrogen center is evident.
8

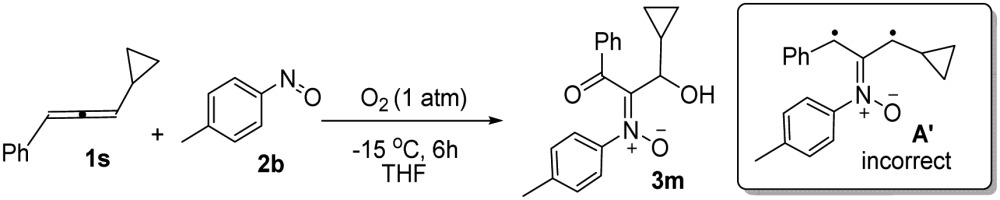



9

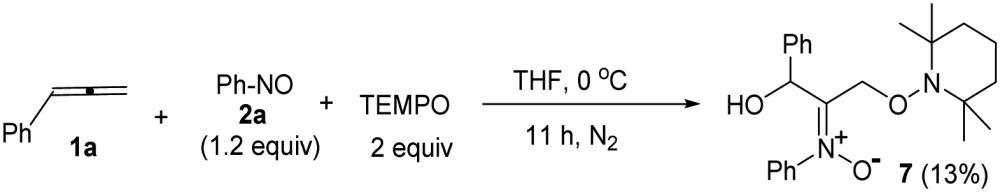




**Scheme 2:**
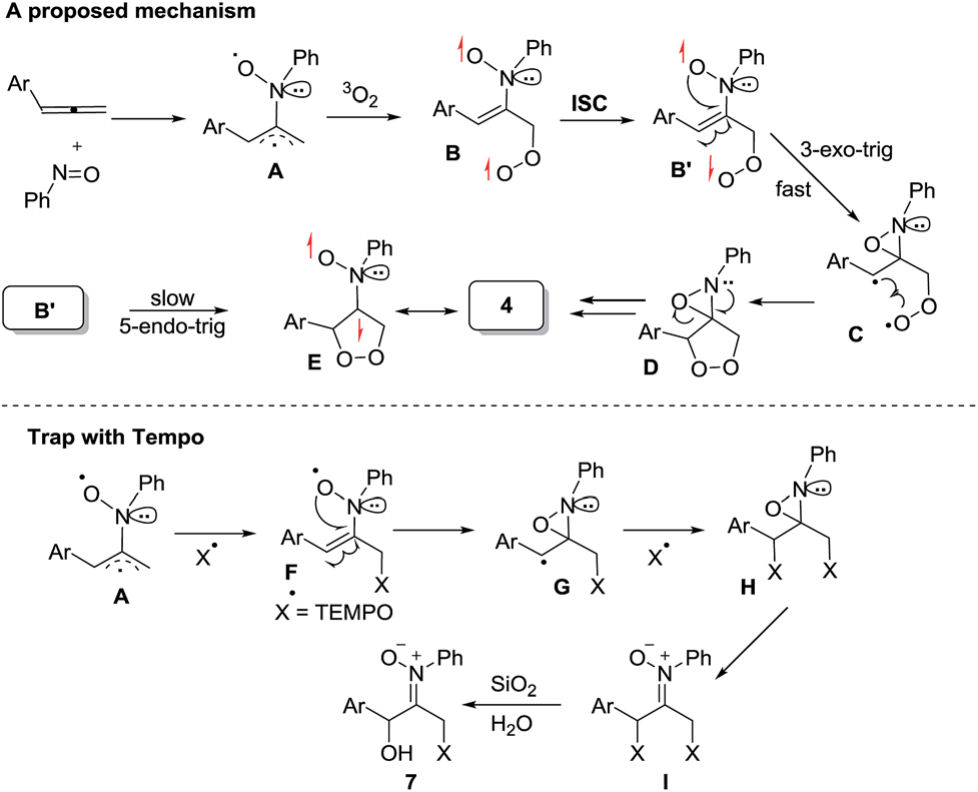
A plausible mechanism.

Scheme 2 depicts a plausible mechanism to rationalize the remarkable facility of such dioxygen annulations. We postulate that allene **1** reacts initially with nitrosobenzene to form 1,4-diradical species **A**, which is likely to be a major component, as detected in the EPR spectra; its nitroxy and allylic radicals are expected to couple with nitrogen in two magnitudes, *i.e. a*_N_ = 10.7 G and 3.0 G respectively.^21^ The capture of molecular dioxygen ^3^O_2_ by 1,4-diradical species **A** forms peroxy diradical **B** in a triplet state, as the two radical centers of species **B** are remote from each other, rendering an intersystem crossing (ISC) feasible. After a change of spin state, singlet-state diradical **B′** is expected to form primary 1,2-oxaziridine diradical **C** through a 3-*exo-trig* cyclization that is more feasible than an alternative 5-*endo-trig* cyclization.^23^ A final radical–radical coupling of resulting species **C** forms precursor **D**, and ultimately yields desired 1,2-dioxolanes **4**. This proposed path rationalizes the formation of compound **7** from the TEMPO experiment (eqn (9)) well. The trapping of the 1,4-biradical generates single radical species **F** that undergoes a rapid 3-*exo*-trig cyclization to form benzylic radical **G**. A second trapping of this species with the TEMPO radical is expected to yield species **I** that is prone to hydrolysis on a silica column to yield observed product **7**.

## Conclusions

Prior to this work, singlet state oxygen ^1^O_2_ failed to react with allenes to give useful oxygenated products.^7^ This study reports the first examples of metal-free [3 + 2]-cycloadditions among allenes, nitrosoarenes and ground-state ^3^O_2_ (1 bar) at low temperatures, efficiently yielding dioxygen-containing oxacycles.^24^ With less hindered 1-arylallene derivatives, the resulting oxacycles undergo skeletal rearrangement to 3-hydroxy-1-ketonyl-2-imine oxides. These transformations highlight a cheap, efficient and clean synthesis of 1,3-dihydroxy-2-amino derivatives. Our experimental data indicate that an initial attack of a nitrosoarene at an allene generates a diradical species that is detectable with EPR. We envisage that the concept of nitrosoarenes as diradical precursors will inspire new synthetic concepts.
