# Supplementary material for: Ground-state dioxygen undergoes metal-free [3 + 2]-annulations with allenes and nitrosoarenes under ambient conditions
Source: Chem Sci. 2017 May 24;8(8):5482–7. doi: 10.1039/c7sc01770g (PMC5613744; doi:10.1039/c7sc01770g)
Supplement: Supplementary file 1 [file SC-008-C7SC01770G-s001.pdf]

Electronic Supplementary Material (ESI) for Chemical Science.
This journal is © The Royal Society of Chemistry 2017

Supporting Information

Ground-State Dioxygen Undergoes Metal-Free [3+2]-Annulations with Allenes and Nitrosoarenes Under Ambient Conditions

Jinxian Liu,[a,b†] Manisha Skaria,[a†] Pankaj Sharma,[a] Yun-Wei Chiang,[a] and Rai-Shung Liu*[a]

[a] Department of Chemistry, National Tsing-Hua University, Hsinchu, Taiwan, ROC
[b] College of Chemistry & Materials Science, Longyan University, Fujian, China
----------------e-mail: rsliu@mx.nthu.edu.tw

TABLE OF CONTENTS

General Experimental 2

General procedures and spectral data 3

EPR analysis 21

References 22

Crystallographic data 23

^{1}H and ^{13}C NMR spectra 58

General Experimental

Unless otherwise noted, all reactions were carried out under a O_2 atmosphere in reaction tube. Tetrahydrofuran was dried with sodium benzophenone and distilled before use. Reagents were purchased from commercial sources and used without purification, unless otherwise stated. Reactions were magnetically stirred and monitored by thin layer chromatography carried out on 0.25 mm E. Merck silica gel plate (60f- 254) using UV light as visualizing agents and/or potassium permanganate ($KMnO_4$). ^{1}H NMR and ^{13}C NMR spectra were recorded on a Bruker 400, Varian 400 MHz and a Bruker 600 MHz spectrometers using chloroform-*d* ($CDCl_3$) as the internal standard. Chemical shifts are reported in parts per million (ppm). Multiplicities are indicated by s (singlet), d (doublet), t (triplet), q (quartet), and m (multiplet). Coupling constants J are reported in Hertz (Hz). The allene substrates **1a-1e** were prepared according to literature[1], **1f-1i**, **1s** were prepared according to literature[2], **1j-1m** were prepared according to literature[3], and **1n-1t** were prepared according to literature[4]; the nitrosoarene substrates **1b-1g** were prepared according to literature[5].

Table 1 Lists of the substrates **1** and **2**

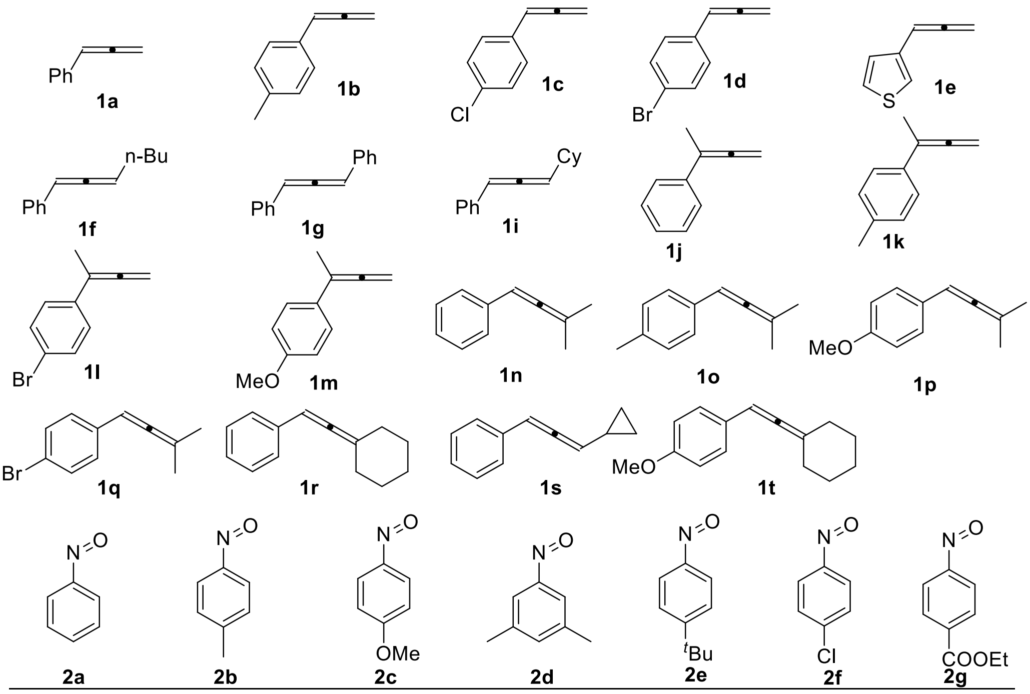

Typical procedure for the preparation of 3a.

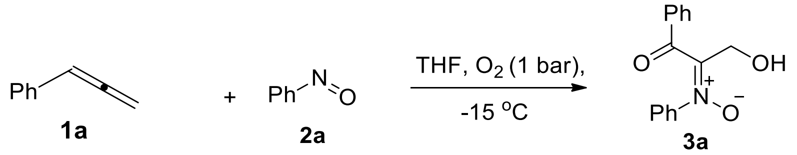

A 100-mL flask was charged with nitrosobenzene **2a** (964.0 mg, 9.0 mmol), then evacuated and backfilled with O_2 balloon. To this solid was added anhydrous THF (15 mL), and the resulting mixture was cooled to -15 ^{o}C. To this mixture was added a THF solution (15 mL) of 1-phenylallene **1a** (348.0 mg, 3.0 mmol, 1 equiv), and the mixture was stirred for 2 h. The solution was concentrated under reduced pressure, and the resulting mixture was purified by a silica column with hexane/diethyl ether (1:3) to get afford compound **3a** (482.0 mg, 1.90 mmol, 63%) as light yellow oil.

^{1}H NMR (600 MHz, $CDCl_3$): δ 7.75 (d, *J* = 7.8 Hz, 2H), 7.51 (t, *J* = 7.2 Hz, 1H), 7.38 (t, *J* = 7.8 Hz, 2H), 7.32 (d, *J* = 7.8 Hz, 2H), 7.28 (t, *J* = 2.4 Hz, 1H), 7.24 (d, *J* = 7.8 Hz, 2H), 4.75 (s, 2H); **^{13}C NMR** (150 MHz, $CDCl_3$): δ 189.1, 149.7, 146.1, 135.3, 134.3, 130.7, 129.2, 129.1, 128.9, 123.7, 60.3; **HRMS** (ESI, m/z) calcd. for $C_{15}H_{14}N_1O_3$ $[M+H]^+$ calc.: 256.0974 ; found: 256.0975.

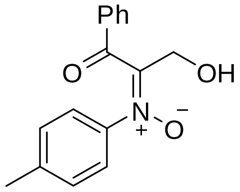

3b

Synthesis of **3b**: Following the typical procedure of **3a, 3b** was isolated by a silica column (549.0 mg, 2.05 mmol, 68%).

^{1}H NMR (600 MHz, $CDCl_3$): δ 7.76 (dd, J_1 = 7.9 Hz, J_2 = 0.6 Hz, 2H), 7.50 (t, *J* = 7.8 Hz, 1H), 7.38 (t, *J* = 7.8 Hz, 2H), 7.21 (d, *J* = 8.4 Hz, 2H), 7.01 (d, *J* = 8.4 Hz, 2H), 4.72 (s, 2H), 2.23 (s, 3H); **^{13}C NMR** (150 MHz, $CDCl_3$): δ 189.2, 149.1, 143.9, 141.2, 135.4, 134.2, 129.6, 129.2, 128.9, 123.5, 60.3, 21.1; **HRMS** (ESI, m/z) calcd. for $C_{16}H_{16}N_1O_3$ $[M+H]^+$ calc.: 270.1130 ; found: 270.1132.

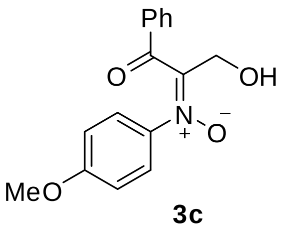

3c

Synthesis of **3c**: Following the typical procedure of **3a, 3c** was isolated by a silica column (462.0 mg, 1.63 mmol, 54%).

^{1}H NMR (600 MHz, $CDCl_3$): δ 7.74 (dd, J_1= 8.1 Hz, J_2= 1.0 Hz, 2H), 7.50 (t, J = 7.5 Hz, 1H), 7.37 (t, J = 8.0 Hz, 2H), 7.27 (dd, J_1 = 6.9 Hz, J_2 = 2.1 Hz, 2H), 6.70 (dd, J_1 = 6.9 Hz, J_2 = 2.1 Hz, 2H), 4.72 (s, 2H), 3.70 (s, 3H); **^{13}C NMR** (150 MHz, $CDCl_3$): δ 189.4, 161.1, 148.6, 139.6, 135.5, 134.2, 129.1, 128.9, 125.3, 114.1, 60.4, 55.5; HRMS (ESI, m/z) calcd. for $C_{16}H_{14}N_1O_4$ $[M\text{-}H]^-$ calc.: 284.0928 ; found: 284.0939.

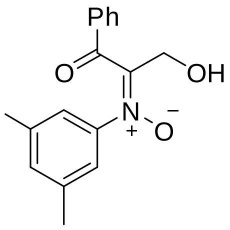

3d

Synthesis of **3d**: Following the typical procedure of **3a, 3d** was isolated by a silica column (493.0 mg, 1.75 mmol, 58%).

^{1}H NMR (600 MHz, $CDCl_3$): δ 7.75 (dd, J_1 = 8.2 Hz, J_2 = 1.3 Hz, 2H), 7.50 (t, J = 7.5 Hz, 1H), 7.38 (t, J = 7.5 Hz, 2H), 6.93 (s, 2H), 6.87 (s, 1H), 4.73 (s, 2H), 2.17 (s, 6H); **^{13}C NMR** (150 MHz, $CDCl_3$): δ 189.3, 148.9, 146.2, 139.2, 134.1, 132.3, 130.3, 129.0, 128.8, 121.4, 60.4, 21.0; HRMS (ESI, m/z) calcd. for $C_{17}H_{17}NNaO_3$ $[M+Na]^+$ calc.: 306.1101 ; found: 306.1095.

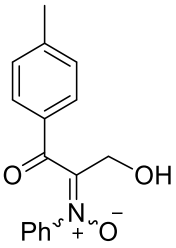

3e (*E*/*Z* = 2.5: 1)

Synthesis of **3e**: Following the typical procedure of **3a, 3e** was isolated by a silica column (598.0 mg, 2.20 mmol, 74%).

^{1}H NMR (400 MHz, $CDCl_3$): δ 7.69 (d, J = 8.4 Hz, 2H), 7.34 (d, J = 8.4 Hz, 2H), 7.32-7.21 (m, 3H), 7.20 (d, J = 7.8 Hz, 2H), 4.73 (s, 2H), 2.36 (s, 3H) (*E-form*); selected peak of *Z-form*: δ 7.96(d, J = 8.0 Hz, 2H),4.79(s, 2H), 2.43(s, 3H); **^{13}C NMR** (100 MHz, $CDCl_3$): δ 188.5, 150.0, 146.0, 132.7, 130.6, 129.3, 129.0, 123.6, 116.0, 60.3, 21.0 (*E-form*); **HRMS** (ESI, m/z) calcd. for $C_{16}H_{16}N_1O_3$ $[M+H]^+$ calc.: 270.1130 ; found: 270.1133.

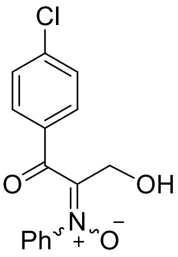

3f (*Z/E* =7.4:1)

Synthesis of **3f**: Following the typical procedure of **3a, 3f** was isolated by a silica column (435.0 mg, 1.50 mmol, 50%).

^{1}H NMR (600 MHz, $CDCl_3$): δ 7.69 (d, *J* = 8.4 Hz, 2H), 7.32 (d, *J* = 7.8 Hz, 2H), 7.29-7.27 (m, 3H), 7.25 (t, *J* = 7.2 Hz, 2H), 4.73 (s, 3H) (*E-form*); selected peak of *Z-form*: δ 8.02 (d, *J* = 8.4 Hz, 2H), 7.47 (d, *J* = 9.0 Hz, 2H), 4.78(s, 2H); **^{13}C NMR** (150 MHz, $CDCl_3$): δ 188.0, 149.1, 146.1, 140.9, 133.8, 131.7, 130.9, 130.5, 129.4, 123.7, 60.1 (*E-form*); **HRMS** (ESI, m/z) calcd. for $C_{15}H_{13}Cl_1N_1O_3$ $[M+H]^+$ calc.: 290.0584 ; found: 290.0586.

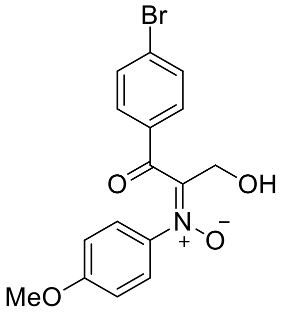

3g

Synthesis of **3g**: Following the typical procedure of **3a, 3g** was isolated by a silica column (765.0 mg, 2.10 mmol, 70%).

^{1}**H NMR** (600 MHz, $CDCl_3$): δ 7.60 (d, J = 8.6 Hz, 2H), 7.51 (d, J = 8.6 Hz, 2H), 7.26 (d, J = 9.0 Hz, 2H), 6.72 (d, J = 9.0 Hz, 2H), 4.70 (s, 2H), 3.73 (s, 3H); ^{13}**C NMR** (150 MHz, $CDCl_3$): δ 188.4, 161.3, 148.0, 139.5, 134.4, 132.3, 130.4, 129.6, 125.3, 114.2, 60.3, 55.6; **HRMS** (ESI, m/z) calcd. for $C_{16}H_{15}BrNO_4$ $[M+H]^+$ calc.: 364.0179; found: 364.0156.

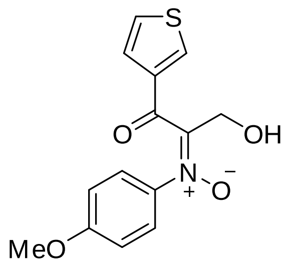

3h

Synthesis of **3h**: Following the typical procedure of **3a, 3h** was isolated by a silica column (525.0 mg, 1.80 mmol, 60%).

^{1}**H NMR** (600 MHz, $CDCl_3$): δ 8.10-8.05 (m, 1H), 7.38 (dd, J_1 = 5.1 Hz, J_2 = 1.2 Hz, 1H), 7.31 (d, J = 9.0 Hz, 2H), 7.27-7.25 (m, 1H), 6.76 (d, J = 9.0 Hz, 2H), 4.72 (s, 2H), 3.74 (s, 3H); ^{13}**C NMR** (150 MHz, $CDCl_3$): δ 182.4, 161.2, 148.7, 140.4, 139.6, 135.1, 127.3, 126.9, 125.1, 114.2, 60.3, 55.6; **HRMS** (ESI, m/z) calcd. for $C_{14}H_{14}NO_4S$ $[M+H]^+$ calc.: 292.0638; found: 292.0637.

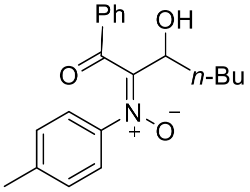

3i

Synthesis of **3i**: Following the typical procedure of **3a, 3i** was isolated by a silica column (645.0 mg, 2.00 mmol, 66%).

^{1}**H NMR** (600 MHz, $CDCl_3$): δ 7.73 (d, J = 7.8 Hz, 2H), 7.48 (t, J = 7.8 Hz, 1H), 7.35 (t, J = 7.8 Hz, 2H), 7.18 (d, J = 7.8 Hz, 2H), 7.01 (d, J = 7.8 Hz, 2H), 4.80-4.76 (m, 1H), 2.20(s, 3H), 2.00-1.90 (m, 2H), 1.60-1.50 (m, 1H), 1.40-1.20 (m, 3H), 0.87(t, J =7.2 Hz, 3H); ^{13}**C NMR** (150 MHz, $CDCl_3$): δ 189.8, 152.2, 143.9, 141.0, 135.7,

134.2, 129.6, 129.2, 128.8, 123.8, 71.2, 32.3, 27.8, 22.4, 21.1, 13.9; **HRMS** (ESI, m/z) calcd. for $C_{20}H_{24}N_1O_3$ $[M+H]^+$ calc.: 326.1756; found: 326.1754.

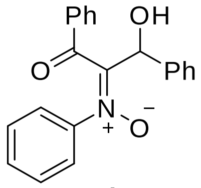

3j

Synthesis of **3j**: Following the typical procedure of **3a, 3j** was isolated by a silica column (537.0 mg, 1.62 mmol, 54%).

^{1}H NMR (600 MHz, $CDCl_3$): δ 7.60-7.51 (m, 4H), 7.40-7.35 (m, 1H), 7.31-7.11 (m, 10H), 6.04 (s, 1H); **^{13}C NMR** (150 MHz, $CDCl_3$): δ 189.7, 151.0, 146.1, 138.3, 135.8, 133.9, 130.7, 129.0, 128.5, 128.4, 128.2, 126.3, 124.0, 72.8; **HRMS** (ESI, m/z) calcd. for $C_{21}H_{18}N_1O_3$ $[M+H]^+$ calc.: 332.1287; found: 332.1281.

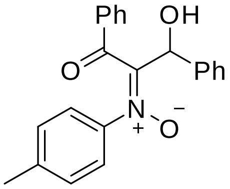

3k

Synthesis of **3k**: Following the typical procedure of **3a, 3k** was isolated by a silica column (642.0 mg, 1.85 mmol, 62%).

^{1}H NMR (600 MHz, $CDCl_3$): δ 7.60-7.51 (m, 4H), 7.43-7.40 (m, 1H), 7.35-7.21 (m, 5H), 7.18 (d, J = 8.4 Hz, 2H), 6.96 (d, J = 8.4 Hz, 2H), 5.98 (s, 1H), 2.19 (s, 3H); **^{13}C NMR** (150 MHz, $CDCl_3$): δ 189.8, 150.6, 144.0, 141.3, 138.4, 135.9, 133.9, 129.6, 129.1, 128.6, 128.5, 128.2, 126.3, 123.9, 73.0, 21.1; **HRMS** (ESI, m/z) calcd. for $C_{22}H_{20}N_1O_3$ $[M+H]^+$ calc.: 346.1443 ; found: 346.1443.

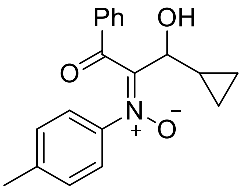

3m

Synthesis of **3m**: Following the typical procedure of **3a, 3m** was isolated by a silica column (660.0 mg, 2.13 mmol, 71%).

¹H NMR (600 MHz, $CDCl_3$): δ 7.74 (d, *J* = 8.2 Hz, 2H), 7.48 (t, *J* = 8.6 Hz, 1H), 7.35 (d, *J* = 8.2 Hz, 2H), 7.24-7.20 (m, 2H), 6.98 (d, *J* = 8.0 Hz, 2H), 4.11 (d, *J* = 9.1 Hz, 1H), 2.12 (s, 3H), 1.42-1.38 (m, 1H), 0.90-0.20 (m, 4H); **¹³C NMR** (150 MHz, $CDCl_3$): δ 189.7, 151.8, 143.8, 141.1, 135.9, 134.2, 129.6, 129.2, 128.8, 123.8, 75.1, 21.1, 13.4, 3.9, 2.7; **HRMS** (ESI, m/z) calcd. for $C_{19}H_{20}N_1O_3$ $[M+H]^+$ calc.: 310.1443; found: 310.1441.

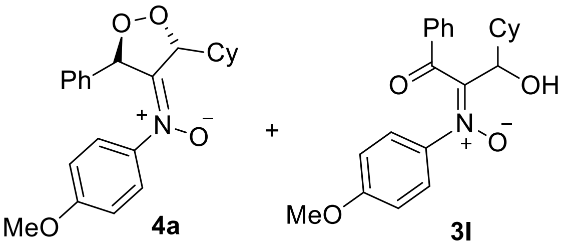

4a + **3l**

A 100-mL flask was charged with nitrosobenzene **2c** (1.23 g, 9.0 mmol), then evacuated and backfilled with O_2 balloon. To this solid was added anhydrous THF (15 mL), and the resulting mixture was cooled to 0 °C. To this mixture was added a THF solution (15 mL) of (3-cyclohexylpropa-1,2-dien-1-yl)benzene **1l** (595.0 mg, 3.0 mmol, 1 equiv), and the mixture was stirred for 72 h. The solution was concentrated under reduced pressure, and the resulting mixture was purified by a silica column with hexane/diethyl ether (1:3) to get afford compound **4a** (496.0 mg, 1.35 mmol, 45%) and **3l** (309.0 mg, 1.90 mmol, 28%) as oil. To a solution of **4a** (367.0 mg, 1.0 mol) in THF (10 ml) was heated to 60 °C for 6 h. The resulting mixture was purified by a silica column to get **3l** (338.0 mg, 0.92 mol, 92%) as oil.

4a

¹H NMR (600 MHz, $CDCl_3$): δ 7.17 (t, *J* = 7.4 Hz, 1H), 7.11 (t, *J* = 7.8 Hz, 2H), 6.98 (d, *J* = 8.9 Hz, 2H), 6.92 (d, *J* = 8.5 Hz, 2H), 6.64 (d, *J* = 8.9 Hz, 2H), 5.54 (d, *J* = 1.9 Hz, 1H), 5.37-5.35 (m, 1H), 3.73 (s, 3H), 2.70-2.65 (m, 1H), 1.85-1.75 (m, 4H), 1.70-1.45(m, 2H), 1.40-1.30 (m, 2H), 1.25-1.20(m, 2H); **¹³C NMR** (150 MHz, $CDCl_3$): δ 160.4, 157.7, 139.0, 134.6, 129.2, 128.4, 128.3, 124.4, 114.1, 86.7, 83.3, 55.6, 37.6, 29.5, 27.8, 26.5, 26.2, 26.0; **HRMS** (ESI, m/z) calcd. for $C_{22}H_{26}N_1O_4$ $[M+H]^+$ calc.: 368.1862 ; found: 368.1862.

NOE for compound 4a:

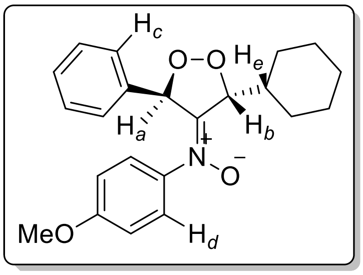

S. No	Irradiation	Intensity increase %
1	H_a (5.54)	H_c (6.98, 8.69%), H_d(6.92, 3.01%)
2	H_b (5.36)	H_c (6.98, 1.88%), H_e (2.67, 3.36%)

3l

^{1}H NMR (600 MHz, $CDCl_3$): δ 7.75 (t, J = 7.2 Hz, 2H), 7.48 (t, J = 7.2 Hz, 1H), 7.36 (t, J = 7.8 Hz, 2H), 7.24 (d, J = 7.8 Hz, 2H), 6.66 (d, J = 7.8 Hz, 2H), 5.71 (d, J = 10.8 Hz, 1H), 4.35-4.41(m, 1H), 3.69 (s, 3H), 2.30-2.10 (m, 2H), 1.80-1.60 (m, 3H), 1.30-1.00(m, 6H); **^{13}C NMR** (150 MHz, $CDCl_3$): δ 189.9, 161.0, 151.9, 140.0, 135.6, 134.1, 129.2, 128.8, 125.5, 114.1, 55.5, 41.5, 29.9, 29.4, 26.3, 25.9; **HRMS** (ESI, m/z) calcd. for $C_{22}H_{26}N_1O_4$ $[M+H]^+$ calc.: 368.1862 ; found: 368.1863.

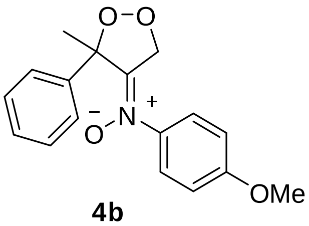

4b

Synthesis of **4b**: Following the typical procedure of **4a, 4b** was isolated by a silica column (584.0 mg, 1.95 mmol, 65%).

^{1}H NMR (600 MHz, $CDCl_3$): δ 7.75 (t, J = 7.6 Hz, 2H), 7.41-7.30 (m, 5H), 6.92 (d, J = 8.9 Hz, 2H), 4.75 (q, J = 12.8 Hz, 2H), 3.82 (s, 3H), 2.12 (s, 3H); **^{13}C NMR** (150 MHz, $CDCl_3$): δ 160.7, 154.9, 140.0, 139.0, 128.7, 128.0, 126.2, 123.5, 114.6, 87.7, 72.1, 55.6, 19.6; **HRMS** (ESI, m/z) calcd. for $C_{17}H_{18}N_1O_4$ $[M+H]^+$ calc.: 300.1236 ; found: 300.1234.

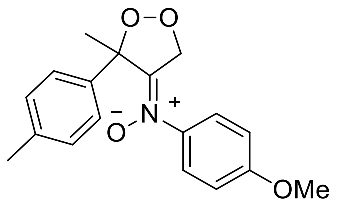

4c (*E*/*Z* = 5:1)

Synthesis of **4c**: Following the typical procedure of **4a, 4c** was isolated by a silica column (743.0 mg, 2.35 mmol, 79%).

^{1}H NMR (600 MHz, $CDCl_3$): δ 7.61 (t, J = 7.0 Hz, 2H), 7.36 (d, J = 7.1 Hz, 2H), 7.19 (d, J = 7.7 Hz, 2H), 6.91 (d, J = 7.3 Hz, 2H), 4.75 (s, 2H), 3.82 (s, 3H), 2.34 (s, 3H), 2.10 (s, 3H) (*E-form*); selected peak of *Z-form*: δ 7.84 (d, J = 7.2 Hz, 2H), 2.56 (s, 3H), 2.39 (s, 3H); **^{13}C NMR** (150 MHz, $CDCl_3$): δ 160.8, 155.3, 140.0, 138.6, 135.7, 129.3, 126.2, 123.5, 114.6, 87.7, 72.2, 55.6, 21.2, 19.5; **HRMS** (ESI, m/z) calcd. for $C_{18}H_{20}N_1O_4$ $[M+H]^+$ calc.: 314.1392; found: 314.1393.

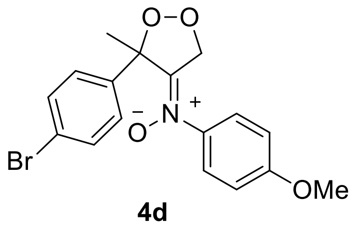

4d

Synthesis of **4d**: Following the typical procedure of **4a, 4d** was isolated by a silica column (806.0 mg, 2.15 mmol, 71%).

^{1}H NMR (400 MHz, $CDCl_3$): δ 7.67 (t, J = 8.8 Hz, 2H), 7.50 (d, J = 8.4 Hz, 2H), 7.35 (d, J = 8.8 Hz, 2H), 6.92 (d, J = 8.8 Hz, 2H), 4.76 (dd, J_1 = 36 Hz, J_2 =13.2 Hz, 2H), 3.82 (s, 3H), 2.08 (s, 3H); **^{13}C NMR** (100 MHz, $CDCl_3$): δ 160.8, 154.3, 140.0, 138.3, 131.6, 123.4, 122.8, 114.6, 87.3, 72.0, 55.7, 19.6; **HRMS** (ESI, m/z) calcd. for $C_{17}H_{17}BrNO_4$ $[M+H]^+$ calc.: 378.0335; found: 378.0337.

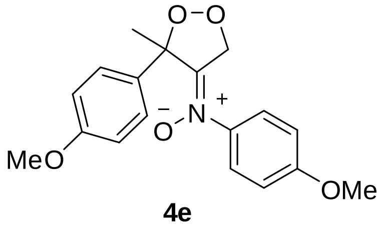

4e

Synthesis of **4e**: Following the typical procedure of **4a, 4e** was isolated by a silica column (750.0 mg, 2.30 mmol, 76%).

1H NMR (400 MHz, $CDCl_3$): δ 7.65 (d, *J* = 8.4 Hz, 2H), 7.37 (t, *J* = 8.8 Hz, 2H), 6.95-6.85 (m, 4H), 4.76 (s, 2H), 3.82 (s, 3H), 3.80 (s, 3H), 2.10 (s, 3H); **13C NMR** (100 MHz, $CDCl_3$): δ 160.7, 159.8, 155.2, 140.0, 130.5, 127.7, 123.5, 114.6, 113.9, 87.5, 72.2, 55.6, 55.3, 19.5; **HRMS** (ESI, m/z) calcd. for $C_{18}H_{20}N_1O_5$ $[M+H]^+$ calc.: 330.1342; found: 330.1340.

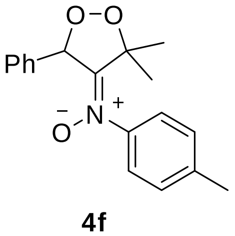

4f

Synthesis of **4f**: Following the typical procedure of **4a, 4f** was isolated by a silica column (535.0 mg, 1.80 mmol, 60%).

1H NMR (600 MHz, $CDCl_3$): δ 7.21 (t, *J* = 7.6 Hz, 1H), 7.13 (t, *J* = 7.8 Hz, 2H), 7.00-6.90 (m, 4H), 6.90 (d, *J* = 8.3 Hz, 2H), 5.63 (s, 1H), 2.25 (s, 3H), 1.92 (s, 3H), 1.86 (s, 3H); **13C NMR** (150 MHz, $CDCl_3$): δ 159.2, 143.5, 140.0, 133.8, 129.5, 129.3, 128.6, 128.4, 122.1, 85.9, 84.4, 21.1, 20.5, 19.9; **HRMS** (ESI, m/z) calcd. for $C_{18}H_{20}N_1O_3$ $[M+H]^+$ calc.: 298.1443 ; found: 298.1444.

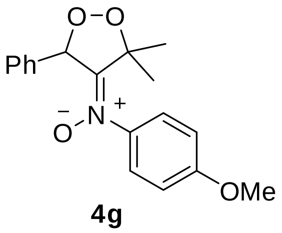

4g

Synthesis of **4g**: Following the typical procedure of **4a, 4g** was isolated by a silica column (675.0 mg, 2.15 mmol, 72%).

1H NMR (600 MHz, $CDCl_3$): δ 7.19 (t, *J* = 7.5 Hz, 1H), 7.14 (t, *J* = 7.8 Hz, 2H), 6.98-6.92 (m, 4H), 6.61 (d, *J* = 8.9 Hz, 2H), 5.63 (s, 1H), 3.71 (s, 3H), 1.91 (s, 3H), 1.84 (s, 3H); **13C NMR** (150 MHz, $CDCl_3$): δ 160.2, 159.3, 139.2, 133.7, 129.3, 128.6, 128.5, 124.2, 114.0, 85.9, 84.5, 55.5, 20.5, 19.8; **HRMS** (ESI, m/z) calcd. for $C_{18}H_{20}N_1O_4$ $[M+H]^+$ calc.: 314.1392; found: 314.1387.

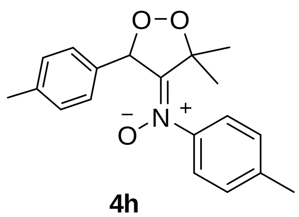

4h

Synthesis of **4h**: Following the typical procedure of **4a, 4h** was isolated by a silica column (672.0 mg, 2.15 mmol, 72%).

^{1}H NMR (600 MHz, $CDCl_3$): δ 6.98-6.85 (m, 6H), 6.81 (d, *J* = 8.0 Hz, 2H), 5.58 (s, 1H), 2.24 (s, 6H), 1.90 (s, 3H), 1.84 (s, 3H); **^{13}C NMR** (150 MHz, $CDCl_3$): δ 159.3, 143.5, 139.9, 139.3, 130.8, 129.4, 129.1, 128.5, 122.7, 85.8, 84.2, 21.1, 21.0, 20.4, 19.9; **HRMS** (ESI, m/z) calcd. for $C_{19}H_{22}N_1O_3$ $[M+H]^+$ calc.: 312.1600; found: 312.1596.

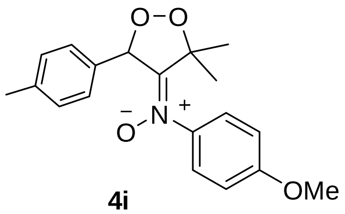

4i

Synthesis of **4i**: Following the typical procedure of **4a, 4i** was isolated by a silica column (707.0 mg, 2.15 mmol, 72%).

^{1}H NMR (600 MHz, $CDCl_3$): δ 7.00-6.90 (m, 4H), 6.84 (d, *J* = 8.0 Hz, 2H), 6.62 (d, *J* = 8.9 Hz, 2H), 5.60 (s, 1H), 3.72 (s, 3H), 2.25 (s, 3H), 1.90 (s, 3H), 1.84 (s, 3H); **^{13}C NMR** (150 MHz, $CDCl_3$): δ 160.2, 159.3, 139.3, 130.8, 129.2, 128.5, 124.3, 114.0, 85.8, 84.3, 55.5, 21.1, 20.5, 19.8; **HRMS** (ESI, m/z) calcd. for $C_{19}H_{22}N_1O_4$ $[M+H]^+$ calc.: 328.1549; found: 328.1546.

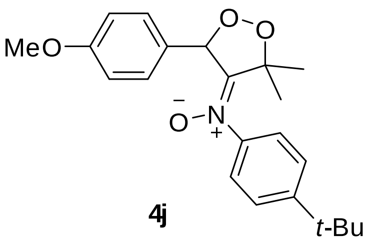

4j

Synthesis of **4j**: Following the typical procedure of **4a, 4j** was isolated by a silica column (688.0 mg, 1.85 mmol, 62%).

^{1}H NMR (600 MHz, $CDCl_3$): δ 7.13 (d, *J* = 8.3 Hz, 2H), 6.90 (d, *J* = 8.4 Hz, 2H), 6.79 (d, *J* = 8.6 Hz, 2H), 6.58 (d, *J* = 8.6 Hz, 2H), 5.58 (s, 1H), 3.68 (s, 3H), 1.90 (s, 3H), 1.84 (s, 3H), 1.20 (s, 9H); **^{13}C NMR** (150 MHz, $CDCl_3$): δ 160.1, 159.6, 153.1, 143.2, 130.1, 125.8, 125.2, 122.5, 113.9, 85.9, 84.3, 55.2, 34.7, 31.1, 20.7, 19.7; **HRMS** (ESI, m/z) calcd. for $C_{22}H_{28}N_1O_4$ $[M+H]^+$ calc.: 370.2018; found: 370.2014.

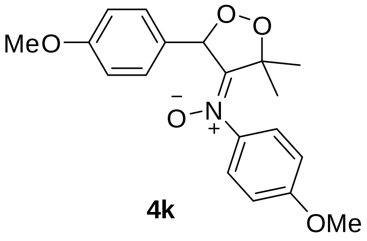

4k

Synthesis of **4k**: Following the typical procedure of **4a, 4k** was isolated by a silica column (390.0 mg, 1.15 mmol, 38%).

^{1}H NMR (600 MHz, $CDCl_3$): δ 6.97 (d, *J* = 8.6 Hz, 2H), 6.88 (d, *J* = 8.6 Hz, 2H), 6.70-6.60 (m, 4H), 5.60 (s, 1H), 3.73 (s, 3H), 1.90 (s, 3H), 1.84 (s, 3H); **^{13}C NMR** (150 MHz, $CDCl_3$): δ 160.3, 160.2, 159.3, 139.3, 130.0, 125.7, 124.3, 114.0, 113.9, 85.9, 84.2, 55.5, 55.3, 20.6, 19.8; **HRMS** (ESI, m/z) calcd. for $C_{19}H_{22}N_1O_5$ $[M+H]^+$ calc.: 344.1498; found: 344.1497.

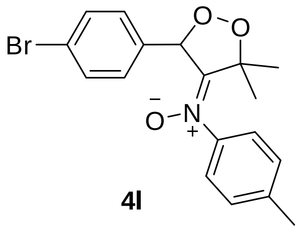

4l

Synthesis of **4l**: Following the typical procedure of **4a, 4l** was isolated by a silica column (790.0 mg, 2.10 mmol, 70%).

^{1}H NMR (600 MHz, $CDCl_3$): δ 7.26 (d, *J* = 9.0 Hz, 2H), 6.98 (d, *J* = 7.8 Hz, 2H), 6.90 (d, *J* = 8.4 Hz, 2H), 6.79 (d, *J* = 8.4 Hz, 2H), 5.58 (s, 1H), 2.27 (s, 3H), 1.87 (s, 3H), 1.82 (s, 3H); **^{13}C NMR** (150 MHz, $CDCl_3$): δ 158.9, 143.4, 140.4, 133.1, 131.6, 130.0, 123.6, 122.6, 85.9, 83.5, 21.1, 20.4, 19.9; **HRMS** (ESI, m/z) calcd. for $C_{18}H_{19}{}^{79}Br_1N_1O_3$ $[M+H]^+$ calc.: 376.0548; found: 376.0547.

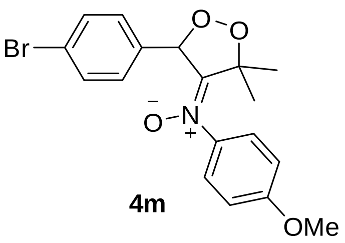

4m

Synthesis of **4m**: Following the typical procedure of **4a, 4m** was isolated by a silica column (706.0 mg, 1.80 mmol, 60%).

^{1}H NMR (600 MHz, $CDCl_3$): δ 7.29 (d, *J* = 8.4 Hz, 2H), 6.98 (d, *J* = 8.9 Hz, 2H), 6.84 (d, *J* = 8.4 Hz, 2H), 6.68 (d, *J* = 8.9 Hz, 2H), 5.61 (s, 1H), 3.76 (s, 3H), 1.89 (s, 3H), 1.84 (s, 3H); **^{13}C NMR** (150 MHz, $CDCl_3$): δ 160.4, 158.6, 139.2, 133.1, 131.7, 130.0, 124.2, 123.6, 114.1, 85.9, 83.6, 55.6, 20.5, 19.8; **HRMS** (ESI, m/z) calcd. for $C_{18}H_{19}{}^{79}Br_1N_1O_4$ $[M+H]^+$ calc.: 392.0498; found: 392.0494.

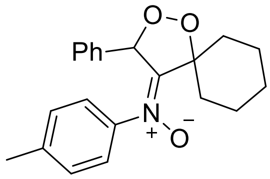

4n

Synthesis of **4n**: Following the typical procedure of **4a, 4n** was isolated by a silica column (670.0 mg, 2.00 mmol, 66%).

^{1}H NMR (600 MHz, $CDCl_3$): δ 7.18 (t, *J* = 8.6 Hz, 1H), 7.11 (t, *J* = 7.8 Hz, 2H), 6.95-6.90 (m, 4H), 6.87 (t, *J* = 7.8 Hz, 2H), 5.58 (s, 3H), 2.95-2.65 (m, 2H), 2.24 (s, 3H), 2.22-2.15 (m, 2H), 1.95-1.90 (m, 1H), 1.78-1.72 (m, 1H), 1.70-1.40 (m, 6H); **^{13}C NMR** (150 MHz, $CDCl_3$): δ 159.0, 143.8, 139.8, 129.1, 128.8, 128.6, 122.7, 87.6, 84.2, 32.1, 27.3, 24.5, 21.6, 21.2 , 20.4; **HRMS** (ESI, m/z) calcd. for $C_{21}H_{24}N_1O_3$ $[M+H]^+$ calc.: 338.1756; found: 338.1759.

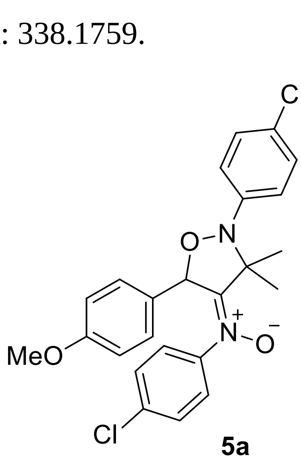

5a

Synthesis of **5a**: Following the typical procedure of **4a, 5a** was isolated by a silica column (1.17 g, 1.60 mmol, 53%).

^{1}H NMR (600 MHz, $CDCl_3$): δ 7.33-1.17 (m, 5H), 7.11 (dd, J_1 = 6.7 Hz, J_2 = 2.0 Hz, 2H), 6.95-6.88 (m, 4H), 6.67 (d, J = 8.8 Hz, 2H), 5.43 (s, 1H), 3.75 (s, 3H), 1.85 (s, 3H), 1.60 (s, 3H) (*E/Z* =3.4:1, *E-form*); selected peak of *Z-form*: δ 5.11(s, 1H),3.80(s, 3H), 1.38(s, 3H), 1.11(s, 3H); **^{13}C NMR** (150 MHz, $CDCl_3$): δ 160.2, 143.3, 135.4, 130.2, 129.1, 128.8, 128.6, 124.6, 122.1, 120.7, 115.5, 114.2, 113.9, 80.2, 69.4, 55.3, 19.6, 17.2 (*E-form*); selected peak of *Z-form*: δ 159.3, 144.4, 80.8, 21.7, 19.0; **HRMS** (ESI, m/z) calcd. for $C_{24}H_{23}Cl_2N_2O_3$ $[M+H]^+$ calc.: 457.1086; found: 457.1082.

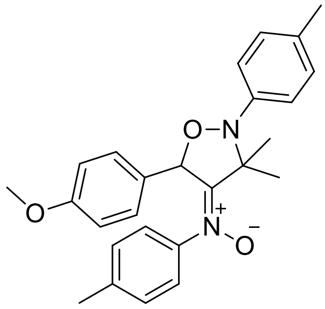

5b

A 100-mL flask was charged with 4-methylphenylnitroso **2b** (1.09 mg, 9.0 mmol), then evacuated and backfilled with N_2 balloon. To this solid was added anhydrous THF (15 mL), and the resulting mixture was cooled to 0 ^{o}C. To this mixture was added a THF solution (15 mL) of **1p** (525.0 mg, 3.0 mmol, 1 equiv), and the mixture was stirred for 2 h. The solution was concentrated under reduced pressure, and the resulting mixture was purified by a silica column with hexane/diethyl ether (1:3) to get afford compound **5b** (750.0 mg, 1.80 mmol, 60%) as white solid.

^{1}H NMR (600 MHz, $CDCl_3$): δ 7.18-7.05 (m, 4H), 6.98-6.82 (m, 6H), 6.62 (d, J =8.7 Hz, 2H), 5.45 (s, 1H), 3.72 (s, 3H), 2.31 (s, 3H), 2.24 (s, 3H), 1.84(s, 3H), 1.61 (s, 3H); **^{13}C NMR** (150 MHz, $CDCl_3$): δ 159.5, 159.1, 143.9, 142.1, 139.5, 134.8, 130.1, 129.4, 128.9, 127.7, 123.0, 121.2, 113.6, 80.1, 69.0, 55.3, 21.1, 20.8, 19.4, 17.4; **HRMS** (ESI, m/z) calcd. for $C_{26}H_{29}N_2O_3$ $[M+H]^+$ calc.: 417.2178; found: 417.2178.

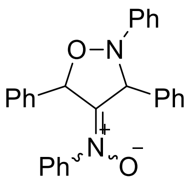

5c (*E/Z* =1:1)

Synthesis of **5c**: Following the typical procedure of **5b, 5c** was isolated by a silica column (1.02 g, 2.50 mmol, 83%).

^{1}H NMR (600 MHz, $CDCl_3$): δ 7.81-7.75 (m, 2H), 7.45-6.80 (m, 18H), 5.94 (s, 1H), 5.85 (s, 1H) (*E-form*); selected peak of *Z-form*: δ 6.42(s, 1H); 5.37(s, 1H); **^{13}C NMR** (150 MHz, $CDCl_3$): δ 155.9, 148.1, 145.5, 135.7, 135.0, 129.7, 129.1, 129.0, 128.8, 128.6, 128.4, 128.2, 128.0,127.9, 123.2, 123.1, 116.9, 80.9, 72.1 (*E-form*); selected peak of *Z-form*: 156.4, 147.1, 145.0, 135.4, 134.6, 117.3, 80.6, 71.5; **HRMS** (ESI, m/z) calcd. for $C_{27}H_{23}N_2O_2$ $[M+H]^+$ calc.: 407.1760; found: 407.1758.

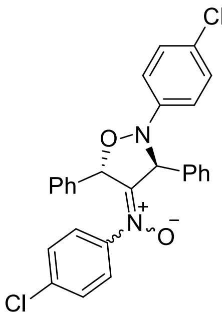

5d (*E/Z* = 1:1)

Synthesis of **5d**: Following the typical procedure of **5b, 5d** was isolated by a silica column (0.81 g, 1.72 mmol, 66%).

^{1}H NMR (600 MHz, $CDCl_3$): δ 7.75-6.78 (m, 18H), 5.88 (s, 1H), 5.66 (s, 1H) (*E-form*); selected peak of *Z-form*: 6.35 (s, 1H), 5.24 (s, 1H); **^{13}C NMR** (150 MHz, $CDCl_3$): δ 156.4, 145.1, 142.8, 135.6, 134.8, 134.0, 133.5, 129.3, 129.1, 128.9, 128.8, 128.7, 128.5, 128.4, 128.2, 124.3, 118.5, 80.3, 72.1; selected peak of *Z-form*: 156.9, 146.2, 143.3, 135.7, 134.4, 117.9, 80.6, 71.2 **HRMS** (ESI, m/z) calcd. for $C_{27}H_{21}Cl_2N_2O_2$ $[M+H]^+$ calc.: 475.0980; found: 475.0979.

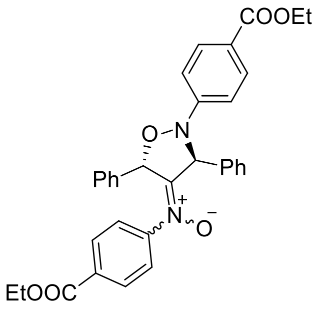

5e (*E*/*Z* = 1:1)

Synthesis of **5e**: Following the typical procedure of **4a, 5e** was isolated by a silica column (1.07 g, 1.95 mmol, 75%).

^{1}H NMR (600 MHz, $CDCl_3$): δ 7.90-6.80 (m, 18H), 6.01 (s, 1H), 5.92 (s, 1H), 4.35-4.22 (m, 4H), 1.38-1.27 (m, 6H) (*E-form*); selected peak of *Z-form*: 6.49 (s, 1H), 5.54 (s, 1H); **^{13}C NMR** (150 MHz, $CDCl_3$): δ 166.1, 164.9, 156.2, 149.6, 147.4, 134.2, 133.9, 131.2, 129.4, 129.0, 128.7, 128.4, 128.1, 127.2, 126.1, 123.8, 123.3, 123.0, 114.6, 79.7, 70.0, 61.6, 60.6, 14.2, 14.1 (*E-form*); selected peak of *Z-form*: δ 166.2, 165.0, 156.7, 150.8, 147.9, 135.3, 115.0, 80.3, 68.6; **HRMS** (ESI, m/z) calcd. for $C_{33}H_{31}N_2O_6$ $[M+H]^+$ calc.: 551.2177; found: 551.2177.

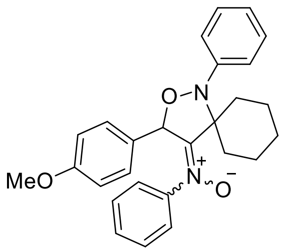

5f (*E*/*Z* = 5.3:1)

Synthesis of **5f**: Following the typical procedure of **5b, 5f** was isolated by a silica column (0.67 g, 1.57 mmol, 58%).

^{1}H NMR (600 MHz, $CDCl_3$): δ 7.65 (d, *J* =8.7 Hz, 2H) 7.51-7.41 (m, 5H), 7.20 (t, *J* =7.47 Hz, 2H), 6.96 (d, *J* =8.2 Hz, 2H), 6.90 (t, *J* =7.4 Hz, 1H), 6.84 (d, *J* =8.76 Hz, 2H), 5.73 (s, 1H), 3.75 (s, 3H), 2.34 (d, *J* =12.6 Hz, 1H), 1.78-1.42 (m, 7H), 1.29-1.27 (m, 1H), 0.92-0.87 (m, 1H) (*E-form*); selected peak of *Z-form*: 6.2 (s, 1H), 3.80 (s, 3H); **^{13}C NMR** (150 MHz, $CDCl_3$): δ 160.2, 158.4, 147.1, 143.2, 130.4, 129.4, 128.5, 126.5, 123.8, 121.7, 115.3,113.8, 113.2, 84.9, 69.3, 55.1, 32.8, 31.8, 24.2, 21.6, 21.4; selected peak of *Z-form*: 160.6, 159.4, 142.7, 125.8, 84.0, 82.0, 55.3, 31.3, 31.2, 24.0,

21.3; **HRMS** (ESI, m/z) calcd. for $C_{27}H_{29}N_2O_3$ $[M+H]^+$ calc.: 429.2173; found: 429.2172.

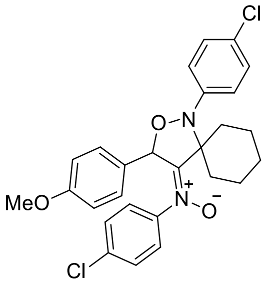

5g

Synthesis of **5g**: Following the typical procedure of **5b, 5g** was isolated by a silica column (0.94 g, 1.9 mmol, 70%).

^{1}H NMR (400 MHz, $CDCl_3$): δ 7.58 (d, *J* =8.4 Hz,2H), 7.39 (d, *J* =8.4 Hz, 2H), 7.21 (d, *J* =8.4 Hz, 2H), 7.12 (d, *J* =8.4 Hz, 2H), 6.88 (d, *J* =8.8 Hz, 2H), 6.83 (d, *J* =8.8 Hz, 2H), 5.69 (s, 1H), 3.76 (s, 3H), 2.28 (d, *J* = 13.6 Hz, 1H), 1.84-1.47 (m, 7H), 1.30-1.26 (m, 1H), 1.03-0.93 (m, 1H); **^{13}C NMR** (100 MHz, $CDCl_3$): δ 160.1, 159.3, 146.6, 142.6, 136.0, 130.3, 129.5, 128.9, 127.1, 126.8, 125.7, 117.3, 113.9, 82.4, 70.2, 55.2, 33.6, 32.7, 24.5, 22.1, 21.8; **HRMS** (ESI, m/z) calcd. for $C_{27}H_{27}Cl_2N_2O_3$ $[M+H]^+$ calc.: 497.1399; found: 497.1399.

Typical procedure for the preparation of 6a.

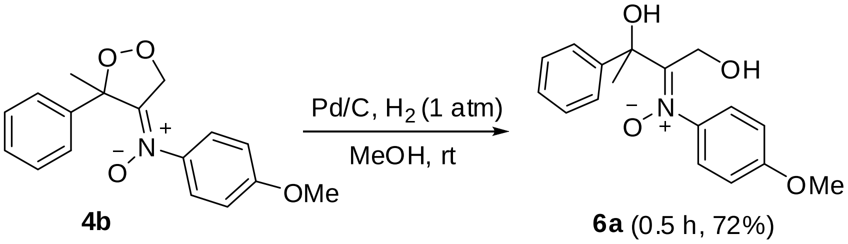

To a suspension of 10 % Pd/C (106.0 mg) in methanol (3.0 mL) under H_2 balloon (1 atm) was added compound **4b** (299.0 mg, 1.0 mmol), and the mixture was stirred at room temperature for 0.5 h. The solution was filtered through a celite pad and the filtrate was concentrated under reduced pressure. The crude residues were purified by a silica column with hexane/diethyl ether (1:3) to yield compound **6a** (217.0 mg, 0.72 mmol, 72%) as colorless oil.

^{1}H NMR (400 MHz, $CDCl_3$): δ 7.53 (d, *J* = 8.4 Hz, 2H), 7.30-7.15 (m, 5H), 6.79 (d, *J* = 8.8 Hz, 2H), 4.11 (d, *J* = 12.4 Hz, 1H), 3.94 (d, *J* = 12.4 Hz, 1H), 3.73 (s, 3H), 3.34(s, 1H), 1.67(s, 3H); **^{13}C NMR** (100 MHz, $CDCl_3$): δ 160.5, 154.6, 145.1, 138.3, 128.2, 127.3, 124.8, 124.7, 114.1, 76.1, 58.9, 55.5, 26.8; **HRMS** (ESI, m/z) calcd. for $C_{17}H_{20}N_1O_4$ $[M+H]^+$ calc.: 302.1392; found: 302.1391.

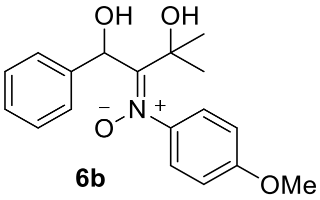

6b

Synthesis of **6b**: Following the typical procedure of **6a, 6c** was isolated by a silica column (205.0 mg, 0.65 mmol, 65%).

^{1}H NMR (400 MHz, $CDCl_3$): δ 7.40-7.20 (m, 7H), 6.84 (d, *J* = 6.8 Hz, 2H), 5.61 (s, 1H), 3.76 (s, 3H), 1.60(s, 3H), 1.45(s, 3H); **^{13}C NMR** (100 MHz, $CDCl_3$): δ 160.1, 156.7, 140.0, 139.1, 128.6, 127.7, 124.9, 124.5, 114.6, 74.6. 72.5, 55.6, 26.7, 26.3; **HRMS** (ESI, m/z) calcd. for $C_{18}H_{22}N_1O_4$ $[M+H]^+$ calc.: 316.1549; found: 316.1550.

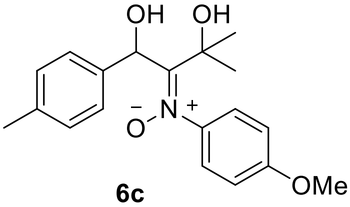

6c

Synthesis of **6c**: Following the typical procedure of **6a, 6b** was isolated by a silica column (224.0 mg, 0.68 mmol, 68%).

^{1}H NMR (600 MHz, $CDCl_3$): δ 7.31 (d, *J* = 8.4 Hz, 2H), 7.20-7.10 (m, 4H), 6.82 (d, *J* = 7.8 Hz, 2H), 5.56 (s, 1H), 3.75 (s, 3H), 2.31 (s, 3H), 1.60(s, 3H), 1.45(s, 3H); **^{13}C NMR** (100 MHz, $CDCl_3$): δ 159.9, 158.0, 138.7, 137.1, 136.9, 129.0, 124.9, 124.4, 114.4, 74.3, 71.9, 55.4, 26.5, 26.3, 21.0; **HRMS** (ESI, m/z) calcd. for $C_{19}H_{24}N_1O_4$ $[M+H]^+$ calc.: 330.1705; found: 330.1702.

Radical trapping experiment

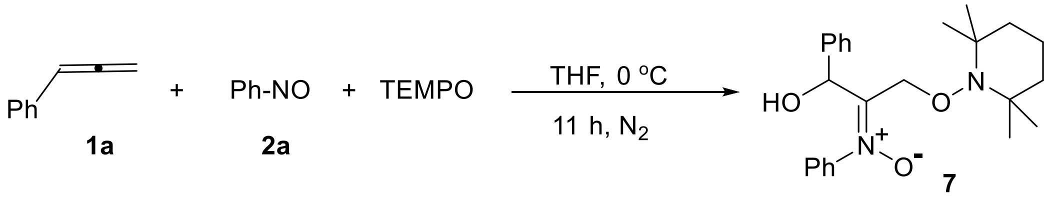

A reaction tube was charged with **2a** (128.6 mg, 1.2 mmol) and TEMPO (344.0 mg, 2.2 mmol), then evacuated and backfilled with N_2 balloon. To this mixture was added **1a** (116.0 mg, 1.0 mmol) in THF (10 mL) at 0 °C, and the mixture was stirred for 11 h. The solution was concentrated under reduced pressure, and the resulting mixture was purified by a silica column with hexane/ethyl acetate (1:1) to get afford compound **7** (52.0 mg, 0.13 mmol, 13%) as light yellow oil.

^{1}H NMR (600 MHz, $CDCl_3$): δ 7.64 (d, *J* = 7.2 Hz, 2H), 7.50-7.15 (m, 8H), 5.80 (d, *J* = 9.3 Hz, 1H), 4.40 (d, *J* = 12.4 Hz, 1H), 4.30 (d, *J* = 12.4 Hz, 1H), 1.50-1.20 (m, 6H), 1.05-0.80 (m, 12H); **^{13}C NMR** (150 MHz, $CDCl_3$): δ 150.1, 144.9, 140.1, 129.9, 129.3, 128.6, 128.1, 126.6, 123.5, 73.4, 72.9, 60.3, 39.7, 32.6, 20.1, 16.7; **HRMS** (ESI, m/z) calcd. for $C_{24}H_{33}N_2O_3$ $[M+H]^+$ calc.: 397.2491; found: 397.2492.

Electron Paramagnetic Resonance Data

EPR spectra were recorded at 80-273 K on a Bruker ESP-300E (X band, 9.8 GHz) with parameters setting as shown below: receiver gain=30 n; receiver phase=0 deg; receiver harmonic = 1; field modulation frequency = 100000 Hz; microwave frequency [Hz] = 9.439577e+09; field modulation amplitude [T] = 0.0001; receiver time constant[S] = 0.32768; microwave power = 0.015 W; receiver offset [%FS] = 0. The reaction of phenylallene **1a** with nitrosobenzene **2a** in THF under N_2 from 80 K to 273k in an EPR chamber while recording the EPR spectra (Fig S1).

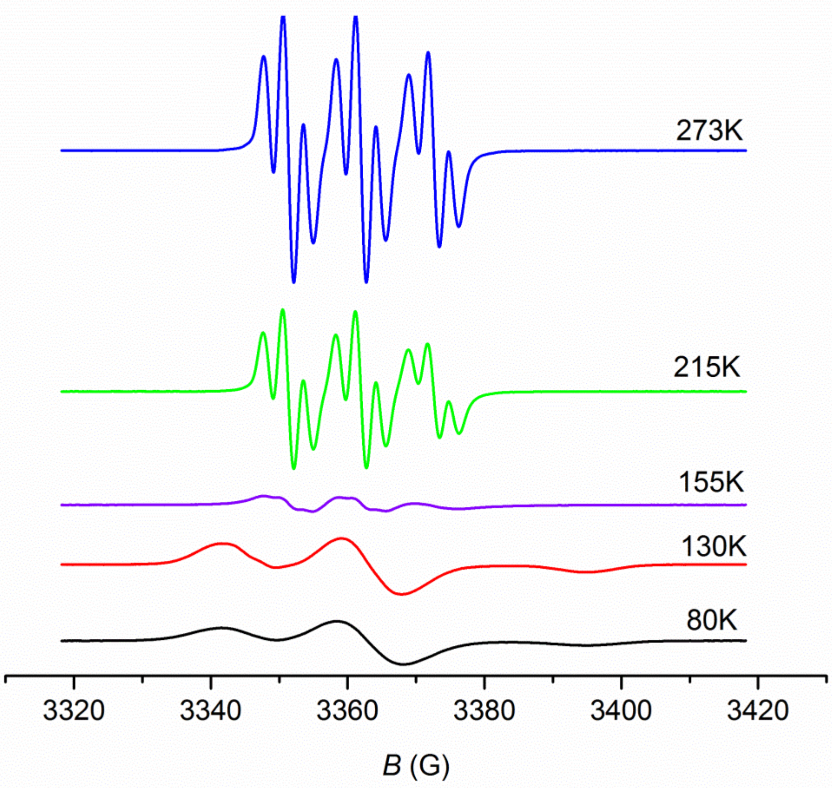

Fig S1 EPR signals of **1n** and **2a** under N_2 from 80K-273K.

Reference

1. Searles, S., Li, Y., Nassim, B., Lopes, M-T. R., Tran, P. T. & Crabbé, P. Observation on the synthesis of allenes by homologation of alk-1-ynes. *J. Chem. Soc., Perkin Trans. 1* **1984**, 747-751.
2. Ye, J., Li, S., Chen, B., Fan, W., Kuang, J., Liu, J., Liu, Y., Miao, B., Wan, B., Wang, Y., Xie, X., Yu, Q., Yuan, W. & Ma, S. Catalytic asymmetric synthesis of optically active allenes from terminal alkynes. *Org. Lett.* **14**, 1346–1349 (2012).
3. Clavier, H., Jeune, K., Riggi, I., Tenaglia, A. & Buono, G. Highly selective cobalt-mediated [6 + 2] cycloaddition of cycloheptatriene and allenes. *Org. Lett.* **13**, 308-311 (2011).
4. Kuang, J., Tang, X. & Ma, S. Zinc diiodide-promoted synthesis of trisubstituted allenes from propargylic amines. *Org. Chem. Front.* **2**, 470–475 (2015).
5. Kubitschke, J., Näther, C., Herges, R. Synthesis of functionalized triazatriangulenes for application in photo-switchable self-assembled monolayers. *Eur. J. Org. Chem.* **2010**, 5041–5055.

Crystallographic data

Crystallographic data for compound 3b

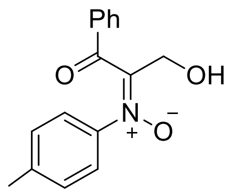

3b

CCDC 1507478

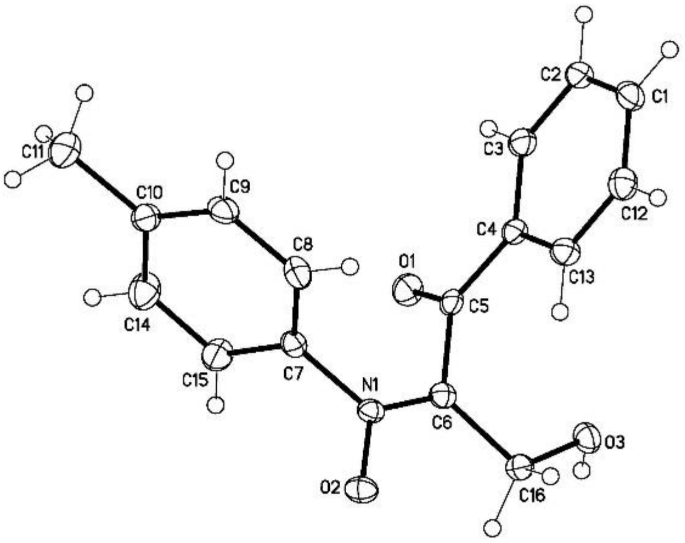

Table 1. Crystal data and structure refinement for 160334LT_0M.

Identification code	160334LT_0m	
Empirical formula	C16 H15 N O3	
Formula weight	269.29	
Temperature	100(2) K	
Wavelength	0.71073 Å	
Crystal system	Trigonal	
Space group	P 32	
Unit cell dimensions	a = 13.9065(11) Å	α= 90°.
	b = 13.9065(11) Å	β= 90°.
	c = 6.0548(5) Å	γ = 120°.
Volume	1014.07(18) Å^3	
Z	3	
Density (calculated)	1.323 Mg/m^3	

Absorption coefficient	0.092 mm^{-1}
F(000)	426
Crystal size	0.20 x 0.02 x 0.02 mm^3
Theta range for data collection	1.691 to 26.327°.
Index ranges	-17<=h<=17, -17<=k<=17, -7<=l<=7
Reflections collected	7841
Independent reflections	2610 [R(int) = 0.0315]
Completeness to theta = 25.242°	99.9 %
Absorption correction	Semi-empirical from equivalents
Max. and min. transmission	0.9485 and 0.8931
Refinement method	Full-matrix least-squares on F^2
Data / restraints / parameters	2610 / 1 / 184
Goodness-of-fit on F^2	1.205
Final R indices [I>2sigma(I)]	R1 = 0.0295, wR2 = 0.0778
R indices (all data)	R1 = 0.0351, wR2 = 0.1020
Absolute structure parameter	0.1(15)
Extinction coefficient	n/a
Largest diff. peak and hole	0.222 and -0.234 e.$Å^{-3}$

Table 2. Atomic coordinates (x 10^4) and equivalent isotropic displacement parameters ($Å^2$x 10^3) for 160334LT_0M. U(eq) is defined as one third of the trace of the orthogonalized U^{ij} tensor.

	x	y	z	U(eq)
O(1)	5477(2)	5166(2)	443(3)	21(1)
O(2)	4920(2)	7420(2)	4454(3)	18(1)
O(3)	2807(2)	4346(2)	1672(3)	19(1)
N(1)	5261(2)	6742(2)	3761(3)	14(1)
C(1)	4566(2)	2463(2)	6716(5)	21(1)
C(2)	5138(2)	2607(2)	4742(5)	20(1)
C(3)	5271(2)	3428(2)	3272(5)	18(1)
C(4)	4844(2)	4125(2)	3787(4)	15(1)
C(5)	5017(2)	5018(2)	2221(5)	14(1)
C(6)	4585(2)	5778(2)	2931(4)	14(1)
C(7)	6453(2)	7162(2)	3875(5)	15(1)
C(8)	6900(2)	6924(2)	5692(5)	20(1)
C(9)	8035(2)	7331(2)	5733(5)	21(1)
C(10)	8721(2)	7963(2)	4006(5)	20(1)
C(11)	9958(3)	8418(3)	4068(6)	28(1)

C(12)	4138(2)	3147(2)	7232(5)	20(1)
C(13)	4278(2)	3981(2)	5781(5)	18(1)
C(14)	8236(3)	8190(3)	2212(5)	26(1)
C(15)	7108(2)	7801(2)	2132(5)	21(1)
C(16)	3365(2)	5383(2)	2759(5)	16(1)

Table 3. Bond lengths [Å] and angles [°] for 160334LT_0M.

O(1)-C(5)	1.216(3)
O(2)-N(1)	1.318(3)
O(3)-C(16)	1.414(3)
O(3)-H(11)	0.8400
N(1)-C(6)	1.293(3)
N(1)-C(7)	1.458(3)
C(1)-C(12)	1.387(4)
C(1)-C(2)	1.393(5)
C(1)-H(1)	0.9500
C(2)-C(3)	1.385(4)
C(2)-H(3)	0.9500
C(3)-C(4)	1.402(4)
C(3)-H(4)	0.9500
C(4)-C(13)	1.400(4)
C(4)-C(5)	1.483(4)
C(5)-C(6)	1.517(4)
C(6)-C(16)	1.503(4)
C(7)-C(8)	1.382(4)
C(7)-C(15)	1.386(4)
C(8)-C(9)	1.385(4)
C(8)-H(10)	0.9500
C(9)-C(10)	1.392(4)
C(9)-H(9)	0.9500
C(10)-C(14)	1.395(4)
C(10)-C(11)	1.508(4)
C(11)-H(2)	0.9800
C(11)-H(5)	0.9800
C(11)-H(6)	0.9800
C(12)-C(13)	1.389(4)
C(12)-H(15)	0.9500

C(13)-H(14)	0.9500
C(14)-C(15)	1.381(4)
C(14)-H(7)	0.9500
C(15)-H(8)	0.9500
C(16)-H(13)	0.9900
C(16)-H(12)	0.9900
C(16)-O(3)-H(11)	109.5
C(6)-N(1)-O(2)	121.9(2)
C(6)-N(1)-C(7)	121.8(2)
O(2)-N(1)-C(7)	116.2(2)
C(12)-C(1)-C(2)	120.3(3)
C(12)-C(1)-H(1)	119.9
C(2)-C(1)-H(1)	119.9
C(3)-C(2)-C(1)	120.1(3)
C(3)-C(2)-H(3)	120.0
C(1)-C(2)-H(3)	120.0
C(2)-C(3)-C(4)	119.9(3)
C(2)-C(3)-H(4)	120.0
C(4)-C(3)-H(4)	120.0
C(13)-C(4)-C(3)	119.7(3)
C(13)-C(4)-C(5)	121.1(2)
C(3)-C(4)-C(5)	119.3(2)
O(1)-C(5)-C(4)	124.0(2)
O(1)-C(5)-C(6)	119.5(2)
C(4)-C(5)-C(6)	116.5(2)
N(1)-C(6)-C(16)	120.6(2)
N(1)-C(6)-C(5)	119.7(2)
C(16)-C(6)-C(5)	119.6(2)
C(8)-C(7)-C(15)	121.9(3)
C(8)-C(7)-N(1)	119.9(2)
C(15)-C(7)-N(1)	118.2(2)
C(7)-C(8)-C(9)	118.2(3)
C(7)-C(8)-H(10)	120.9
C(9)-C(8)-H(10)	120.9
C(8)-C(9)-C(10)	121.7(3)
C(8)-C(9)-H(9)	119.2
C(10)-C(9)-H(9)	119.2
C(9)-C(10)-C(14)	118.2(3)

C(9)-C(10)-C(11)	121.6(3)
C(14)-C(10)-C(11)	120.2(3)
C(10)-C(11)-H(2)	109.5
C(10)-C(11)-H(5)	109.5
H(2)-C(11)-H(5)	109.5
C(10)-C(11)-H(6)	109.5
H(2)-C(11)-H(6)	109.5
H(5)-C(11)-H(6)	109.5
C(1)-C(12)-C(13)	120.1(3)
C(1)-C(12)-H(15)	120.0
C(13)-C(12)-H(15)	120.0
C(12)-C(13)-C(4)	119.9(3)
C(12)-C(13)-H(14)	120.0
C(4)-C(13)-H(14)	120.0
C(15)-C(14)-C(10)	121.4(3)
C(15)-C(14)-H(7)	119.3
C(10)-C(14)-H(7)	119.3
C(14)-C(15)-C(7)	118.6(3)
C(14)-C(15)-H(8)	120.7
C(7)-C(15)-H(8)	120.7
O(3)-C(16)-C(6)	110.4(2)
O(3)-C(16)-H(13)	109.6
C(6)-C(16)-H(13)	109.6
O(3)-C(16)-H(12)	109.6
C(6)-C(16)-H(12)	109.6
H(13)-C(16)-H(12)	108.1

Symmetry transformations used to generate equivalent atoms:

Table 4. Anisotropic displacement parameters ($Å^2$x 10^3) for 160334LT_0M. The anisotropic displacement factor exponent takes the form: $-2\pi^2$[h^2 $a^{*2}U^{11}$ + ... + 2 h k a* b* U^{12}]

	U^{11}	U^{22}	U^{33}	U^{23}	U^{13}	U^{12}
O(1)	21(1)	21(1)	20(1)	1(1)	5(1)	11(1)
O(2)	22(1)	18(1)	19(1)	-4(1)	-1(1)	14(1)
O(3)	19(1)	14(1)	21(1)	0(1)	-4(1)	6(1)
N(1)	16(1)	15(1)	13(1)	2(1)	1(1)	9(1)
C(1)	19(1)	15(1)	26(2)	3(1)	-6(1)	7(1)

C(2)	16(1)	14(1)	31(2)	-1(1)	-4(1)	8(1)
C(3)	15(1)	16(1)	23(2)	-3(1)	-1(1)	7(1)
C(4)	12(1)	14(1)	19(2)	-2(1)	-3(1)	6(1)
C(5)	10(1)	13(1)	18(1)	-2(1)	-2(1)	4(1)
C(6)	16(1)	15(1)	12(1)	2(1)	1(1)	8(1)
C(7)	16(1)	12(1)	18(1)	-1(1)	-1(1)	7(1)
C(8)	21(1)	15(1)	19(2)	2(1)	0(1)	6(1)
C(9)	21(2)	16(1)	25(2)	1(1)	-9(1)	9(1)
C(10)	17(1)	16(1)	26(2)	-3(1)	-3(1)	8(1)
C(11)	19(2)	25(2)	38(2)	-3(1)	-5(1)	8(1)
C(12)	19(1)	20(2)	18(1)	1(1)	0(1)	8(1)
C(13)	19(1)	17(1)	20(1)	-1(1)	0(1)	10(1)
C(14)	21(2)	29(2)	22(2)	4(1)	1(1)	9(1)
C(15)	19(1)	26(2)	18(2)	5(1)	0(1)	10(1)
C(16)	15(1)	17(1)	17(1)	1(1)	1(1)	9(1)

Table 5. Hydrogen coordinates (x 10^4) and isotropic displacement parameters (Å^{2}x 10 ^{3}) for 160334LT_0M.

	x	y	z	U(eq)
H(11)	2624	4432	396	28
H(1)	4468	1894	7712	25
H(3)	5436	2142	4404	24
H(4)	5651	3519	1917	22
H(10)	6441	6493	6881	23
H(9)	8353	7174	6972	25
H(2)	10333	9203	4490	42
H(5)	10216	8344	2604	42
H(6)	10130	8001	5150	42
H(15)	3748	3045	8579	24
H(14)	3990	4454	6142	22
H(7)	8692	8622	1020	31
H(8)	6788	7966	910	26
H(13)	3050	5313	4256	19
H(12)	3255	5934	1928	19

Crystallographic data for compound 4b

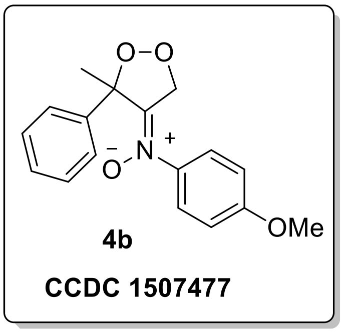

4b

CCDC 1507477

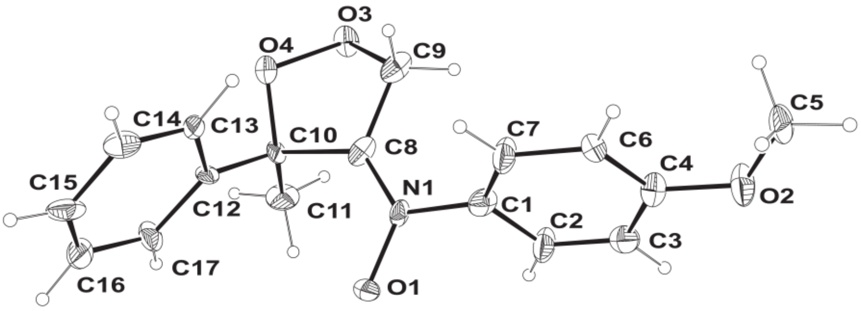

Table 1. Crystal data and structure refinement for a18125.

Identification code	a18125	
Empirical formula	C17 H17 N O4	
Formula weight	299.32	
Temperature	200(2) K	
Wavelength	0.71073 Å	
Crystal system	Monoclinic	
Space group	P 21	
Unit cell dimensions	a = 8.480(7) Å	α= 90°.
	b = 5.698(4) Å	β= 93.20(5)°.
	c = 15.656(13) Å	γ = 90°.
Volume	755.3(11) $Å^3$	
Z	2	
Density (calculated)	1.316 Mg/m^3	
Absorption coefficient	0.094 mm^{-1}	
F(000)	316	
Crystal size	1.00 x 0.19 x 0.06 mm^3	
Theta range for data collection	1.30 to 25.21°.	
Index ranges	-10<=h<=8, -6<=k<=6, -18<=l<=13	
Reflections collected	4585	
Independent reflections	2196 [R(int) = 0.3365]	

Completeness to theta = 25.21°	91.5 %
Absorption correction	multi-scan
Max. and min. transmission	0.9944 and 0.9117
Refinement method	Full-matrix least-squares on F^2
Data / restraints / parameters	2196 / 8 / 195
Goodness-of-fit on F^2	1.213
Final R indices [I>2sigma(I)]	R1 = 0.1803, wR2 = 0.4062
R indices (all data)	R1 = 0.2549, wR2 = 0.4474
Absolute structure parameter	-3(9)
Extinction coefficient	0.08(4)
Largest diff. peak and hole	0.628 and -0.425 e.$Å^{-3}$

Table 2. Atomic coordinates (x 10^4) and equivalent isotropic displacement parameters ($Å^2$x 10^3) for a18125. U(eq) is defined as one third of the trace of the orthogonalized U^{ij} tensor.

	x	y	z	U(eq)
C(1)	7240(20)	7850(30)	958(11)	51(5)
C(2)	8220(20)	9490(30)	554(10)	68(5)
C(3)	8500(20)	9120(30)	-264(12)	52(5)
C(4)	7879(19)	7150(30)	-722(10)	46(4)
C(5)	7810(30)	4820(40)	-2007(11)	73(6)
C(6)	6930(20)	5640(30)	-339(11)	52(5)
C(7)	6580(30)	5980(30)	508(11)	68(5)
C(8)	7474(19)	6970(30)	2414(9)	44(4)
C(9)	8460(20)	4840(30)	2287(10)	55(5)
C(10)	7320(30)	7280(30)	3413(11)	66(6)
C(11)	8230(30)	9270(30)	3711(13)	64(5)
C(12)	5530(17)	7160(20)	3601(9)	32(3)
C(13)	4610(20)	5230(30)	3347(10)	70(7)
C(14)	3010(20)	5040(30)	3544(11)	57(5)
C(15)	2500(30)	6830(30)	4070(12)	65(6)
C(16)	3220(30)	8590(30)	4289(11)	64(6)
C(17)	4900(20)	8920(30)	4120(11)	51(5)
N(1)	6869(16)	8310(20)	1799(7)	39(3)
O(1)	6110(15)	10298(18)	1972(7)	48(3)
O(2)	8349(16)	6940(30)	-1531(7)	72(4)
O(3)	9293(10)	4630(20)	3160(6)	44(3)
O(4)	7857(15)	5070(20)	3673(7)	57(3)

Table 3. Bond lengths [Å] and angles [°] for a18125.

C(1)-C(7)	1.38(3)
C(1)-N(1)	1.40(2)
C(1)-C(2)	1.42(2)
C(2)-C(3)	1.33(2)
C(2)-H(2)	0.9500
C(3)-C(4)	1.42(2)
C(3)-H(3)	0.9500
C(4)-C(6)	1.34(2)
C(4)-O(2)	1.354(18)
C(5)-O(2)	1.48(2)
C(5)-H(5A)	0.9800
C(5)-H(5B)	0.9800
C(5)-H(5C)	0.9800
C(6)-C(7)	1.39(2)
C(6)-H(6)	0.9500
C(7)-H(7)	0.9500
C(8)-N(1)	1.311(15)
C(8)-C(9)	1.49(2)
C(8)-C(10)	1.59(2)
C(9)-O(3)	1.507(19)
C(9)-H(9A)	0.9900
C(9)-H(9B)	0.9900
C(10)-O(4)	1.39(2)
C(10)-C(11)	1.43(2)
C(10)-C(12)	1.57(3)
C(11)-H(11A)	0.9800
C(11)-H(11B)	0.9800
C(11)-H(11C)	0.9800
C(12)-C(13)	1.39(2)
C(12)-C(17)	1.412(19)
C(13)-C(14)	1.42(3)
C(13)-H(13)	0.9500
C(14)-C(15)	1.40(2)
C(14)-H(14)	0.9500
C(15)-C(16)	1.21(3)
C(15)-H(15)	0.9500

C(16)-C(17)	1.48(3)
C(16)-H(16)	0.9500
C(17)-H(17)	0.9500
N(1)-O(1)	1.338(15)
O(3)-O(4)	1.516(14)
C(7)-C(1)-N(1)	121.4(15)
C(7)-C(1)-C(2)	120.8(14)
N(1)-C(1)-C(2)	117.6(14)
C(3)-C(2)-C(1)	117.8(16)
C(3)-C(2)-H(2)	121.1
C(1)-C(2)-H(2)	121.1
C(2)-C(3)-C(4)	121.9(16)
C(2)-C(3)-H(3)	119.1
C(4)-C(3)-H(3)	119.1
C(6)-C(4)-O(2)	125.2(16)
C(6)-C(4)-C(3)	119.8(14)
O(2)-C(4)-C(3)	115.0(15)
O(2)-C(5)-H(5A)	109.5
O(2)-C(5)-H(5B)	109.5
H(5A)-C(5)-H(5B)	109.5
O(2)-C(5)-H(5C)	109.5
H(5A)-C(5)-H(5C)	109.5
H(5B)-C(5)-H(5C)	109.5
C(4)-C(6)-C(7)	120.2(17)
C(4)-C(6)-H(6)	119.9
C(7)-C(6)-H(6)	119.9
C(1)-C(7)-C(6)	119.5(16)
C(1)-C(7)-H(7)	120.3
C(6)-C(7)-H(7)	120.2
N(1)-C(8)-C(9)	125.2(14)
N(1)-C(8)-C(10)	127.4(15)
C(9)-C(8)-C(10)	107.4(12)
C(8)-C(9)-O(3)	100.4(12)
C(8)-C(9)-H(9A)	111.7
O(3)-C(9)-H(9A)	111.7
C(8)-C(9)-H(9B)	111.7
O(3)-C(9)-H(9B)	111.7
H(9A)-C(9)-H(9B)	109.5

O(4)-C(10)-C(11)	117.4(19)
O(4)-C(10)-C(12)	102.1(12)
C(11)-C(10)-C(12)	118.9(13)
O(4)-C(10)-C(8)	98.2(11)
C(11)-C(10)-C(8)	109.8(14)
C(12)-C(10)-C(8)	108.2(16)
C(10)-C(11)-H(11A)	109.5
C(10)-C(11)-H(11B)	109.5
H(11A)-C(11)-H(11B)	109.5
C(10)-C(11)-H(11C)	109.5
H(11A)-C(11)-H(11C)	109.5
H(11B)-C(11)-H(11C)	109.5
C(13)-C(12)-C(17)	120.2(14)
C(13)-C(12)-C(10)	120.9(12)
C(17)-C(12)-C(10)	118.5(13)
C(14)-C(13)-C(12)	121.8(14)
C(14)-C(13)-H(13)	119.1
C(12)-C(13)-H(13)	119.1
C(13)-C(14)-C(15)	114.0(17)
C(13)-C(14)-H(14)	123.0
C(15)-C(14)-H(14)	123.0
C(16)-C(15)-C(14)	127(2)
C(16)-C(15)-H(15)	116.6
C(14)-C(15)-H(15)	116.6
C(15)-C(16)-C(17)	122.4(18)
C(15)-C(16)-H(16)	118.8
C(17)-C(16)-H(16)	118.8
C(12)-C(17)-C(16)	114.2(15)
C(12)-C(17)-H(17)	122.9
C(16)-C(17)-H(17)	122.9
C(8)-N(1)-O(1)	121.3(12)
C(8)-N(1)-C(1)	118.8(13)
O(1)-N(1)-C(1)	119.2(11)
C(4)-O(2)-C(5)	116.4(14)
C(9)-O(3)-O(4)	96.8(10)
C(10)-O(4)-O(3)	104.6(11)

Symmetry transformations used to generate equivalent atoms:

Table 4. Anisotropic displacement parameters ($Å^2$x 10^3)for a18125. The anisotropic displacement factor exponent takes the form: $-2\pi^2[h^2a^{*2}U^{11} + ... + 2 h k a^* b^* U^{12}]$

	U^{11}	U^{22}	U^{33}	U^{23}	U^{13}	U^{12}
C(1)	71(13)	52(11)	28(9)	2(7)	-15(10)	5(9)
C(2)	116(13)	56(7)	35(7)	-6(6)	27(8)	-22(8)
C(3)	45(10)	48(9)	62(12)	3(9)	6(9)	0(8)
C(4)	34(9)	67(10)	35(9)	-1(9)	-2(8)	-9(9)
C(5)	118(19)	75(13)	27(9)	-18(9)	13(11)	0(13)
C(6)	68(12)	48(10)	43(10)	-3(7)	18(10)	11(8)
C(7)	116(13)	56(7)	35(7)	-6(6)	27(8)	-22(8)
C(8)	42(10)	52(10)	36(9)	10(8)	-12(8)	-17(8)
C(9)	77(13)	56(11)	30(9)	6(8)	-24(9)	-19(10)
C(10)	130(18)	21(7)	53(10)	-13(8)	58(12)	3(10)
C(11)	91(15)	29(8)	69(12)	-16(9)	-3(11)	-15(9)
C(12)	36(8)	16(6)	44(8)	-7(6)	8(7)	-3(7)
C(13)	121(18)	28(8)	70(12)	6(8)	65(13)	8(10)
C(14)	67(12)	31(8)	73(13)	-5(9)	-5(10)	-12(9)
C(15)	99(17)	42(11)	51(11)	9(8)	-15(11)	28(11)
C(16)	111(18)	44(11)	39(10)	-11(8)	29(12)	-1(11)
C(17)	61(12)	49(10)	46(10)	-19(8)	19(9)	-3(9)
N(1)	35(8)	61(8)	20(7)	13(6)	0(6)	11(7)
O(1)	64(8)	36(5)	43(7)	0(5)	8(6)	15(6)
O(2)	88(11)	92(10)	38(7)	-15(7)	20(7)	-17(9)
O(3)	12(5)	73(8)	47(7)	-1(6)	-6(5)	-1(5)
O(4)	73(8)	58(7)	42(7)	3(6)	23(6)	7(7)

Table 5. Hydrogen coordinates (x 10^4) and isotropic displacement parameters ($Å^2$x 10^3) for a18125.

	x	y	z	U(eq)
H(2)	8661	10797	857	82
H(3)	9133	10216	-549	62
H(5A)	8220	4841	-2579	110
H(5B)	6649	4800	-2059	110

H(5C)	8188	3416	-1699	110
H(6)	6498	4334	-648	63
H(7)	5881	4940	775	82
H(9A)	9220	5089	1837	67
H(9B)	7807	3443	2145	67
H(11A)	9326	9091	3551	95
H(11B)	8208	9380	4335	95
H(11C)	7782	10703	3450	95
H(13)	5077	4011	3032	85
H(14)	2343	3801	3338	69
H(15)	1471	6663	4277	78
H(16)	2690	9788	4579	76
H(17)	5512	10204	4342	62

Crystallographic data for compound 5b

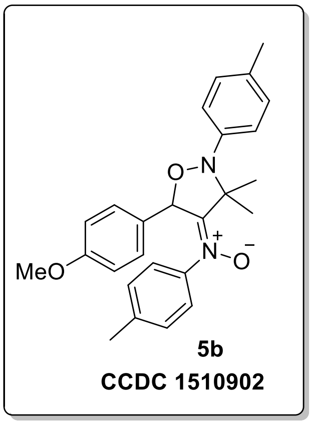

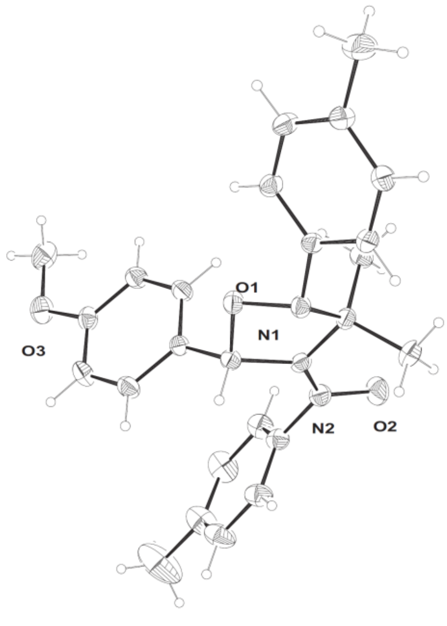

Table 1. Crystal data and structure refinement for a18191.

Identification code	a18191	
Empirical formula	C26 H28 N2 O3	
Formula weight	416.50	
Temperature	296(2) K	
Wavelength	0.71073 Å	
Crystal system	Triclinic	
Space group	P -1	
Unit cell dimensions	a = 10.294(2) Å	α= 69.312(6)°.
	b = 10.7033(19) Å	β= 79.586(8)°.
	c = 12.376(2) Å	γ = 66.460(6)°.
Volume	1168.2(4) $Å^3$	
Z	2	
Density (calculated)	1.184 Mg/m^3	
Absorption coefficient	0.078 mm^{-1}	
F(000)	444	
Crystal size	0.38 x 0.14 x 0.03 mm^3	
Theta range for data collection	2.18 to 25.01°.	
Index ranges	-12<=h<=12, -12<=k<=12, -14<=l<=14	
Reflections collected	10194	
Independent reflections	4036 [R(int) = 0.0362]	
Completeness to theta = 25.01°	97.7 %	
Absorption correction	multi-scan	
Max. and min. transmission	0.9977 and 0.9711	
Refinement method	Full-matrix least-squares on F^2	
Data / restraints / parameters	4036 / 0 / 280	
Goodness-of-fit on F^2	1.054	
Final R indices [I>2sigma(I)]	R1 = 0.0641, wR2 = 0.1185	
R indices (all data)	R1 = 0.1260, wR2 = 0.1408	
Largest diff. peak and hole	0.198 and -0.173 e.$Å^{-3}$	

Table 2. Atomic coordinates (x 10^4) and equivalent isotropic displacement parameters ($Å^2$x 10^3) for a18191. U(eq) is defined as one third of the trace of the orthogonalized U^{ij} tensor.

	x	y	z	U(eq)
C(1)	11657(3)	6495(3)	11398(2)	44(1)
C(2)	10710(3)	7375(3)	12007(2)	52(1)
C(3)	11140(3)	7435(3)	12983(3)	61(1)

C(4)	12499(4)	6646(3)	13372(3)	58(1)
C(5)	12965(4)	6745(4)	14421(3)	93(1)
C(6)	13422(3)	5761(3)	12760(3)	58(1)
C(7)	13010(3)	5666(3)	11797(2)	53(1)
C(8)	11834(3)	7152(3)	9237(2)	45(1)
C(9)	11764(3)	8625(3)	9182(3)	57(1)
C(10)	13332(3)	6211(3)	8961(3)	64(1)
C(11)	10752(3)	7297(3)	8492(2)	43(1)
C(12)	9853(3)	7694(3)	6691(2)	52(1)
C(13)	9003(4)	8963(4)	5980(3)	68(1)
C(14)	8057(4)	8948(4)	5335(3)	90(1)
C(15)	7963(5)	7673(5)	5385(3)	93(1)
C(16)	6887(6)	7670(6)	4690(4)	175(3)
C(17)	8854(4)	6420(4)	6083(3)	84(1)
C(18)	9805(4)	6410(4)	6743(3)	66(1)
C(19)	9499(3)	6992(3)	9210(2)	44(1)
C(20)	8041(3)	8091(3)	8895(2)	42(1)
C(21)	7015(3)	7727(3)	8637(3)	57(1)
C(22)	5671(4)	8727(4)	8368(3)	64(1)
C(23)	5331(3)	10111(4)	8366(2)	53(1)
C(24)	3548(4)	12439(4)	8136(3)	82(1)
C(25)	6336(3)	10495(3)	8612(2)	55(1)
C(26)	7682(3)	9485(3)	8865(2)	53(1)
N(1)	11315(2)	6334(2)	10389(2)	44(1)
N(2)	10865(3)	7705(3)	7364(2)	54(1)
O(1)	9761(2)	6987(2)	10322(2)	49(1)
O(2)	11881(2)	8120(3)	6760(2)	76(1)
O(3)	3953(2)	11017(3)	8116(2)	74(1)

Table 3. Bond lengths [Å] and angles [°] for a18191.

C(1)-C(2)	1.382(4)
C(1)-C(7)	1.387(4)
C(1)-N(1)	1.439(3)
C(2)-C(3)	1.389(4)
C(2)-H(2)	0.9300
C(3)-C(4)	1.383(4)
C(3)-H(3)	0.9300
C(4)-C(6)	1.379(4)

C(4)-C(5)	1.513(4)
C(5)-H(5A)	0.9600
C(5)-H(5B)	0.9600
C(5)-H(5C)	0.9600
C(6)-C(7)	1.383(4)
C(6)-H(6)	0.9300
C(7)-H(7)	0.9300
C(8)-C(11)	1.499(3)
C(8)-N(1)	1.517(3)
C(8)-C(10)	1.524(4)
C(8)-C(9)	1.526(4)
C(9)-H(9A)	0.9600
C(9)-H(9B)	0.9600
C(9)-H(9C)	0.9600
C(10)-H(10A)	0.9600
C(10)-H(10B)	0.9600
C(10)-H(10C)	0.9600
C(11)-N(2)	1.306(3)
C(11)-C(19)	1.508(4)
C(12)-C(13)	1.367(4)
C(12)-C(18)	1.373(4)
C(12)-N(2)	1.453(3)
C(13)-C(14)	1.374(4)
C(13)-H(13)	0.9300
C(14)-C(15)	1.386(5)
C(14)-H(14)	0.9300
C(15)-C(17)	1.368(5)
C(15)-C(16)	1.522(5)
C(16)-H(16A)	0.9600
C(16)-H(16B)	0.9600
C(16)-H(16C)	0.9600
C(17)-C(18)	1.379(4)
C(17)-H(17)	0.9300
C(18)-H(18)	0.9300
C(19)-O(1)	1.447(3)
C(19)-C(20)	1.505(4)
C(19)-H(19)	0.9800
C(20)-C(26)	1.375(4)

C(20)-C(21)	1.381(4)
C(21)-C(22)	1.379(4)
C(21)-H(21)	0.9300
C(22)-C(23)	1.379(4)
C(22)-H(22)	0.9300
C(23)-C(25)	1.369(4)
C(23)-O(3)	1.377(3)
C(24)-O(3)	1.417(4)
C(24)-H(24A)	0.9600
C(24)-H(24B)	0.9600
C(24)-H(24C)	0.9600
C(25)-C(26)	1.381(4)
C(25)-H(25)	0.9300
C(26)-H(26)	0.9300
N(1)-O(1)	1.472(3)
N(2)-O(2)	1.305(3)

C(2)-C(1)-C(7)	118.7(3)
C(2)-C(1)-N(1)	124.1(3)
C(7)-C(1)-N(1)	117.2(2)
C(1)-C(2)-C(3)	119.6(3)
C(1)-C(2)-H(2)	120.2
C(3)-C(2)-H(2)	120.2
C(4)-C(3)-C(2)	122.3(3)
C(4)-C(3)-H(3)	118.8
C(2)-C(3)-H(3)	118.8
C(6)-C(4)-C(3)	117.2(3)
C(6)-C(4)-C(5)	121.0(3)
C(3)-C(4)-C(5)	121.9(3)
C(4)-C(5)-H(5A)	109.5
C(4)-C(5)-H(5B)	109.5
H(5A)-C(5)-H(5B)	109.5
C(4)-C(5)-H(5C)	109.5
H(5A)-C(5)-H(5C)	109.5
H(5B)-C(5)-H(5C)	109.5
C(4)-C(6)-C(7)	121.5(3)
C(4)-C(6)-H(6)	119.3
C(7)-C(6)-H(6)	119.3

C(6)-C(7)-C(1)	120.7(3)
C(6)-C(7)-H(7)	119.6
C(1)-C(7)-H(7)	119.6
C(11)-C(8)-N(1)	98.4(2)
C(11)-C(8)-C(10)	113.0(2)
N(1)-C(8)-C(10)	108.3(2)
C(11)-C(8)-C(9)	110.7(2)
N(1)-C(8)-C(9)	114.1(2)
C(10)-C(8)-C(9)	111.6(2)
C(8)-C(9)-H(9A)	109.5
C(8)-C(9)-H(9B)	109.5
H(9A)-C(9)-H(9B)	109.5
C(8)-C(9)-H(9C)	109.5
H(9A)-C(9)-H(9C)	109.5
H(9B)-C(9)-H(9C)	109.5
C(8)-C(10)-H(10A)	109.5
C(8)-C(10)-H(10B)	109.5
H(10A)-C(10)-H(10B)	109.5
C(8)-C(10)-H(10C)	109.5
H(10A)-C(10)-H(10C)	109.5
H(10B)-C(10)-H(10C)	109.5
N(2)-C(11)-C(8)	123.5(2)
N(2)-C(11)-C(19)	125.1(2)
C(8)-C(11)-C(19)	111.3(2)
C(13)-C(12)-C(18)	121.2(3)
C(13)-C(12)-N(2)	119.2(3)
C(18)-C(12)-N(2)	119.6(3)
C(12)-C(13)-C(14)	119.1(3)
C(12)-C(13)-H(13)	120.5
C(14)-C(13)-H(13)	120.5
C(13)-C(14)-C(15)	121.2(4)
C(13)-C(14)-H(14)	119.4
C(15)-C(14)-H(14)	119.4
C(17)-C(15)-C(14)	118.2(3)
C(17)-C(15)-C(16)	121.0(4)
C(14)-C(15)-C(16)	120.8(4)
C(15)-C(16)-H(16A)	109.5
C(15)-C(16)-H(16B)	109.5

H(16A)-C(16)-H(16B)	109.5
C(15)-C(16)-H(16C)	109.5
H(16A)-C(16)-H(16C)	109.5
H(16B)-C(16)-H(16C)	109.5
C(15)-C(17)-C(18)	121.6(3)
C(15)-C(17)-H(17)	119.2
C(18)-C(17)-H(17)	119.2
C(12)-C(18)-C(17)	118.7(3)
C(12)-C(18)-H(18)	120.6
C(17)-C(18)-H(18)	120.6
O(1)-C(19)-C(20)	107.1(2)
O(1)-C(19)-C(11)	100.75(19)
C(20)-C(19)-C(11)	117.8(2)
O(1)-C(19)-H(19)	110.2
C(20)-C(19)-H(19)	110.2
C(11)-C(19)-H(19)	110.2
C(26)-C(20)-C(21)	117.8(3)
C(26)-C(20)-C(19)	121.2(2)
C(21)-C(20)-C(19)	121.0(3)
C(22)-C(21)-C(20)	121.1(3)
C(22)-C(21)-H(21)	119.4
C(20)-C(21)-H(21)	119.4
C(21)-C(22)-C(23)	119.9(3)
C(21)-C(22)-H(22)	120.1
C(23)-C(22)-H(22)	120.1
C(25)-C(23)-O(3)	124.6(3)
C(25)-C(23)-C(22)	119.9(3)
O(3)-C(23)-C(22)	115.5(3)
O(3)-C(24)-H(24A)	109.5
O(3)-C(24)-H(24B)	109.5
H(24A)-C(24)-H(24B)	109.5
O(3)-C(24)-H(24C)	109.5
H(24A)-C(24)-H(24C)	109.5
H(24B)-C(24)-H(24C)	109.5
C(23)-C(25)-C(26)	119.5(3)
C(23)-C(25)-H(25)	120.3
C(26)-C(25)-H(25)	120.3
C(20)-C(26)-C(25)	121.9(3)

C(20)-C(26)-H(26)	119.1
C(25)-C(26)-H(26)	119.1
C(1)-N(1)-O(1)	107.03(19)
C(1)-N(1)-C(8)	115.9(2)
O(1)-N(1)-C(8)	103.17(18)
O(2)-N(2)-C(11)	123.8(2)
O(2)-N(2)-C(12)	115.2(2)
C(11)-N(2)-C(12)	121.0(2)
C(19)-O(1)-N(1)	104.46(18)
C(23)-O(3)-C(24)	117.3(2)

Symmetry transformations used to generate equivalent atoms:

Table 4. Anisotropic displacement parameters (Å^{2}x 10^3)for a18191. The anisotropic displacement factor exponent takes the form: $-2\pi^2[h^2a^{*2}U^{11} + ... + 2 h k a^* b^* U^{12}]$

	U^{11}	U^{22}	U^{33}	U^{23}	U^{13}	U^{12}
C(1)	45(2)	38(2)	49(2)	-17(1)	2(2)	-16(2)
C(2)	49(2)	45(2)	59(2)	-20(2)	1(2)	-12(2)
C(3)	62(2)	59(2)	65(2)	-34(2)	9(2)	-19(2)
C(4)	62(2)	63(2)	56(2)	-26(2)	2(2)	-25(2)
C(5)	101(3)	117(3)	80(3)	-55(2)	-9(2)	-35(3)
C(6)	48(2)	64(2)	64(2)	-23(2)	-8(2)	-18(2)
C(7)	42(2)	55(2)	62(2)	-25(2)	-1(2)	-12(2)
C(8)	40(2)	42(2)	51(2)	-16(2)	5(1)	-14(1)
C(9)	59(2)	51(2)	69(2)	-20(2)	1(2)	-27(2)
C(10)	46(2)	63(2)	71(2)	-27(2)	8(2)	-8(2)
C(11)	46(2)	37(2)	46(2)	-17(1)	7(1)	-14(1)
C(12)	64(2)	53(2)	39(2)	-13(2)	3(2)	-26(2)
C(13)	85(3)	59(2)	58(2)	-6(2)	-5(2)	-34(2)
C(14)	110(3)	89(3)	59(2)	11(2)	-25(2)	-46(3)
C(15)	135(4)	113(4)	49(2)	1(2)	-22(2)	-76(3)
C(16)	249(7)	203(6)	118(4)	8(4)	-98(4)	-141(5)
C(17)	132(4)	90(3)	54(2)	-19(2)	-7(2)	-68(3)
C(18)	91(3)	55(2)	54(2)	-15(2)	-2(2)	-32(2)
C(19)	45(2)	41(2)	48(2)	-14(1)	0(1)	-18(2)
C(20)	44(2)	42(2)	42(2)	-12(1)	2(1)	-20(2)
C(21)	60(2)	51(2)	69(2)	-20(2)	-3(2)	-29(2)

C(22)	55(2)	70(2)	79(2)	-20(2)	-10(2)	-34(2)
C(23)	41(2)	64(2)	48(2)	-13(2)	-4(2)	-18(2)
C(24)	55(2)	77(3)	90(3)	-25(2)	-9(2)	0(2)
C(25)	51(2)	47(2)	68(2)	-21(2)	-7(2)	-15(2)
C(26)	48(2)	53(2)	66(2)	-23(2)	-7(2)	-22(2)
N(1)	37(2)	43(1)	51(1)	-15(1)	-2(1)	-12(1)
N(2)	58(2)	51(2)	53(2)	-18(1)	7(1)	-25(1)
O(1)	41(1)	59(1)	47(1)	-17(1)	0(1)	-18(1)
O(2)	78(2)	99(2)	58(1)	-19(1)	19(1)	-55(2)
O(3)	49(2)	77(2)	85(2)	-20(1)	-15(1)	-13(1)

Table 5. Hydrogen coordinates (x 10^4) and isotropic displacement parameters ($Å^2$x 10^3) for a18191.

	x	y	z	U(eq)
H(2)	9790	7925	11764	62
H(3)	10491	8025	13390	73
H(5A)	13937	6121	14553	140
H(5B)	12380	6462	15085	140
H(5C)	12875	7714	14290	140
H(6)	14343	5215	13000	69
H(7)	13647	5041	11414	63
H(9A)	12465	8510	9662	86
H(9B)	10838	9146	9451	86
H(9C)	11944	9146	8398	86
H(10A)	13978	6156	9462	96
H(10B)	13603	6617	8171	96
H(10C)	13358	5265	9077	96
H(13)	9065	9824	5933	81
H(14)	7469	9810	4856	107
H(16A)	6349	8638	4260	262
H(16B)	6259	7237	5205	262
H(16C)	7375	7132	4164	262
H(17)	8818	5554	6112	100
H(18)	10403	5550	7215	79
H(19)	9543	6043	9258	53
H(21)	7235	6793	8645	69

H(22)	4995	8467	8188	77
H(24A)	2571	12961	7949	123
H(24B)	4129	12899	7578	123
H(24C)	3670	12421	8893	123
H(25)	6115	11428	8609	66
H(26)	8365	9756	9020	63

Crystallographic data for compound 5c:

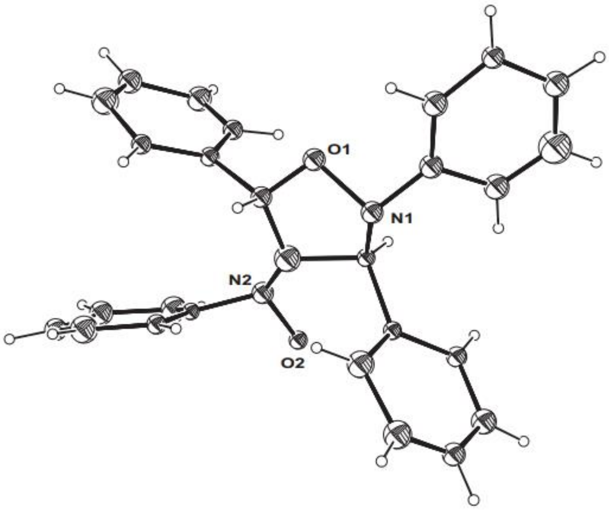

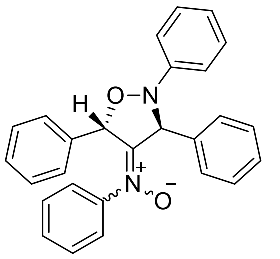

5c (CCDC 1540299)

Table 1. Crystal data and structure refinement for d18485f.

Identification code d18485f

Empirical formula C28 H24 Cl2 N2 O2

Formula weight 491.39

Temperature 200(2) K

Wavelength 0.71073 Å

Crystal system Monoclinic

Space group P 21

Unit cell dimensions a = 16.420(2) Å □= 90°.

b = 5.9044(8) Å □= 90.487(4)°.

c = 25.212(3) Å □ = 90°.

Volume 2444.3(6) Å3

Z 4

Density (calculated) 1.335 Mg/m3

Absorption coefficient 0.294 mm-1

F(000) 1024

Crystal size 0.29 x 0.05 x 0.01 mm3

Theta range for data collection 2.42 to 25.46°.

Index ranges -19<=h<=19, -7<=k<=7, -29<=l<=30

Reflections collected 38821

Independent reflections 8777 [R(int) = 0.0853]

Completeness to theta = 25.46° 97.8 %

Absorption correction None

Max. and min. transmission 0.9971 and 0.9196

Refinement methodFull-matrix least-squares on F2

Data / restraints / parameters 8777 / 25 / 296

Goodness-of-fit on F2 2.629

Final R indices [I>2sigma(I)] R1 = 0.2542, wR2 = 0.5938

R indices (all data) R1 = 0.2885, wR2 = 0.6117

Absolute structure parameter 0.4(4)

Largest diff. peak and hole 0.985 and -1.595 e.Å-3

Table 2. Atomic coordinates (x 104) and equivalent isotropic displacement parameters (Å2x 103) for d18485f. U(eq) is defined as one third of the trace of the orthogonalized Uij tensor.

	x	y	z	U(eq)
C(1)	7707(9)	1010(30)	-590(6)	49(4)
C(2)	8053(10)	-1050(40)	-795(7)	63(5)
C(3)	8132(13)	-1330(40)	-1324(7)	99
C(4)	7890(10)	260(30)	-1688(7)	57(4)
C(5)	7530(8)	2250(30)	-1508(5)	40(3)
C(6)	7486(9)	2450(30)	-958(6)	52(4)
C(7)	6791(9)	2680(30)	540(5)	40(3)
C(8)	5807(8)	2730(30)	698(5)	35(3)
C(9)	5576(8)	4910(30)	948(5)	38(3)
C(10)	4677(12)	4960(40)	1001(7)	70(5)
C(11)	4237(12)	3340(30)	851(7)	59(4)
C(12)	4502(12)	950(40)	610(7)	65(5)
C(13)	5303(9)	1190(30)	519(5)	38(3)
C(14)	7210(12)	760(40)	776(7)	63(5)
C(15)	6770(7)	710(20)	1691(4)	27(3)
C(16)	7145(8)	2890(30)	1799(5)	36(3)
C(17)	6681(11)	3740(40)	2262(7)	68(5)
C(18)	6172(9)	3060(30)	2548(6)	45(4)

C(19)	5837(11)	790(40)	2348(7)	58(4)
C(20)	6215(12)	-590(40)	1923(7)	67(5)
C(21)	7666(8)	-510(30)	330(5)	31(3)
C(22)	8565(8)	-980(20)	525(5)	32(3)
C(23)	8910(9)	-3390(30)	369(6)	42(3)
C(24)	9794(11)	-3480(40)	503(7)	60(5)
C(25)	10171(10)	-1850(30)	740(6)	48(4)
C(26)	9935(13)	280(40)	872(8)	69(5)
C(27)	8985(12)	650(40)	776(7)	63(5)
C(28)	7750(8)	1160(30)	4360(6)	40(3)
C(29)	7531(8)	-760(30)	4008(6)	44(3)
C(30)	7711(13)	-410(50)	3521(10)	97(7)
C(31)	8005(9)	1630(30)	3330(6)	45(4)
C(32)	8277(8)	3370(30)	3633(5)	38(3)
C(33)	8152(8)	3020(20)	4205(5)	31(3)
C(34)	7693(9)	2540(30)	5286(5)	40(3)
C(35)	8537(9)	3290(30)	5435(5)	41(3)
C(36)	8932(9)	5140(30)	5368(5)	41(3)
C(37)	9632(9)	5700(30)	5510(5)	40(3)
C(38)	10122(12)	4200(40)	5769(7)	63(5)
C(39)	9757(8)	2040(30)	5865(5)	35(3)
C(40)	9007(8)	1500(30)	5707(5)	36(3)
C(41)	7190(7)	1730(20)	5724(5)	27(3)
C(42)	6771(12)	1380(40)	6614(7)	66(5)
C(43)	6119(8)	2160(30)	6945(5)	41(3)
C(44)	5798(14)	900(50)	7421(9)	82(6)
C(45)	6095(10)	-620(40)	7492(7)	56(4)
C(46)	6774(9)	-1900(30)	7230(6)	47(4)
C(47)	7007(11)	-580(30)	6802(6)	54(4)
C(48)	6747(8)	-550(30)	5587(5)	31(3)
C(49)	5920(8)	-940(30)	5663(5)	36(3)
C(50)	5615(10)	-2550(30)	5902(5)	51(4)
C(51)	4783(8)	-2790(30)	6053(5)	37(3)
C(52)	4218(10)	-910(30)	5922(6)	50(4)
C(53)	4527(9)	590(30)	5642(5)	41(3)
C(54)	5400(10)	1140(30)	5523(6)	47(4)
C(55)	11315(14)	2510(40)	2476(8)	83
C(56)	9227(9)	-3470(30)	1939(6)	41(4)

N(1)	7689(7)	1250(20)	-24(5)	43(3)
N(2)	7014(8)	-450(30)	1188(5)	48(3)
N(3)	7636(7)	590(20)	4918(4)	34(3)
N(4)	7175(6)	2559(19)	6229(4)	26(2)
O(1)	6839(6)	2340(20)	41(4)	46(3)
O(2)	7395(5)	-2485(18)	1309(3)	34(2)
O(3)	6875(5)	-533(17)	4988(3)	34(2)
O(4)	7407(7)	4620(20)	6303(5)	61(3)
Cl(1)	10741(4)	3549(10)	3025(2)	80(2)
Cl(2)	11108(4)	-445(12)	2446(2)	85(2)
Cl(3)	8785(5)	-2138(16)	2504(3)	120(3)
Cl(4)	9321(8)	-6040(20)	1871(5)	180(6)

Table 3. Bond lengths [Å] and angles [°] for d18485f.

C(1)-C(6)	1.31(2)
C(1)-N(1)	1.436(19)
C(1)-C(2)	1.44(3)
C(2)-C(3)	1.352(17)
C(2)-H(2)	0.9500
C(3)-C(4)	1.373(17)
C(3)-H(3)	0.9500
C(4)-C(5)	1.39(2)
C(4)-H(4)	0.9500
C(5)-C(6)	1.40(2)
C(5)-H(5)	0.9500
C(6)-H(6)	0.9500
C(7)-O(1)	1.277(17)
C(7)-C(14)	1.45(3)
C(7)-C(8)	1.667(19)
C(7)-H(7)	1.0000
C(8)-C(13)	1.31(2)
C(8)-C(9)	1.48(2)
C(9)-C(10)	1.48(2)
C(9)-H(9)	0.9500
C(10)-C(11)	1.25(3)
C(10)-H(10)	0.9500
C(11)-C(12)	1.60(3)

C(11)-H(11)	0.9500
C(12)-C(13)	1.35(2)
C(12)-H(12)	0.9500
C(13)-H(13)	0.9500
C(14)-N(2)	1.30(2)
C(14)-C(21)	1.55(2)
C(15)-C(20)	1.33(2)
C(15)-C(16)	1.45(2)
C(15)-N(2)	1.498(17)
C(16)-C(17)	1.48(2)
C(16)-H(16)	0.9500
C(17)-C(18)	1.18(2)
C(17)-H(17)	0.9500
C(18)-C(19)	1.53(3)
C(18)-H(18)	0.9500
C(19)-C(20)	1.49(3)
C(19)-H(19)	0.9500
C(20)-H(20)	0.9500
C(21)-N(1)	1.369(19)
C(21)-C(22)	1.578(18)
C(21)-H(21)	1.0000
C(22)-C(27)	1.34(3)
C(22)-C(23)	1.58(2)
C(23)-C(24)	1.49(2)
C(23)-H(23)	0.9500
C(24)-C(25)	1.29(3)
C(24)-H(24)	0.9500
C(25)-C(26)	1.36(3)
C(25)-H(25)	0.9500
C(26)-C(27)	1.59(3)
C(26)-H(26)	0.9500
C(27)-H(27)	0.9500
C(28)-C(33)	1.34(2)
C(28)-N(3)	1.462(17)
C(28)-C(29)	1.48(2)
C(29)-C(30)	1.28(3)
C(29)-H(29)	0.9500
C(30)-C(31)	1.39(3)

C(30)-H(30)	0.9500
C(31)-C(32)	1.35(2)
C(31)-H(31)	0.9500
C(32)-C(33)	1.473(18)
C(32)-H(32)	0.9500
C(33)-H(33)	0.9500
C(34)-N(3)	1.48(2)
C(34)-C(41)	1.463(19)
C(34)-C(35)	1.50(2)
C(34)-H(34)	1.0000
C(35)-C(36)	1.28(2)
C(35)-C(40)	1.48(2)
C(36)-C(37)	1.24(2)
C(36)-H(36)	0.9500
C(37)-C(38)	1.36(3)
C(37)-H(37)	0.9500
C(38)-C(39)	1.43(3)
C(38)-H(38)	0.9500
C(39)-C(40)	1.33(2)
C(39)-H(39)	0.9500
C(40)-H(40)	0.9500
C(41)-N(4)	1.366(15)
C(41)-C(48)	1.568(19)
C(42)-C(47)	1.31(3)
C(42)-N(4)	1.37(2)
C(42)-C(43)	1.44(2)
C(43)-C(44)	1.51(3)
C(43)-H(43)	0.9500
C(44)-C(45)	1.04(3)
C(44)-H(44)	0.9500
C(45)-C(46)	1.50(2)
C(45)-H(45)	0.9500
C(46)-C(47)	1.39(2)
C(46)-H(46)	0.9500
C(47)-H(47)	0.9500
C(48)-C(49)	1.393(19)
C(48)-O(3)	1.526(14)
C(48)-H(48)	1.0000

C(49)-C(50)	1.23(2)
C(49)-C(54)	1.54(2)
C(50)-C(51)	1.43(2)
C(50)-H(50)	0.9500
C(51)-C(52)	1.48(2)
C(51)-H(51)	0.9500
C(52)-C(53)	1.24(2)
C(52)-H(52)	0.9500
C(53)-C(54)	1.50(2)
C(53)-H(53)	0.9500
C(54)-H(54)	0.9500
C(55)-Cl(2)	1.78(3)
C(55)-Cl(1)	1.791(16)
C(55)-H(55A)	0.9900
C(55)-H(55B)	0.9900
C(56)-Cl(4)	1.53(2)
C(56)-Cl(3)	1.788(16)
C(56)-H(56A)	0.9900
C(56)-H(56B)	0.9900
N(1)-O(1)	1.547(17)
N(2)-O(2)	1.388(18)
N(3)-O(3)	1.425(15)
N(4)-O(4)	1.288(18)
C(6)-C(1)-N(1)	129.1(17)
C(6)-C(1)-C(2)	113.9(15)
N(1)-C(1)-C(2)	116.9(15)
C(3)-C(2)-C(1)	120(2)
C(3)-C(2)-H(2)	119.9
C(1)-C(2)-H(2)	120.1
C(2)-C(3)-C(4)	123(2)
C(2)-C(3)-H(3)	118.5
C(4)-C(3)-H(3)	118.3
C(3)-C(4)-C(5)	118.9(18)
C(3)-C(4)-H(4)	120.6
C(5)-C(4)-H(4)	120.5
C(6)-C(5)-C(4)	114.9(15)
C(6)-C(5)-H(5)	122.5
C(4)-C(5)-H(5)	122.6

C(1)-C(6)-C(5)	129.2(18)
C(1)-C(6)-H(6)	115.3
C(5)-C(6)-H(6)	115.5
O(1)-C(7)-C(14)	104.3(14)
O(1)-C(7)-C(8)	107.9(11)
C(14)-C(7)-C(8)	111.7(13)
O(1)-C(7)-H(7)	110.9
C(14)-C(7)-H(7)	110.9
C(8)-C(7)-H(7)	110.9
C(13)-C(8)-C(9)	125.8(13)
C(13)-C(8)-C(7)	121.3(13)
C(9)-C(8)-C(7)	111.5(12)
C(10)-C(9)-C(8)	108.3(14)
C(10)-C(9)-H(9)	125.8
C(8)-C(9)-H(9)	125.9
C(11)-C(10)-C(9)	122(2)
C(11)-C(10)-H(10)	119.1
C(9)-C(10)-H(10)	119.0
C(10)-C(11)-C(12)	129.0(18)
C(10)-C(11)-H(11)	115.4
C(12)-C(11)-H(11)	115.6
C(13)-C(12)-C(11)	104.0(17)
C(13)-C(12)-H(12)	128.1
C(11)-C(12)-H(12)	127.9
C(12)-C(13)-C(8)	129.2(16)
C(12)-C(13)-H(13)	115.3
C(8)-C(13)-H(13)	115.5
N(2)-C(14)-C(7)	129.5(17)
N(2)-C(14)-C(21)	115.8(17)
C(7)-C(14)-C(21)	108.1(14)
C(20)-C(15)-C(16)	136.0(14)
C(20)-C(15)-N(2)	107.3(14)
C(16)-C(15)-N(2)	116.7(11)
C(15)-C(16)-C(17)	103.1(13)
C(15)-C(16)-H(16)	128.5
C(17)-C(16)-H(16)	128.4
C(18)-C(17)-C(16)	138(2)
C(18)-C(17)-H(17)	111.1

C(16)-C(17)-H(17)	111.2
C(17)-C(18)-C(19)	110.3(17)
C(17)-C(18)-H(18)	124.9
C(19)-C(18)-H(18)	124.8
C(20)-C(19)-C(18)	124.6(16)
C(20)-C(19)-H(19)	117.7
C(18)-C(19)-H(19)	117.7
C(15)-C(20)-C(19)	106.9(18)
C(15)-C(20)-H(20)	126.5
C(19)-C(20)-H(20)	126.5
N(1)-C(21)-C(14)	96.9(13)
N(1)-C(21)-C(22)	107.9(11)
C(14)-C(21)-C(22)	108.4(11)
N(1)-C(21)-H(21)	114.0
C(14)-C(21)-H(21)	114.0
C(22)-C(21)-H(21)	114.1
C(27)-C(22)-C(23)	125.6(14)
C(27)-C(22)-C(21)	119.6(14)
C(23)-C(22)-C(21)	114.7(11)
C(24)-C(23)-C(22)	108.8(14)
C(24)-C(23)-H(23)	125.6
C(22)-C(23)-H(23)	125.5
C(25)-C(24)-C(23)	123.0(18)
C(25)-C(24)-H(24)	118.5
C(23)-C(24)-H(24)	118.5
C(24)-C(25)-C(26)	131.9(19)
C(24)-C(25)-H(25)	114.2
C(26)-C(25)-H(25)	114.0
C(25)-C(26)-C(27)	111.9(18)
C(25)-C(26)-H(26)	124.2
C(27)-C(26)-H(26)	123.9
C(22)-C(27)-C(26)	118.0(18)
C(22)-C(27)-H(27)	120.9
C(26)-C(27)-H(27)	121.1
C(33)-C(28)-N(3)	122.5(13)
C(33)-C(28)-C(29)	124.8(13)
N(3)-C(28)-C(29)	111.5(13)
C(30)-C(29)-C(28)	113(2)

C(30)-C(29)-H(29)	123.2
C(28)-C(29)-H(29)	123.6
C(31)-C(30)-C(29)	124(3)
C(31)-C(30)-H(30)	117.9
C(29)-C(30)-H(30)	118.3
C(30)-C(31)-C(32)	125.1(17)
C(30)-C(31)-H(31)	117.5
C(32)-C(31)-H(31)	117.4
C(31)-C(32)-C(33)	113.6(14)
C(31)-C(32)-H(32)	123.2
C(33)-C(32)-H(32)	123.1
C(28)-C(33)-C(32)	118.1(13)
C(28)-C(33)-H(33)	120.9
C(32)-C(33)-H(33)	120.9
N(3)-C(34)-C(41)	100.6(12)
N(3)-C(34)-C(35)	116.1(12)
C(41)-C(34)-C(35)	115.8(11)
N(3)-C(34)-H(34)	108.0
C(41)-C(34)-H(34)	107.9
C(35)-C(34)-H(34)	107.9
C(36)-C(35)-C(40)	114.1(13)
C(36)-C(35)-C(34)	133.4(15)
C(40)-C(35)-C(34)	112.4(13)
C(37)-C(36)-C(35)	130.9(16)
C(37)-C(36)-H(36)	114.5
C(35)-C(36)-H(36)	114.6
C(36)-C(37)-C(38)	120.4(18)
C(36)-C(37)-H(37)	119.7
C(38)-C(37)-H(37)	119.8
C(37)-C(38)-C(39)	114.5(16)
C(37)-C(38)-H(38)	122.7
C(39)-C(38)-H(38)	122.9
C(40)-C(39)-C(38)	123.5(14)
C(40)-C(39)-H(39)	118.3
C(38)-C(39)-H(39)	118.1
C(39)-C(40)-C(35)	116.5(14)
C(39)-C(40)-H(40)	121.7
C(35)-C(40)-H(40)	121.8

N(4)-C(41)-C(34)	126.8(12)
N(4)-C(41)-C(48)	120.2(11)
C(34)-C(41)-C(48)	112.3(11)
C(47)-C(42)-N(4)	124.2(18)
C(47)-C(42)-C(43)	107.1(17)
N(4)-C(42)-C(43)	127.9(18)
C(42)-C(43)-C(44)	124.6(17)
C(42)-C(43)-H(43)	117.7
C(44)-C(43)-H(43)	117.7
C(45)-C(44)-C(43)	113(2)
C(45)-C(44)-H(44)	123.4
C(43)-C(44)-H(44)	123.2
C(44)-C(45)-C(46)	135(2)
C(44)-C(45)-H(45)	112.4
C(46)-C(45)-H(45)	112.5
C(47)-C(46)-C(45)	105.8(16)
C(47)-C(46)-H(46)	127.1
C(45)-C(46)-H(46)	127.1
C(42)-C(47)-C(46)	134.0(18)
C(42)-C(47)-H(47)	113.0
C(46)-C(47)-H(47)	113.0
C(49)-C(48)-O(3)	106.4(10)
C(49)-C(48)-C(41)	124.1(12)
O(3)-C(48)-C(41)	98.2(9)
C(49)-C(48)-H(48)	108.9
O(3)-C(48)-H(48)	108.9
C(41)-C(48)-H(48)	109.0
C(50)-C(49)-C(48)	126.5(15)
C(50)-C(49)-C(54)	120.1(14)
C(48)-C(49)-C(54)	112.1(13)
C(49)-C(50)-C(51)	126.9(17)
C(49)-C(50)-H(50)	116.5
C(51)-C(50)-H(50)	116.6
C(50)-C(51)-C(52)	117.8(15)
C(50)-C(51)-H(51)	121.1
C(52)-C(51)-H(51)	121.1
C(53)-C(52)-C(51)	113.6(15)
C(53)-C(52)-H(52)	123.1

C(51)-C(52)-H(52)	123.3
C(52)-C(53)-C(54)	131.5(16)
C(52)-C(53)-H(53)	114.3
C(54)-C(53)-H(53)	114.2
C(53)-C(54)-C(49)	107.9(14)
C(53)-C(54)-H(54)	126.1
C(49)-C(54)-H(54)	126.0
Cl(2)-C(55)-Cl(1)	105.5(12)
Cl(2)-C(55)-H(55A)	110.8
Cl(1)-C(55)-H(55A)	110.8
Cl(2)-C(55)-H(55B)	110.5
Cl(1)-C(55)-H(55B)	110.6
H(55A)-C(55)-H(55B)	108.7
Cl(4)-C(56)-Cl(3)	124.5(12)
Cl(4)-C(56)-H(56A)	106.2
Cl(3)-C(56)-H(56A)	106.2
Cl(4)-C(56)-H(56B)	106.2
Cl(3)-C(56)-H(56B)	106.2
H(56A)-C(56)-H(56B)	106.4
C(21)-N(1)-C(1)	124.9(13)
C(21)-N(1)-O(1)	102.6(10)
C(1)-N(1)-O(1)	99.9(10)
C(14)-N(2)-O(2)	122.5(13)
C(14)-N(2)-C(15)	119.5(15)
O(2)-N(2)-C(15)	109.4(10)
O(3)-N(3)-C(28)	110.3(9)
O(3)-N(3)-C(34)	109.5(10)
C(28)-N(3)-C(34)	114.5(12)
O(4)-N(4)-C(41)	117.8(11)
O(4)-N(4)-C(42)	121.4(13)
C(41)-N(4)-C(42)	119.4(13)
C(7)-O(1)-N(1)	103.5(10)
N(3)-O(3)-C(48)	104.7(8)

Symmetry transformations used to generate equivalent atoms:

Table 4. Anisotropic displacement parameters (Å2x 103)for d18485f. The anisotropic displacement factor exponent takes the form: -2□2[h2a*2U11 + ... + 2 h k a* b* U12]

	U11	U22	U33	U23
C(56)	29(7)	51(9)	44(7)	-4(7)
Cl(1)	76(3)	71(3)	91(4)	-14(3)
Cl(2)	83(4)	91(4)	81(3)	-18(3)
Cl(3)	141(6)	124(6)	95(4)	-47(5)
Cl(4)	203(10)	134(8)	200(9)	-97(8)

Table 5. Hydrogen coordinates (x 104) and isotropic displacement parameters (Å2x 103) for d18485f.

	x	y	z	U(eq)
H(2)	8226	-2213	-558	76
H(3)	8366	-2703	-1450	119
H(4)	7968	16	-2057	68
H(5)	7330	3387	-1743	48
H(6)	7264	3837	-832	62
H(7)	7063	4139	641	47
H(9)	5941	6074	1055	45
H(10)	4428	6255	1153	84
H(11)	3667	3571	886	71
H(12)	4170	-341	546	78
H(13)	5535	87	292	46
H(16)	7581	3606	1618	43
H(17)	6838	5236	2352	82
H(18)	5995	3819	2859	54
H(19)	5355	230	2507	70
H(20)	6084	-2115	1834	80
H(21)	7374	-1881	194	37
H(23)	8603	-4592	216	50
H(24)	10094	-4790	410	72
H(25)	10715	-2201	841	58
H(26)	10294	1412	1005	82
H(27)	8722	1994	894	76
H(29)	7283	-2111	4131	53
H(30)	7638	-1617	3277	116

H(31)	8017	1829	2956	54
H(32)	8526	4683	3491	46
H(33)	8350	4084	4458	38
H(34)	7406	3850	5117	48
H(36)	8642	6276	5177	49
H(37)	9824	7185	5438	49
H(38)	10663	4556	5877	75
H(39)	10067	928	6050	41
H(40)	8785	32	5766	43
H(43)	5873	3572	6858	49
H(44)	5366	1445	7634	99
H(45)	5890	-1393	7793	67
H(46)	6997	-3315	7336	57
H(47)	7430	-1252	6600	65
H(48)	7063	-1838	5745	37
H(50)	5970	-3754	5995	61
H(51)	4598	-4105	6231	45
H(52)	3670	-860	6041	60
H(53)	4142	1552	5472	49
H(54)	5597	2539	5386	57
H(55A)	11141	3264	2143	99
H(55B)	11904	2784	2532	99
H(56A)	8912	-2936	1627	49
H(56B)	9781	-2825	1905	49

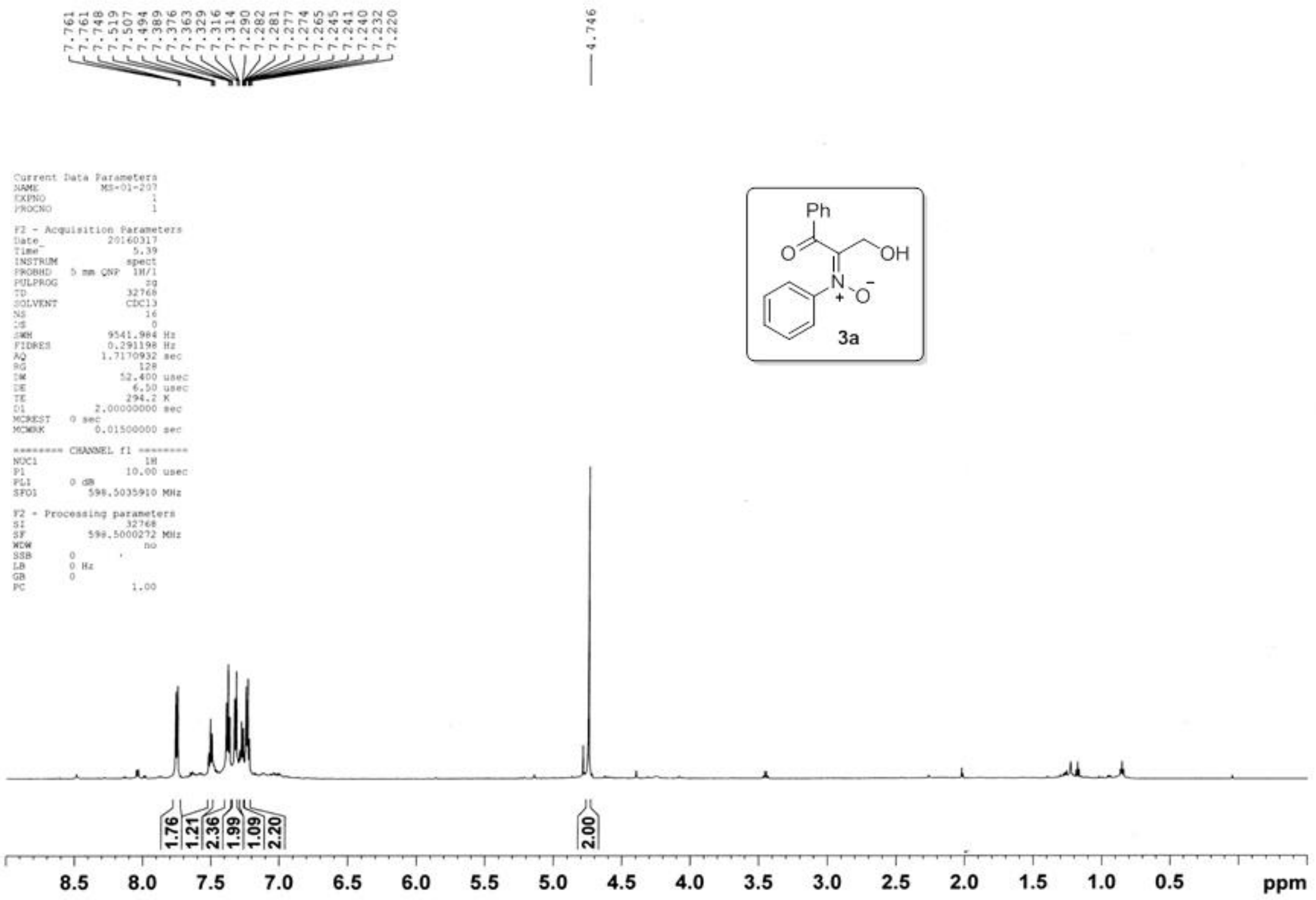
7.761
7.761
7.748
7.519
7.507
7.494
7.389
7.376
7.363
7.329
7.316
7.314
7.290
7.282
7.281
7.277
7.274
7.265
7.245
7.241
7.240
7.232
7.220
4.746
Current Data Parameters
NAME MS-01-207
EXPNO 1
PROCNO 1
F2 - Acquisition Parameters
Date_ 20160317
Time 5.39
INSTRUM spect
PROBHD 5 mm QNP 1H/1
PULPROG zg
TD 32768
SOLVENT CDCl3
NS 16
DS 0
SWH 9541.984 Hz
FIDRES 0.291198 Hz
AQ 1.7170932 sec
RG 128
DW 52.400 usec
DE 6.50 usec
TE 294.2 K
D1 2.00000000 sec
MCREST 0 sec
MCWRK 0.01500000 sec
======== CHANNEL f1 ========
NUC1 1H
P1 10.00 usec
PL1 0 dB
SFO1 598.5035910 MHz
F2 - Processing parameters
SI 32768
SF 598.5000272 MHz
WDW no
SSB 0
LB 0 Hz
GB 0
PC 1.00
Ph
O
OH
N
+
O
3a
1.76
1.21
2.36
1.99
1.09
2.20
2.00
8.5
8.0
7.5
7.0
6.5
6.0
5.5
5.0
4.5
4.0
3.5
3.0
2.5
2.0
1.5
1.0
0.5
ppm

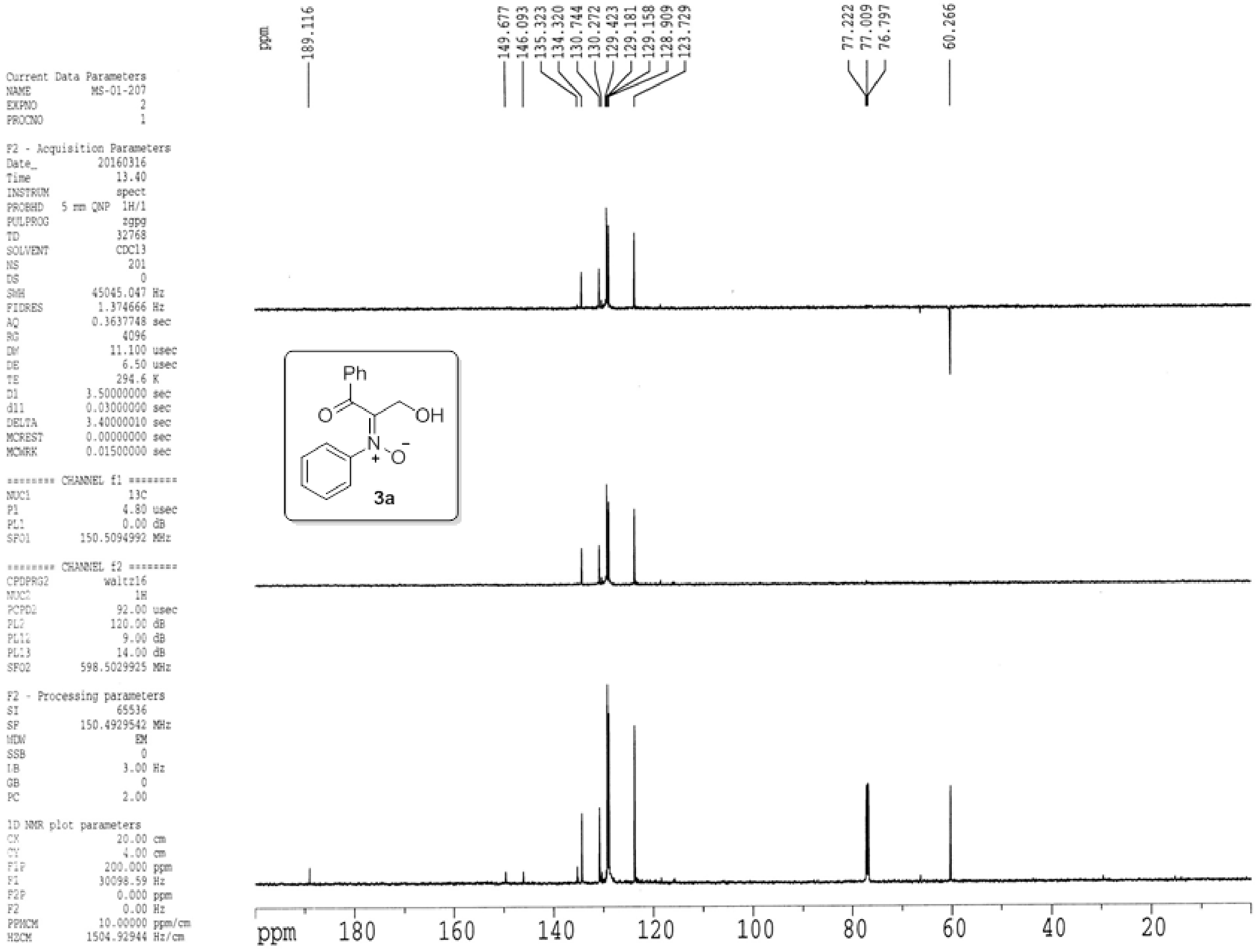
Current Data Parameters
NAME MS-01-207
EXPNO 2
PROCNO 1
F2 - Acquisition Parameters
Date_ 20160316
Time 13.40
INSTRUM spect
PROBHD 5 mm QNP 1H/1
PULPROG zgpg
TD 32768
SOLVENT CDCl3
NS 201
DS 0
SWH 45045.047 Hz
FIDRES 1.374666 Hz
AQ 0.3637748 sec
RG 4096
DW 11.100 usec
DE 6.50 usec
TE 294.6 K
D1 3.50000000 sec
d11 0.03000000 sec
DELTA 3.40000010 sec
MCREST 0.00000000 sec
MCWRK 0.01500000 sec
======== CHANNEL f1 ========
NUC1 13C
P1 4.80 usec
PL1 0.00 dB
SFO1 150.5094992 MHz
======== CHANNEL f2 ========
CPDPRG2 waltz16
NUC2 1H
PCPD2 92.00 usec
PL2 120.00 dB
PL12 9.00 dB
PL13 14.00 dB
SFO2 598.5029925 MHz
F2 - Processing parameters
SI 65536
SF 150.4929542 MHz
WDW EM
SSB 0
LB 3.00 Hz
GB 0
PC 2.00
1D NMR plot parameters
CX 20.00 cm
CY 4.00 cm
F1P 200.000 ppm
F1 30098.59 Hz
F2P 0.000 ppm
F2 0.00 Hz
PPMCM 10.00000 ppm/cm
HZCM 1504.92944 Hz/cm
ppm
189.116
149.677
146.093
135.323
134.320
130.744
130.272
129.423
129.181
129.158
128.909
123.729
77.222
77.009
76.797
60.266
Ph
O
OH
N
+
O
-
3a
ppm 180 160 140 120 100 80 60 40 20

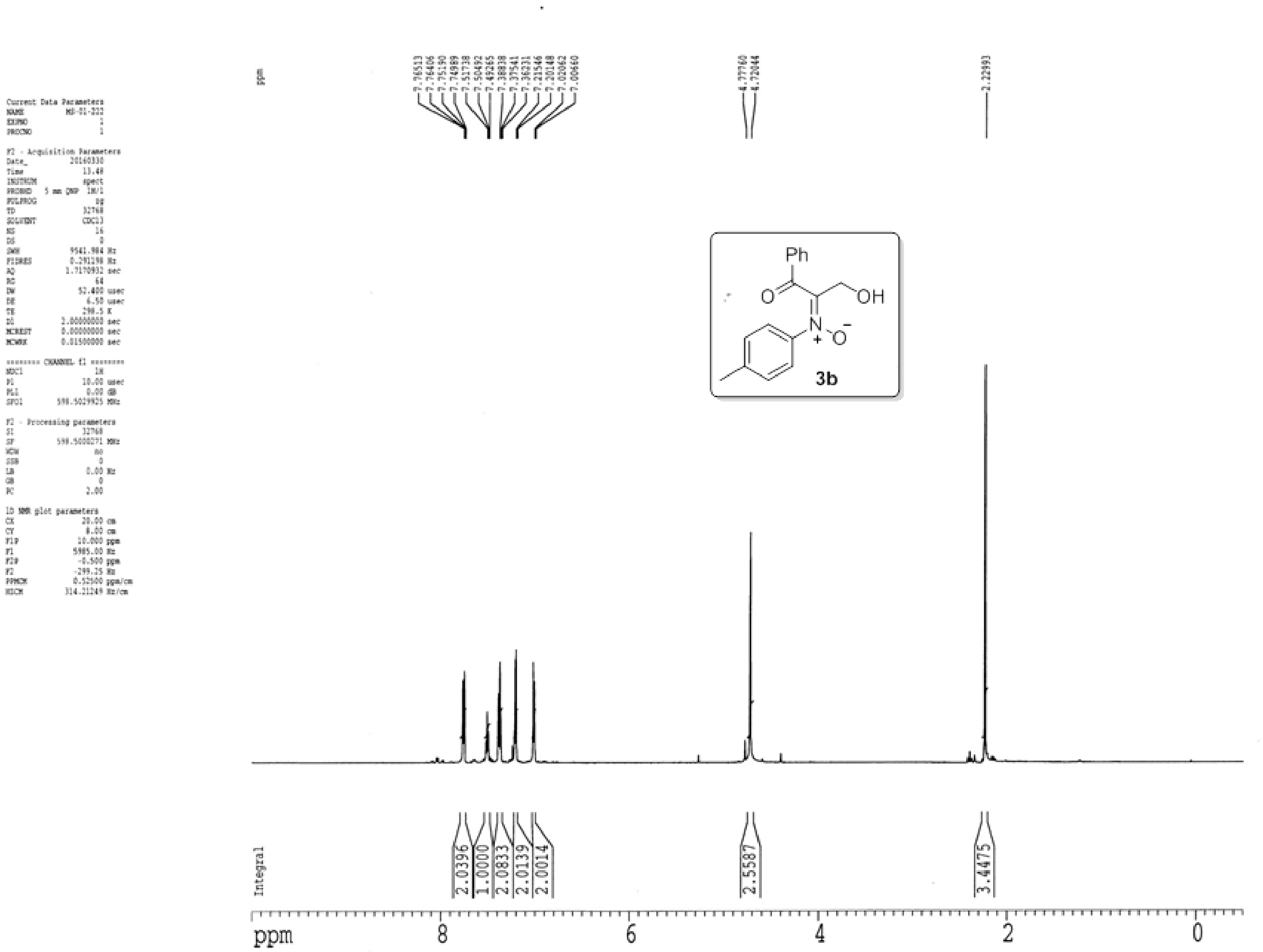
Ph
O
OH
N
O
3b
Integral
2.0396
1.0000
2.0833
2.0139
2.0014
2.5587
3.4475
ppm
8
6
4
2
0

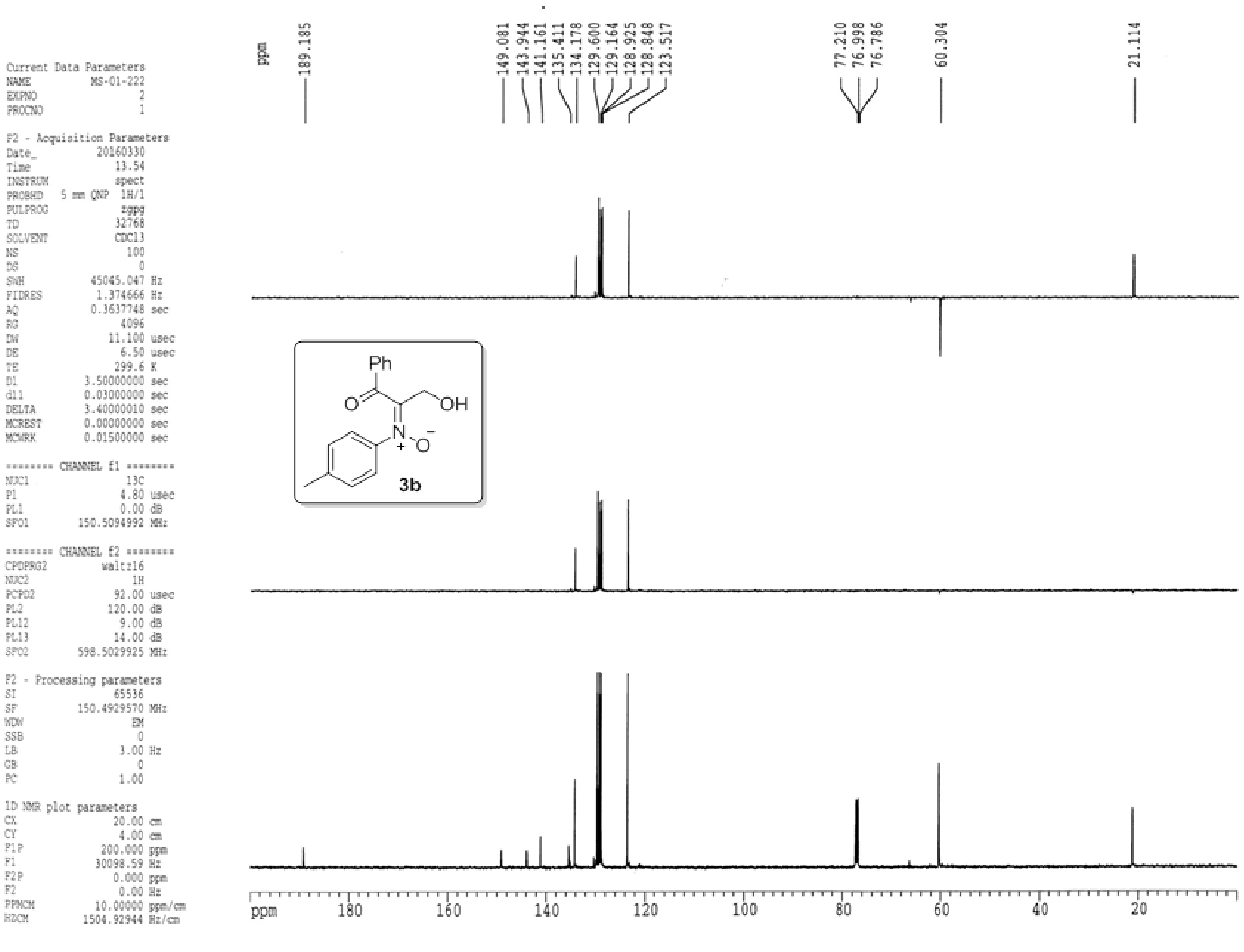
Current Data Parameters
NAME MS-01-222
EXPNO 2
PROCNO 1
F2 - Acquisition Parameters
Date_ 20160330
Time 13.54
INSTRUM spect
PROBHD 5 mm QNP 1H/1
PULPROG zgpg
TD 32768
SOLVENT CDCl3
NS 100
DS 0
SWH 45045.047 Hz
FIDRES 1.374666 Hz
AQ 0.3637748 sec
RG 4096
DW 11.100 usec
DE 6.50 usec
TE 299.6 K
D1 3.50000000 sec
d11 0.03000000 sec
DELTA 3.40000010 sec
MCREST 0.00000000 sec
MCWRK 0.01500000 sec
======== CHANNEL f1 ========
NUC1 13C
P1 4.80 usec
PL1 0.00 dB
SFO1 150.5094992 MHz
======== CHANNEL f2 ========
CPDPRG2 waltz16
NUC2 1H
PCPD2 92.00 usec
PL2 120.00 dB
PL12 9.00 dB
PL13 14.00 dB
SFO2 598.5029925 MHz
F2 - Processing parameters
SI 65536
SF 150.4929570 MHz
WDW EM
SSB 0
LB 3.00 Hz
GB 0
PC 1.00
1D NMR plot parameters
CX 20.00 cm
CY 4.00 cm
F1P 200.000 ppm
F1 30098.59 Hz
F2P 0.000 ppm
F2 0.00 Hz
PPMCM 10.00000 ppm/cm
HZCM 1504.92944 Hz/cm
ppm
189.185
149.081
143.944
141.161
135.411
134.178
129.600
129.164
128.925
128.848
123.517
77.210
76.998
76.786
60.304
21.114
Ph
O
OH
N
+
O
−
3b
ppm
180
160
140
120
100
80
60
40
20

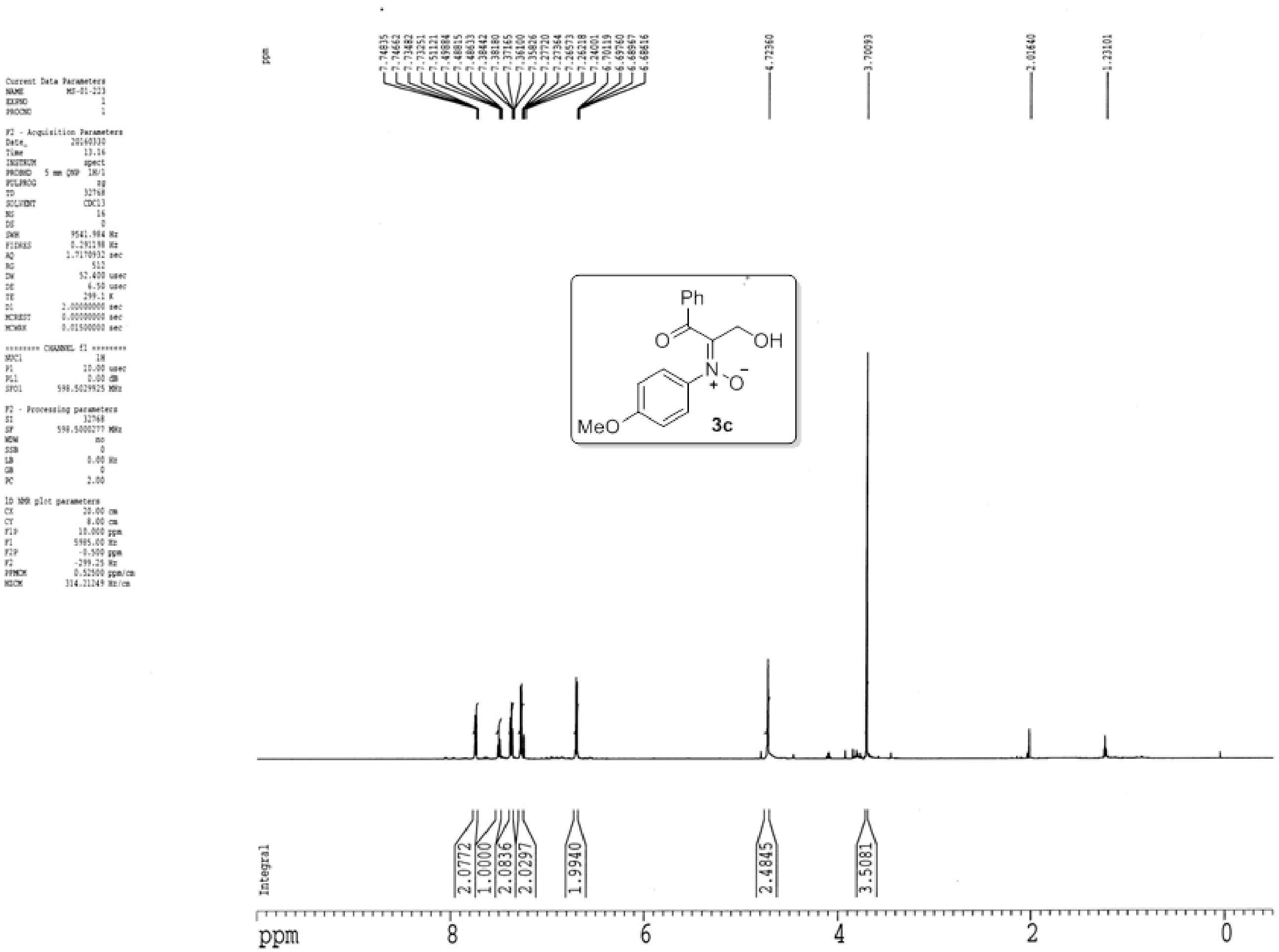
Current Data Parameters
NAME MS-01-223
EXPNO 1
PROCNO 1
F2 - Acquisition Parameters
Date_ 20160330
Time 13.16
INSTRUM spect
PROBHD 5 mm QNP 1H/1
PULPROG zg
TD 32768
SOLVENT CDCl3
NS 16
DS 0
SWH 9541.984 Hz
FIDRES 0.291198 Hz
AQ 1.7170932 sec
RG 512
DW 52.400 usec
DE 6.50 usec
TE 299.1 K
D1 2.00000000 sec
MCREST 0.00000000 sec
MCWRK 0.01500000 sec
======== CHANNEL f1 ========
NUC1 1H
P1 10.00 usec
PL1 0.00 dB
SFO1 598.5029925 MHz
F2 - Processing parameters
SI 32768
SF 598.5000277 MHz
WDW no
SSB 0
LB 0.00 Hz
GB 0
PC 2.00
1D NMR plot parameters
CX 20.00 cm
CY 8.00 cm
F1P 10.000 ppm
F1 5985.00 Hz
F2P -0.500 ppm
F2 -299.25 Hz
PPMCM 0.52500 ppm/cm
HZCM 314.21249 Hz/cm
ppm
7.74835
7.74662
7.73482
7.73251
7.51121
7.49884
7.48815
7.48633
7.38442
7.38180
7.37165
7.36100
7.35826
7.27720
7.27364
7.26573
7.26218
7.24001
6.70119
6.69760
6.68987
6.68616
4.72360
3.70093
2.01640
1.23101
Ph
O
OH
N
+
O
-
MeO
3c
Integral
2.0772
1.0000
2.0836
2.0297
1.9940
2.4845
3.5081
ppm
8
6
4
2
0

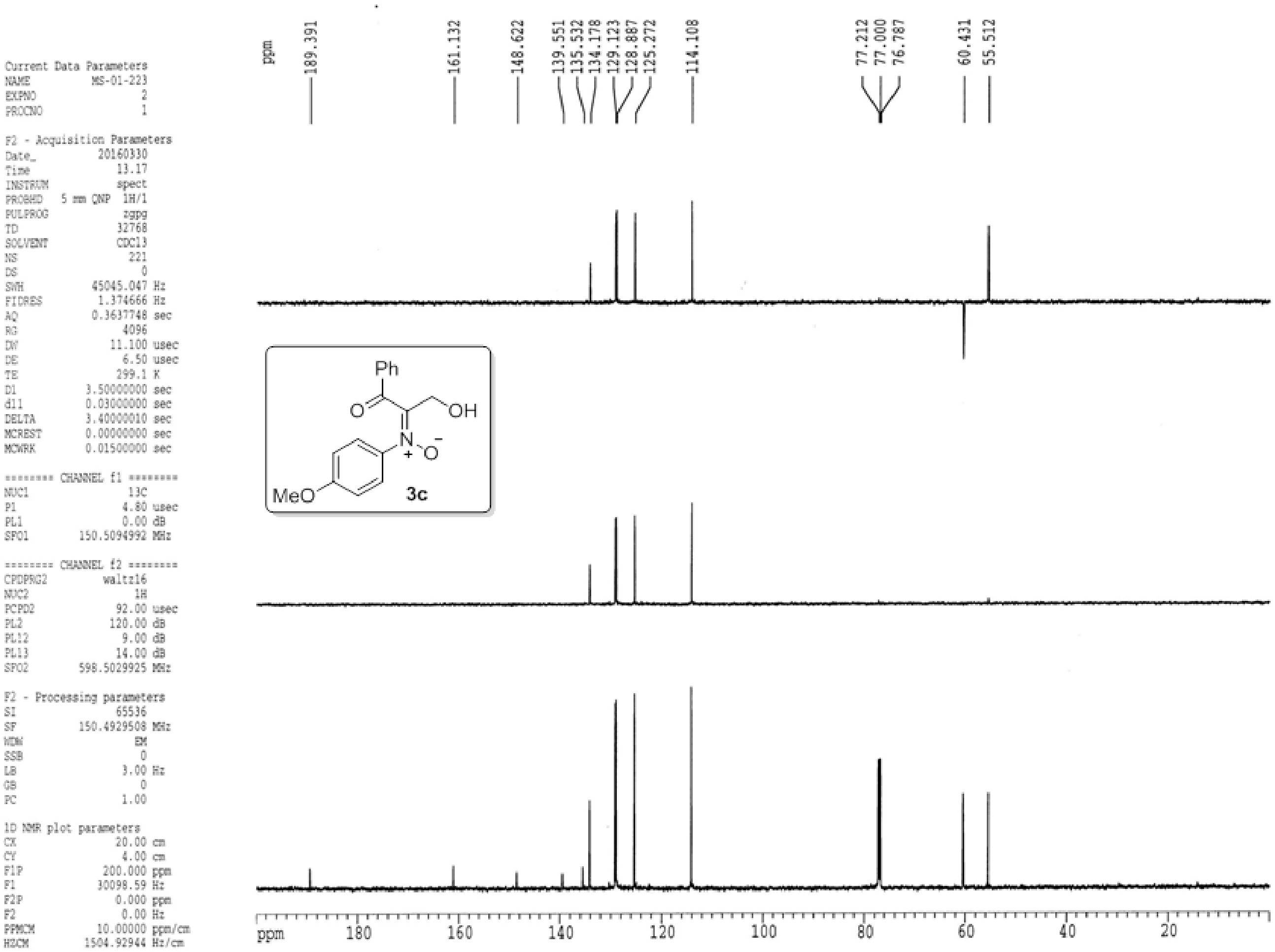
Current Data Parameters
NAME MS-01-223
EXPNO 2
PROCNO 1
F2 - Acquisition Parameters
Date_ 20160330
Time 13.17
INSTRUM spect
PROBHD 5 mm QNP 1H/1
PULPROG zgpg
TD 32768
SOLVENT CDCl3
NS 221
DS 0
SWH 45045.047 Hz
FIDRES 1.374666 Hz
AQ 0.3637748 sec
RG 4096
DW 11.100 usec
DE 6.50 usec
TE 299.1 K
D1 3.50000000 sec
d11 0.03000000 sec
DELTA 3.40000010 sec
MCREST 0.00000000 sec
MCWRK 0.01500000 sec
======== CHANNEL f1 ========
NUC1 13C
P1 4.80 usec
PL1 0.00 dB
SFO1 150.5094992 MHz
======== CHANNEL f2 ========
CPDPRG2 waltz16
NUC2 1H
PCPD2 92.00 usec
PL2 120.00 dB
PL12 9.00 dB
PL13 14.00 dB
SFO2 598.5029925 MHz
F2 - Processing parameters
SI 65536
SF 150.4929508 MHz
WDW EM
SSB 0
LB 3.00 Hz
GB 0
PC 1.00
1D NMR plot parameters
CX 20.00 cm
CY 4.00 cm
F1P 200.000 ppm
F1 30098.59 Hz
F2P 0.000 ppm
F2 0.00 Hz
PPMCM 10.00000 ppm/cm
HZCM 1504.92944 Hz/cm
ppm
189.391
161.132
148.622
139.551
135.532
134.178
129.123
128.887
125.272
114.108
77.212
77.000
76.787
60.431
55.512
Ph
O
OH
N
+
O
-
MeO
3c
ppm 180 160 140 120 100 80 60 40 20

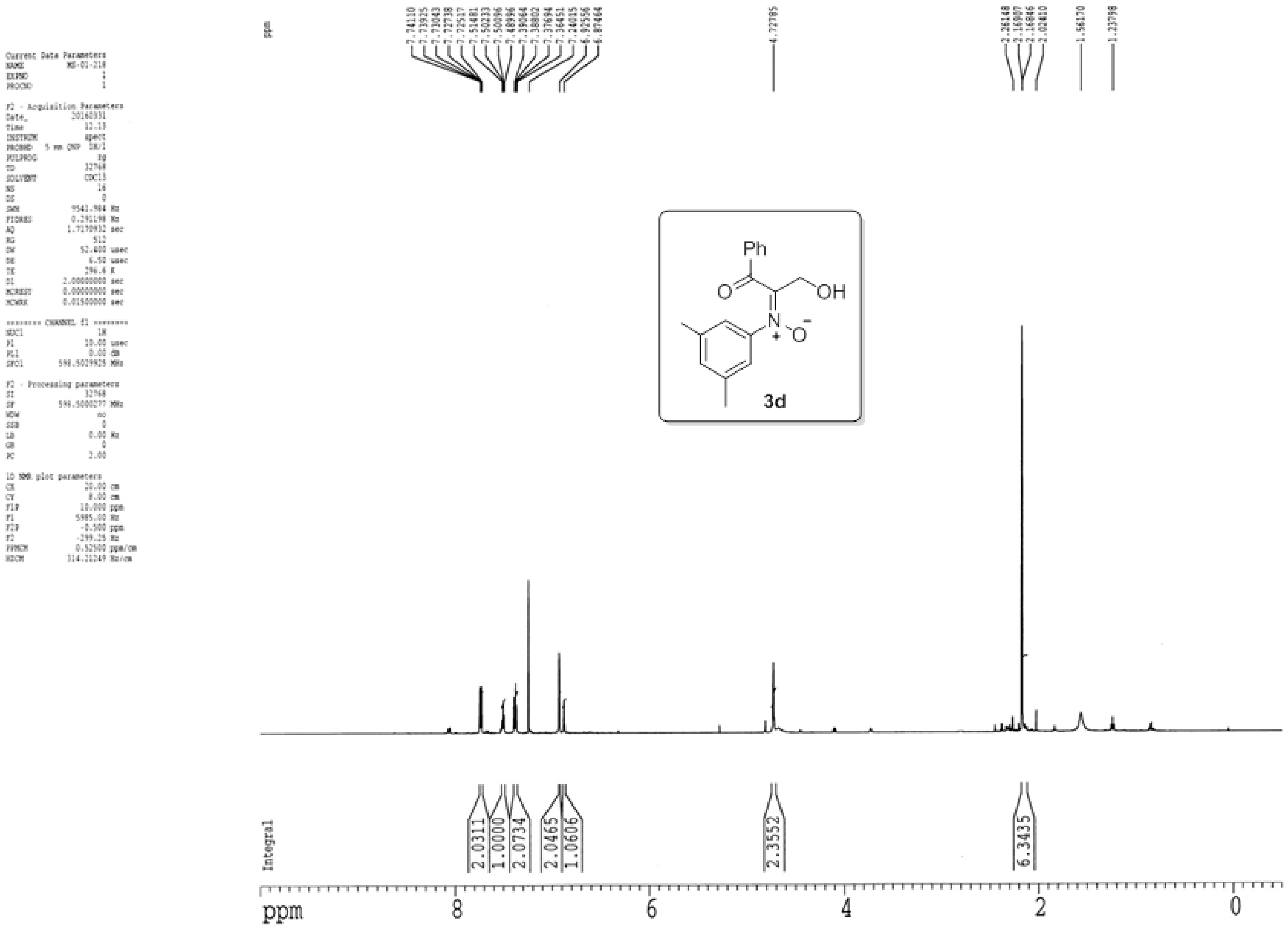
Current Data Parameters
NAME MS-01-218
EXPNO 1
PROCNO 1
F2 - Acquisition Parameters
Date_ 20160331
Time 12.13
INSTRUM spect
PROBHD 5 mm QNP 1H/1
PULPROG zg
TD 32768
SOLVENT CDCl3
NS 16
DS 0
SWH 9541.984 Hz
FIDRES 0.291198 Hz
AQ 1.7170932 sec
RG 512
DW 52.400 usec
DE 6.50 usec
TE 296.6 K
D1 2.00000000 sec
MCREST 0.00000000 sec
MCWRK 0.01500000 sec
======== CHANNEL f1 ========
NUC1 1H
P1 10.00 usec
PL1 0.00 dB
SFO1 598.5029925 MHz
F2 - Processing parameters
SI 32768
SF 598.5000277 MHz
WDW no
SSB 0
LB 0.00 Hz
GB 0
PC 2.00
1D NMR plot parameters
CX 20.00 cm
CY 8.00 cm
F1P 10.000 ppm
F1 5985.00 Hz
F2P -0.500 ppm
F2 -299.25 Hz
PPMCM 0.52500 ppm/cm
HZCM 314.21249 Hz/cm
ppm
7.74110
7.73925
7.73043
7.72738
7.72517
7.51481
7.50233
7.50096
7.48996
7.39064
7.38802
7.37694
7.36451
7.24015
6.92556
6.87464
4.72785
2.26148
2.16907
2.15846
2.02410
1.56170
1.23798
Ph
O
OH
N
+
O
-
3d
Integral
2.0311
1.0000
2.0734
2.0465
1.0606
2.3552
6.3435
ppm
8
6
4
2
0

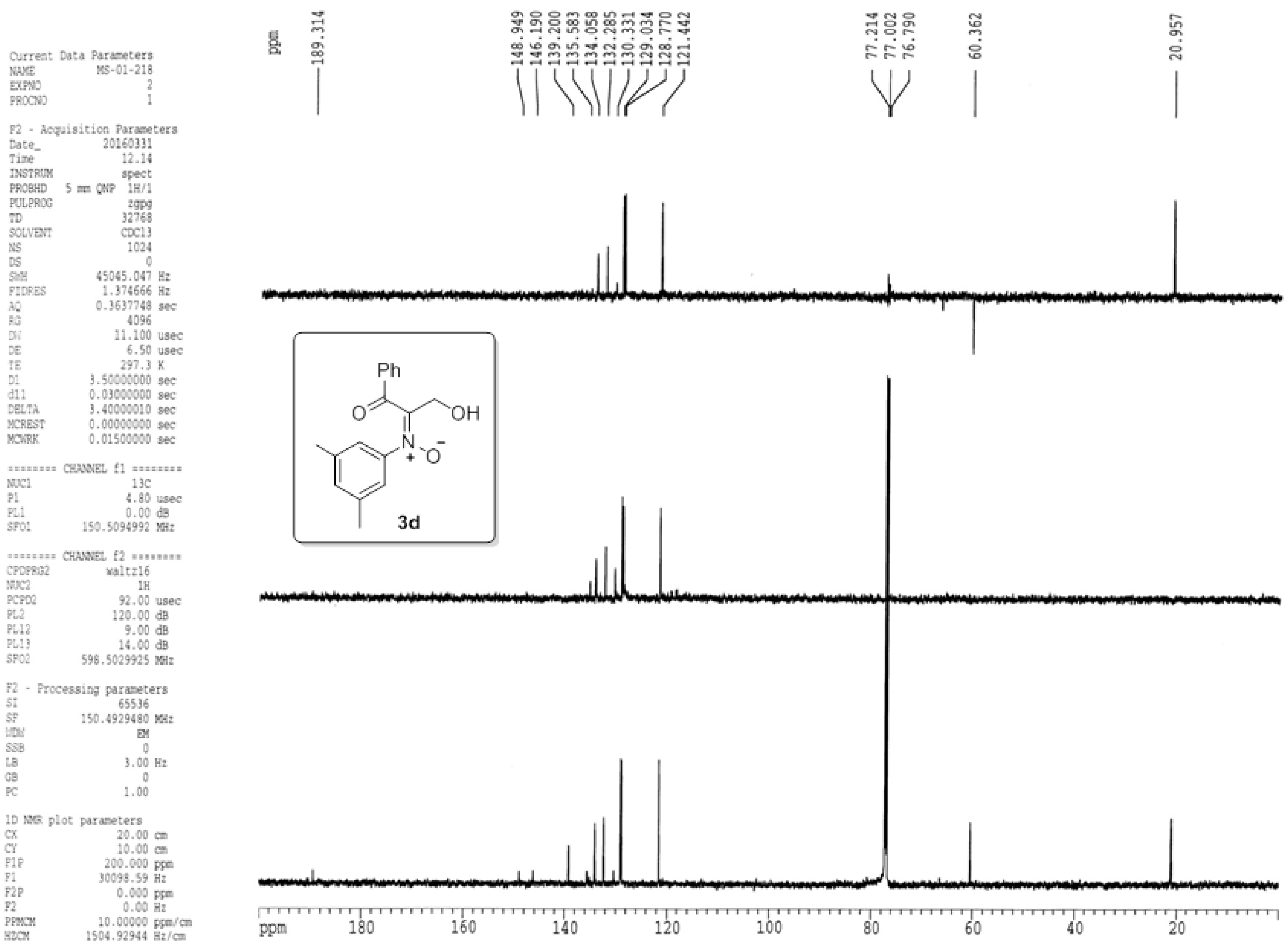

Current Data Parameters
NAME MS-01-218
EXPNO 2
PROCNO 1
F2 - Acquisition Parameters
Date_ 20160331
Time 12.14
INSTRUM spect
PROBHD 5 mm QNP 1H/1
PULPROG zgpg
TD 32768
SOLVENT CDCl3
NS 1024
DS 0
SWH 45045.047 Hz
FIDRES 1.374666 Hz
AQ 0.3637748 sec
RG 4096
DW 11.100 usec
DE 6.50 usec
TE 297.3 K
D1 3.50000000 sec
d11 0.03000000 sec
DELTA 3.40000010 sec
MCREST 0.00000000 sec
MCWRK 0.01500000 sec
======== CHANNEL f1 ========
NUC1 13C
P1 4.80 usec
PL1 0.00 dB
SFO1 150.5094992 MHz
======== CHANNEL f2 ========
CPDPRG2 waltz16
NUC2 1H
PCPD2 92.00 usec
PL2 120.00 dB
PL12 9.00 dB
PL13 14.00 dB
SFO2 598.5029925 MHz
F2 - Processing parameters
SI 65536
SF 150.4929480 MHz
WDW EM
SSB 0
LB 3.00 Hz
GB 0
PC 1.00
1D NMR plot parameters
CX 20.00 cm
CY 10.00 cm
F1P 200.000 ppm
F1 30098.59 Hz
F2P 0.000 ppm
F2 0.00 Hz
PPMCM 10.00000 ppm/cm
HZCM 1504.92944 Hz/cm
ppm
189.314
148.949
146.190
139.200
135.583
134.058
132.285
130.331
129.034
128.770
121.442
77.214
77.002
76.790
60.362
20.957
Ph
O
OH
N
+
O
-
3d
ppm 180 160 140 120 100 80 60 40 20

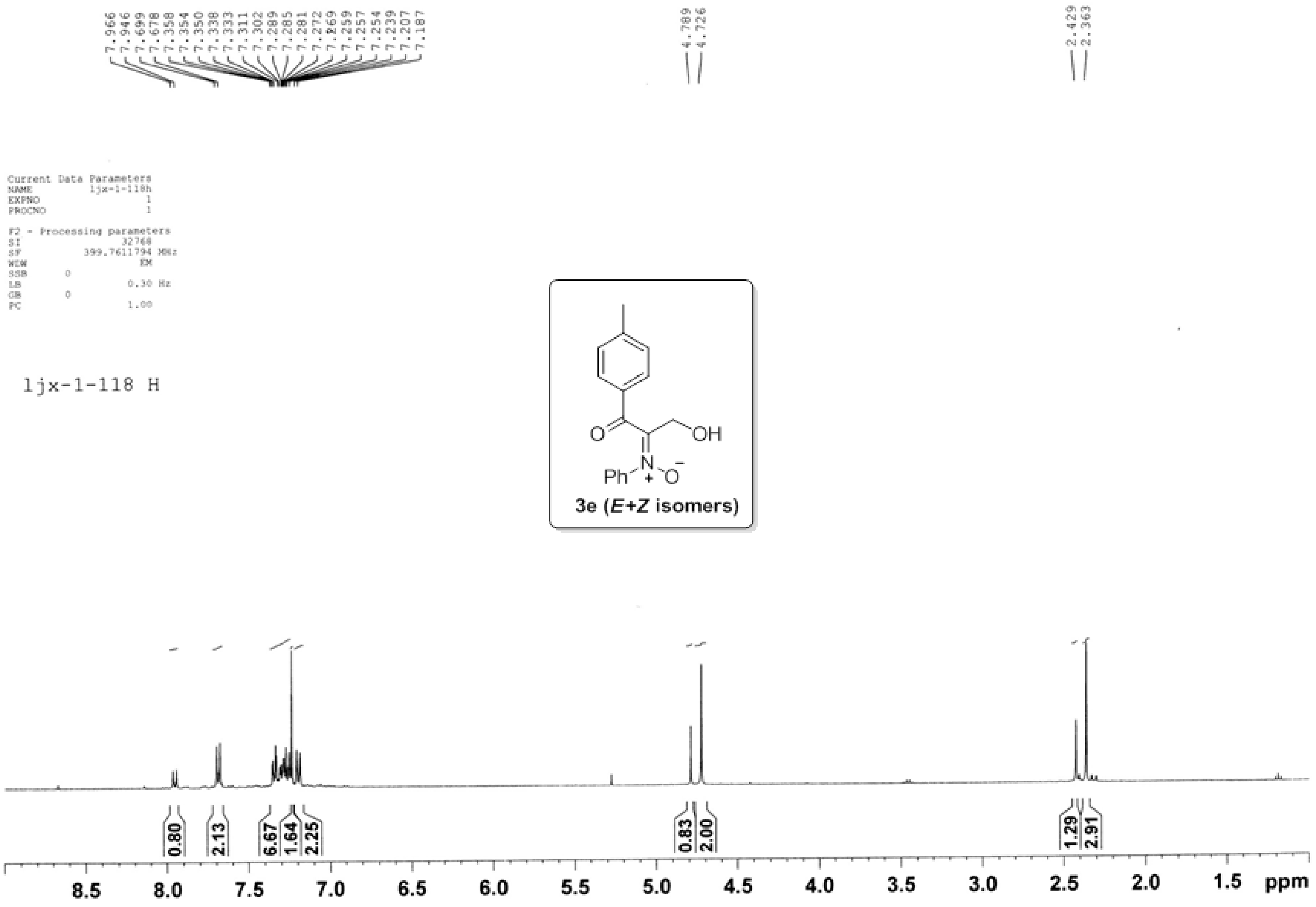
7.966 7.946 7.699 7.678 7.358 7.354 7.350 7.338 7.333 7.311 7.302 7.289 7.285 7.281 7.272 7.269 7.259 7.257 7.254 7.239 7.207 7.187
4.789 4.726
2.429 2.363
Current Data Parameters
NAME ljx-1-118h
EXPNO 1
PROCNO 1
F2 - Processing parameters
SI 32768
SF 399.7611794 MHz
WDW EM
SSB 0
LB 0.30 Hz
GB 0
PC 1.00
ljx-1-118 H
O
OH
N
Ph
O
3e (E+Z isomers)
0.80
2.13
6.67
1.64
2.25
0.83
2.00
1.29
2.91
8.5 8.0 7.5 7.0 6.5 6.0 5.5 5.0 4.5 4.0 3.5 3.0 2.5 2.0 1.5 ppm

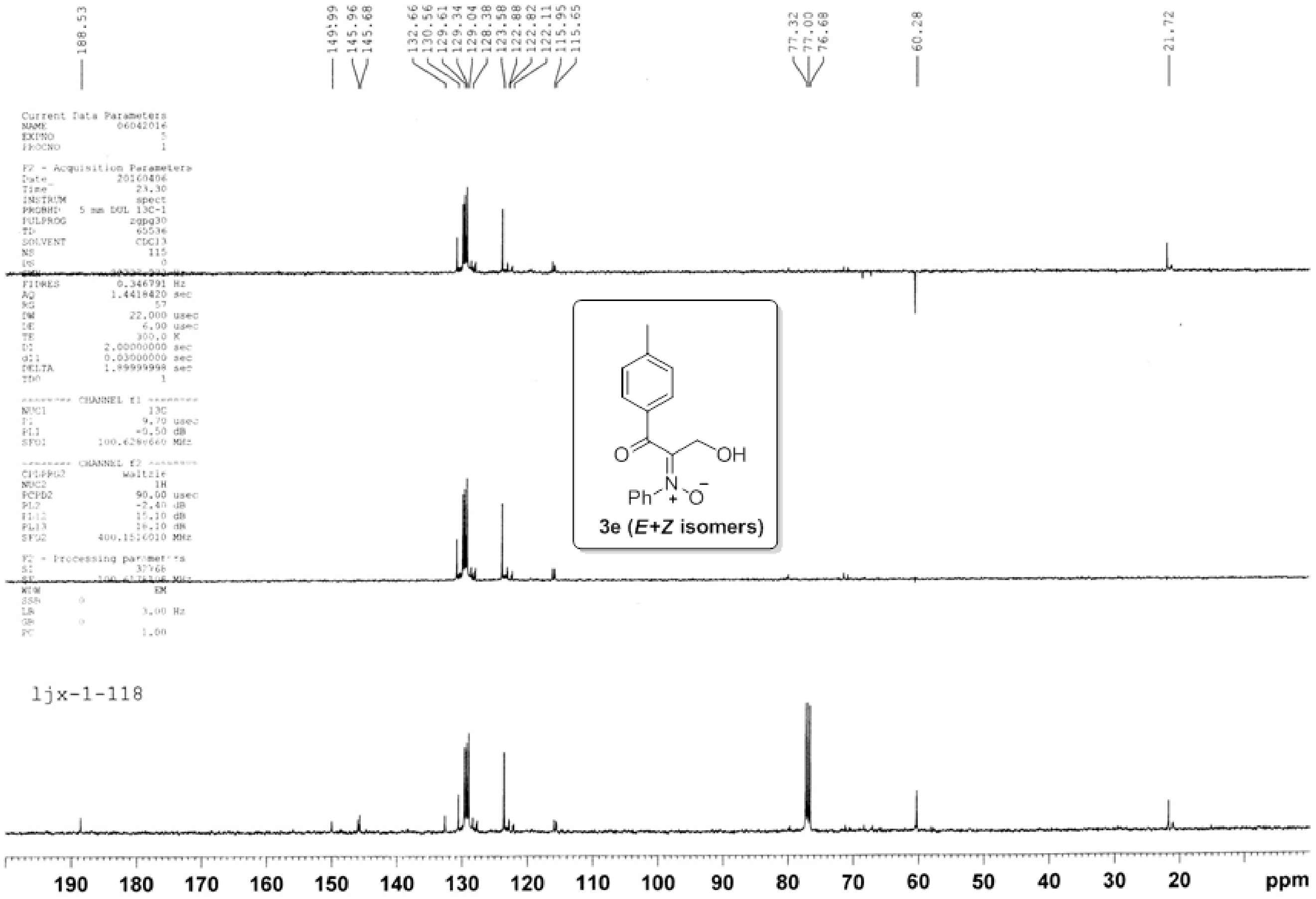
188.53
149.99
145.96
145.68
132.66
130.56
129.61
129.34
129.04
128.38
123.58
122.88
122.82
122.11
115.95
115.65
77.32
77.00
76.68
60.28
21.72
OH
O
N
Ph
3e (E+Z isomers)
ljx-1-118
190 180 170 160 150 140 130 120 110 100 90 80 70 60 50 40 30 20 ppm

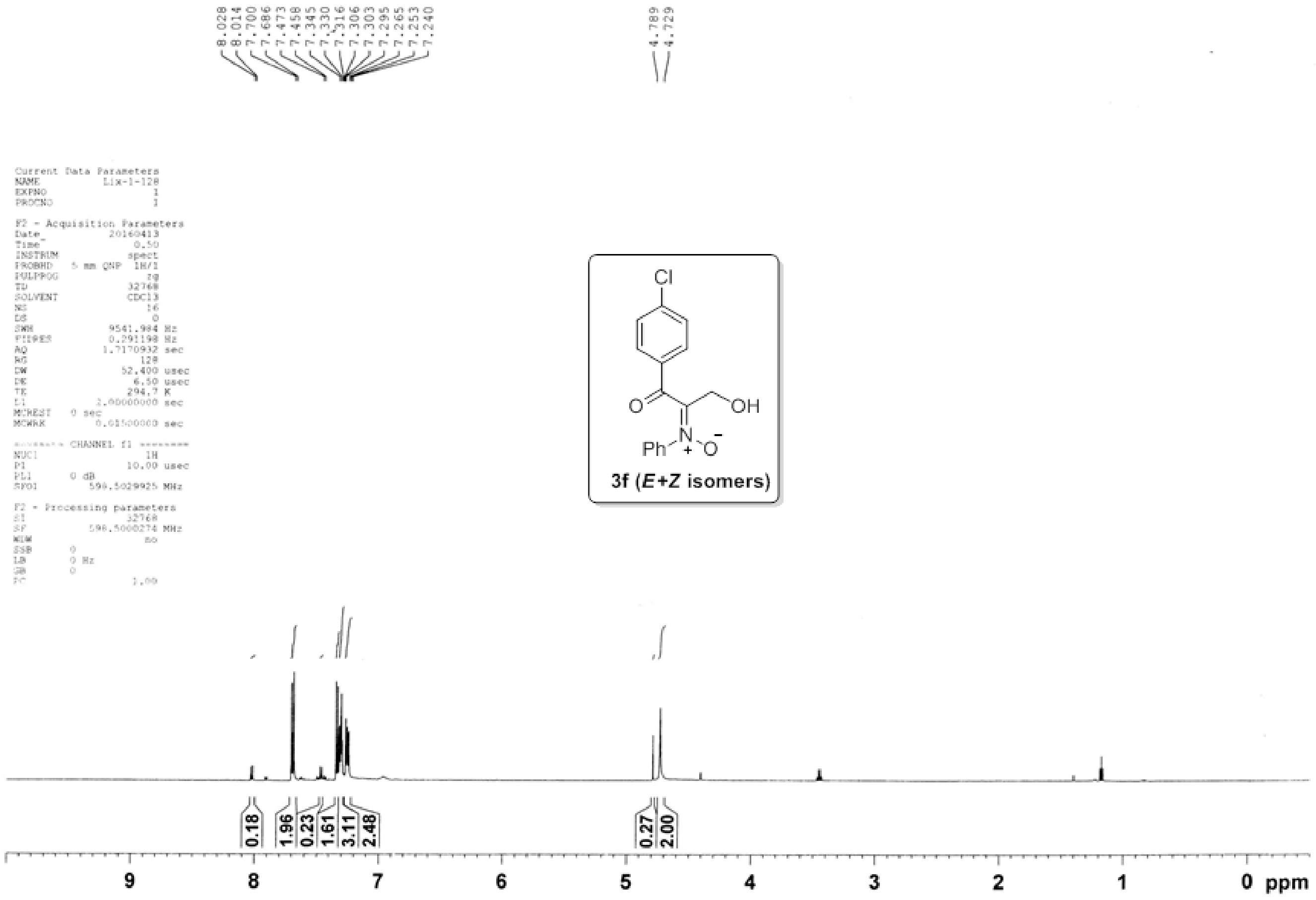
8.028
8.014
7.700
7.686
7.473
7.458
7.345
7.330
7.316
7.306
7.303
7.295
7.265
7.253
7.240
4.789
4.729
Current Data Parameters
NAME Lix-1-128
EXPNO 1
PROCNO 1
F2 - Acquisition Parameters
Date_ 20160413
Time 0.50
INSTRUM spect
PROBHD 5 mm QNP 1H/1
PULPROG zg
TD 32768
SOLVENT CDCl3
NS 16
DS 0
SWH 9541.984 Hz
FIDRES 0.291198 Hz
AQ 1.7170932 sec
RG 128
DW 52.400 usec
DE 6.50 usec
TE 294.7 K
D1 2.00000000 sec
MCREST 0 sec
MCWRK 0.01500000 sec
======== CHANNEL f1 ========
NUC1 1H
P1 10.00 usec
PL1 0 dB
SFO1 598.5029925 MHz
F2 - Processing parameters
SI 32768
SF 598.5000274 MHz
WDW no
SSB 0
LB 0 Hz
GB 0
PC 1.00
Cl
O
OH
N
Ph
+
O
–
3f (E+Z isomers)
0.18
1.96
0.23
1.61
3.11
2.48
0.27
2.00
9
8
7
6
5
4
3
2
1
0 ppm

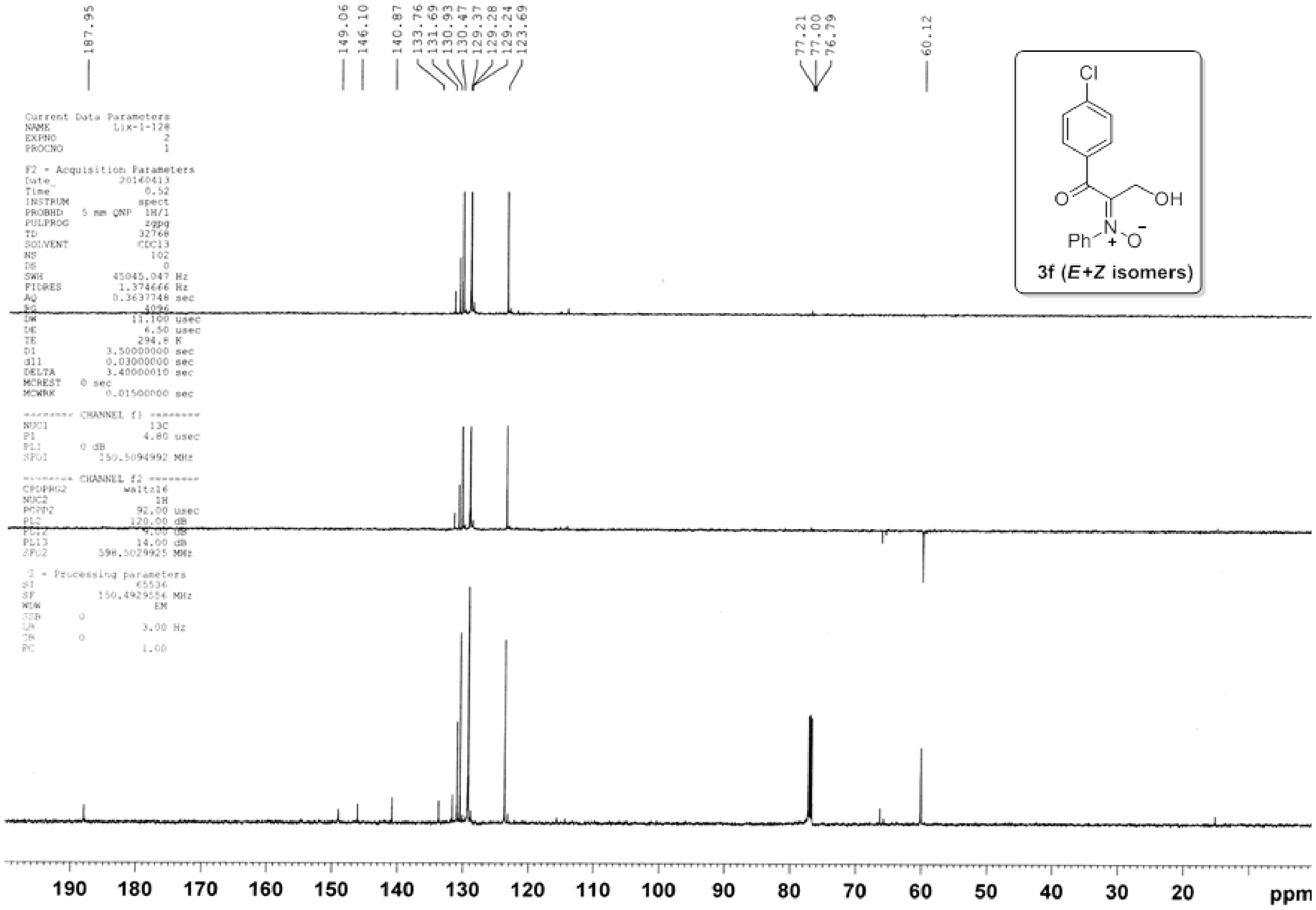
187.95
149.06
146.10
140.87
133.76
131.69
130.93
130.47
129.37
129.28
129.24
123.69
77.21
77.00
76.79
60.12
Current Data Parameters
NAME Lix-1-128
EXPNO 2
PROCNO 1
F2 - Acquisition Parameters
Date_ 20160413
Time 0.52
INSTRUM spect
PROBHD 5 mm QNP 1H/1
PULPROG zgpg
TD 32768
SOLVENT CDCl3
NS 102
DS 0
SWH 45045.047 Hz
FIDRES 1.374666 Hz
AQ 0.3637748 sec
RG 4096
DW 11.100 usec
DE 6.50 usec
TE 294.8 K
D1 3.50000000 sec
d11 0.03000000 sec
DELTA 3.40000010 sec
MCREST 0 sec
MCWRK 0.01500000 sec
======== CHANNEL f1 ========
NUC1 13C
P1 4.80 usec
PL1 0 dB
SFO1 150.5094992 MHz
======== CHANNEL f2 ========
CPDPRG2 waltz16
NUC2 1H
PCPD2 92.00 usec
PL2 120.00 dB
PL12 4.00 dB
PL13 14.00 dB
SFO2 598.5029925 MHz
F2 - Processing parameters
SI 65536
SF 150.4929556 MHz
WDW EM
SSB 0
LB 3.00 Hz
GB 0
PC 1.00
Cl
O
OH
N
Ph
O
3f (E+Z isomers)
190
180
170
160
150
140
130
120
110
100
90
80
70
60
50
40
30
20
ppm

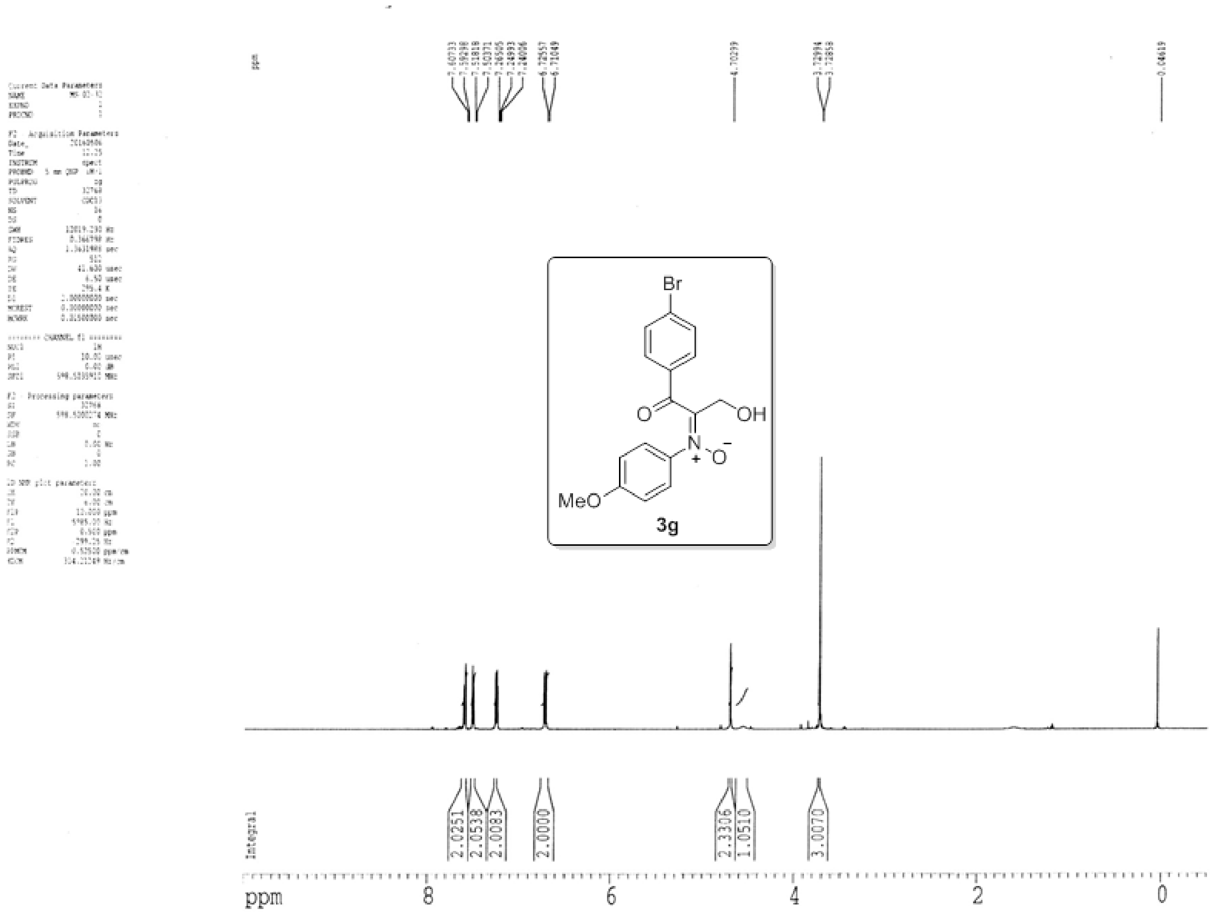
Br
O
OH
N
O
MeO
3g
Integral
2.0251
2.0538
2.0083
2.0000
2.3306
1.0510
3.0070
ppm
8
6
4
2
0

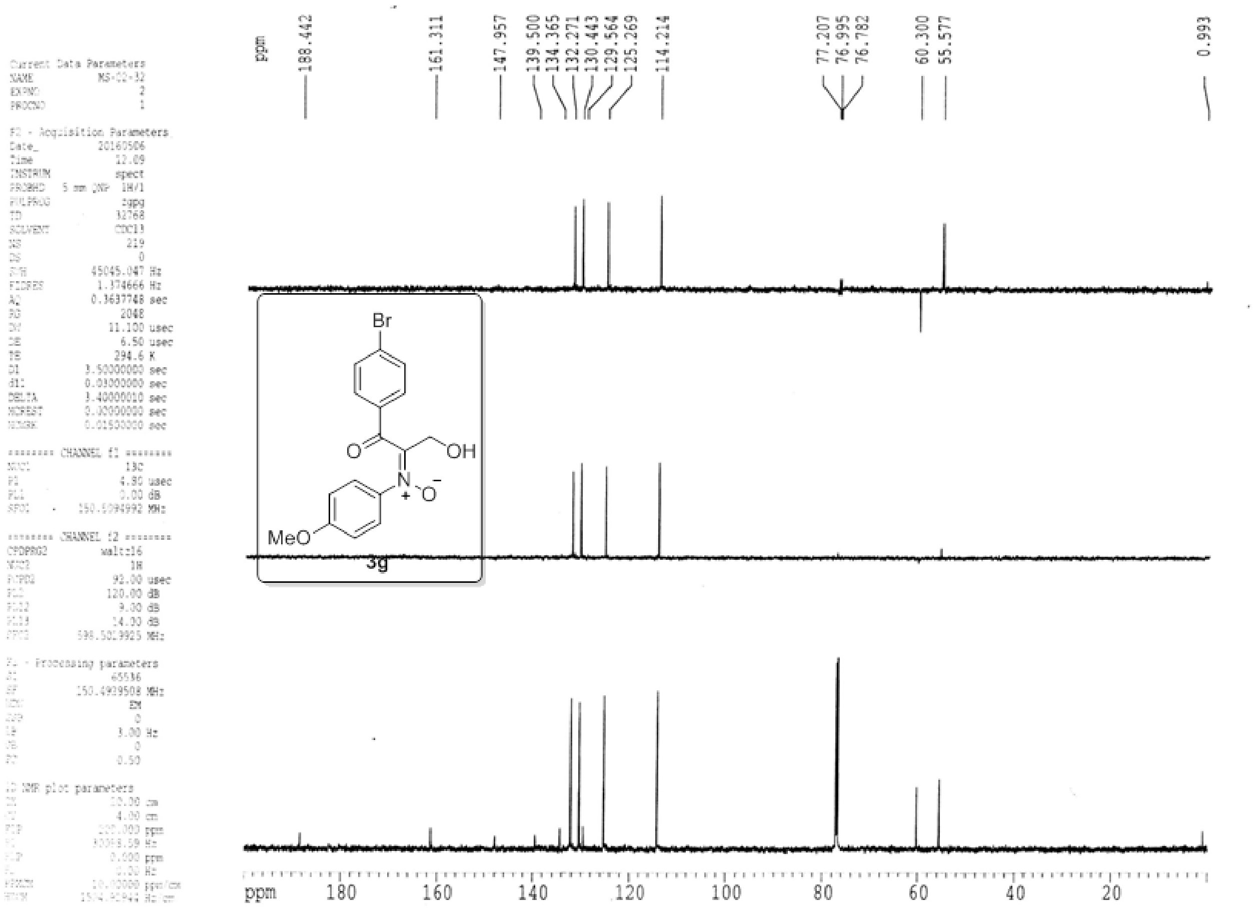
Current Data Parameters
NAME MS-02-32
EXPNO 2
PROCNO 1
F2 - Acquisition Parameters
Date_ 20160506
SOLVENT CDCl3
188.442
161.311
147.957
139.500
134.365
132.271
130.443
129.564
125.269
114.214
77.207
76.995
76.782
60.300
55.577
0.993
Br
O
OH
N
+
O
-
MeO
3g
ppm 180 160 140 120 100 80 60 40 20

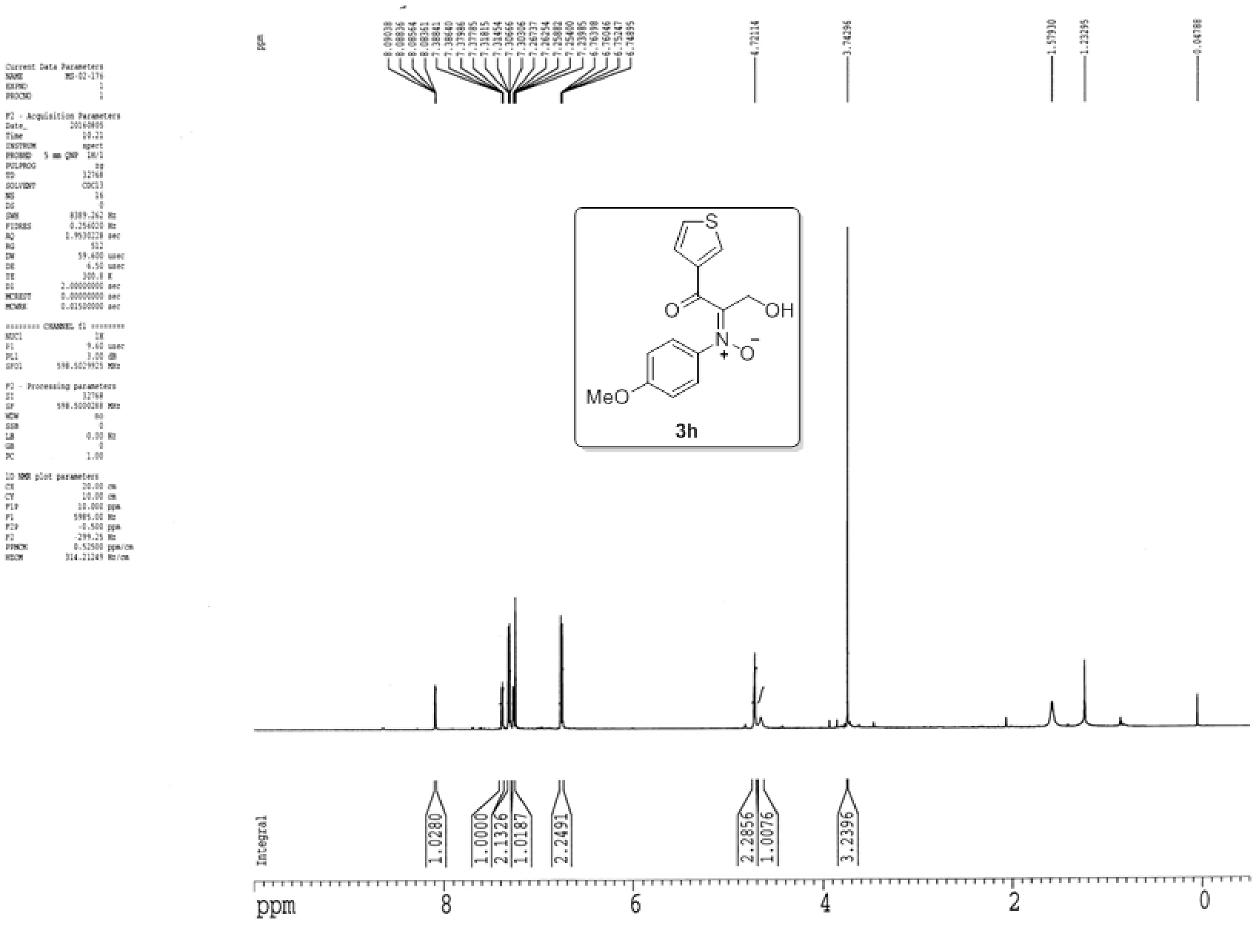

MeO
OH
3h
Integral
ppm
1.0280
1.0000
2.1326
1.0187
2.2491
2.2856
1.0076
3.2396

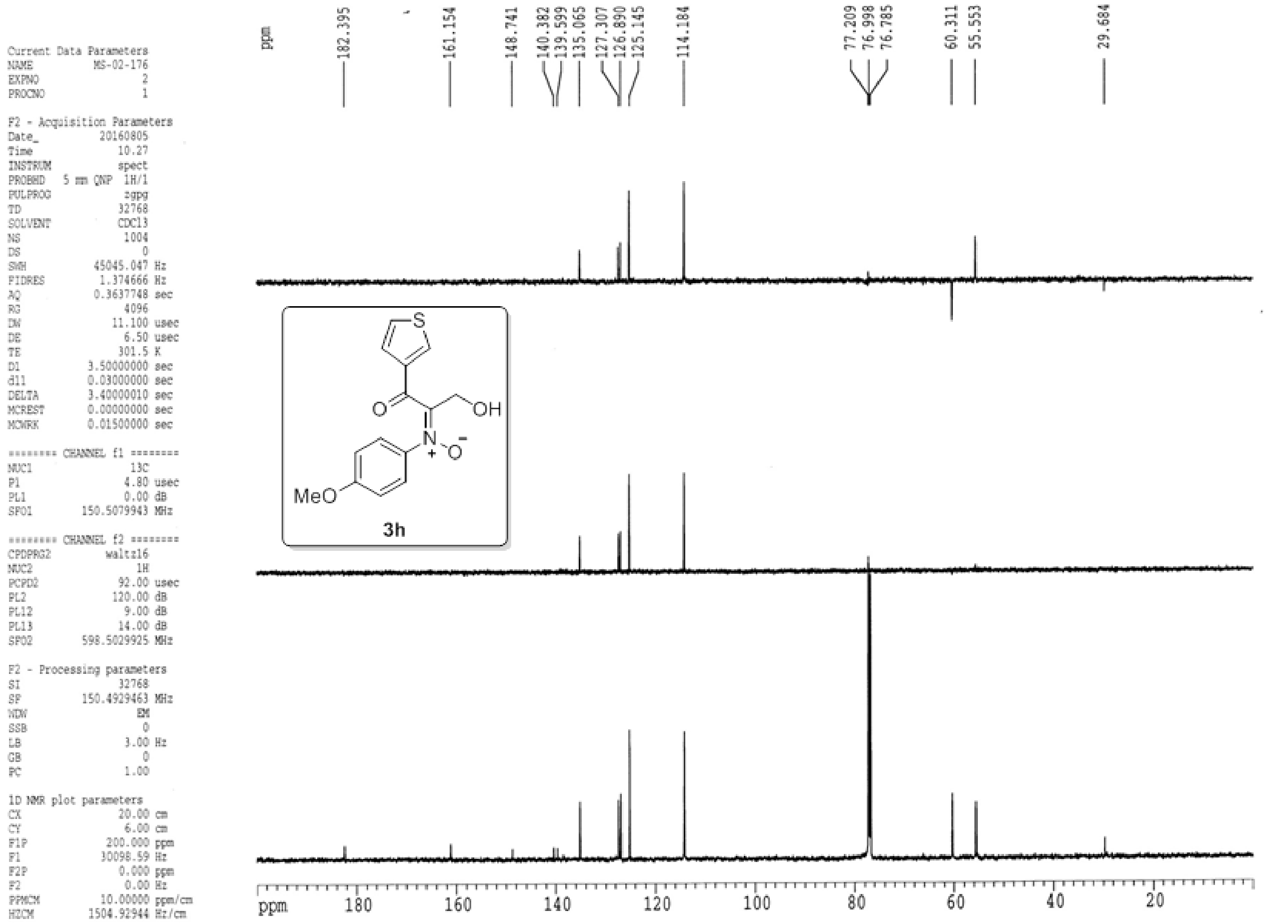
Current Data Parameters
NAME MS-02-176
EXPNO 2
PROCNO 1
F2 - Acquisition Parameters
Date_ 20160805
Time 10.27
INSTRUM spect
PROBHD 5 mm QNP 1H/1
PULPROG zgpg
TD 32768
SOLVENT CDCl3
NS 1004
DS 0
SWH 45045.047 Hz
FIDRES 1.374666 Hz
AQ 0.3637748 sec
RG 4096
DW 11.100 usec
DE 6.50 usec
TE 301.5 K
D1 3.50000000 sec
d11 0.03000000 sec
DELTA 3.40000010 sec
MCREST 0.00000000 sec
MCWRK 0.01500000 sec
======== CHANNEL f1 ========
NUC1 13C
P1 4.80 usec
PL1 0.00 dB
SFO1 150.5079943 MHz
======== CHANNEL f2 ========
CPDPRG2 waltz16
NUC2 1H
PCPD2 92.00 usec
PL2 120.00 dB
PL12 9.00 dB
PL13 14.00 dB
SFO2 598.5029925 MHz
F2 - Processing parameters
SI 32768
SF 150.4929463 MHz
WDW EM
SSB 0
LB 3.00 Hz
GB 0
PC 1.00
1D NMR plot parameters
CX 20.00 cm
CY 6.00 cm
F1P 200.000 ppm
F1 30098.59 Hz
F2P 0.000 ppm
F2 0.00 Hz
PPMCM 10.00000 ppm/cm
HZCM 1504.92944 Hz/cm
ppm
182.395
161.154
148.741
140.382
139.599
135.065
127.307
126.890
125.145
114.184
77.209
76.998
76.785
60.311
55.553
29.684
S
O
OH
N
+
O
-
MeO
3h
ppm 180 160 140 120 100 80 60 40 20

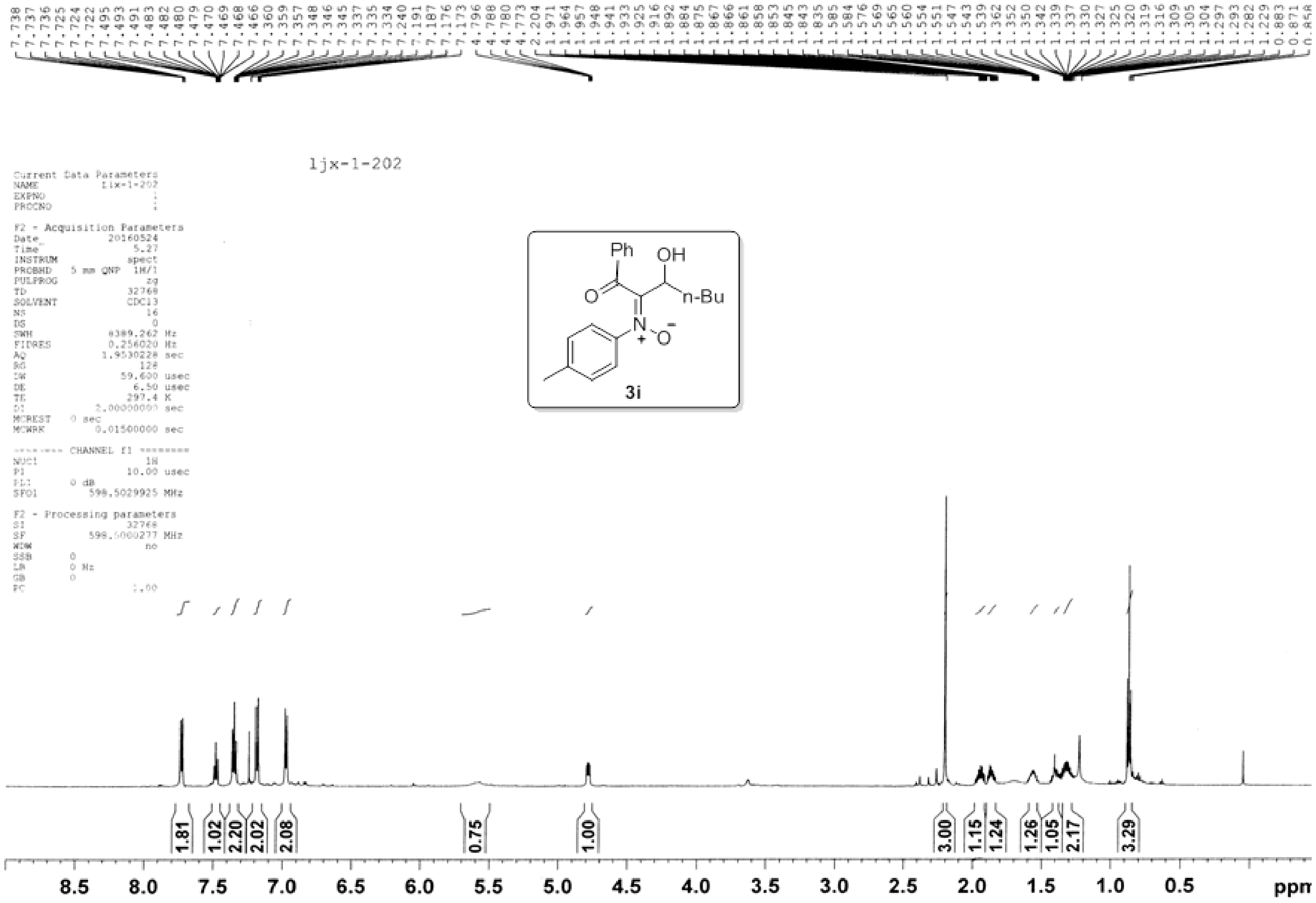

ljx-1-202
Ph
OH
O
n-Bu
N
O
3i
ppm

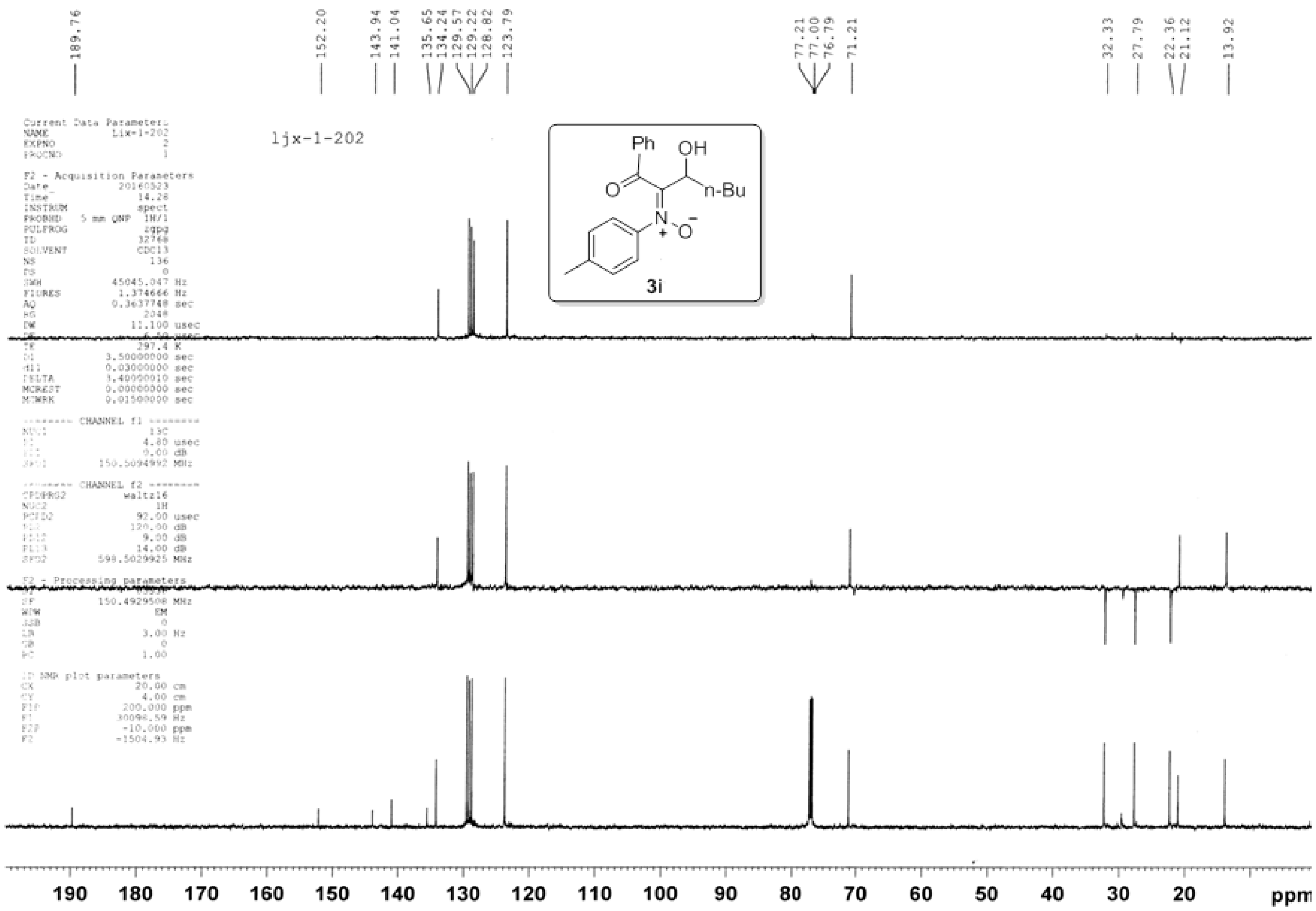
189.76
152.20
143.94
141.04
135.65
134.24
129.57
129.22
128.82
123.79
77.21
77.00
76.79
71.21
32.33
27.79
22.36
21.12
13.92
ljx-1-202
Ph
OH
O
n-Bu
N
O
+
3i
190 180 170 160 150 140 130 120 110 100 90 80 70 60 50 40 30 20 ppm

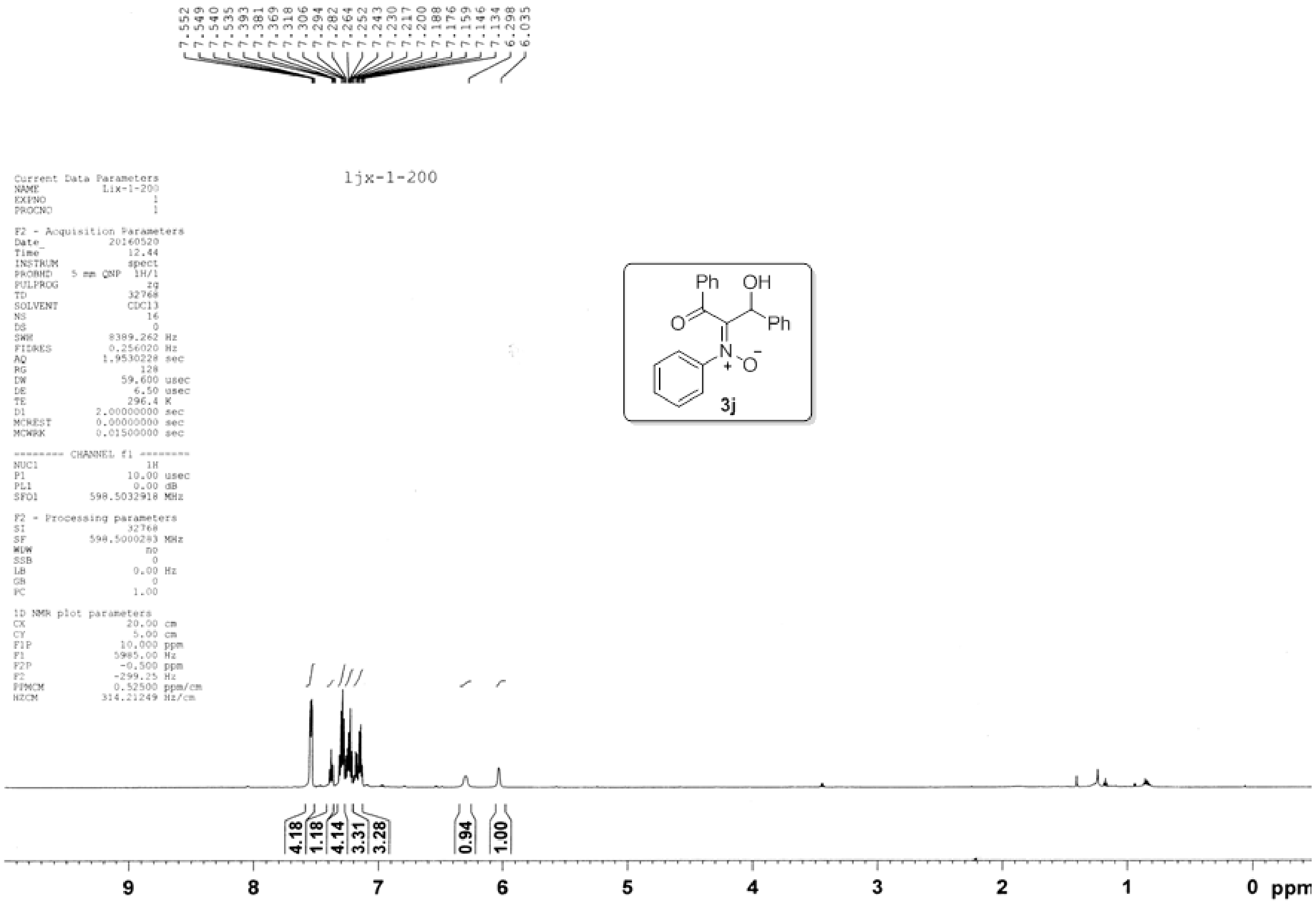
7.552
7.549
7.540
7.535
7.393
7.381
7.369
7.318
7.306
7.294
7.282
7.264
7.252
7.243
7.230
7.217
7.200
7.188
7.176
7.159
7.146
7.134
6.298
6.035
Current Data Parameters
NAME Ljx-1-200
EXPNO 1
PROCNO 1
F2 - Acquisition Parameters
Date_ 20160520
Time 12.44
INSTRUM spect
PROBHD 5 mm QNP 1H/1
PULPROG zg
TD 32768
SOLVENT CDCl3
NS 16
DS 0
SWH 8389.262 Hz
FIDRES 0.256020 Hz
AQ 1.9530228 sec
RG 128
DW 59.600 usec
DE 6.50 usec
TE 296.4 K
D1 2.00000000 sec
MCREST 0.00000000 sec
MCWRK 0.01500000 sec
======== CHANNEL f1 ========
NUC1 1H
P1 10.00 usec
PL1 0.00 dB
SFO1 598.5032918 MHz
F2 - Processing parameters
SI 32768
SF 598.5000283 MHz
WDW no
SSB 0
LB 0.00 Hz
GB 0
PC 1.00
1D NMR plot parameters
CX 20.00 cm
CY 5.00 cm
F1P 10.000 ppm
F1 5985.00 Hz
F2P -0.500 ppm
F2 -299.25 Hz
PPMCM 0.52500 ppm/cm
HZCM 314.21249 Hz/cm
1jx-1-200
Ph
OH
O
Ph
N
+
O
-
3j
4.18
1.18
4.14
3.31
3.28
0.94
1.00
9
8
7
6
5
4
3
2
1
0 ppm

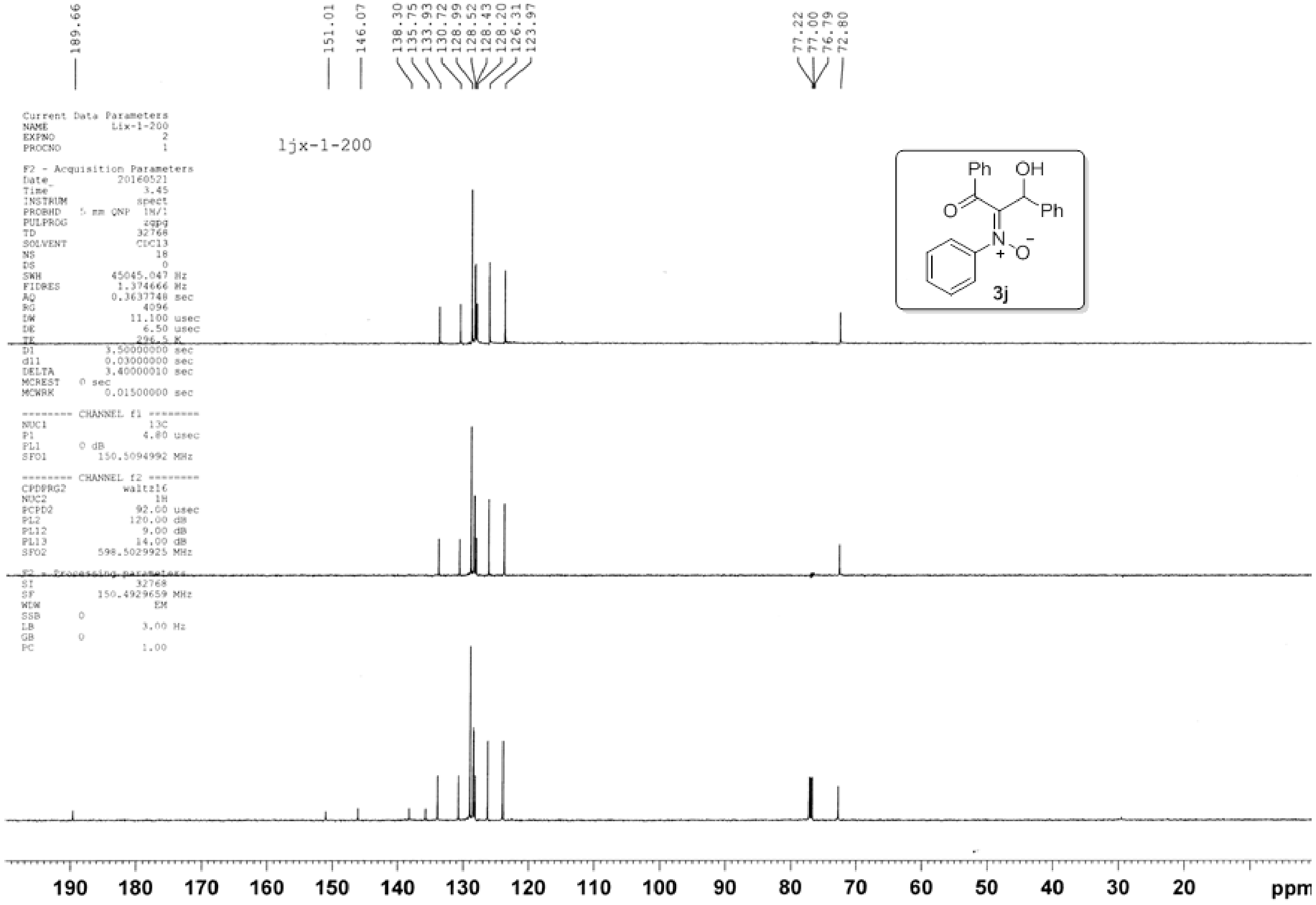
189.66
151.01
146.07
138.30
135.75
133.93
130.72
128.99
128.52
128.43
128.20
126.31
123.97
77.22
77.00
76.79
72.80
Current Data Parameters
NAME Lix-1-200
EXPNO 2
PROCNO 1
F2 - Acquisition Parameters
Date_ 20160521
Time 3.45
INSTRUM spect
PROBHD 5 mm QNP 1H/1
PULPROG zgpg
TD 32768
SOLVENT CDCl3
NS 18
DS 0
SWH 45045.047 Hz
FIDRES 1.374666 Hz
AQ 0.3637748 sec
RG 4096
DW 11.100 usec
DE 6.50 usec
TE 296.5 K
D1 3.50000000 sec
d11 0.03000000 sec
DELTA 3.40000010 sec
MCREST 0 sec
MCWRK 0.01500000 sec
======== CHANNEL f1 ========
NUC1 13C
P1 4.80 usec
PL1 0 dB
SFO1 150.5094992 MHz
======== CHANNEL f2 ========
CPDPRG2 waltz16
NUC2 1H
PCPD2 92.00 usec
PL2 120.00 dB
PL12 9.00 dB
PL13 14.00 dB
SFO2 598.5029925 MHz
F2 - Processing parameters
SI 32768
SF 150.4929659 MHz
WDW EM
SSB 0
LB 3.00 Hz
GB 0
PC 1.00
ljx-1-200
Ph
OH
O
Ph
N
+
O
-
3j
190
180
170
160
150
140
130
120
110
100
90
80
70
60
50
40
30
20
ppm

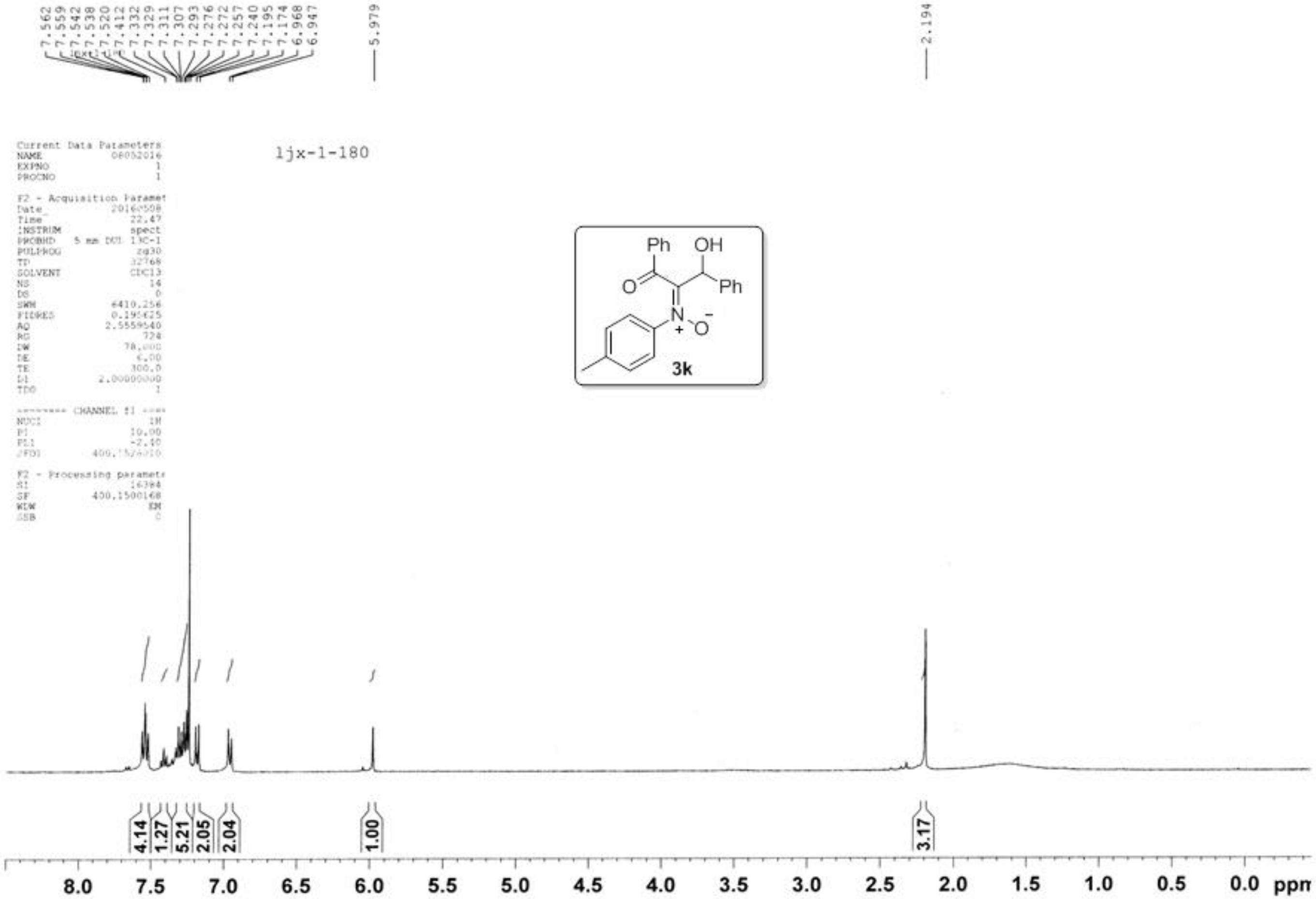

ljx-1-180
Ph
OH
O
Ph
N
+
O
-
3k
8.0
7.5
7.0
6.5
6.0
5.5
5.0
4.5
4.0
3.5
3.0
2.5
2.0
1.5
1.0
0.5
0.0 ppm
4.14
1.27
5.21
2.05
2.04
1.00
3.17
5.979
2.194

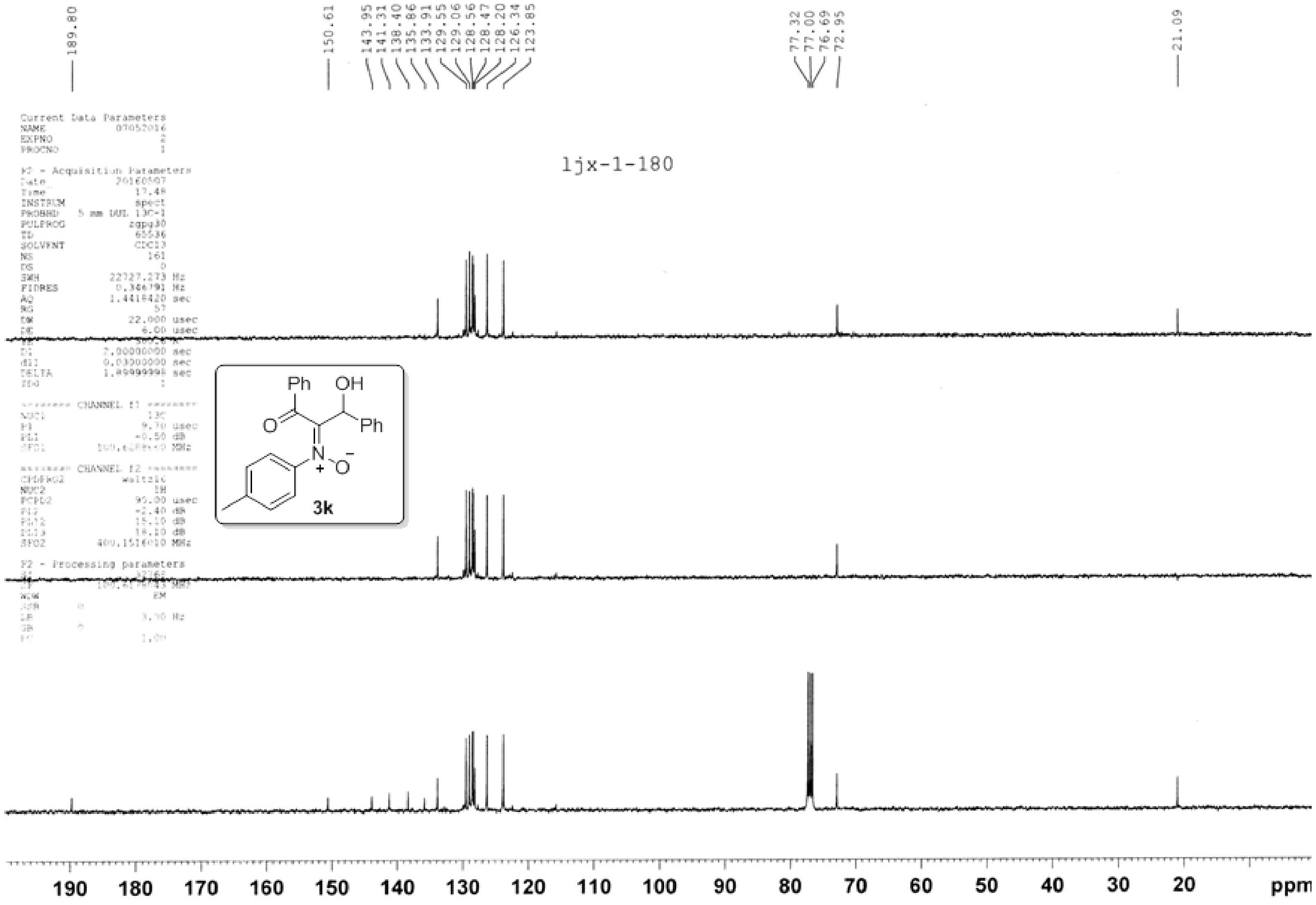
189.80
150.61
143.95
141.31
138.40
135.86
133.91
129.55
129.06
128.56
128.47
128.20
126.34
123.85
77.32
77.00
76.69
72.95
21.09
ljx-1-180
3k
ppm

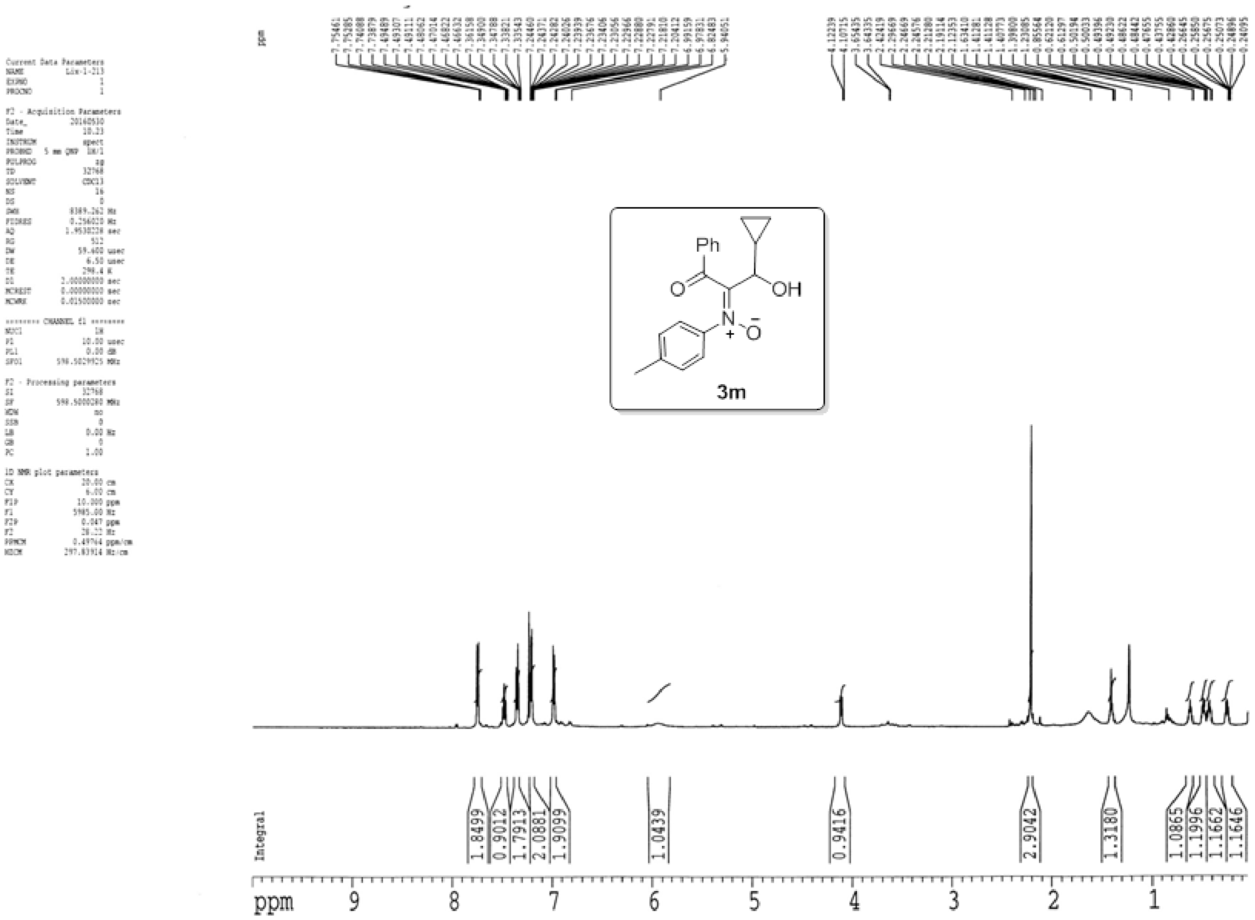
Ph
OH
3m
Integral
ppm

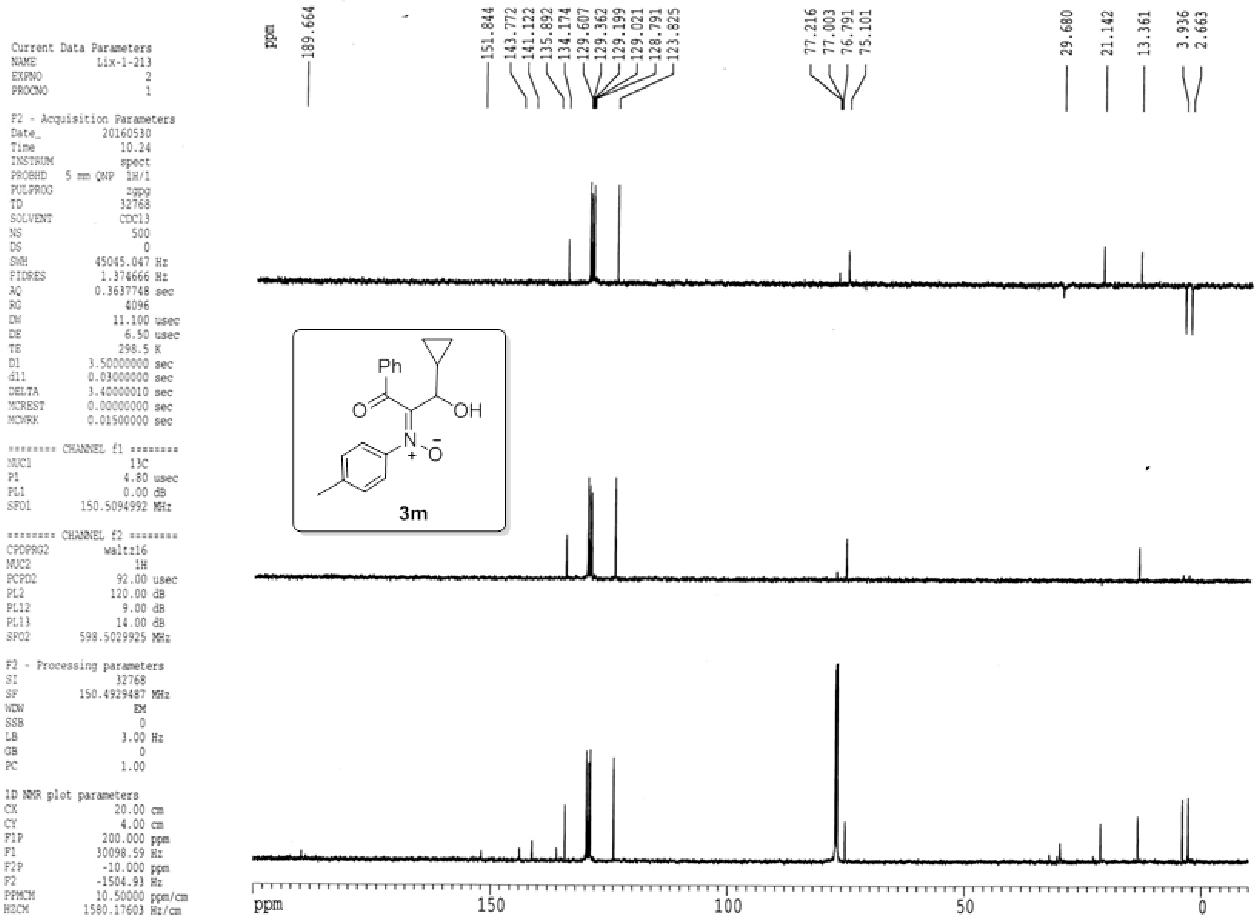
Current Data Parameters
NAME Lix-1-213
EXPNO 2
PROCNO 1
F2 - Acquisition Parameters
Date_ 20160530
Time 10.24
INSTRUM spect
PROBHD 5 mm QNP 1H/1
PULPROG zgpg
TD 32768
SOLVENT CDCl3
NS 500
DS 0
SWH 45045.047 Hz
FIDRES 1.374666 Hz
AQ 0.3637748 sec
RG 4096
DW 11.100 usec
DE 6.50 usec
TE 298.5 K
D1 3.50000000 sec
d11 0.03000000 sec
DELTA 3.40000010 sec
MCREST 0.00000000 sec
MCWRK 0.01500000 sec
======== CHANNEL f1 ========
NUC1 13C
P1 4.80 usec
PL1 0.00 dB
SFO1 150.5094992 MHz
======== CHANNEL f2 ========
CPDPRG2 waltz16
NUC2 1H
PCPD2 92.00 usec
PL2 120.00 dB
PL12 9.00 dB
PL13 14.00 dB
SFO2 598.5029925 MHz
F2 - Processing parameters
SI 32768
SF 150.4929487 MHz
WDW EM
SSB 0
LB 3.00 Hz
GB 0
PC 1.00
1D NMR plot parameters
CX 20.00 cm
CY 4.00 cm
F1P 200.000 ppm
F1 30098.59 Hz
F2P -10.000 ppm
F2 -1504.93 Hz
PPMCM 10.50000 ppm/cm
HZCM 1580.17603 Hz/cm
ppm
189.664
151.844
143.772
141.122
135.892
134.174
129.607
129.362
129.199
129.021
128.791
123.825
77.216
77.003
76.791
75.101
29.680
21.142
13.361
3.936
2.663
Ph
O
OH
N
+
O
-
3m
ppm
150
100
50
0

Current Data Parameters
NAME Lix-1-164
EXPNO 1
PROCNO 1

F2 - Acquisition Parameters
Date_ 20160509
Time 7.57
INSTRUM spect
PROBHD 5 mm QNP 1H/1
PULPROG zg
TD 32768
SOLVENT CDCl3
NS 16
DS 0
SWH 9541.984 Hz
FIDRES 0.291198 Hz
AQ 1.7170932 sec
RG 512
DW 52.400 usec
DE 6.50 usec
TE 296.6 K
D1 1.50000000 sec
MCREST 0.00000000 sec
MCWRK 0.01500000 sec

======== CHANNEL f1 ========
NUC1 1H
P1 8.50 usec
PL1 3.00 dB
SFO1 598.5028429 MHz

F2 - Processing parameters
SI 32768
SF 598.5000275 MHz
WDW no
SSB 0
LB 0.00 Hz
GB 0
PC 1.00

1D NMR plot parameters
CX 20.00 cm
CY 10.00 cm
F1P 10.000 ppm
F1 5985.00 Hz
F2P -0.500 ppm
F2 -299.25 Hz
PPMCM 0.52500 ppm/cm
HZCM 314.21249 Hz/cm

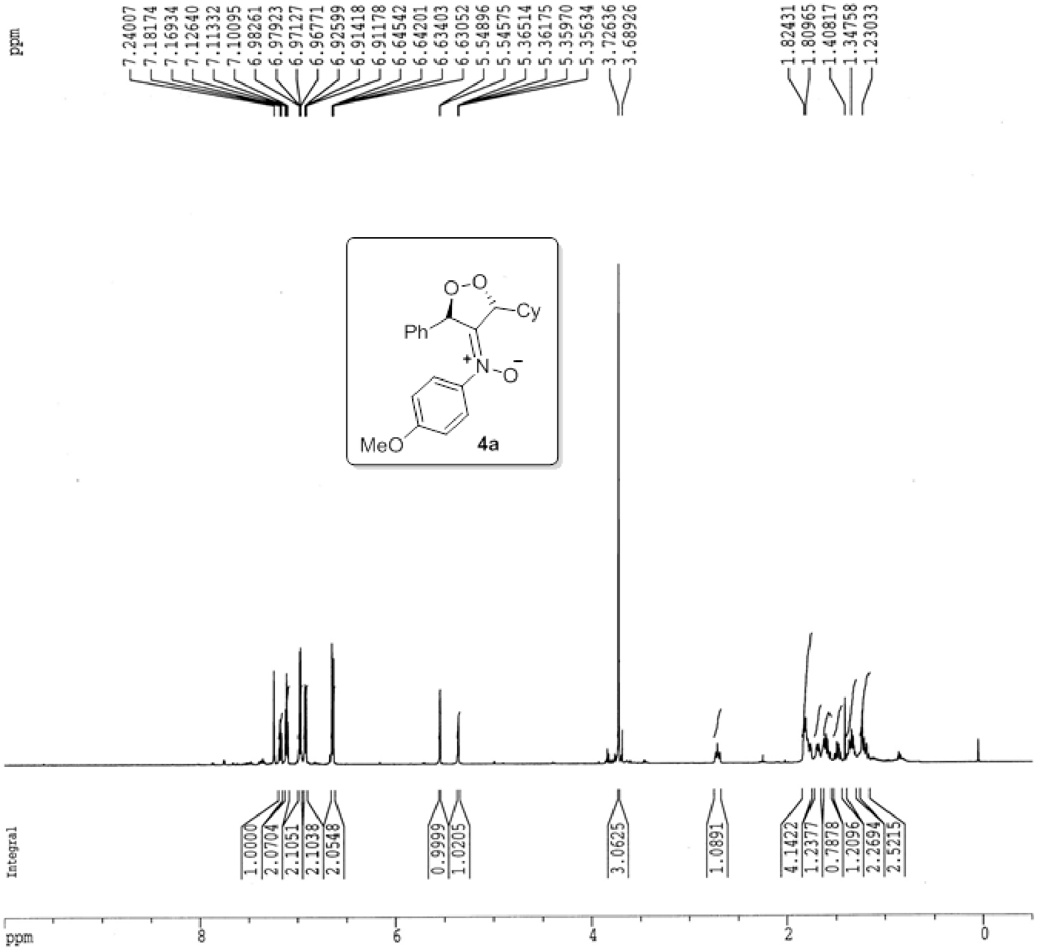

```
Current Data Parameters
NAME             Lix-1-164
EXPNO                    2
PROCNO                   1

F2 - Acquisition Parameters
Date_             20160509
Time                  8.12
INSTRUM              spect
PROBHD   5 mm QNP  1H/1
PULPROG               zgpg
TD                   32768
SOLVENT              CDCl3
NS                     258
DS                       0
SWH             45045.047 Hz
FIDRES           1.374666 Hz
AQ              0.3637748 sec
RG                    4096
DW                  11.100 usec
DE                    6.50 usec
TE                   297.6 K
D1              3.50000000 sec
d11             0.03000000 sec
DELTA           3.40000010 sec
MCREST          0.00000000 sec
MCWRK           0.01500000 sec

======== CHANNEL f1 ========
NUC1                   13C
P1                    4.80 usec
PL1                   0.00 dB
SFO1           150.5094992 MHz

======== CHANNEL f2 ========
CPDPRG2            waltz16
NUC2                    1H
PCPD2                92.00 usec
PL2                 120.00 dB
PL12                  9.00 dB
PL13                 14.00 dB
SFO2           598.5029925 MHz

F2 - Processing parameters
SI                   32768
SF             150.4929487 MHz
WDW                     EM
SSB                      0
LB                    3.00 Hz
GB                       0
PC                    1.00

1D NMR plot parameters
CX                   20.00 cm
CY                    4.00 cm
F1P                200.000 ppm
F1                30098.59 Hz
F2P                  0.000 ppm
F2                    0.00 Hz
PPMCM             10.00000 ppm/cm
HZCM            1504.92944 Hz/cm
```

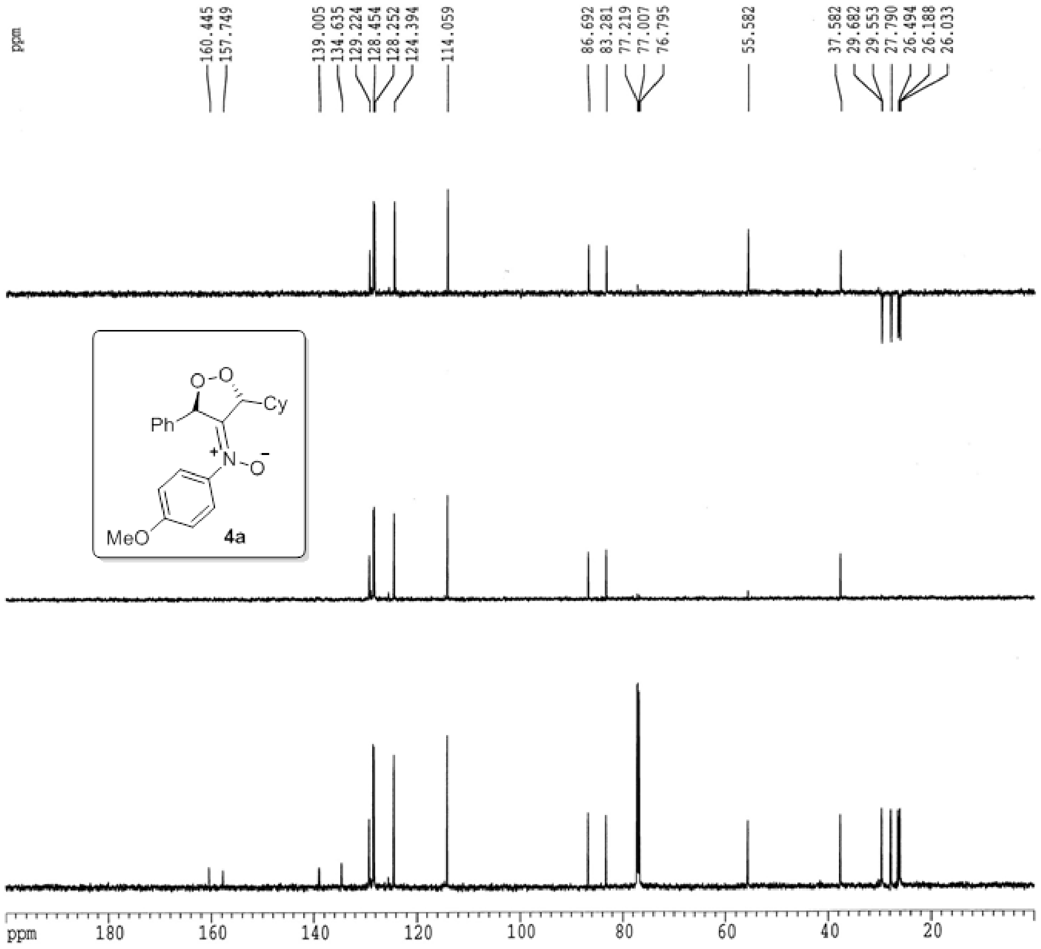

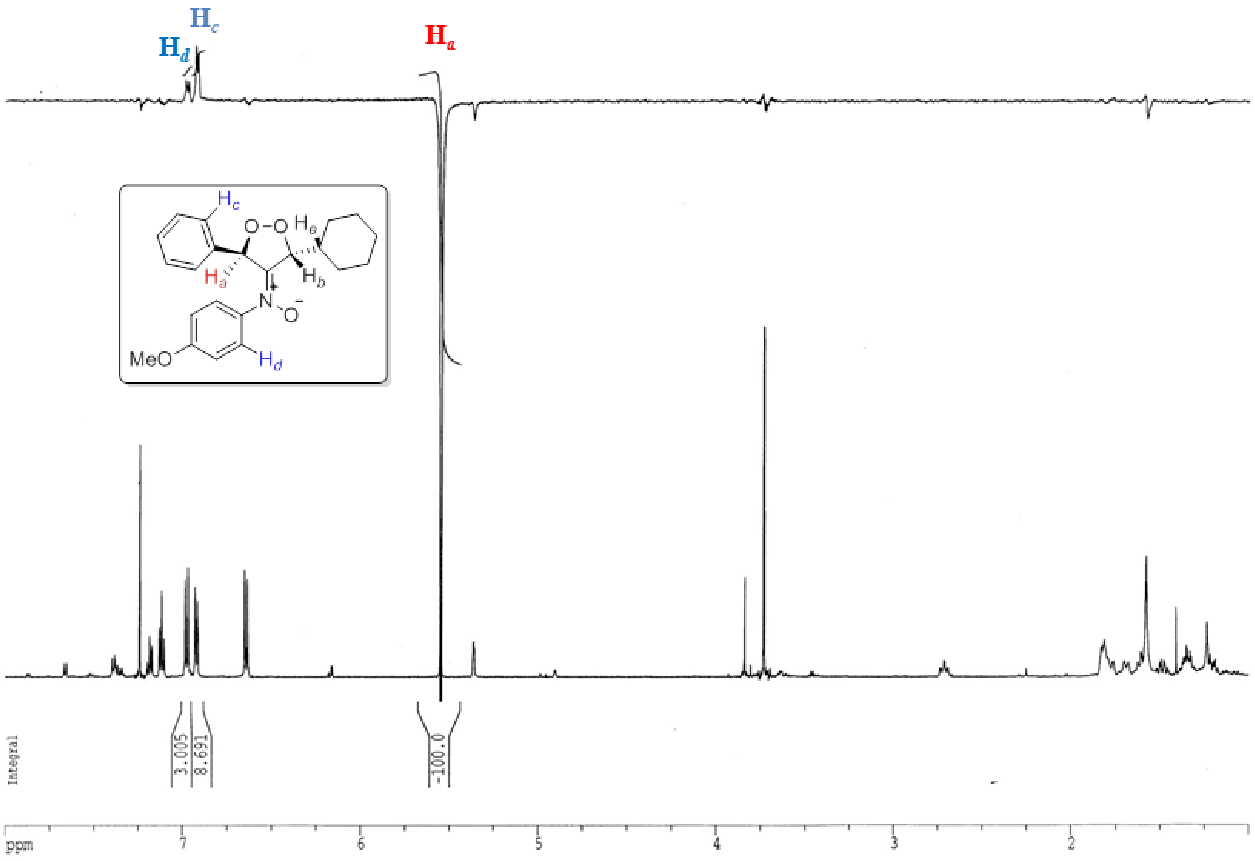
H$_c$
H$_d$
H$_a$
H$_c$
O–O
H$_e$
H$_a$
H$_b$
N
MeO
H$_d$
Integral
3.005
8.691
-100.0
ppm
7
6
5
4
3
2

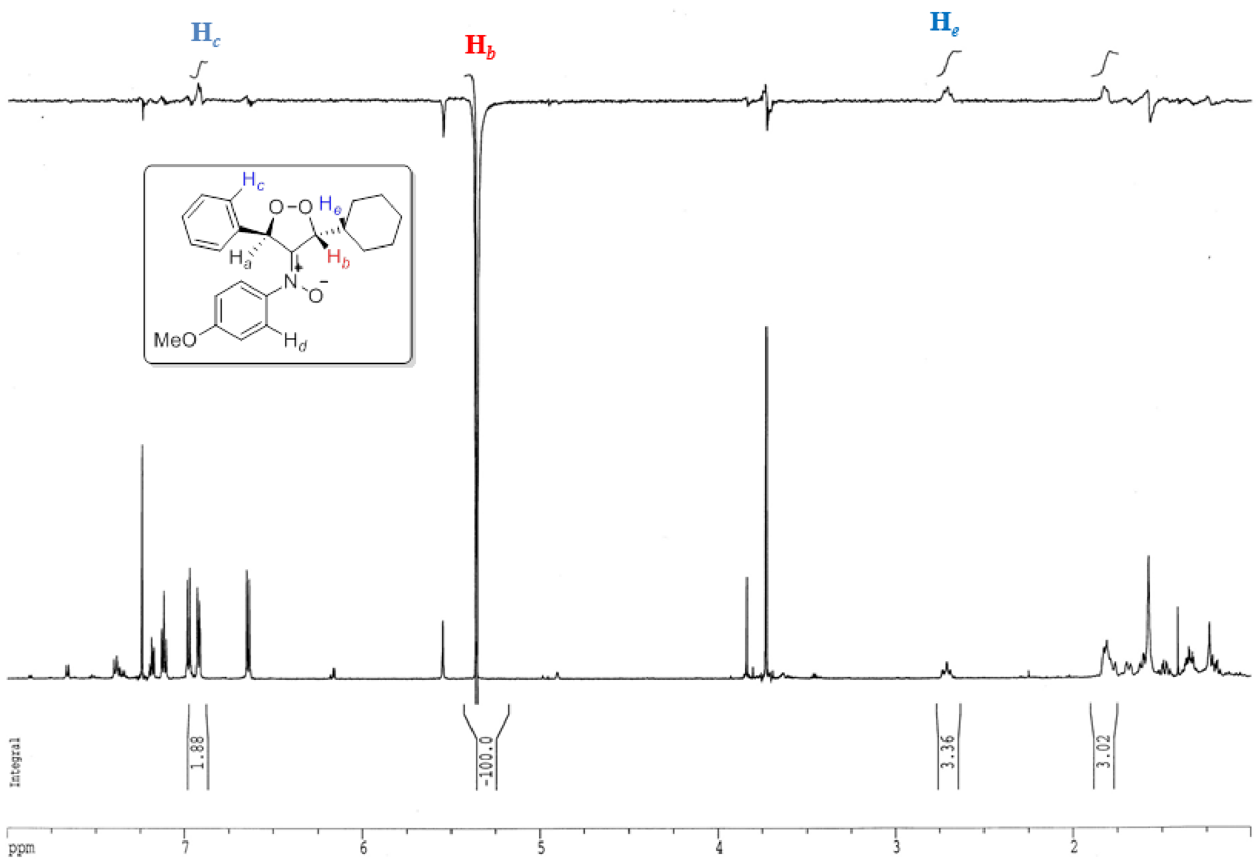

H_c
H_b
H_e
MeO
Integral
1.88
-100.0
3.36
3.02
ppm
7
6
5
4
3
2

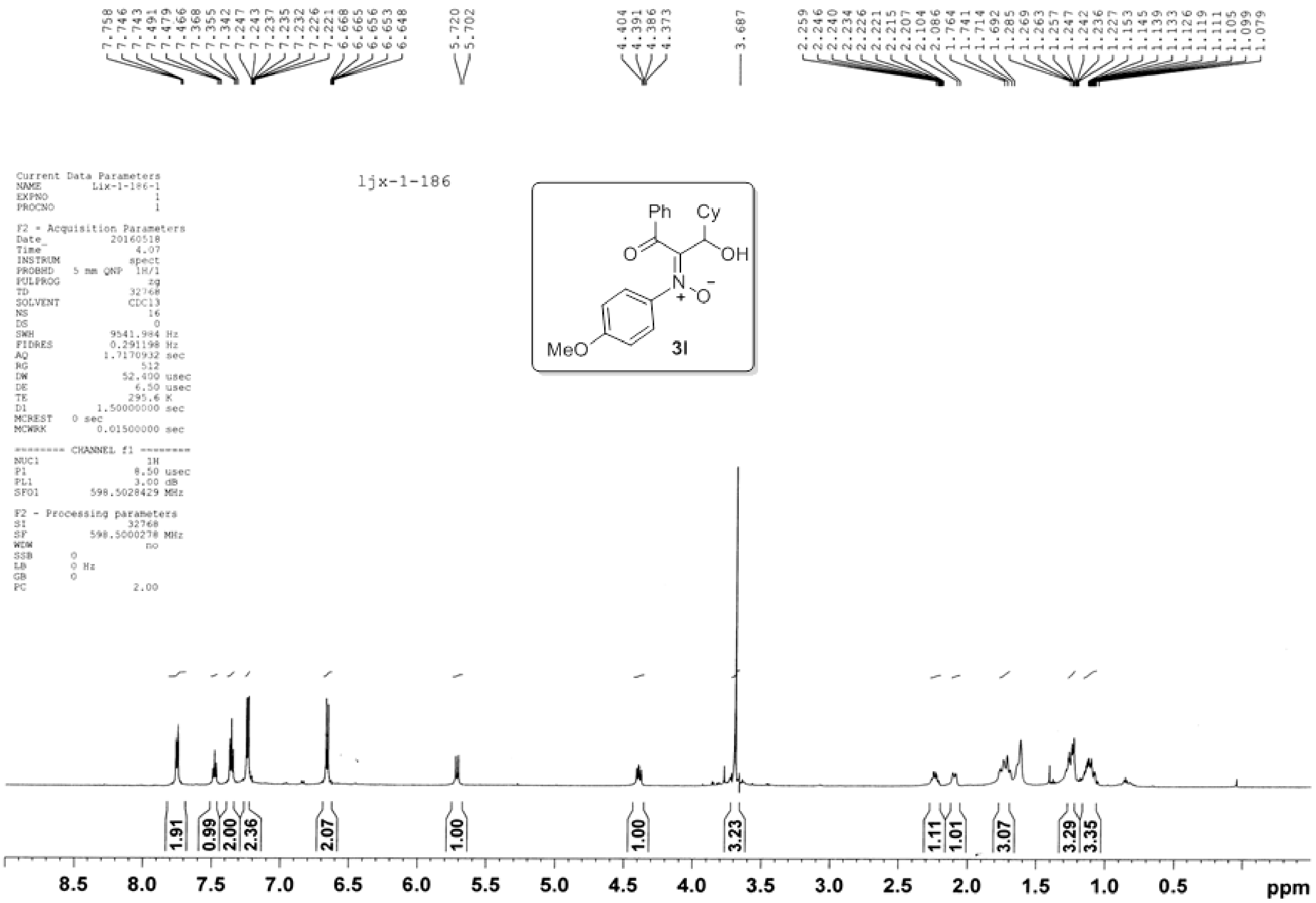
ljx-1-186
Ph
Cy
O
OH
N
O
MeO
3l
ppm

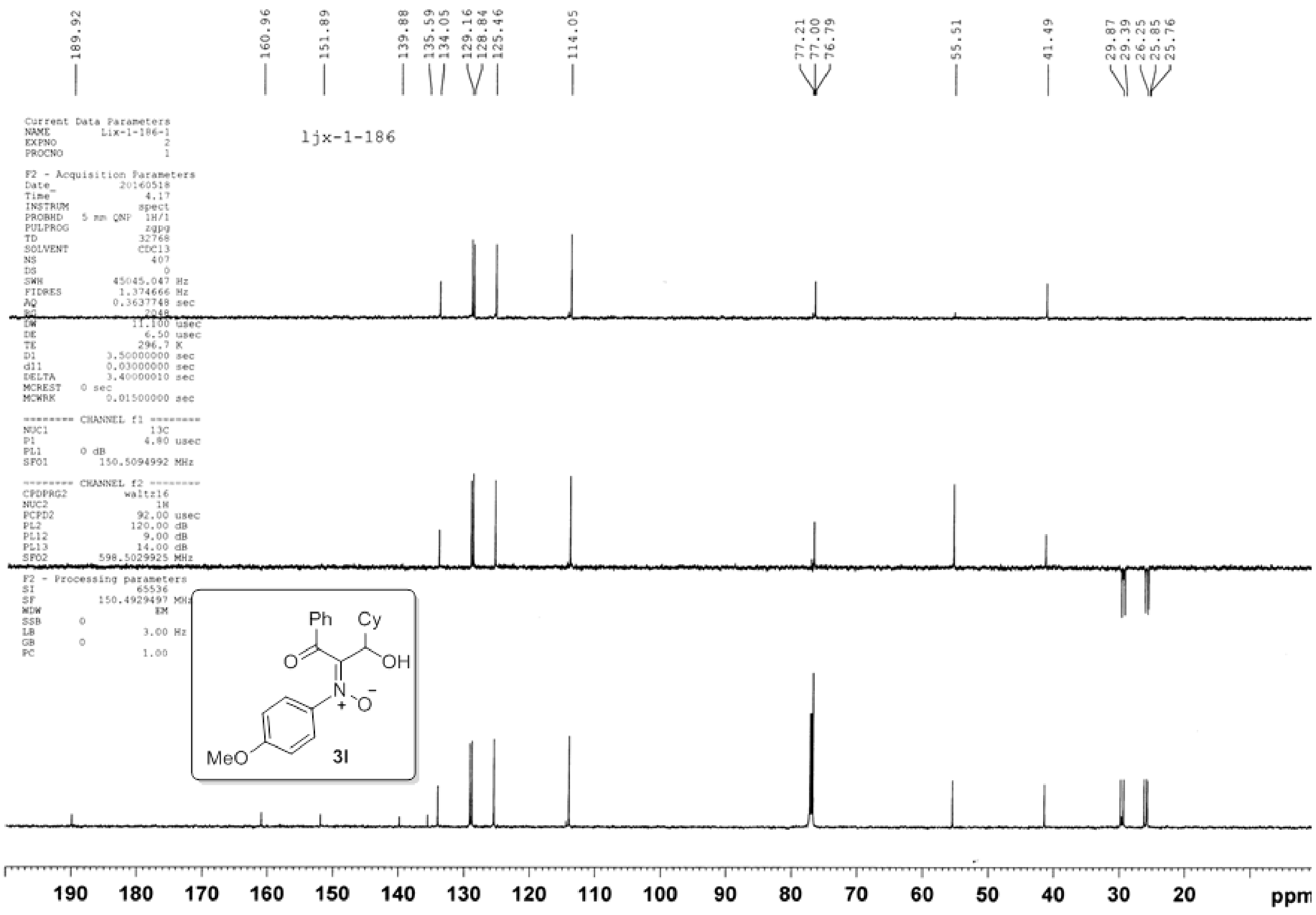
189.92
160.96
151.89
139.88
135.59
134.05
129.16
128.84
125.46
114.05
77.21
77.00
76.79
55.51
41.49
29.87
29.39
26.25
25.85
25.76
ljx-1-186
Current Data Parameters
NAME Lix-1-186-1
EXPNO 2
PROCNO 1
F2 - Acquisition Parameters
Date_ 20160518
Time 4.17
INSTRUM spect
PROBHD 5 mm QNP 1H/1
PULPROG zgpg
TD 32768
SOLVENT CDCl3
NS 407
DS 0
SWH 45045.047 Hz
FIDRES 1.374666 Hz
AQ 0.3637748 sec
RG 2048
DW 11.100 usec
DE 6.50 usec
TE 296.7 K
D1 3.50000000 sec
d11 0.03000000 sec
DELTA 3.40000010 sec
MCREST 0 sec
MCWRK 0.01500000 sec
======== CHANNEL f1 ========
NUC1 13C
P1 4.80 usec
PL1 0 dB
SFO1 150.5094992 MHz
======== CHANNEL f2 ========
CPDPRG2 waltz16
NUC2 1H
PCPD2 92.00 usec
PL2 120.00 dB
PL12 9.00 dB
PL13 14.00 dB
SFO2 598.5029925 MHz
F2 - Processing parameters
SI 65536
SF 150.4929497 MHz
WDW EM
SSB 0
LB 3.00 Hz
GB 0
PC 1.00
Ph
Cy
O
OH
N
O
MeO
3l
190
180
170
160
150
140
130
120
110
100
90
80
70
60
50
40
30
20
ppm

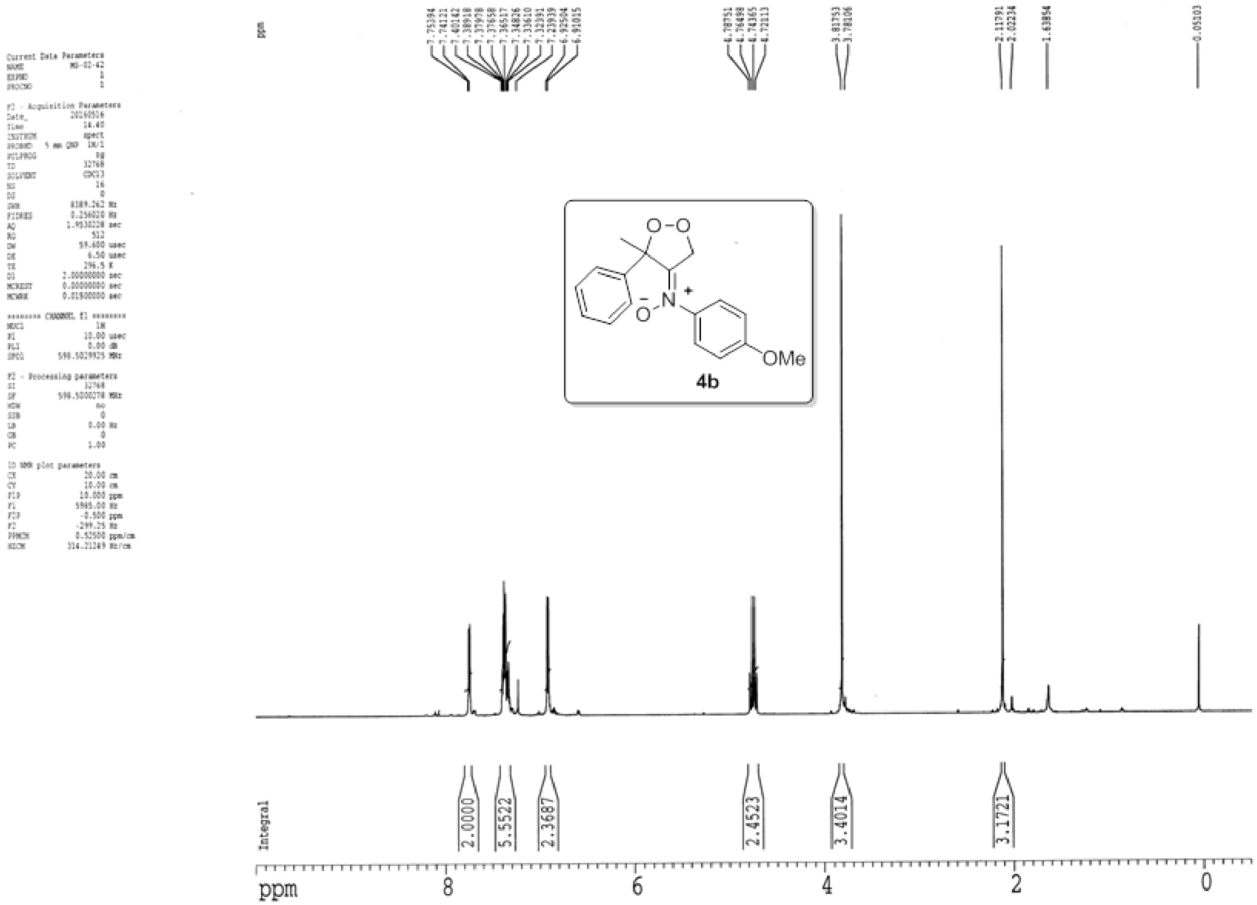

4b
OMe
Integral
2.0000
5.5522
2.3687
2.4523
3.4014
3.1721
ppm
8
6
4
2
0

Current Data Parameters
NAME MS-02-42
EXPNO 2
PROCNO 1

F2 - Acquisition Parameters
Date_ 20160516
Time 14.43
INSTRUM spect
PROBHD 5 mm QNP 1H/1
PULPROG zgpg
TD 32768
SOLVENT CDCl3
NS 169
DS 0
SWH 45045.047 Hz
FIDRES 1.374666 Hz
AQ 0.3637748 sec
RG 2048
DW 11.100 usec
DE 6.50 usec
TE 297.3 K
D1 3.50000000 sec
d11 0.03000000 sec
DELTA 3.40000010 sec
MCREST 0.00000000 sec
MCWRK 0.01500000 sec

======== CHANNEL f1 ========
NUC1 13C
P1 4.80 usec
PL1 0.00 dB
SFO1 150.5094992 MHz

======== CHANNEL f2 ========
CPDPRG2 waltz16
NUC2 1H
PCPD2 92.00 usec
PL2 120.00 dB
PL12 9.00 dB
PL13 14.00 dB
SFO2 598.5029925 MHz

F2 - Processing parameters
SI 65536
SF 150.4929522 MHz
WDW EM
SSB 0
LB 3.00 Hz
GB 0
PC 1.00

1D NMR plot parameters
CX 20.00 cm
CY 4.00 cm
F1P 200.000 ppm
F1 30098.59 Hz
F2P -10.000 ppm
F2 -1504.93 Hz
PPMCM 10.50000 ppm/cm
HZCM 1580.17603 Hz/cm

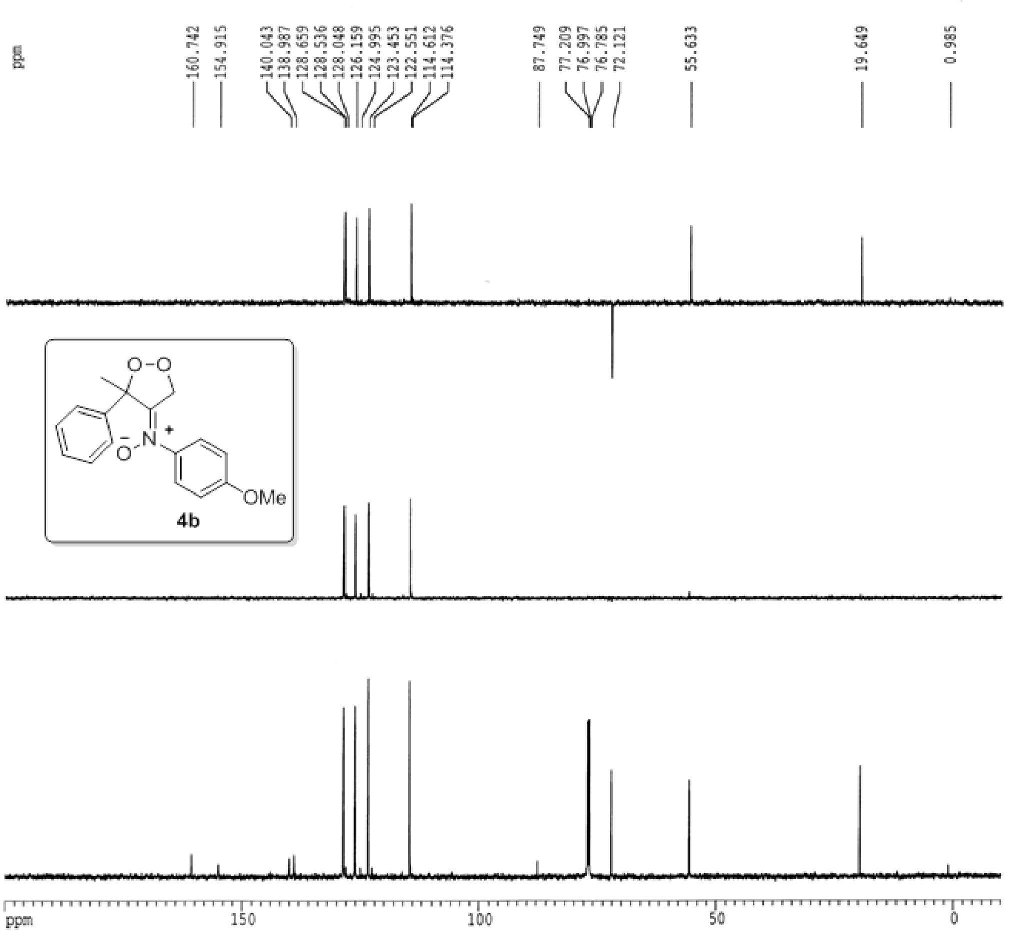

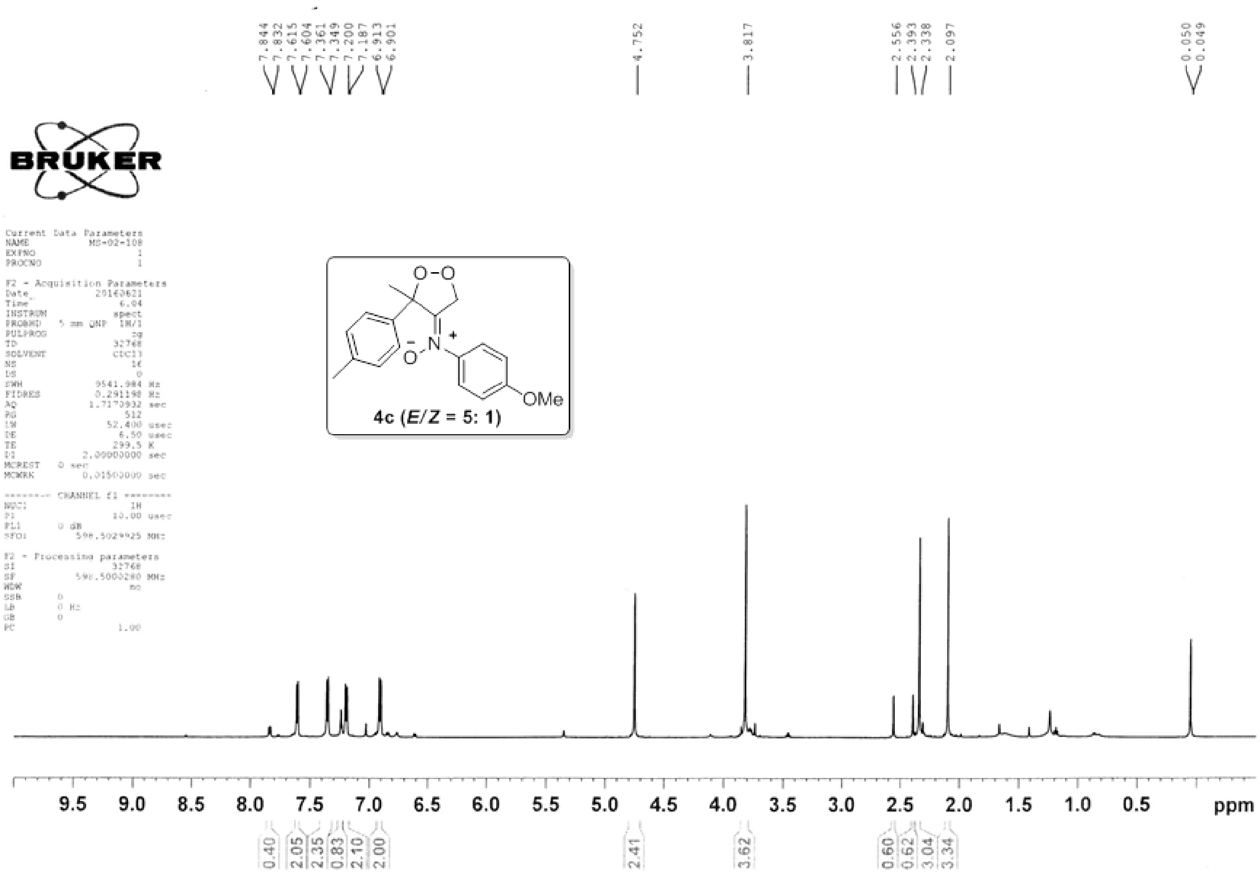
BRUKER
4c (*E*/*Z* = 5: 1)
OMe
ppm

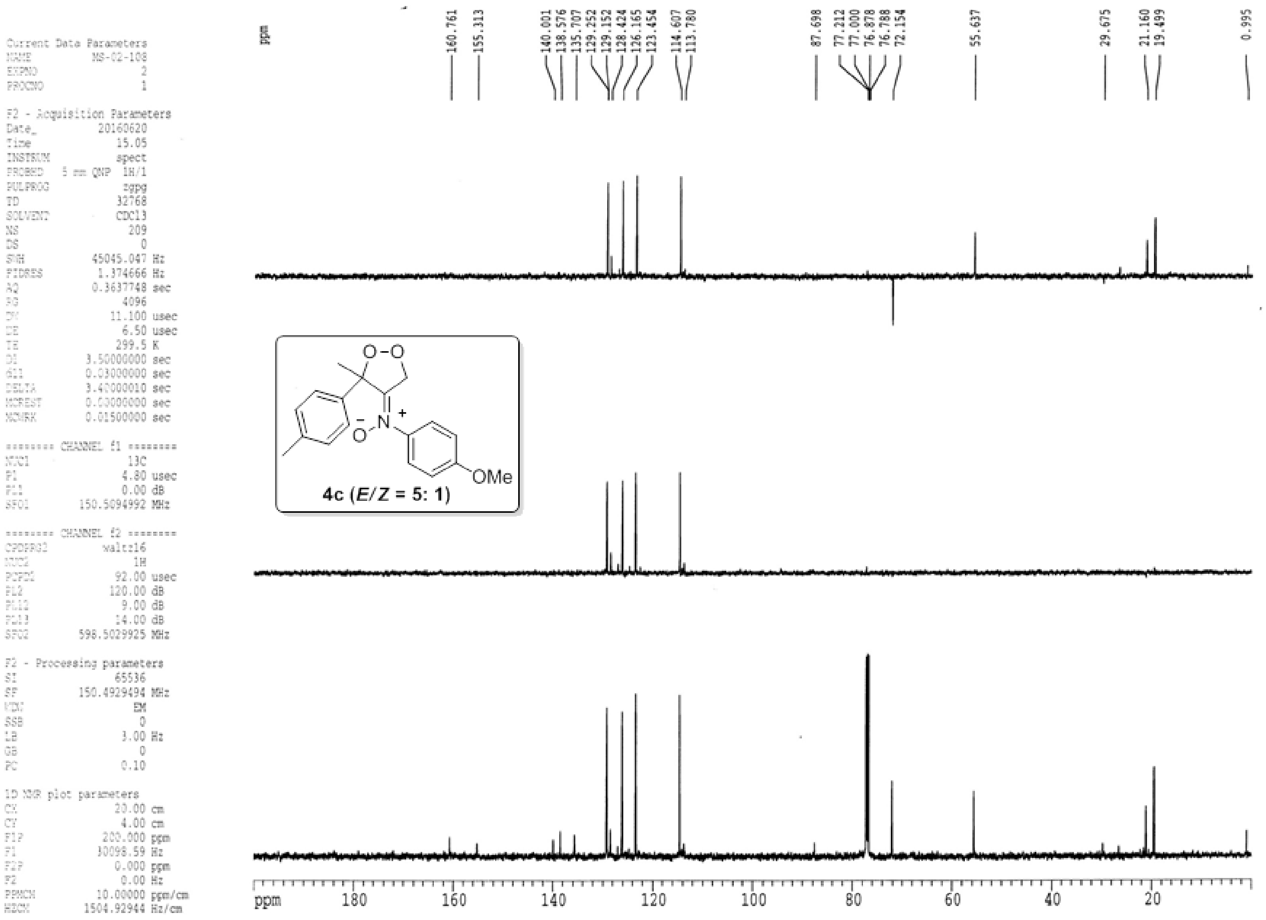

ppm
160.761
155.313
140.001
138.576
135.707
129.252
129.152
128.424
126.165
123.454
114.607
113.780
87.698
77.212
77.000
76.878
76.788
72.154
55.637
29.675
21.160
19.499
0.995
OMe
4c (E/Z = 5: 1)
ppm 180 160 140 120 100 80 60 40 20

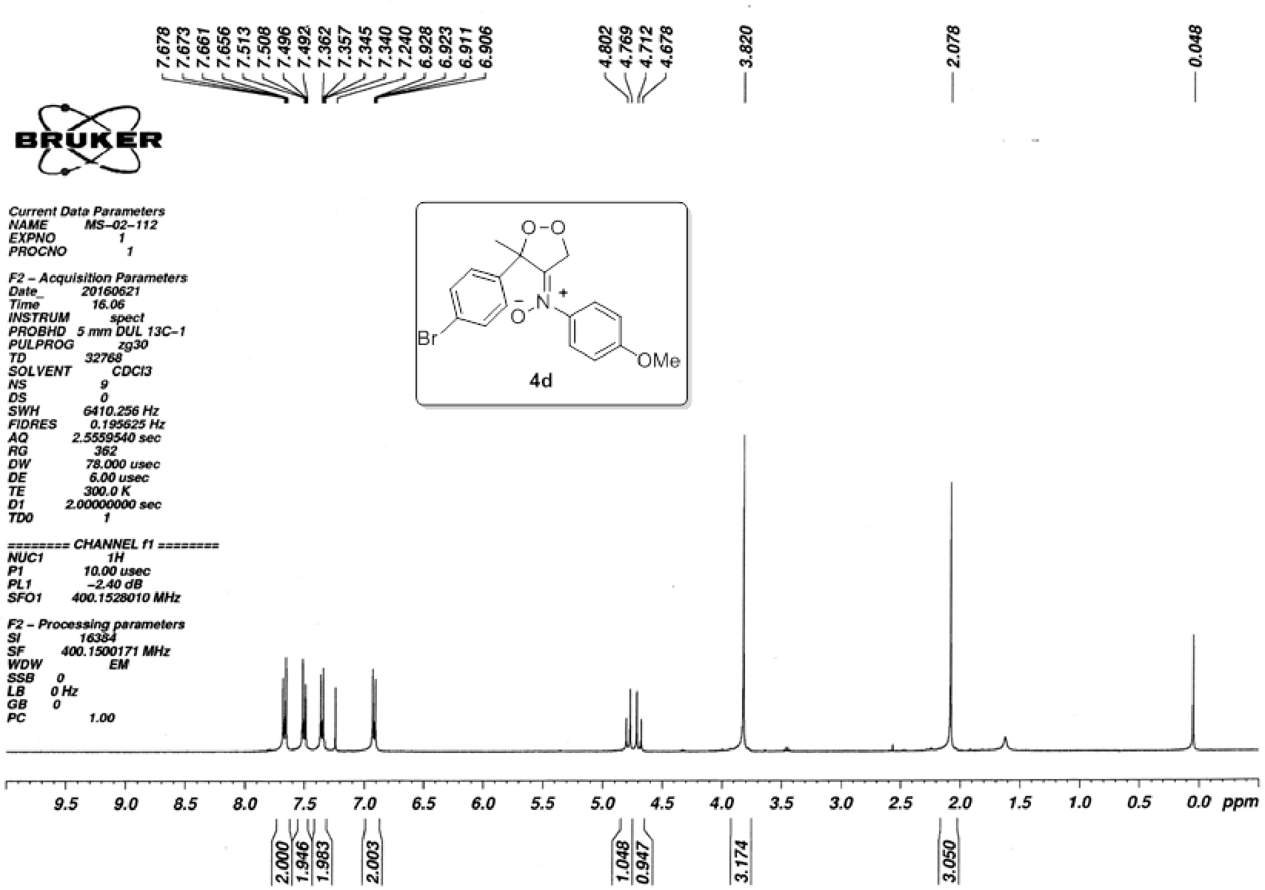
7.678 7.673 7.661 7.656 7.513 7.508 7.496 7.492 7.362 7.357 7.345 7.340 7.240 6.928 6.923 6.911 6.906
4.802 4.769 4.712 4.678
3.820
2.078
0.048
BRUKER
Current Data Parameters
NAME MS–02–112
EXPNO 1
PROCNO 1
F2 – Acquisition Parameters
Date_ 20160621
Time 16.06
INSTRUM spect
PROBHD 5 mm DUL 13C–1
PULPROG zg30
TD 32768
SOLVENT CDCl3
NS 9
DS 0
SWH 6410.256 Hz
FIDRES 0.195625 Hz
AQ 2.5559540 sec
RG 362
DW 78.000 usec
DE 6.00 usec
TE 300.0 K
D1 2.00000000 sec
TD0 1
======== CHANNEL f1 ========
NUC1 1H
P1 10.00 usec
PL1 –2.40 dB
SFO1 400.1528010 MHz
F2 – Processing parameters
SI 16384
SF 400.1500171 MHz
WDW EM
SSB 0
LB 0 Hz
GB 0
PC 1.00
O–O
N
Br
OMe
4d
9.5 9.0 8.5 8.0 7.5 7.0 6.5 6.0 5.5 5.0 4.5 4.0 3.5 3.0 2.5 2.0 1.5 1.0 0.5 0.0 ppm
2.000 1.946 1.983 2.003 1.048 0.947 3.174 3.050

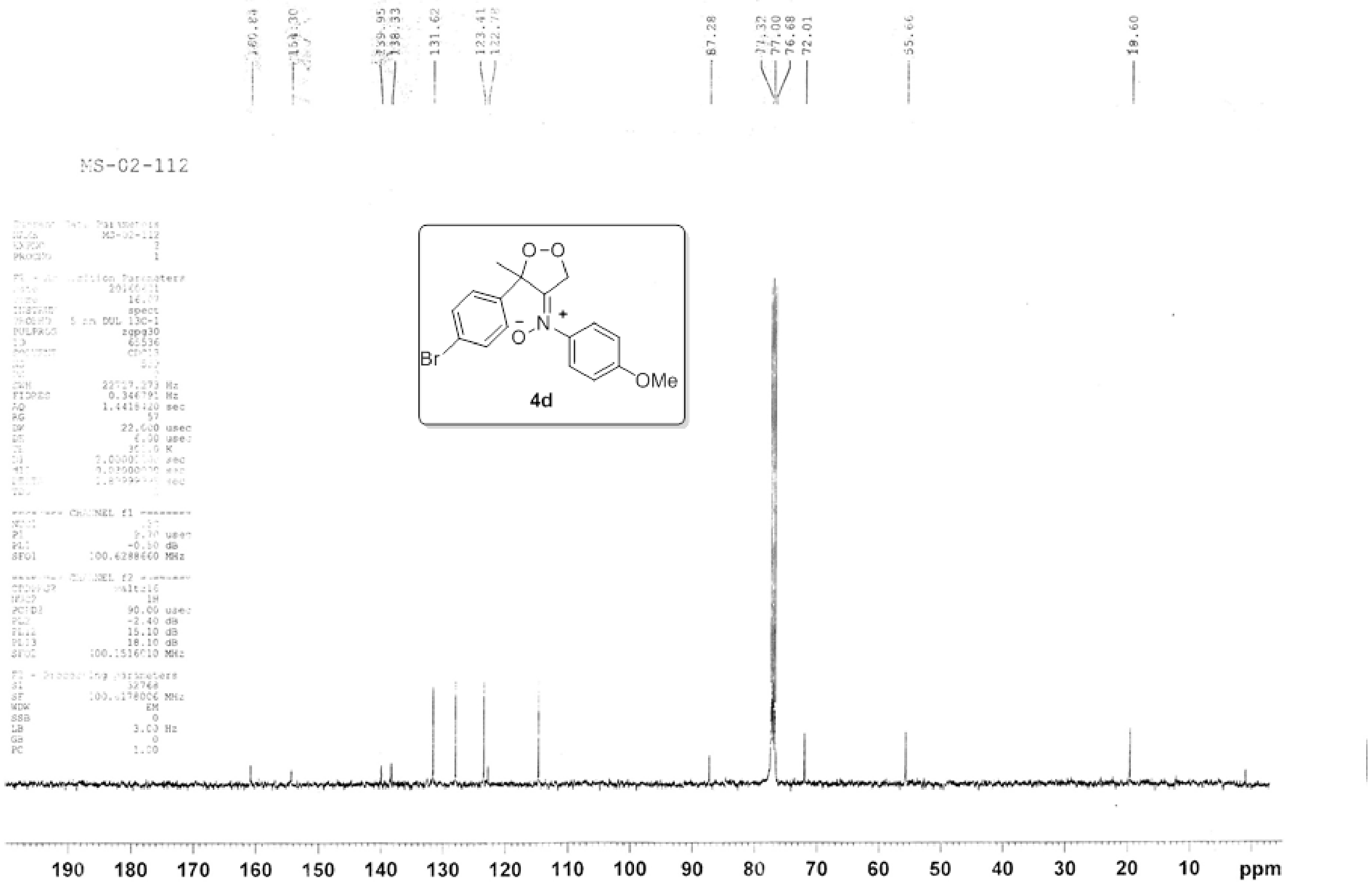
160.84
154.30
139.95
138.33
131.62
123.41
122.78
87.28
77.32
77.00
76.68
72.01
55.66
19.60
MS-C2-112
O–O
N
Br
OMe
4d
190 180 170 160 150 140 130 120 110 100 90 80 70 60 50 40 30 20 10 ppm

Current Data Parameters
NAME MS-02-160
DATE JUL 27 2016
SOLVENT CDCl3
EXPNO 1
PROCNO 1

F2 - Processing parameters
SI 32768
SF 399.7611793 MHz
WDW EM
SSB 0
LB 0.30 Hz
GB 0
PC 1.00

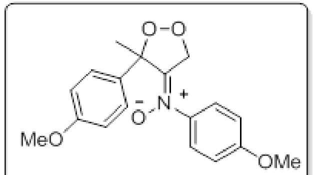

4e

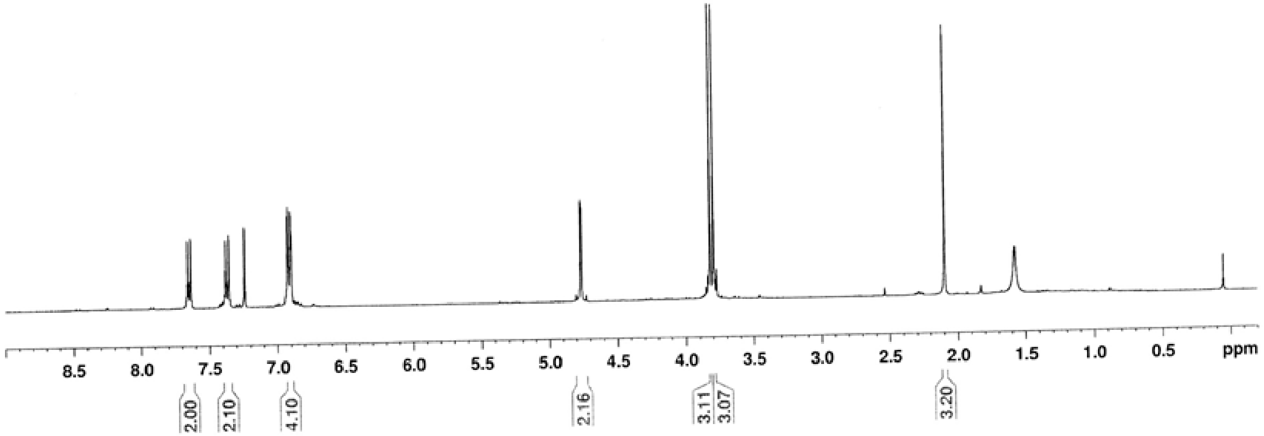

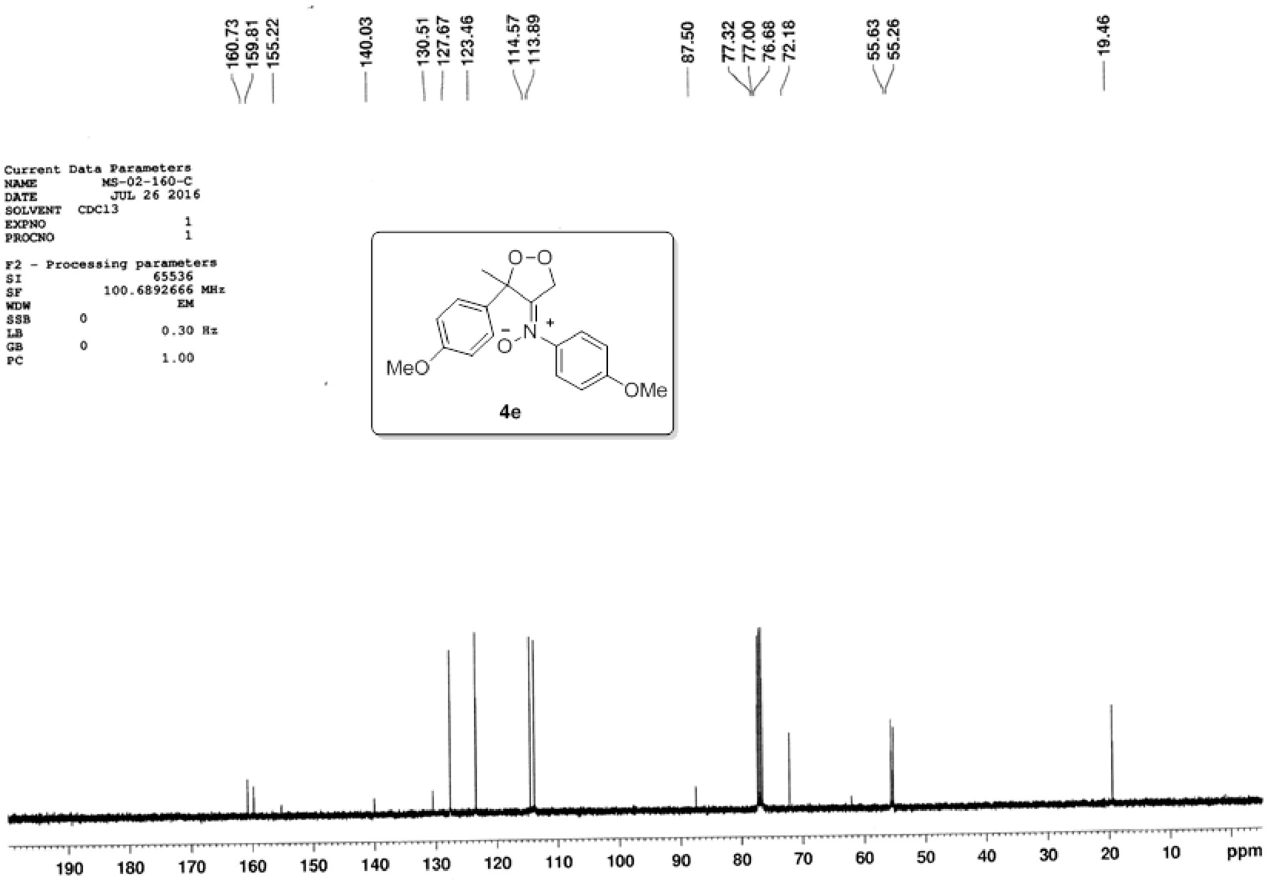
160.73
159.81
155.22
140.03
130.51
127.67
123.46
114.57
113.89
87.50
77.32
77.00
76.68
72.18
55.63
55.26
19.46
Current Data Parameters
NAME MS-02-160-C
DATE JUL 26 2016
SOLVENT CDCl3
EXPNO 1
PROCNO 1
F2 - Processing parameters
SI 65536
SF 100.6892666 MHz
WDW EM
SSB 0
LB 0.30 Hz
GB 0
PC 1.00
MeO
OMe
4e
190 180 170 160 150 140 130 120 110 100 90 80 70 60 50 40 30 20 10 ppm

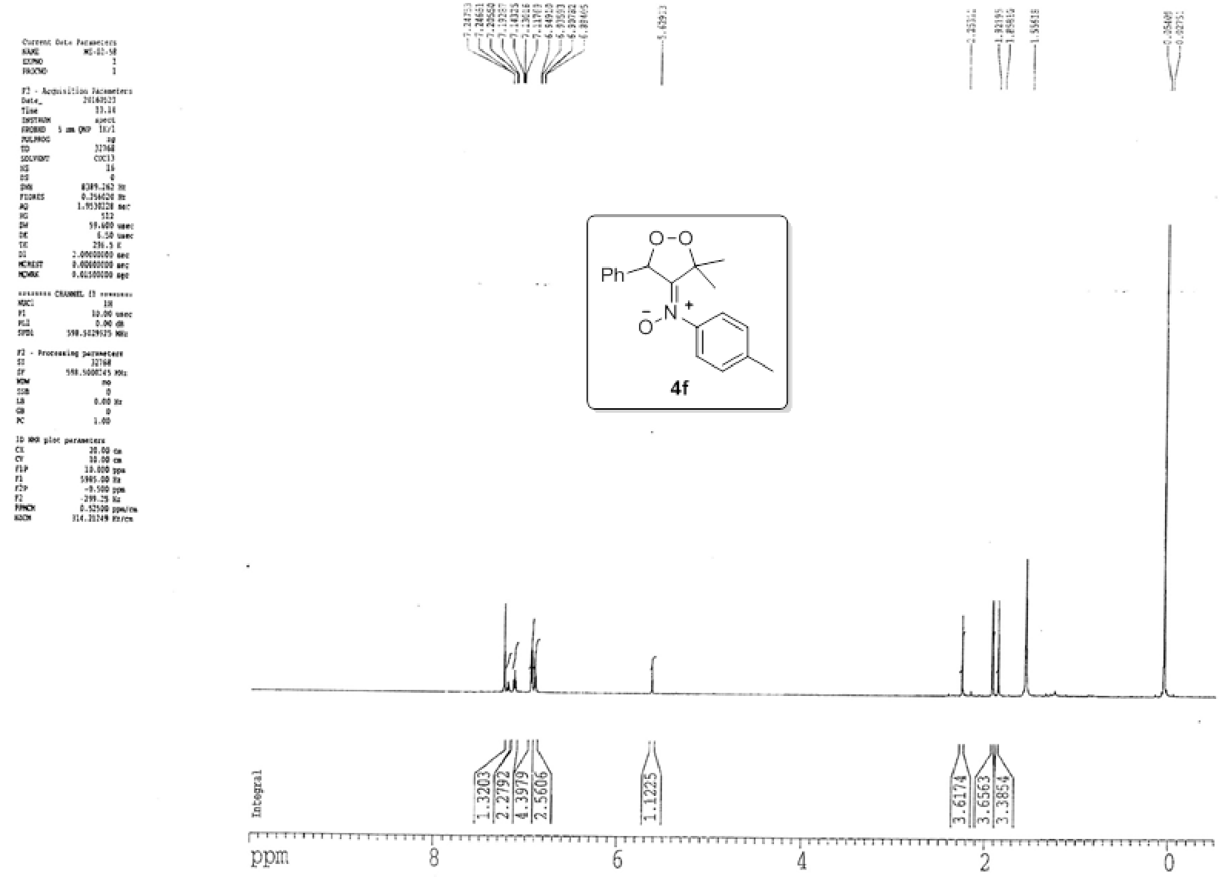
Ph
4f
Integral
1.3203
2.2792
4.3979
2.5606
1.1225
3.6174
3.6563
3.3854
ppm
8
6
4
2
0

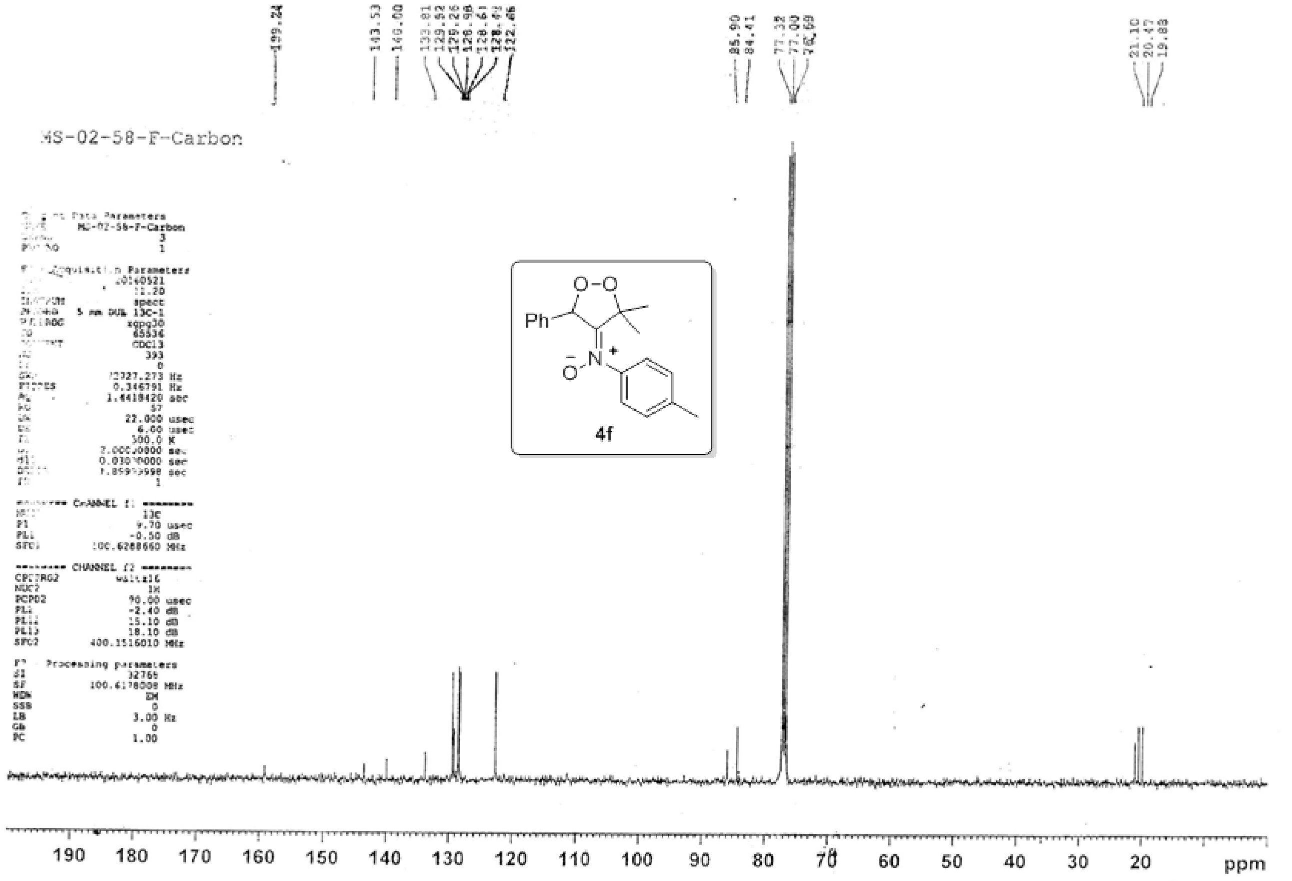
MS-02-58-F-Carbon
4f
ppm

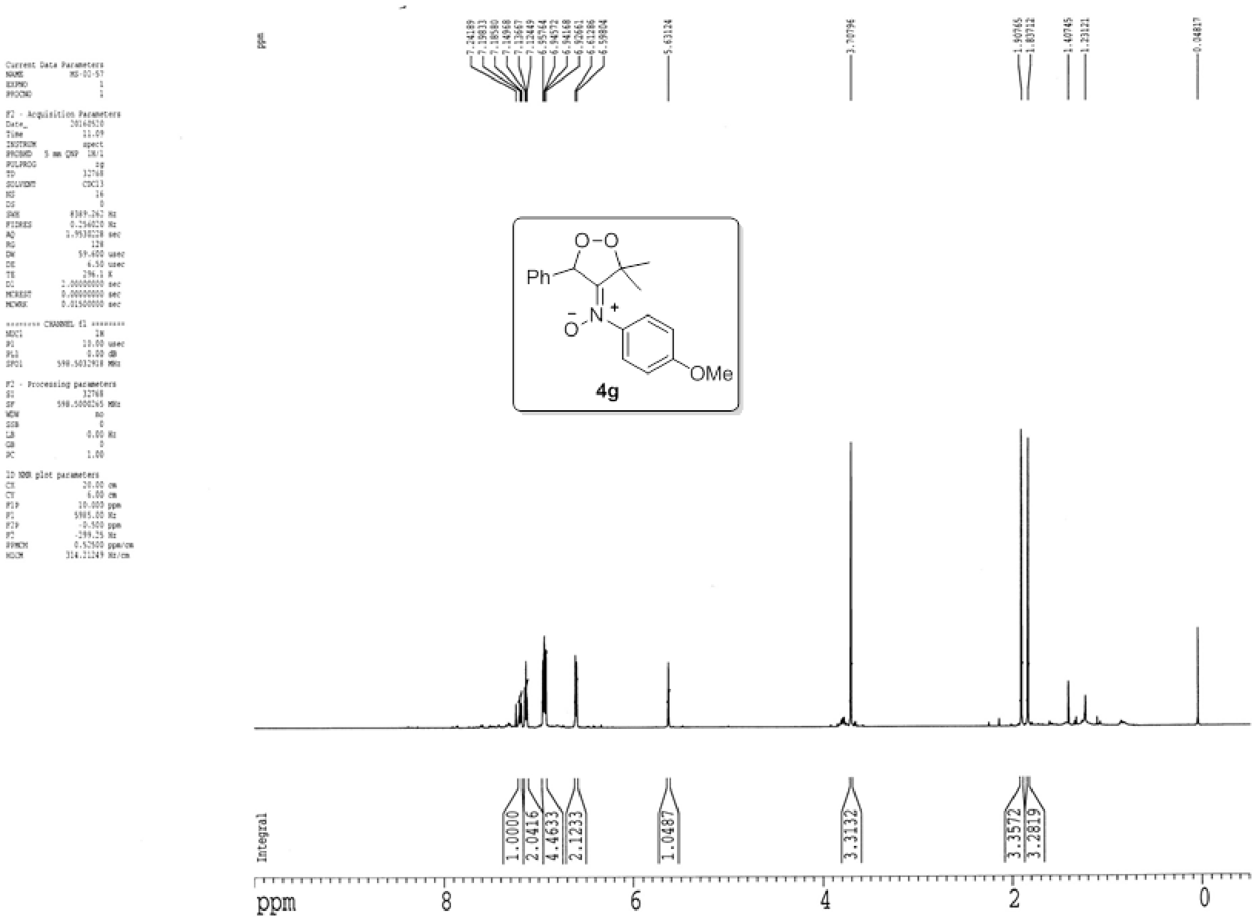
Ph
OMe
4g
Integral
1.0000
2.0416
4.4633
2.1233
1.0487
3.3132
3.3572
3.2819
ppm
8
6
4
2
0

```
Current Data Parameters
NAME            MS-02-57
EXPNO                  2
PROCNO                 1

F2 - Acquisition Parameters
Date_           20160520
Time               11.22
INSTRUM            spect
PROBHD   5 mm QNP  1H/1
PULPROG             zgpg
TD                 32768
SOLVENT            CDCl3
NS                   200
DS                     0
SWH            45045.047 Hz
FIDRES          1.374666 Hz
AQ             0.3637748 sec
RG                  4096
DW                11.100 usec
DE                  6.50 usec
TE                 297.1 K
D1            3.50000000 sec
d11           0.03000000 sec
DELTA         3.40000010 sec
MCREST        0.00000000 sec
MCWRK         0.01500000 sec

======== CHANNEL f1 ========
NUC1                 13C
P1                  4.80 usec
PL1                 0.00 dB
SFO1         150.5094992 MHz

======== CHANNEL f2 ========
CPDPRG2          waltz16
NUC2                  1H
PCPD2              92.00 usec
PL2               120.00 dB
PL12                9.00 dB
PL13               14.00 dB
SFO2         598.5029925 MHz

F2 - Processing parameters
SI                 32768
SF           150.4929529 MHz
WDW                   EM
SSB                    0
LB                  3.00 Hz
GB                     0
PC                  1.00

1D NMR plot parameters
CX                 20.00 cm
CY                  4.00 cm
F1P              200.000 ppm
F1              30098.59 Hz
F2P                0.000 ppm
F2                  0.00 Hz
PPMCM           10.00000 ppm/cm
HZCM          1504.92944 Hz/cm
```

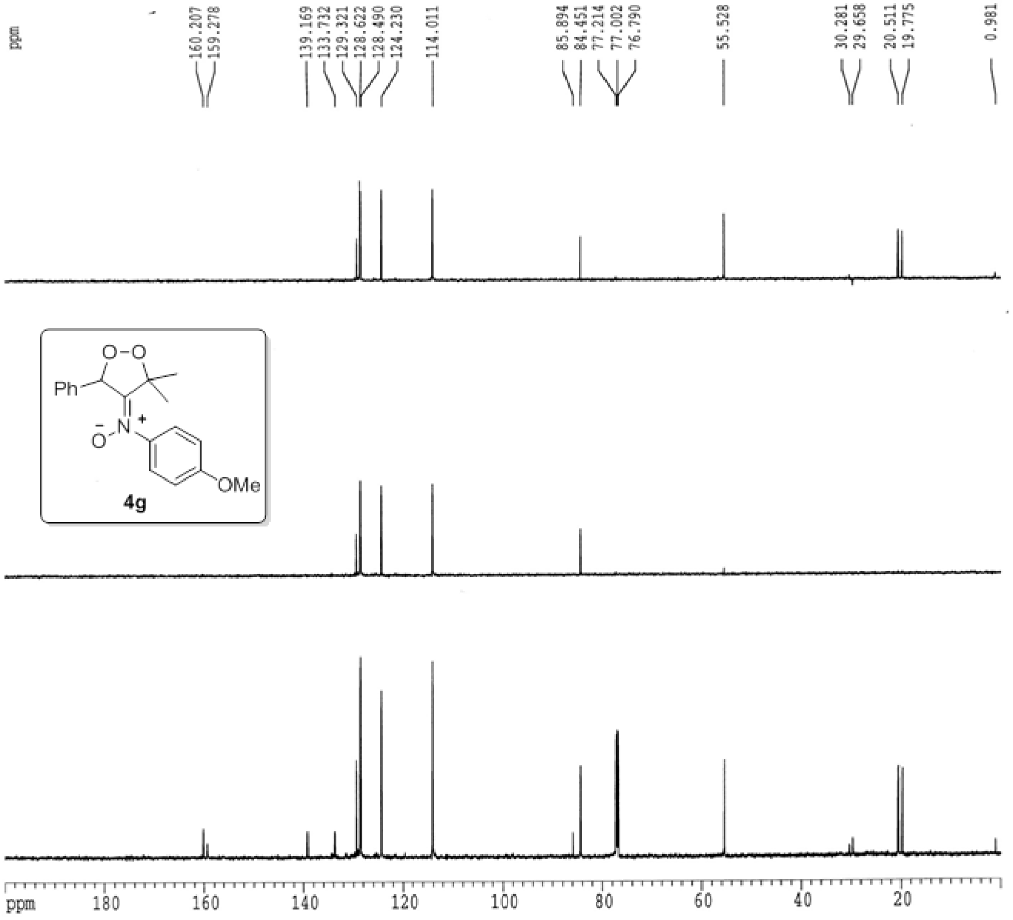

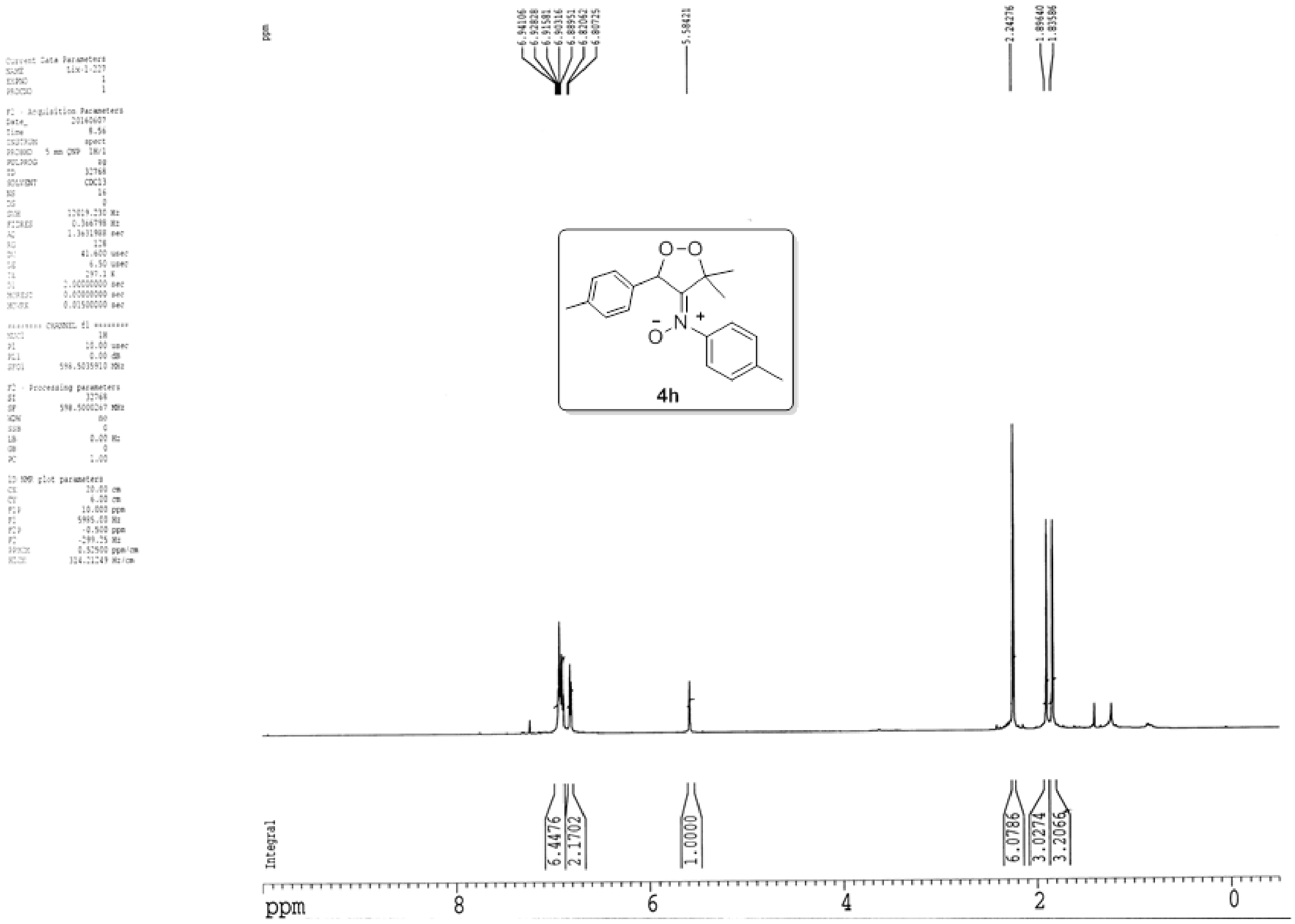
6.94106
6.92828
6.91581
6.90316
6.88951
6.82062
6.80725
5.58421
2.24276
1.89640
1.83586
4h
6.4476
2.1702
1.0000
6.0786
3.0274
3.2066
Integral
ppm
8
6
4
2
0

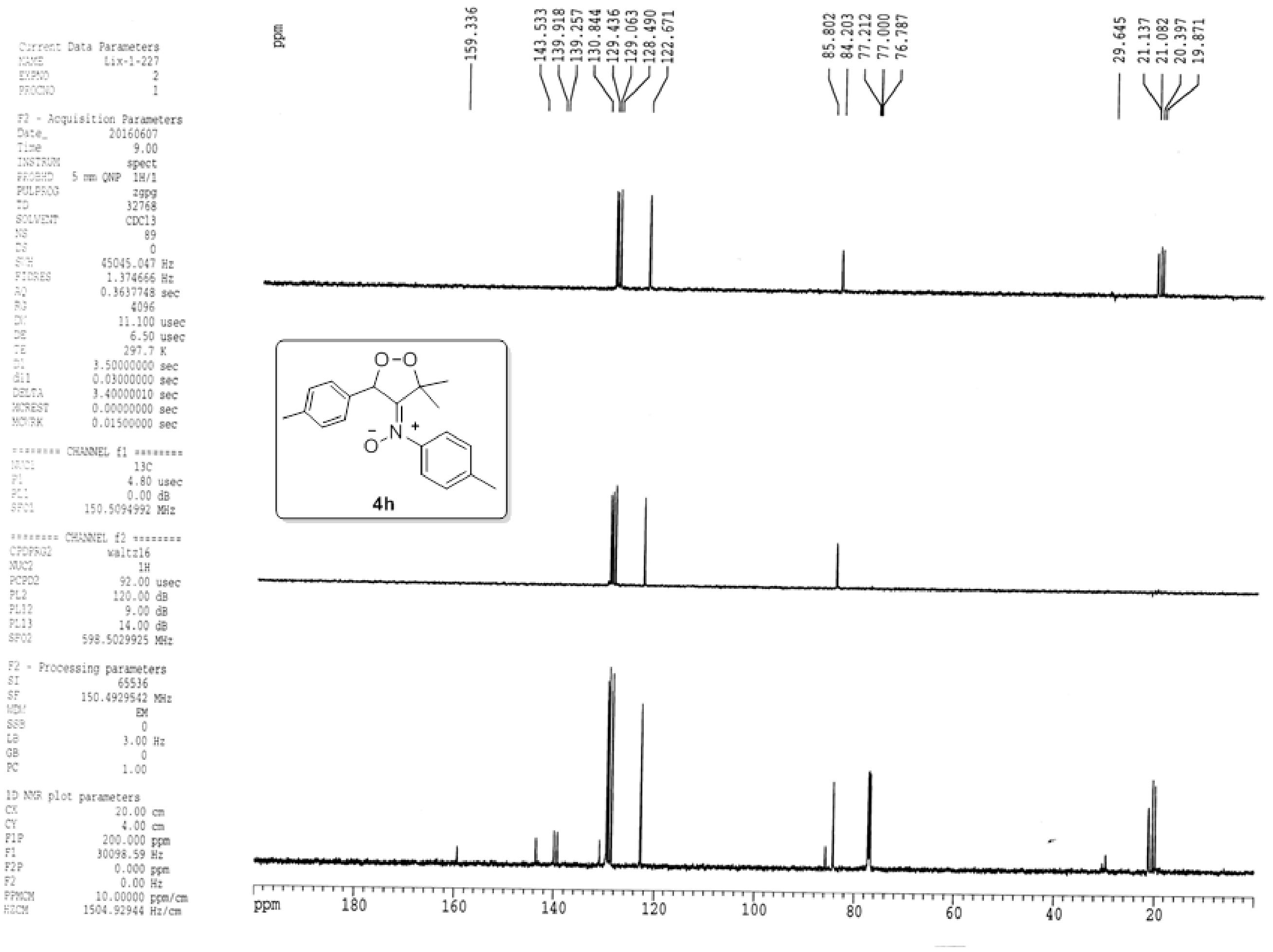
Current Data Parameters
NAME Lix-1-227
EXPNO 2
PROCNO 1
F2 - Acquisition Parameters
Date_ 20160607
Time 9.00
INSTRUM spect
PROBHD 5 mm QNP 1H/1
PULPROG zgpg
TD 32768
SOLVENT CDCl3
NS 89
DS 0
SWH 45045.047 Hz
FIDRES 1.374666 Hz
AQ 0.3637748 sec
RG 4096
DW 11.100 usec
DE 6.50 usec
TE 297.7 K
D1 3.50000000 sec
d11 0.03000000 sec
DELTA 3.40000010 sec
MCREST 0.00000000 sec
MCWRK 0.01500000 sec
======== CHANNEL f1 ========
NUC1 13C
P1 4.80 usec
PL1 0.00 dB
SFO1 150.5094992 MHz
======== CHANNEL f2 ========
CPDPRG2 waltz16
NUC2 1H
PCPD2 92.00 usec
PL2 120.00 dB
PL12 9.00 dB
PL13 14.00 dB
SFO2 598.5029925 MHz
F2 - Processing parameters
SI 65536
SF 150.4929542 MHz
WDW EM
SSB 0
LB 3.00 Hz
GB 0
PC 1.00
1D NMR plot parameters
CX 20.00 cm
CY 4.00 cm
F1P 200.000 ppm
F1 30098.59 Hz
F2P 0.000 ppm
F2 0.00 Hz
PPMCM 10.00000 ppm/cm
HZCM 1504.92944 Hz/cm
ppm
159.336
143.533
139.918
139.257
130.844
129.436
129.063
128.490
122.671
85.802
84.203
77.212
77.000
76.787
29.645
21.137
21.082
20.397
19.871
4h
ppm 180 160 140 120 100 80 60 40 20

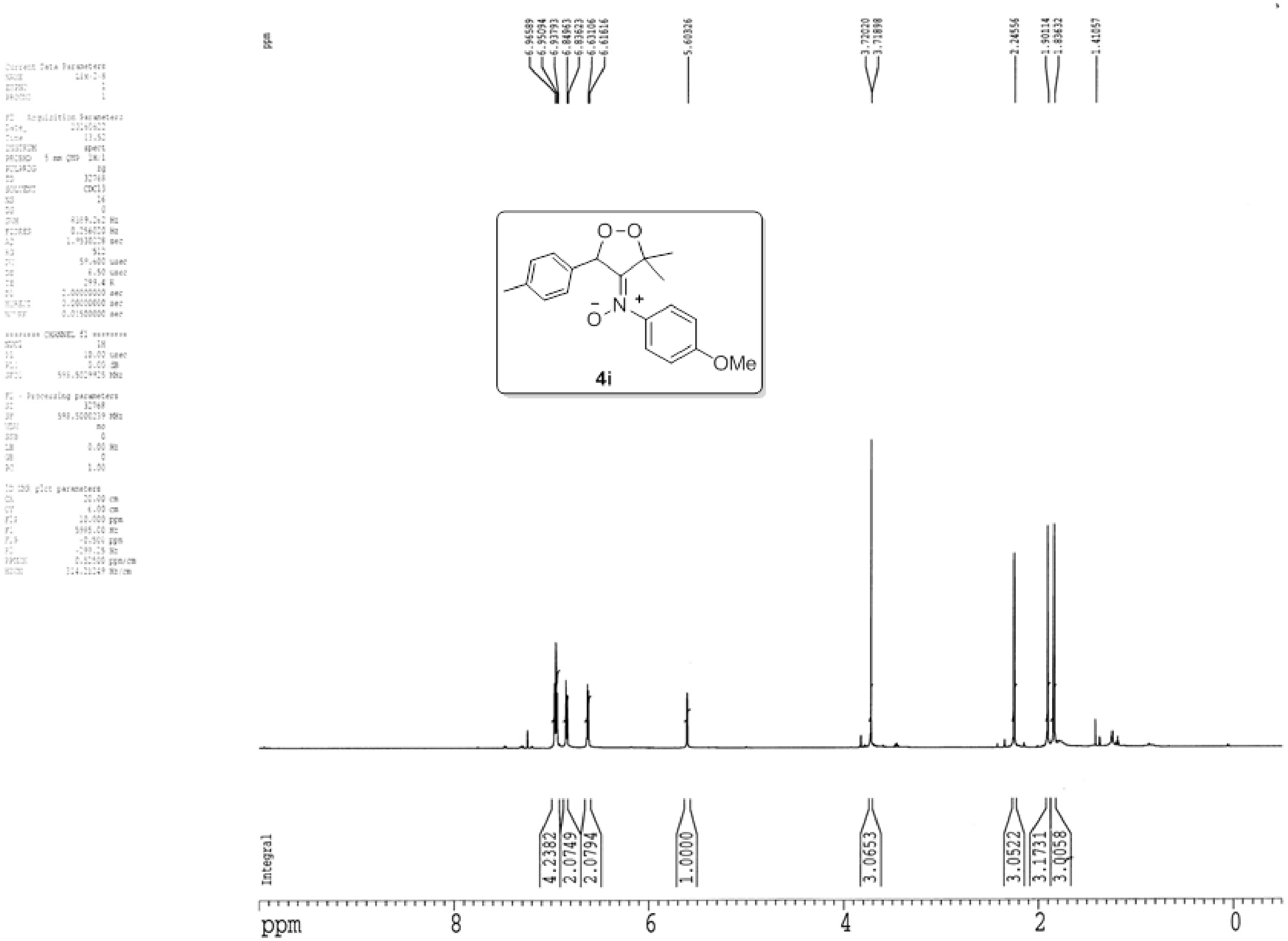
ppm
6.96589
6.95094
6.93793
6.84963
6.83623
6.63106
6.61616
5.60326
3.72020
3.71898
2.24556
1.90114
1.83632
1.41057
O–O
N
O
OMe
4i
Integral
4.2382
2.0749
2.0794
1.0000
3.0653
3.0522
3.1731
3.0058
ppm
8
6
4
2
0

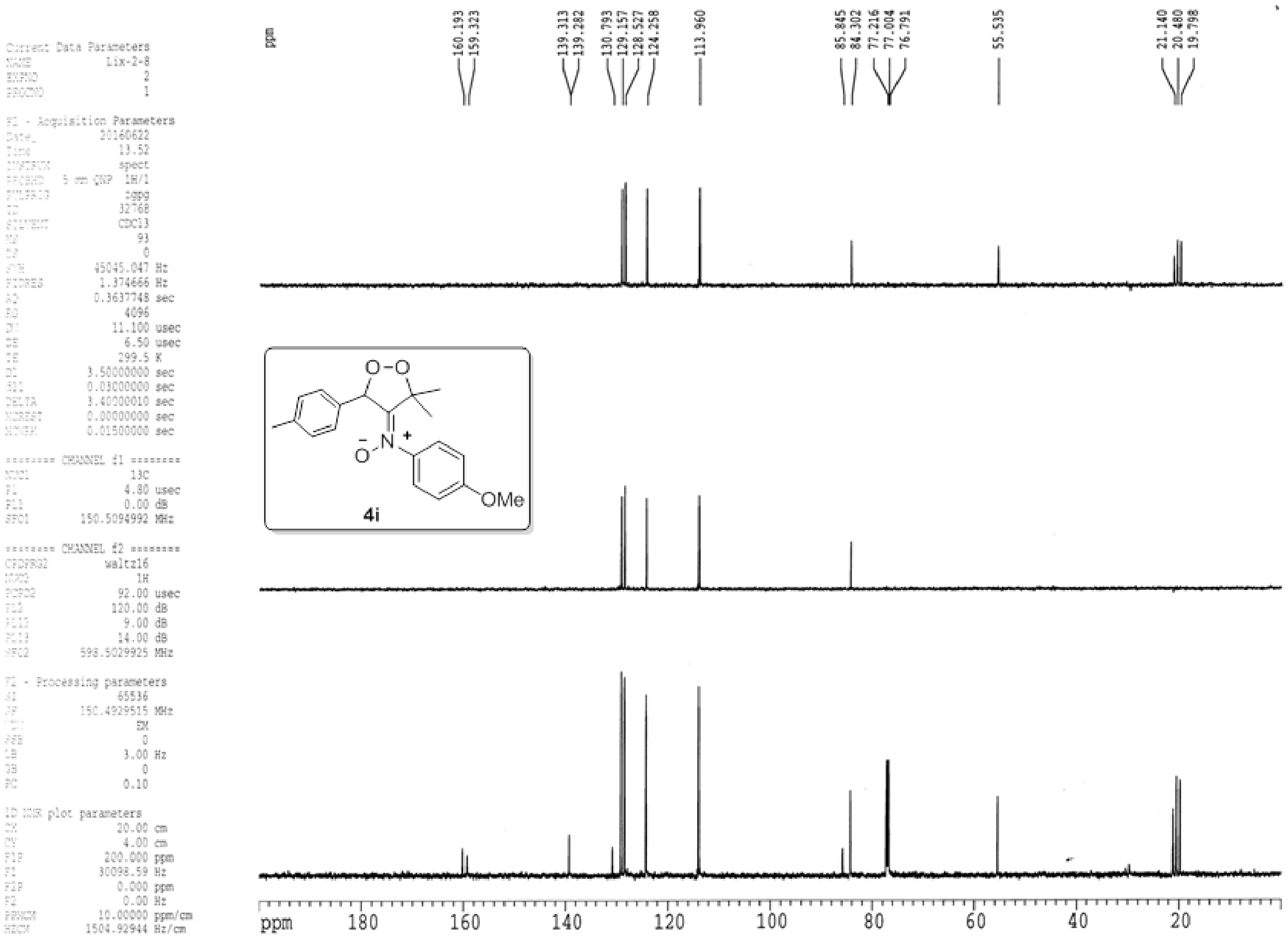
Current Data Parameters
NAME Lix-2-8
EXPNO 2
PROCNO 1
F2 - Acquisition Parameters
Date_ 20160622
Time 13.52
INSTRUM spect
PROBHD 5 mm QNP 1H/1
PULPROG zgpg
TD 32768
SOLVENT CDCl3
NS 93
DS 0
SWH 45045.047 Hz
FIDRES 1.374666 Hz
AQ 0.3637748 sec
RG 4096
DW 11.100 usec
DE 6.50 usec
TE 299.5 K
D1 3.50000000 sec
d11 0.03000000 sec
DELTA 3.40000010 sec
MCREST 0.00000000 sec
MCWRK 0.01500000 sec
======== CHANNEL f1 ========
NUC1 13C
P1 4.80 usec
PL1 0.00 dB
SFO1 150.5094992 MHz
======== CHANNEL f2 ========
CPDPRG2 waltz16
NUC2 1H
PCPD2 92.00 usec
PL2 120.00 dB
PL12 9.00 dB
PL13 14.00 dB
SFO2 598.5029925 MHz
F2 - Processing parameters
SI 65536
SF 150.4929515 MHz
WDW EM
SSB 0
LB 3.00 Hz
GB 0
PC 0.10
1D NMR plot parameters
CX 20.00 cm
CY 4.00 cm
F1P 200.000 ppm
F1 30098.59 Hz
F2P 0.000 ppm
F2 0.00 Hz
PPMCM 10.00000 ppm/cm
HZCM 1504.92944 Hz/cm
ppm
160.193
159.323
139.313
139.282
130.793
129.157
128.527
124.258
113.960
85.845
84.302
77.216
77.004
76.791
55.535
21.140
20.480
19.798
O-O
N
+
-
O
OMe
4i
ppm
180
160
140
120
100
80
60
40
20

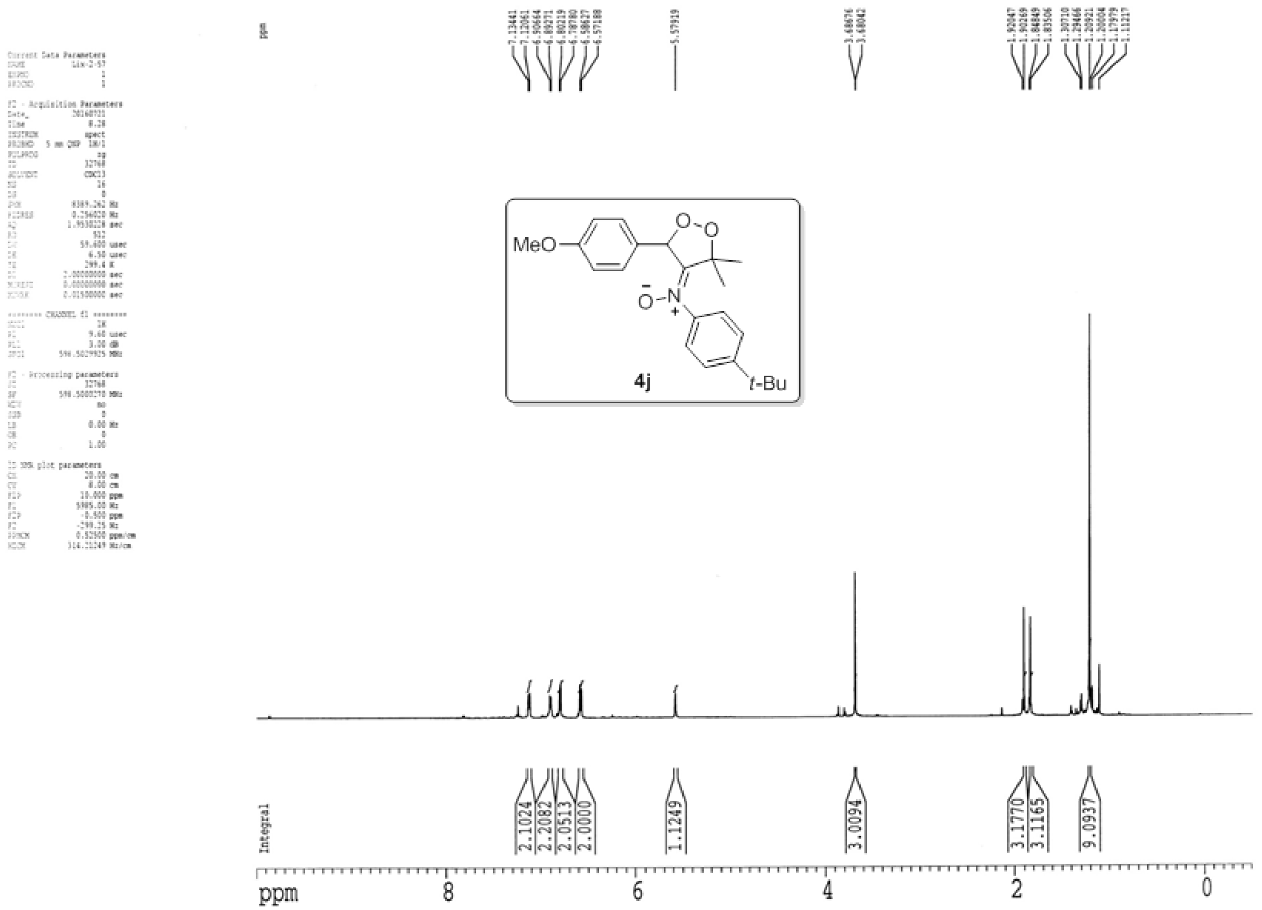
7.13441
7.12061
6.90664
6.89271
6.80219
6.78780
6.58627
6.57188
5.57919
3.68676
3.68042
1.92047
1.90269
1.84849
1.83506
1.30710
1.29466
1.20921
1.20004
1.17979
1.11217
MeO
4j
t-Bu
Integral
2.1024
2.2082
2.0513
2.0000
1.1249
3.0094
3.1770
3.1165
9.0937
ppm
8
6
4
2
0

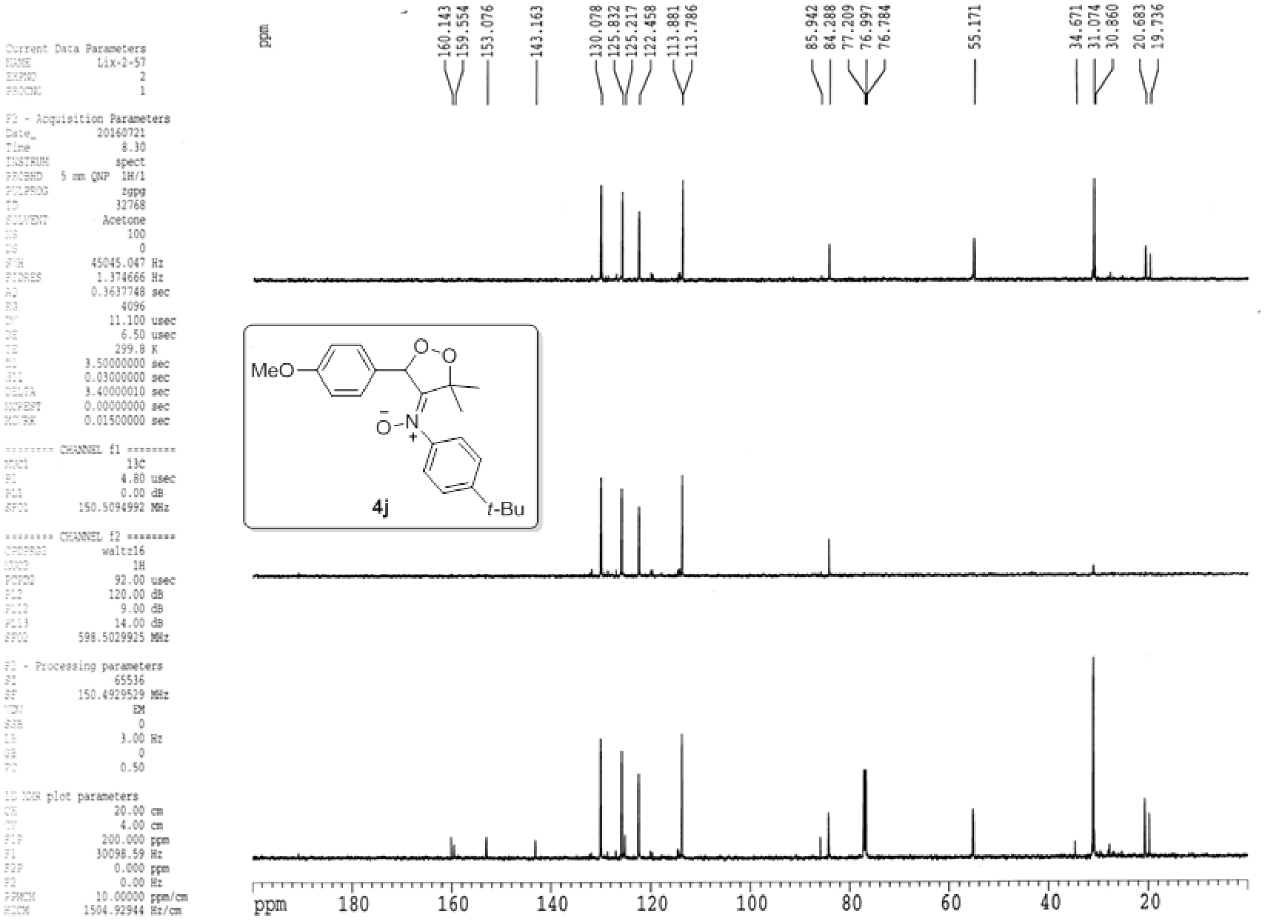
Current Data Parameters
Lix-2-57
F2 - Acquisition Parameters
20160721
8.30
spect
5 mm QNP 1H/1
zgpg
32768
Acetone
100
45045.047 Hz
1.374666 Hz
0.3637748 sec
4096
11.100 usec
6.50 usec
299.8 K
3.50000000 sec
0.03000000 sec
3.40000010 sec
0.00000000 sec
0.01500000 sec
======== CHANNEL f1 ========
13C
4.80 usec
0.00 dB
150.5094992 MHz
======== CHANNEL f2 ========
waltz16
1H
92.00 usec
120.00 dB
9.00 dB
14.00 dB
598.5029925 MHz
F2 - Processing parameters
65536
150.4929529 MHz
EM
0
3.00 Hz
0
0.50
1D NMR plot parameters
20.00 cm
4.00 cm
200.000 ppm
30098.59 Hz
0.000 ppm
0.00 Hz
10.00000 ppm/cm
1504.92944 Hz/cm
ppm
160.143
159.554
153.076
143.163
130.078
125.832
125.217
122.458
113.881
113.786
85.942
84.288
77.209
76.997
76.784
55.171
34.671
31.074
30.860
20.683
19.736
MeO
O
O
N
+
4j
t-Bu
ppm
180
160
140
120
100
80
60
40
20

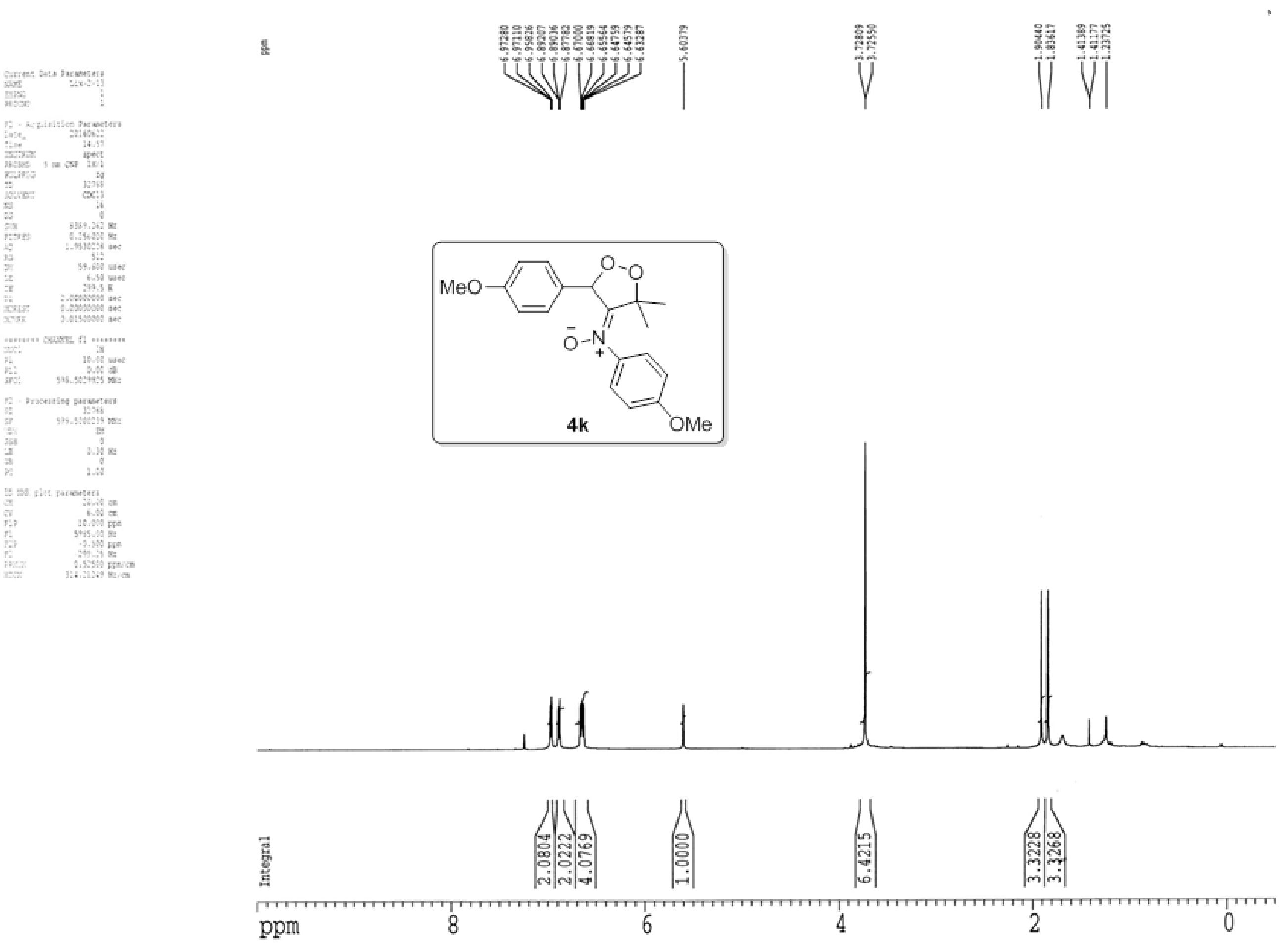
6.97280
6.97110
6.95826
6.89207
6.89036
6.87782
6.67000
6.66819
6.65564
6.64759
6.64579
6.63287
5.60379
3.72809
3.72550
1.90440
1.83617
1.41389
1.41177
1.23725
MeO
O
O
N
OMe
4k
2.0804
2.0222
4.0769
1.0000
6.4215
3.3228
3.3268
Integral
ppm
8
6
4
2
0

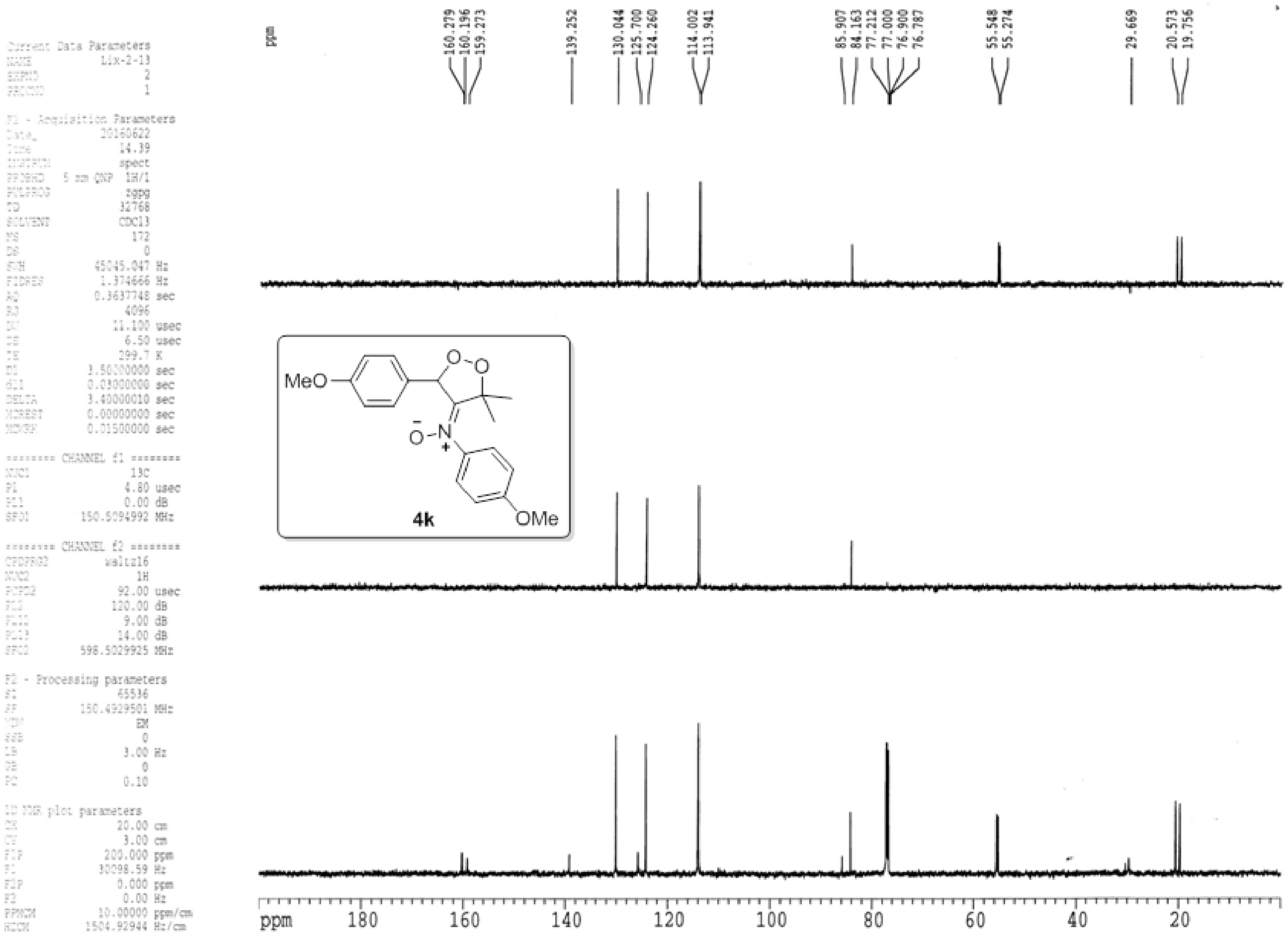
Current Data Parameters
NAME Lix-2-13
EXPNO 2
PROCNO 1
F2 - Acquisition Parameters
Date_ 20160622
Time 14.39
INSTRUM spect
PROBHD 5 mm QNP 1H/1
PULPROG zgpg
TD 32768
SOLVENT CDCl3
NS 172
DS 0
SWH 45045.047 Hz
FIDRES 1.374666 Hz
AQ 0.3637748 sec
RG 4096
DW 11.100 usec
DE 6.50 usec
TE 299.7 K
D1 3.50000000 sec
d11 0.03000000 sec
DELTA 3.40000010 sec
MCREST 0.00000000 sec
MCWRK 0.01500000 sec
======== CHANNEL f1 ========
NUC1 13C
P1 4.80 usec
PL1 0.00 dB
SFO1 150.5094992 MHz
======== CHANNEL f2 ========
CPDPRG2 waltz16
NUC2 1H
PCPD2 92.00 usec
PL2 120.00 dB
PL12 9.00 dB
PL13 14.00 dB
SFO2 598.5029925 MHz
F2 - Processing parameters
SI 65536
SF 150.4929501 MHz
WDW EM
SSB 0
LB 3.00 Hz
GB 0
PC 0.10
1D NMR plot parameters
CX 20.00 cm
CY 3.00 cm
F1P 200.000 ppm
F1 30098.59 Hz
F2P 0.000 ppm
F2 0.00 Hz
PPMCM 10.00000 ppm/cm
HZCM 1504.92944 Hz/cm
ppm
160.279
160.196
159.273
139.252
130.044
125.700
124.260
114.002
113.941
85.907
84.163
77.212
77.000
76.900
76.787
55.548
55.274
29.669
20.573
19.756
MeO
O
O
O
N
+
OMe
4k
ppm
180
160
140
120
100
80
60
40
20

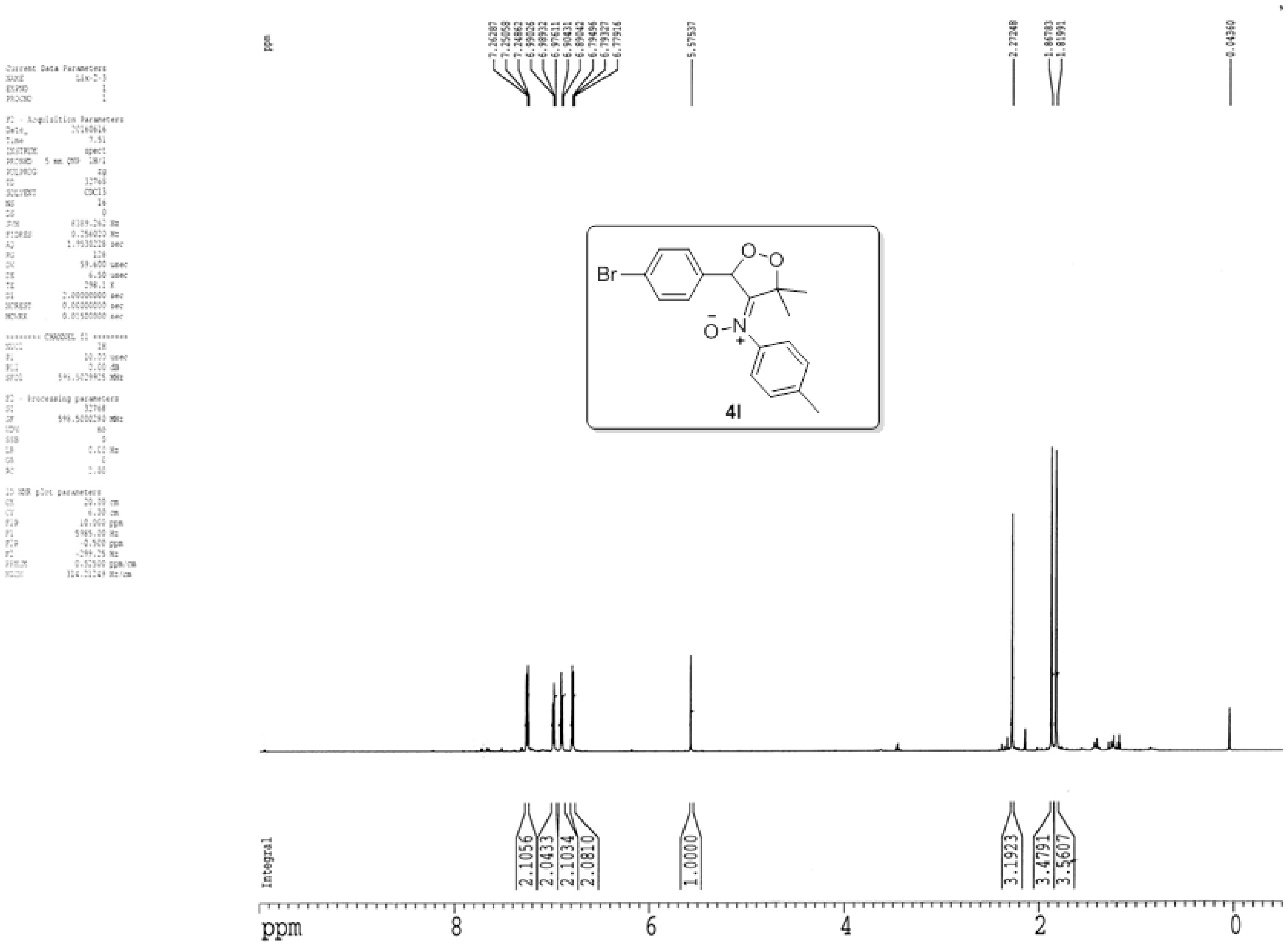
7.26287
7.25058
7.24862
6.99026
6.98932
6.97611
6.90431
6.89042
6.79496
6.79327
6.77916
5.57537
2.27248
1.86783
1.81991
0.04360
Br
O
O
N
4l
Integral
2.1056
2.0433
2.1034
2.0810
1.0000
3.1923
3.4791
3.5607
ppm
8
6
4
2
0

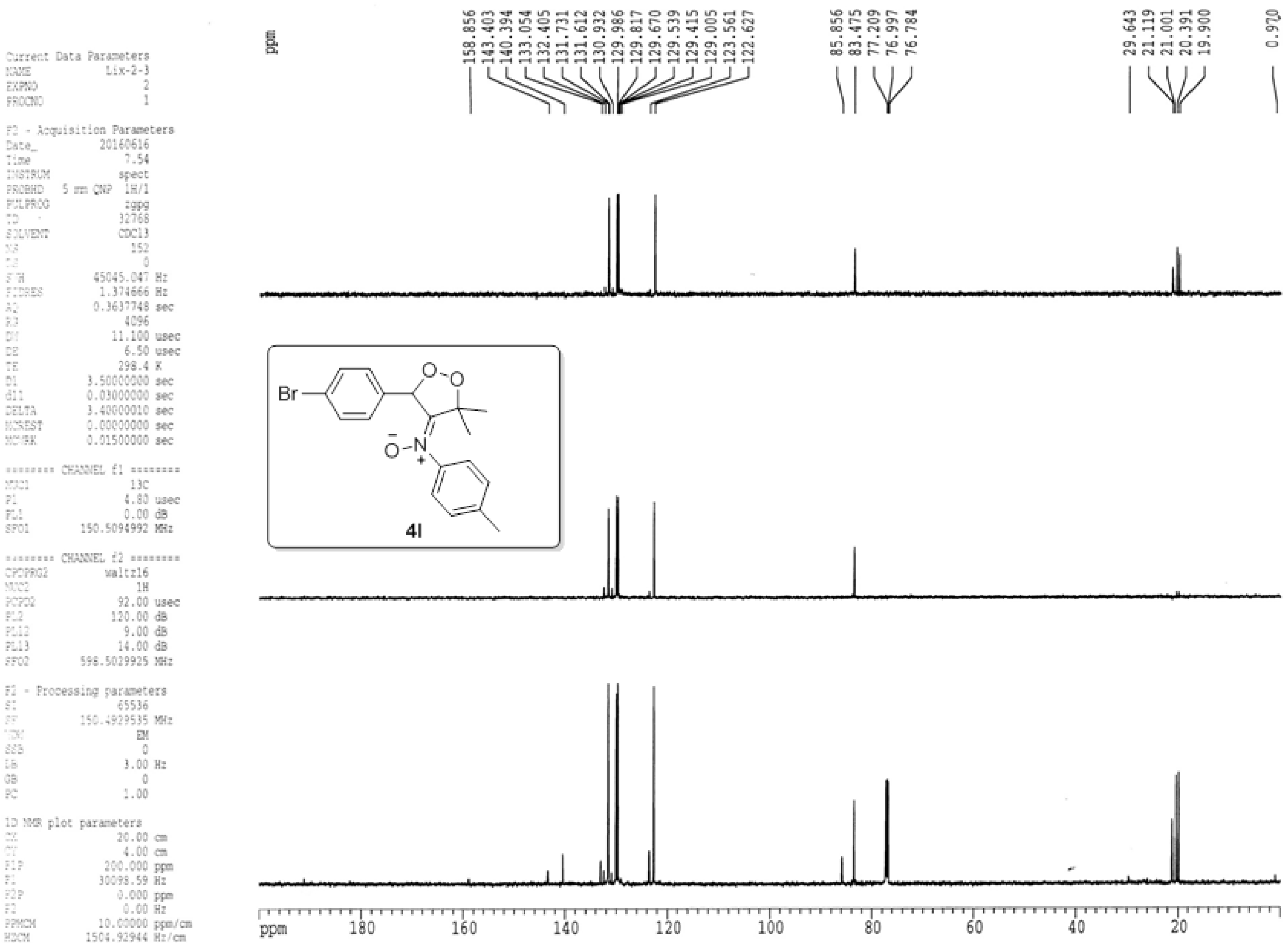
Current Data Parameters
NAME Lix-2-3
EXPNO 2
PROCNO 1
F2 - Acquisition Parameters
Date_ 20160616
Time 7.54
INSTRUM spect
PROBHD 5 mm QNP 1H/1
PULPROG zgpg
TD 32768
SOLVENT CDCl3
NS 152
DS 0
SWH 45045.047 Hz
FIDRES 1.374666 Hz
AQ 0.3637748 sec
RG 4096
DW 11.100 usec
DE 6.50 usec
TE 298.4 K
D1 3.50000000 sec
d11 0.03000000 sec
DELTA 3.40000010 sec
MCREST 0.00000000 sec
MCWRK 0.01500000 sec
======== CHANNEL f1 ========
NUC1 13C
P1 4.80 usec
PL1 0.00 dB
SFO1 150.5094992 MHz
======== CHANNEL f2 ========
CPDPRG2 waltz16
NUC2 1H
PCPD2 92.00 usec
PL2 120.00 dB
PL12 9.00 dB
PL13 14.00 dB
SFO2 598.5029925 MHz
F2 - Processing parameters
SI 65536
SF 150.4929535 MHz
WDW EM
SSB 0
LB 3.00 Hz
GB 0
PC 1.00
1D NMR plot parameters
CX 20.00 cm
CY 4.00 cm
F1P 200.000 ppm
F1 30098.59 Hz
F2P 0.000 ppm
F2 0.00 Hz
PPMCM 10.00000 ppm/cm
HZCM 1504.92944 Hz/cm
ppm
158.856
143.403
140.394
133.054
132.405
131.731
131.612
130.932
129.986
129.817
129.670
129.539
129.415
129.005
123.561
122.627
85.856
83.475
77.209
76.997
76.784
29.643
21.119
21.001
20.391
19.900
0.970
Br
O
O
O
N
4l
ppm
180
160
140
120
100
80
60
40
20

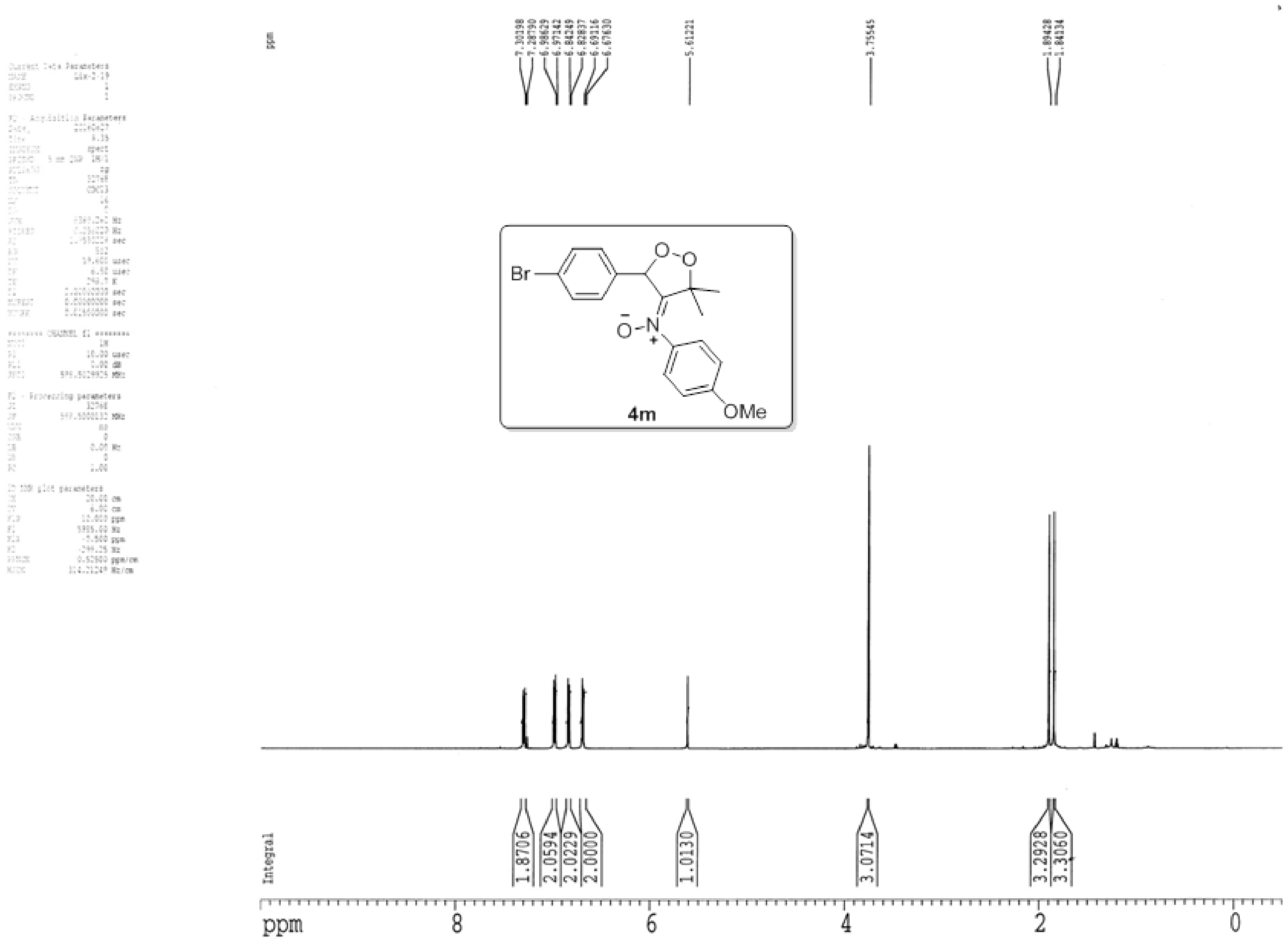
Br
OMe
4m
5.61221
3.75545
Integral
1.8706
2.0594
2.0229
2.0000
1.0130
3.0714
3.2928
3.3060
ppm
8
6
4
2
0

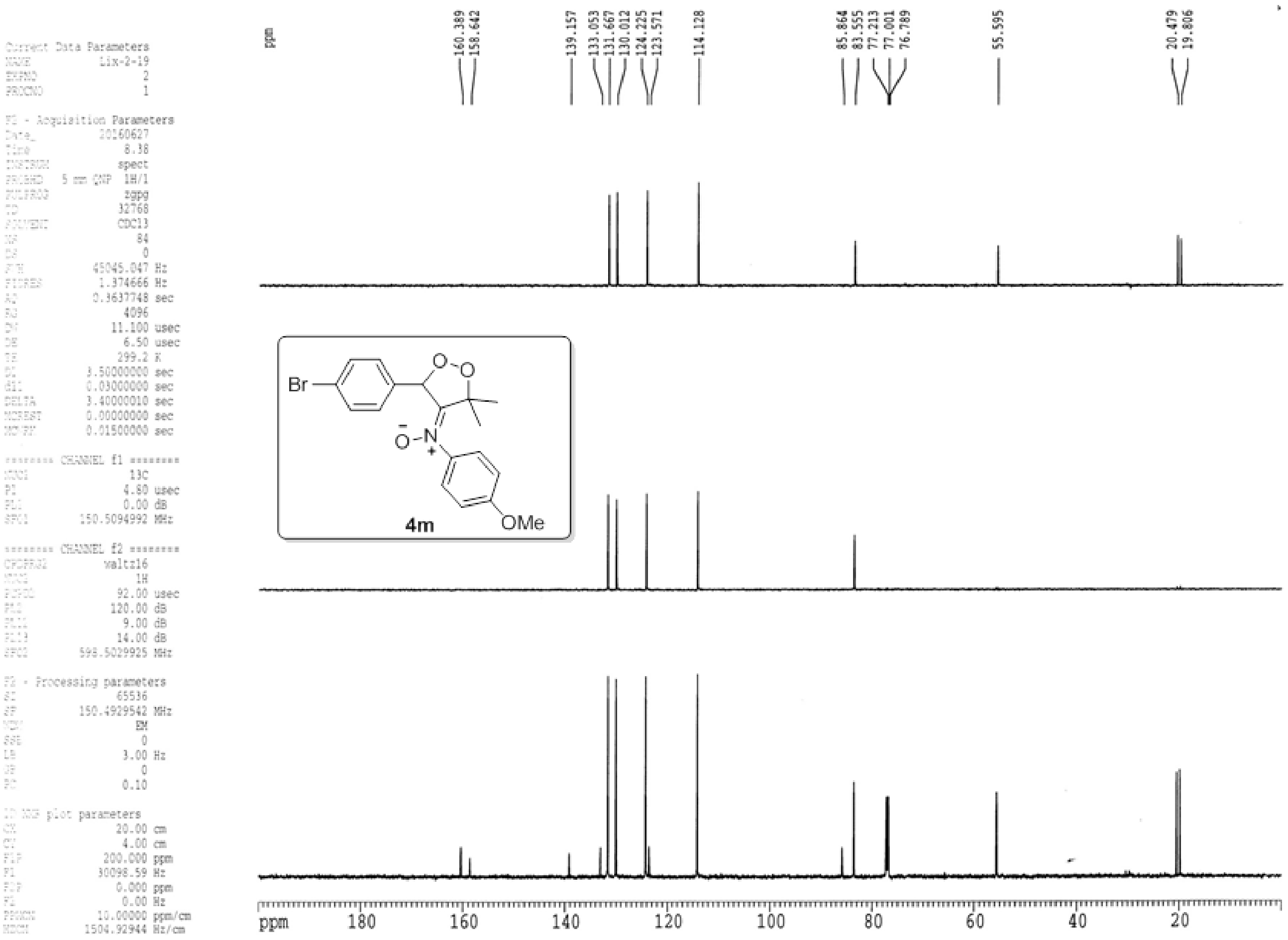
Current Data Parameters
F2 - Acquisition Parameters
CHANNEL f1
CHANNEL f2
F2 - Processing parameters
1D NMR plot parameters
160.389
158.642
139.157
133.053
131.667
130.012
124.225
123.571
114.128
85.864
83.555
77.213
77.001
76.789
55.595
20.479
19.806
Br
O
O
N
OMe
4m
ppm
180
160
140
120
100
80
60
40
20

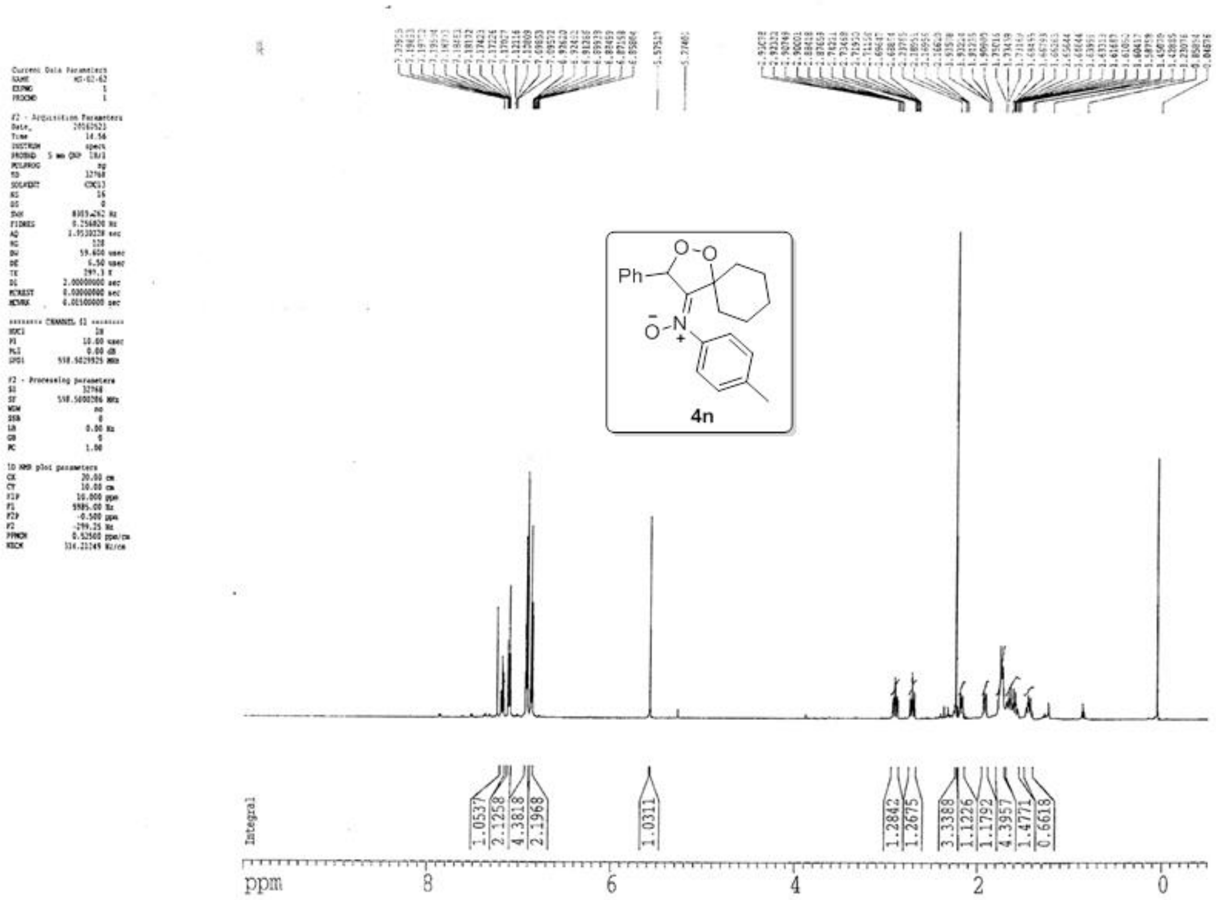

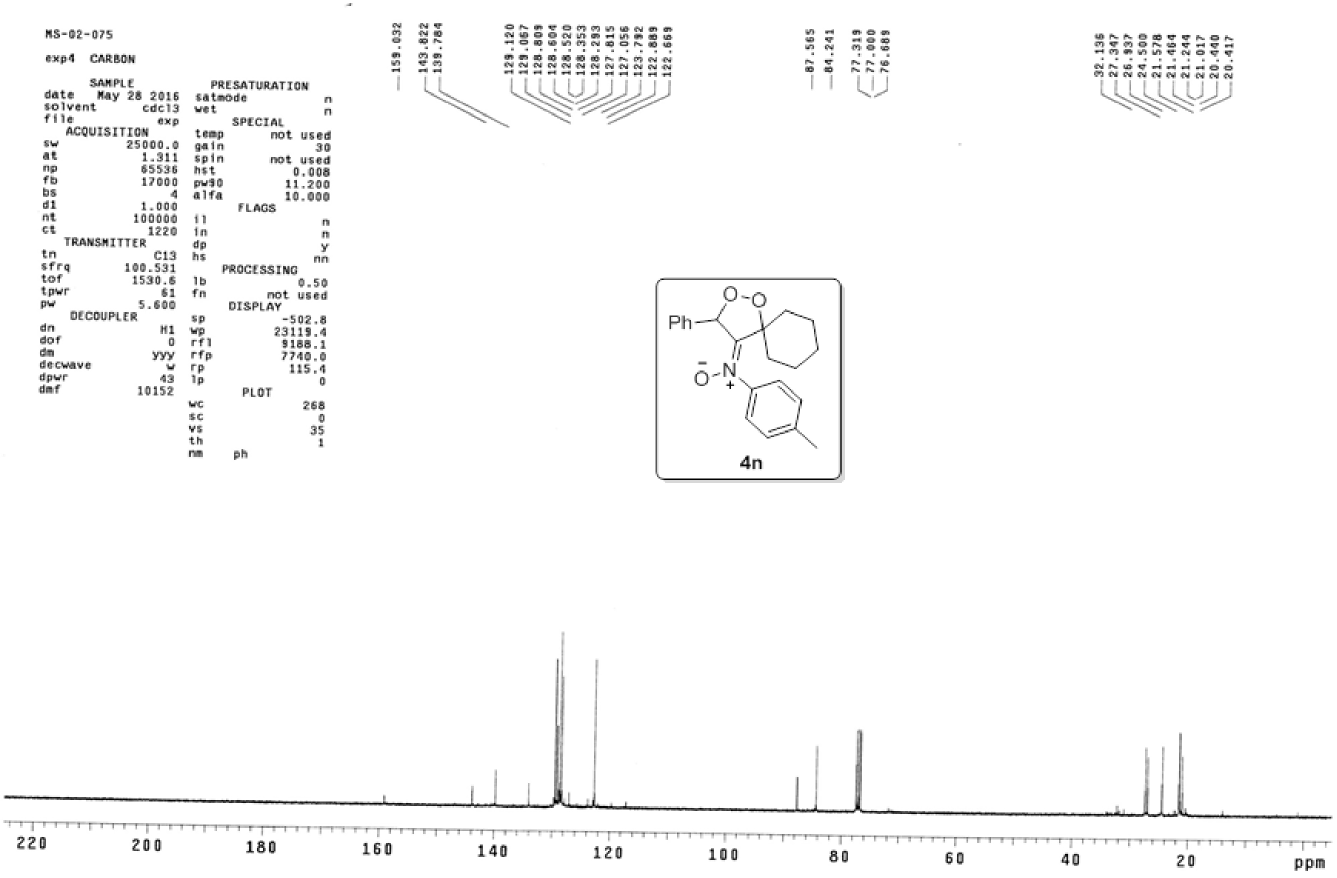
MS-02-075
exp4 CARBON
SAMPLE
date May 28 2016
solvent cdcl3
file exp
ACQUISITION
sw 25000.0
at 1.311
np 65536
fb 17000
bs 4
d1 1.000
nt 100000
ct 1220
TRANSMITTER
tn C13
sfrq 100.531
tof 1530.6
tpwr 61
pw 5.600
DECOUPLER
dn H1
dof 0
dm yyy
decwave w
dpwr 43
dmf 10152
PRESATURATION
satmode n
wet n
SPECIAL
temp not used
gain 30
spin not used
hst 0.008
pw90 11.200
alfa 10.000
FLAGS
il n
in n
dp y
hs nn
PROCESSING
lb 0.50
fn not used
DISPLAY
sp -502.8
wp 23119.4
rfl 9188.1
rfp 7740.0
rp 115.4
lp 0
PLOT
wc 268
sc 0
vs 35
th 1
nm ph
159.032
143.822
139.784
129.120
129.067
128.809
128.604
128.520
128.353
128.293
127.815
127.056
123.792
122.889
122.669
87.565
84.241
77.319
77.000
76.689
32.136
27.347
26.937
24.500
21.578
21.464
21.244
21.017
20.440
20.417
Ph
O
O
N
4n
220
200
180
160
140
120
100
80
60
40
20
ppm

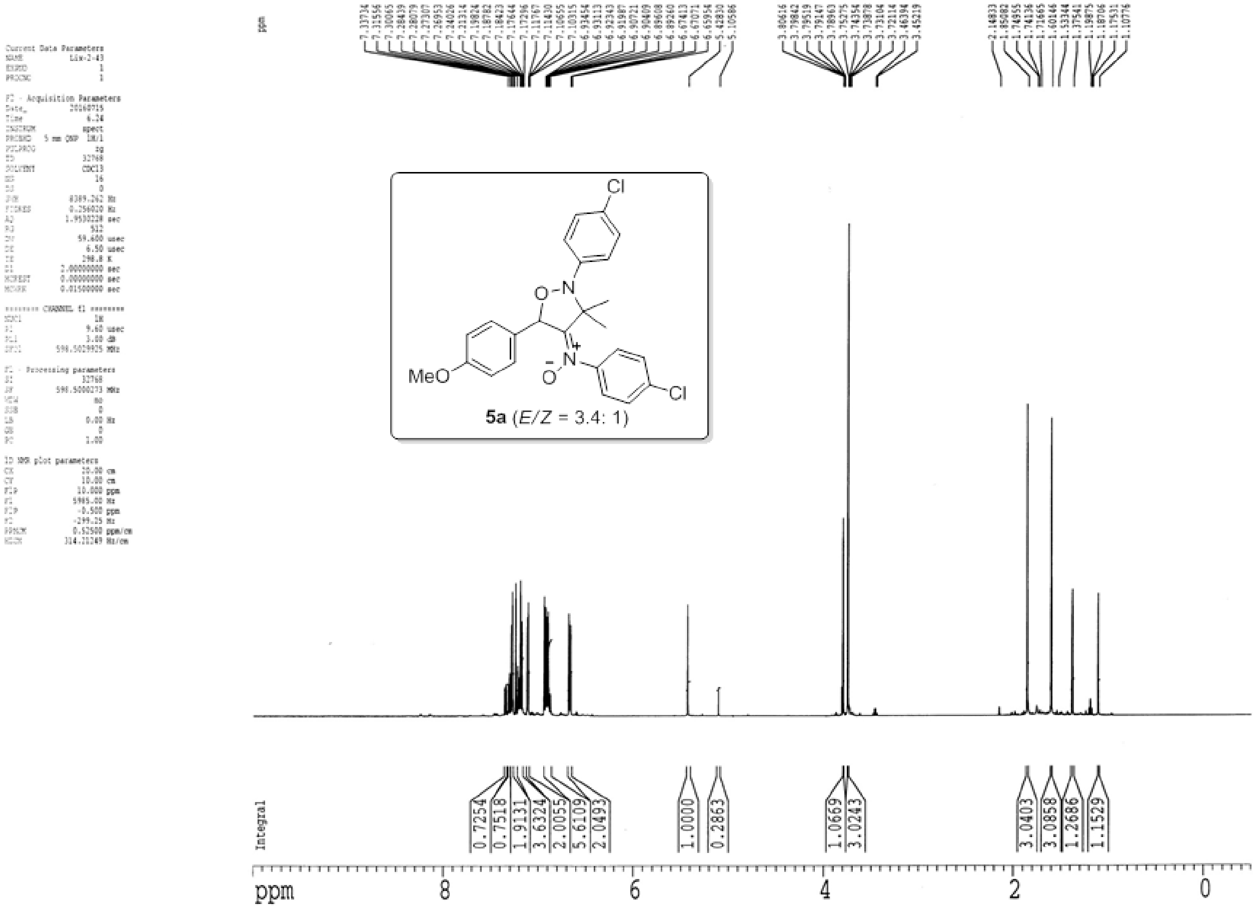
Cl
O
N
MeO
N+
O-
Cl
5a (E/Z = 3.4: 1)
Integral
0.7254
0.7518
1.9131
3.6324
2.0055
5.6109
2.0493
1.0000
0.2863
1.0669
3.0243
3.0403
3.0858
1.2686
1.1529
ppm
8
6
4
2
0

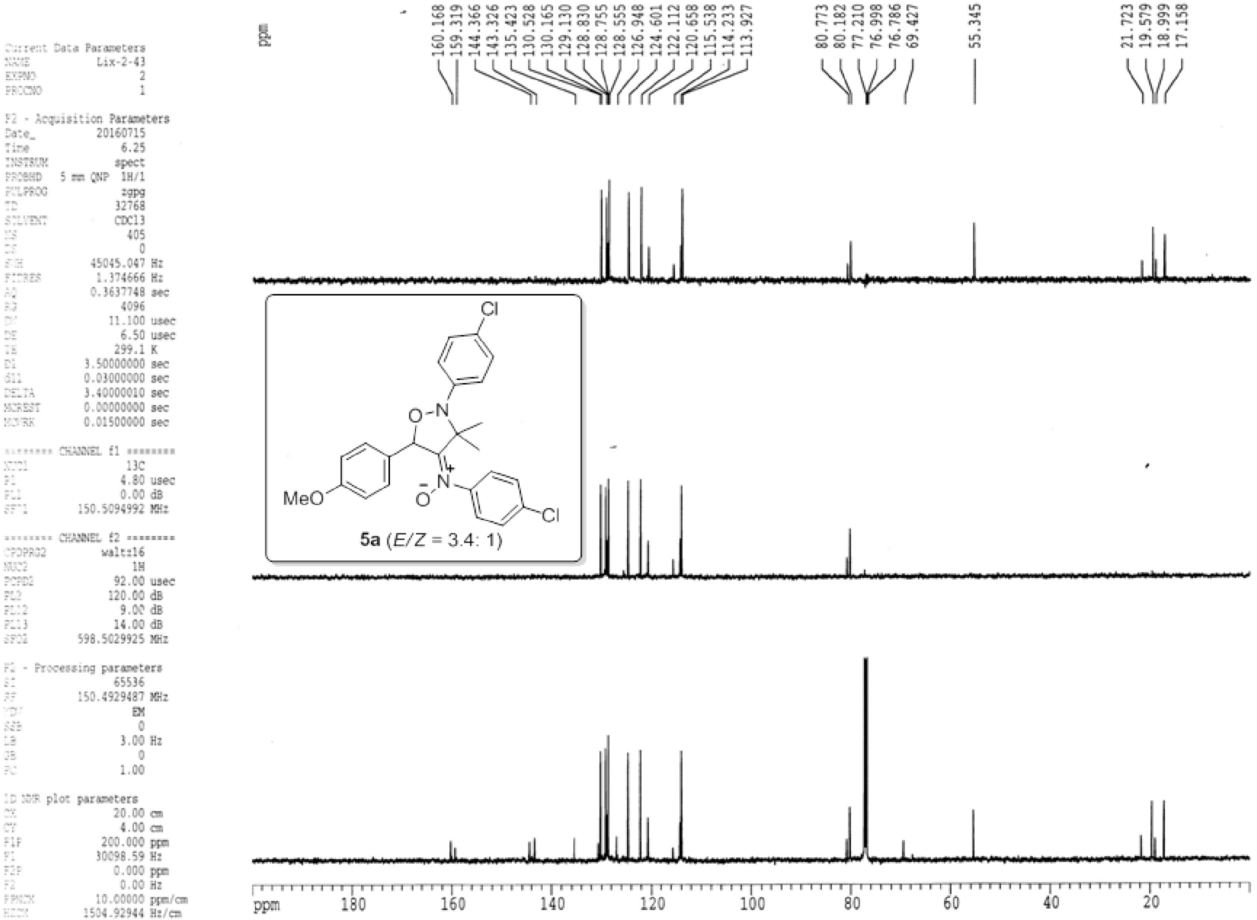
Current Data Parameters
NAME Lix-2-43
EXPNO 2
PROCNO 1
F2 - Acquisition Parameters
Date_ 20160715
Time 6.25
INSTRUM spect
PROBHD 5 mm QNP 1H/1
PULPROG zgpg
TD 32768
SOLVENT CDCl3
NS 405
DS 0
SWH 45045.047 Hz
FIDRES 1.374666 Hz
AQ 0.3637748 sec
RG 4096
DW 11.100 usec
DE 6.50 usec
TE 299.1 K
D1 3.50000000 sec
d11 0.03000000 sec
DELTA 3.40000010 sec
MCREST 0.00000000 sec
MCWRK 0.01500000 sec
======== CHANNEL f1 ========
NUC1 13C
P1 4.80 usec
PL1 0.00 dB
SFO1 150.5094992 MHz
======== CHANNEL f2 ========
CPDPRG2 waltz16
NUC2 1H
PCPD2 92.00 usec
PL2 120.00 dB
PL12 9.00 dB
PL13 14.00 dB
SFO2 598.5029925 MHz
F2 - Processing parameters
SI 65536
SF 150.4929487 MHz
WDW EM
SSB 0
LB 3.00 Hz
GB 0
PC 1.00
1D NMR plot parameters
CX 20.00 cm
CY 4.00 cm
F1P 200.000 ppm
F1 30098.59 Hz
F2P 0.000 ppm
F2 0.00 Hz
PPMCM 10.00000 ppm/cm
HZCM 1504.92944 Hz/cm
ppm
160.168
159.319
144.366
143.326
135.423
130.528
130.165
129.130
128.830
128.755
128.555
126.948
124.601
122.112
120.658
115.538
114.233
113.927
80.773
80.182
77.210
76.998
76.786
69.427
55.345
21.723
19.579
18.999
17.158
Cl
O-N
MeO
N
O
Cl
5a (E/Z = 3.4: 1)
ppm
180
160
140
120
100
80
60
40
20

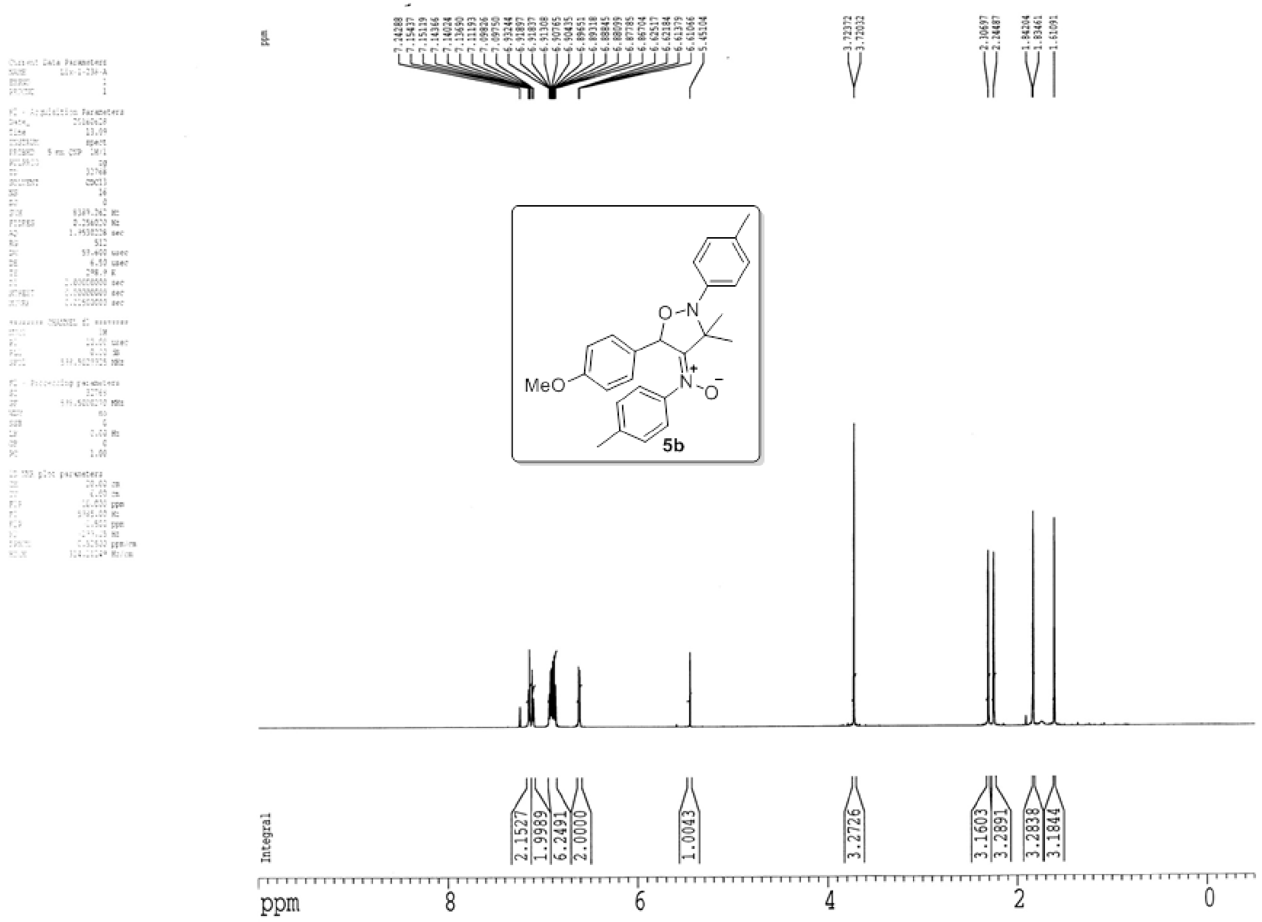
MeO
5b
Integral
2.1527
1.9989
6.2491
2.0000
1.0043
3.2726
3.1603
3.2891
3.2838
3.1844
ppm
8
6
4
2
0

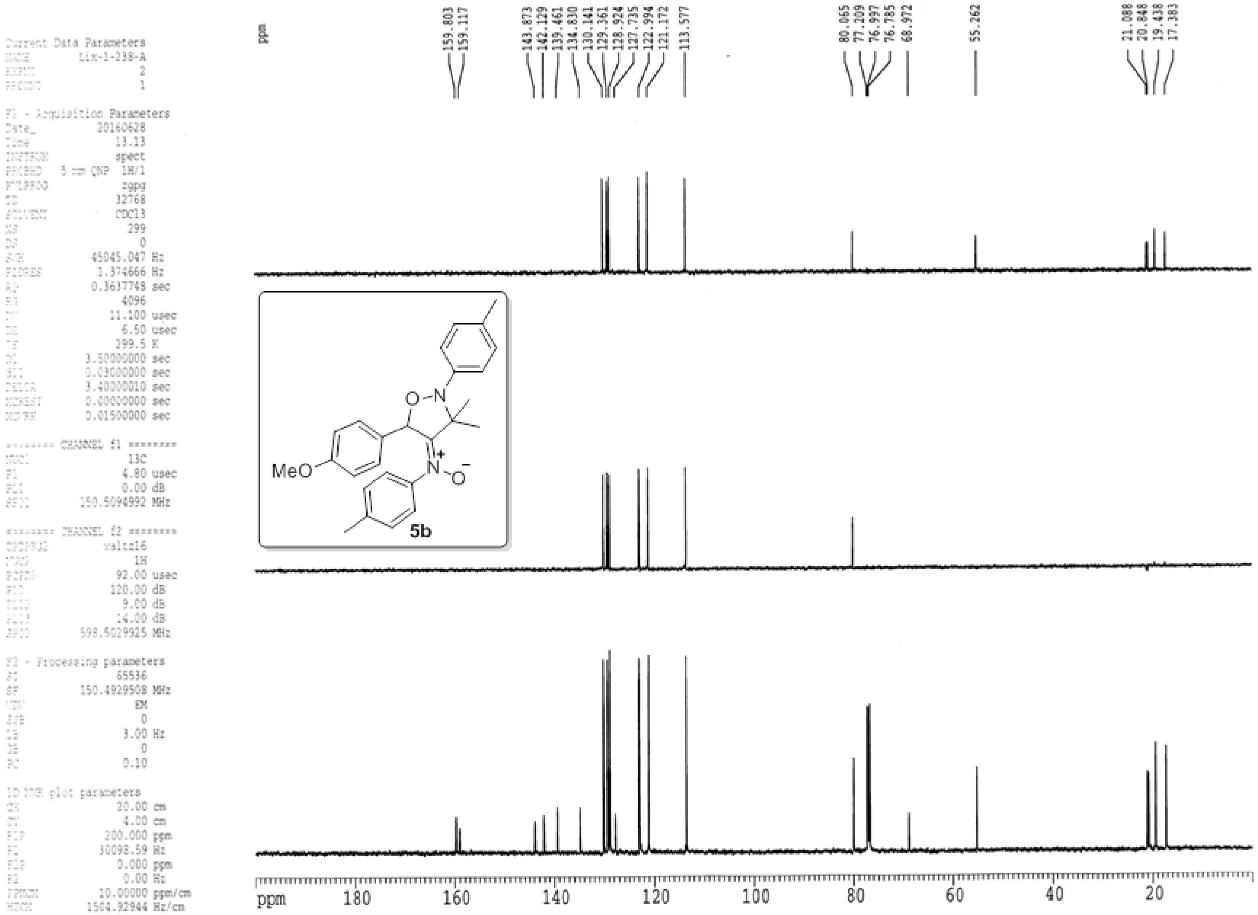
Current Data Parameters
Lix-1-238-A
F2 - Acquisition Parameters
20160628
13.13
spect
5 mm QNP 1H/1
zgpg
32768
CDCl3
299
45045.047 Hz
1.374666 Hz
0.3637748 sec
11.100 usec
6.50 usec
299.5 K
3.50000000 sec
CHANNEL f1
13C
4.80 usec
0.00 dB
150.5094992 MHz
CHANNEL f2
waltz16
1H
92.00 usec
120.00 dB
9.00 dB
14.00 dB
598.5029925 MHz
F2 - Processing parameters
65536
150.4929508 MHz
EM
3.00 Hz
1D NMR plot parameters
20.00 cm
4.00 cm
200.000 ppm
30098.59 Hz
0.000 ppm
0.00 Hz
10.00000 ppm/cm
1504.92944 Hz/cm
159.803
159.117
143.873
142.129
139.461
134.830
130.141
129.361
128.924
127.735
122.994
121.172
113.577
80.065
77.209
76.997
76.785
68.972
55.262
21.088
20.848
19.438
17.383
MeO
O
N
N
+
O
-
5b
ppm
180
160
140
120
100
80
60
40
20

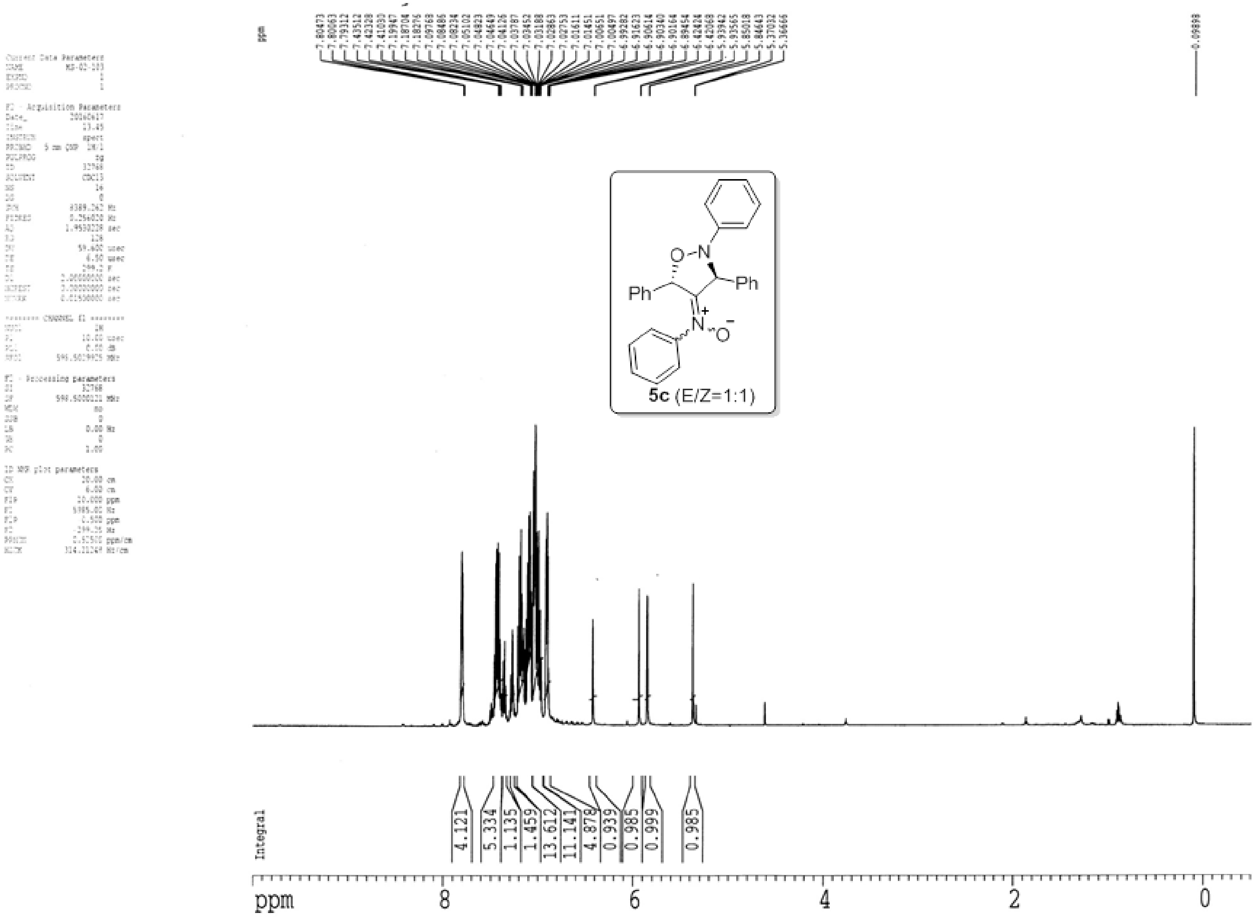

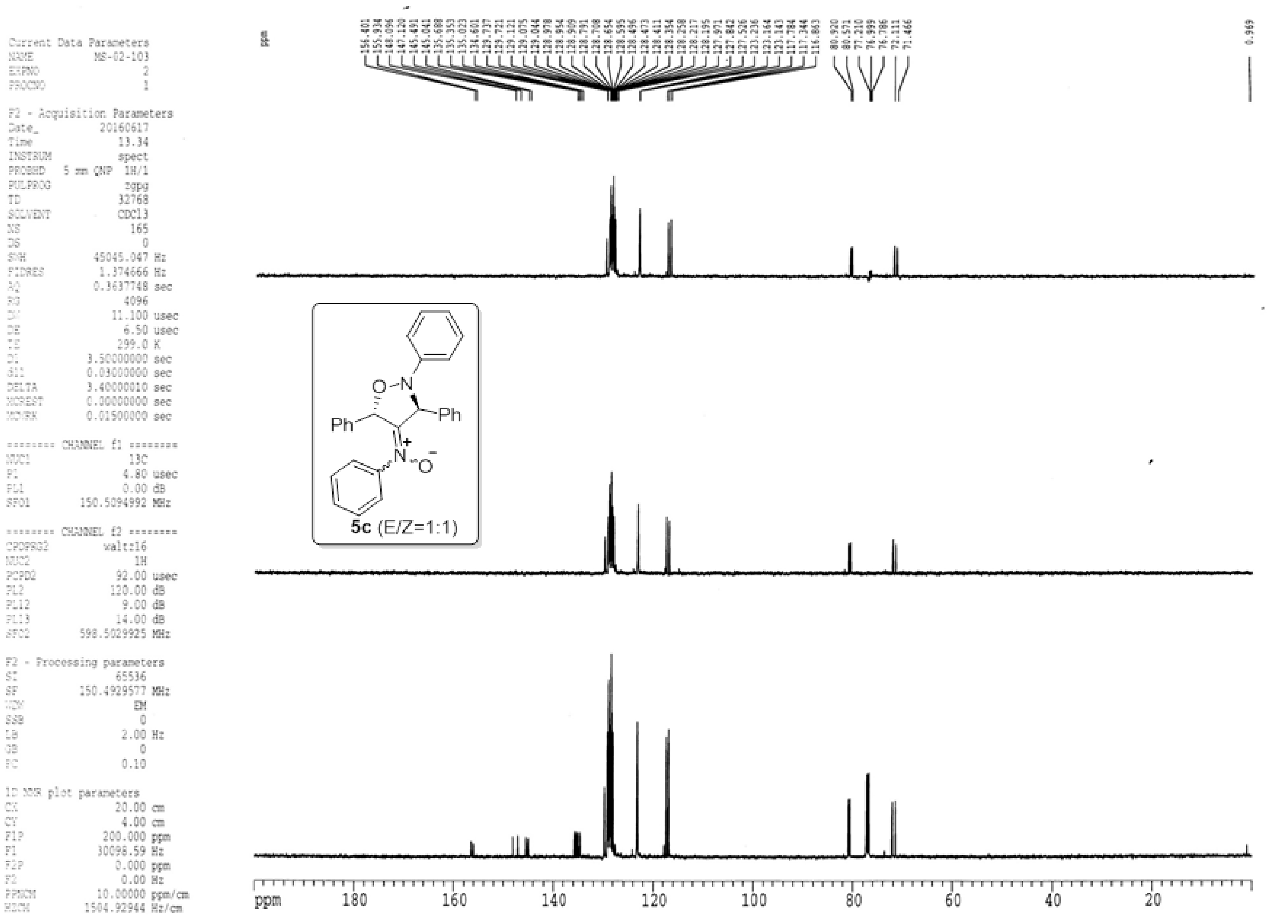

Current Data Parameters
NAME MS-02-103
EXPNO 2
PROCNO 1
F2 - Acquisition Parameters
Date_ 20160617
Time 13.34
INSTRUM spect
PROBHD 5 mm QNP 1H/1
PULPROG zgpg
TD 32768
SOLVENT CDCl3
NS 165
DS 0
SWH 45045.047 Hz
FIDRES 1.374666 Hz
AQ 0.3637748 sec
RG 4096
DW 11.100 usec
DE 6.50 usec
TE 299.0 K
D1 3.50000000 sec
d11 0.03000000 sec
DELTA 3.40000010 sec
MCREST 0.00000000 sec
MCWRK 0.01500000 sec
======== CHANNEL f1 ========
NUC1 13C
P1 4.80 usec
PL1 0.00 dB
SFO1 150.5094992 MHz
======== CHANNEL f2 ========
CPDPRG2 waltz16
NUC2 1H
PCPD2 92.00 usec
PL2 120.00 dB
PL12 9.00 dB
PL13 14.00 dB
SFO2 598.5029925 MHz
F2 - Processing parameters
SI 65536
SF 150.4929577 MHz
WDW EM
SSB 0
LB 2.00 Hz
GB 0
PC 0.10
1D NMR plot parameters
CX 20.00 cm
CY 4.00 cm
F1P 200.000 ppm
F1 30098.59 Hz
F2P 0.000 ppm
F2 0.00 Hz
PPMCM 10.00000 ppm/cm
HZCM 1504.92944 Hz/cm
ppm
156.401
155.934
148.096
147.120
145.491
145.041
135.688
135.353
135.023
134.601
129.737
129.721
129.121
129.075
129.044
128.978
128.954
128.909
128.791
128.708
128.654
128.595
128.496
128.473
128.411
128.354
128.258
128.217
128.195
127.971
127.842
127.526
123.236
123.164
123.143
117.784
117.344
116.863
80.920
80.571
77.210
76.999
76.786
72.111
71.466
0.969
O–N
Ph
Ph
N+
O−
5c (E/Z=1:1)
ppm 180 160 140 120 100 80 60 40 20

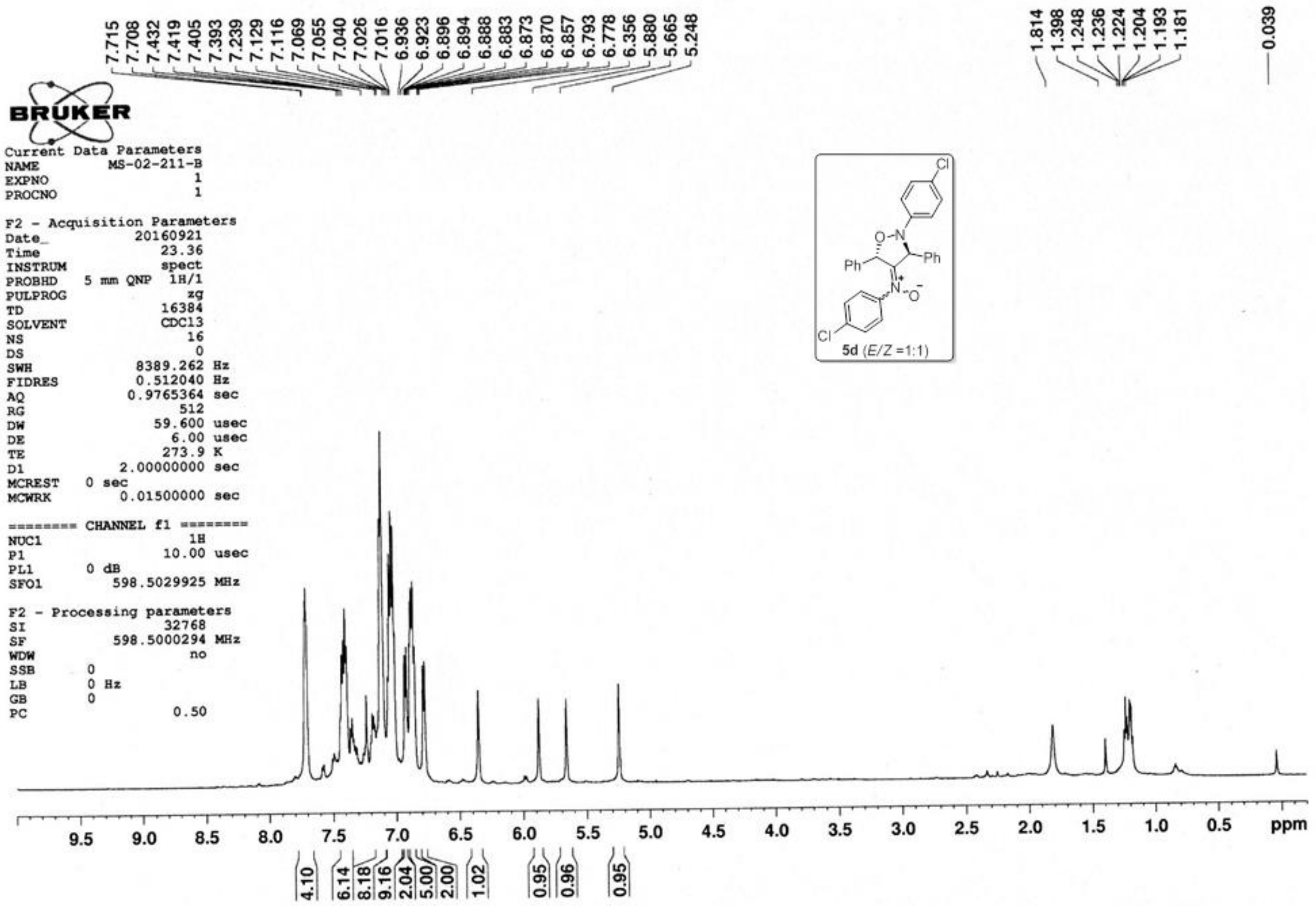
7.715 7.708 7.432 7.419 7.405 7.393 7.239 7.129 7.116 7.069 7.055 7.040 7.026 7.016 6.936 6.923 6.896 6.894 6.888 6.883 6.873 6.870 6.857 6.793 6.778 6.356 5.880 5.665 5.248
1.814 1.398 1.248 1.236 1.224 1.204 1.193 1.181
0.039
BRUKER
Current Data Parameters
NAME MS-02-211-B
EXPNO 1
PROCNO 1
F2 - Acquisition Parameters
Date_ 20160921
Time 23.36
INSTRUM spect
PROBHD 5 mm QNP 1H/1
PULPROG zg
TD 16384
SOLVENT CDCl3
NS 16
DS 0
SWH 8389.262 Hz
FIDRES 0.512040 Hz
AQ 0.9765364 sec
RG 512
DW 59.600 usec
DE 6.00 usec
TE 273.9 K
D1 2.00000000 sec
MCREST 0 sec
MCWRK 0.01500000 sec
======== CHANNEL f1 ========
NUC1 1H
P1 10.00 usec
PL1 0 dB
SFO1 598.5029925 MHz
F2 - Processing parameters
SI 32768
SF 598.5000294 MHz
WDW no
SSB 0
LB 0 Hz
GB 0
PC 0.50
Cl
O-N
Ph
Ph
N+
O-
Cl
5d (E/Z =1:1)
9.5 9.0 8.5 8.0 7.5 7.0 6.5 6.0 5.5 5.0 4.5 4.0 3.5 3.0 2.5 2.0 1.5 1.0 0.5 ppm
4.10 6.14 8.18 9.16 2.04 5.00 2.00 1.02 0.95 0.96 0.95

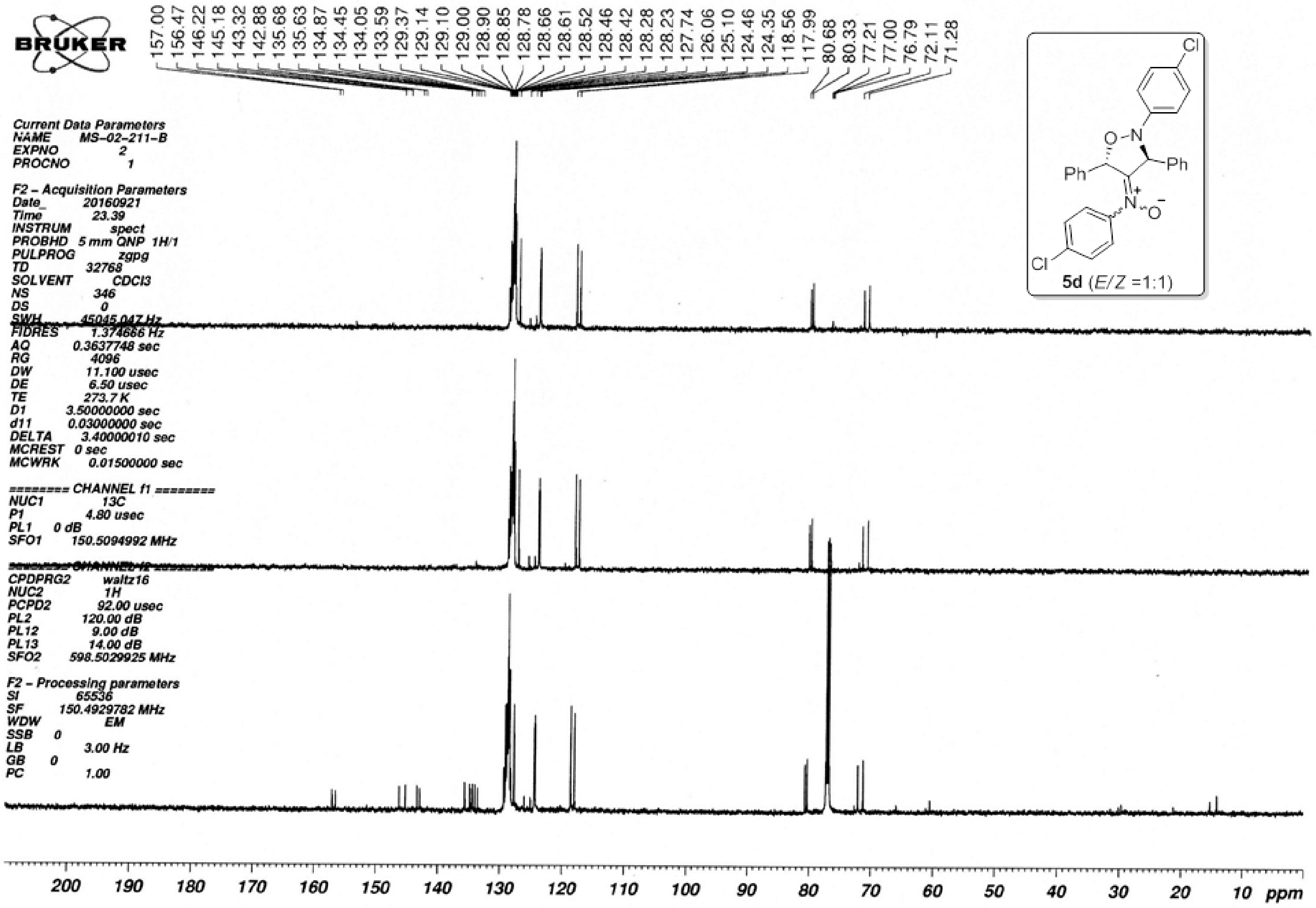
BRUKER
157.00
156.47
146.22
145.18
143.32
142.88
135.68
135.63
134.87
134.45
134.05
133.59
129.37
129.14
129.10
129.00
128.90
128.85
128.78
128.66
128.61
128.52
128.46
128.42
128.28
128.23
127.74
126.06
125.10
124.46
124.35
118.56
117.99
80.68
80.33
77.21
77.00
76.79
72.11
71.28
Current Data Parameters
NAME MS-02-211-B
EXPNO 2
PROCNO 1
F2 - Acquisition Parameters
Date_ 20160921
Time 23.39
INSTRUM spect
PROBHD 5 mm QNP 1H/1
PULPROG zgpg
TD 32768
SOLVENT CDCl3
NS 346
DS 0
SWH 45045.047 Hz
FIDRES 1.374666 Hz
AQ 0.3637748 sec
RG 4096
DW 11.100 usec
DE 6.50 usec
TE 273.7 K
D1 3.50000000 sec
d11 0.03000000 sec
DELTA 3.40000010 sec
MCREST 0 sec
MCWRK 0.01500000 sec
======== CHANNEL f1 ========
NUC1 13C
P1 4.80 usec
PL1 0 dB
SFO1 150.5094992 MHz
======== CHANNEL f2 ========
CPDPRG2 waltz16
NUC2 1H
PCPD2 92.00 usec
PL2 120.00 dB
PL12 9.00 dB
PL13 14.00 dB
SFO2 598.5029925 MHz
F2 - Processing parameters
SI 65536
SF 150.4929782 MHz
WDW EM
SSB 0
LB 3.00 Hz
GB 0
PC 1.00
200 190 180 170 160 150 140 130 120 110 100 90 80 70 60 50 40 30 20 10 ppm
Cl
O
N
Ph
Ph
N
O
Cl
5d (E/Z =1:1)

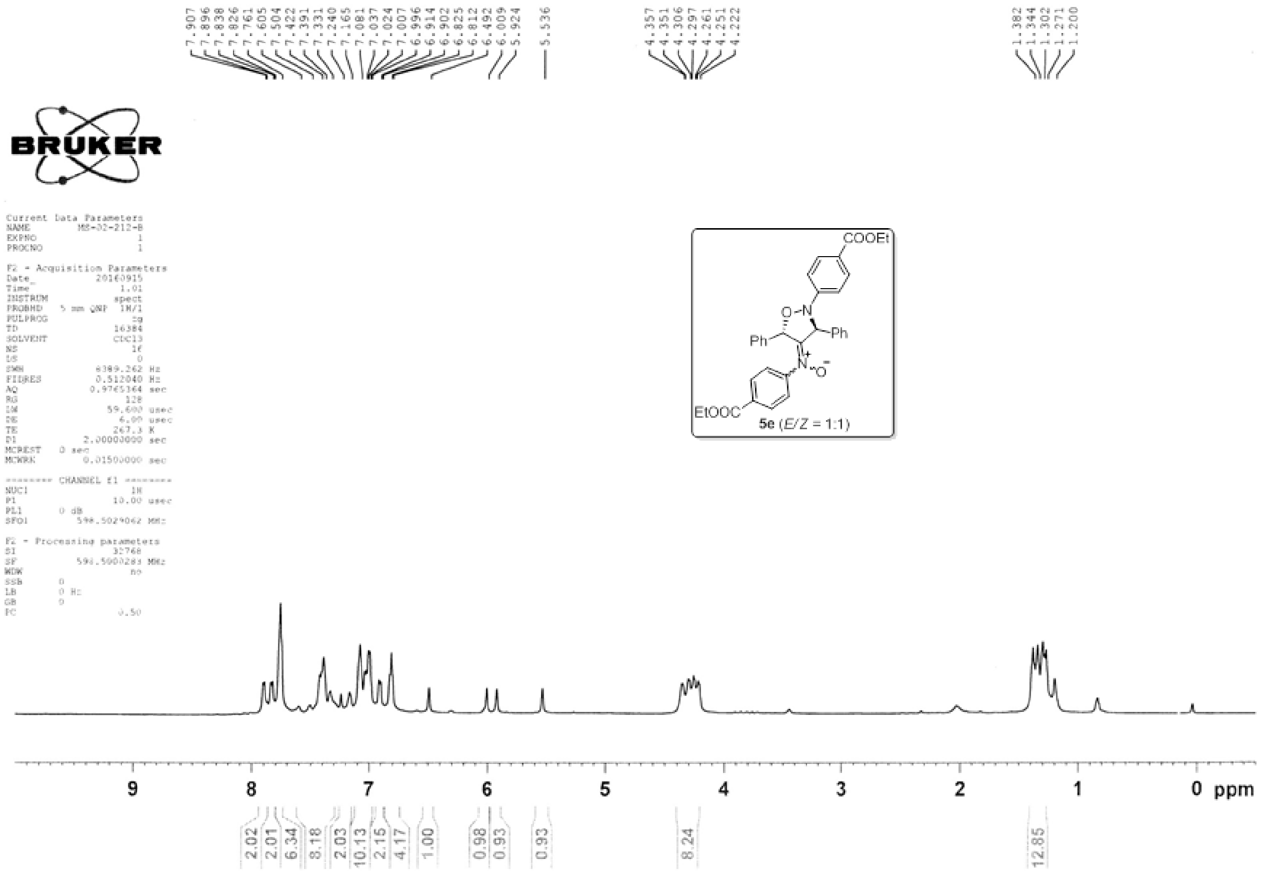

BRUKER
COOEt
O–N
Ph
Ph
EtOOC
5e (E/Z = 1:1)
9
8
7
6
5
4
3
2
1
0 ppm

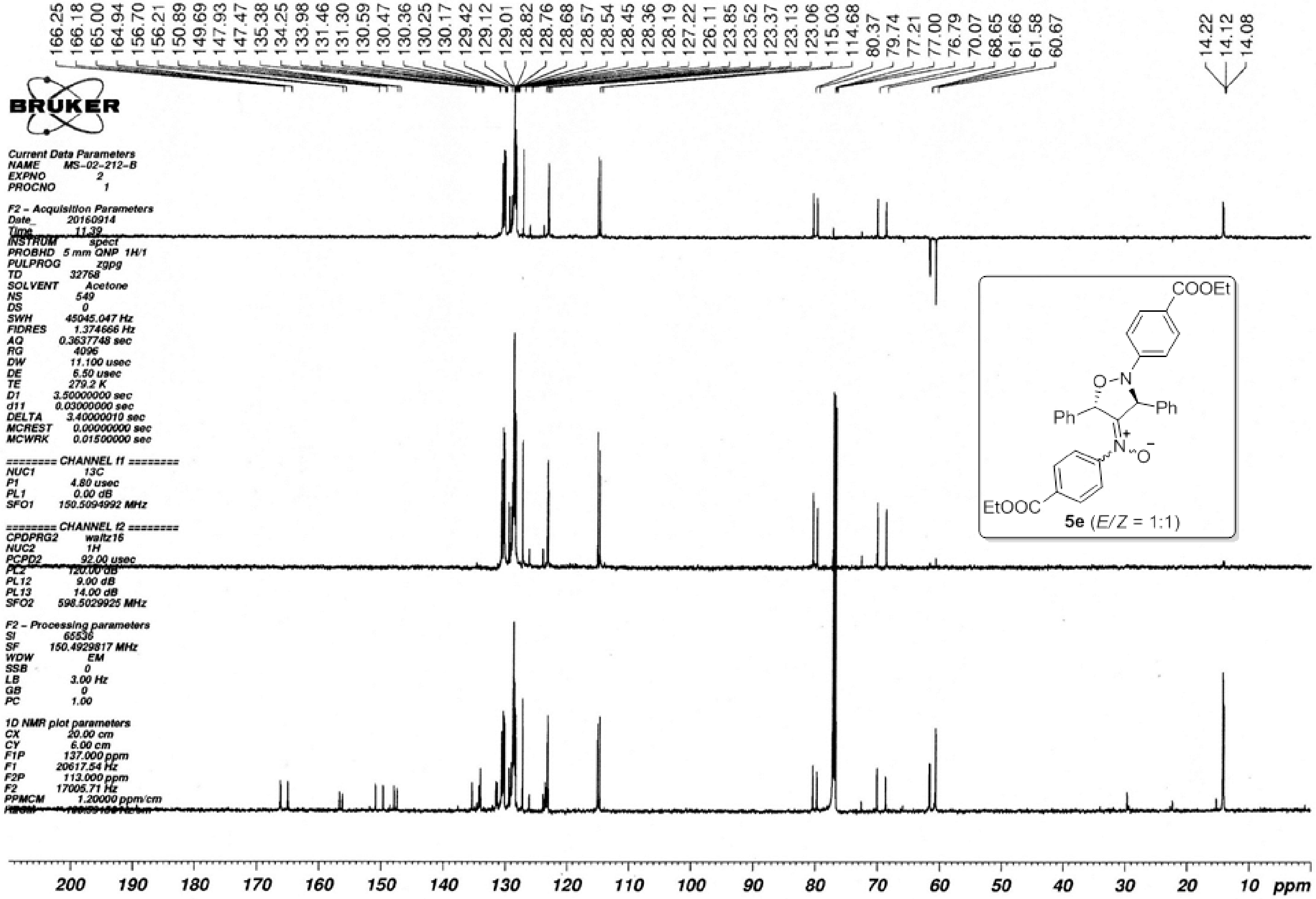
166.25
166.18
165.00
164.94
156.70
156.21
150.89
149.69
147.93
147.47
135.38
134.25
133.98
131.46
131.30
130.59
130.47
130.36
130.25
130.17
129.42
129.12
129.01
128.82
128.76
128.68
128.57
128.54
128.45
128.36
128.19
127.22
126.11
123.85
123.52
123.37
123.13
123.06
115.03
114.68
80.37
79.74
77.21
77.00
76.79
70.07
68.65
61.66
61.58
60.67
14.22
14.12
14.08
BRUKER
Current Data Parameters
NAME MS-02-212-B
EXPNO 2
PROCNO 1
F2 - Acquisition Parameters
Date_ 20160914
Time 11.39
INSTRUM spect
PROBHD 5 mm QNP 1H/1
PULPROG zgpg
TD 32768
SOLVENT Acetone
NS 549
DS 0
SWH 45045.047 Hz
FIDRES 1.374666 Hz
AQ 0.3637748 sec
RG 4096
DW 11.100 usec
DE 6.50 usec
TE 279.2 K
D1 3.50000000 sec
d11 0.03000000 sec
DELTA 3.40000010 sec
MCREST 0.00000000 sec
MCWRK 0.01500000 sec
======== CHANNEL f1 ========
NUC1 13C
P1 4.80 usec
PL1 0.00 dB
SFO1 150.5094992 MHz
======== CHANNEL f2 ========
CPDPRG2 waltz16
NUC2 1H
PCPD2 92.00 usec
PL2 120.00 dB
PL12 9.00 dB
PL13 14.00 dB
SFO2 598.5029925 MHz
F2 - Processing parameters
SI 65536
SF 150.4929817 MHz
WDW EM
SSB 0
LB 3.00 Hz
GB 0
PC 1.00
1D NMR plot parameters
CX 20.00 cm
CY 6.00 cm
F1P 137.000 ppm
F1 20617.54 Hz
F2P 113.000 ppm
F2 17005.71 Hz
PPMCM 1.20000 ppm/cm
COOEt
O
N
Ph
Ph
N
O
EtOOC
5e (E/Z = 1:1)
200 190 180 170 160 150 140 130 120 110 100 90 80 70 60 50 40 30 20 10 ppm

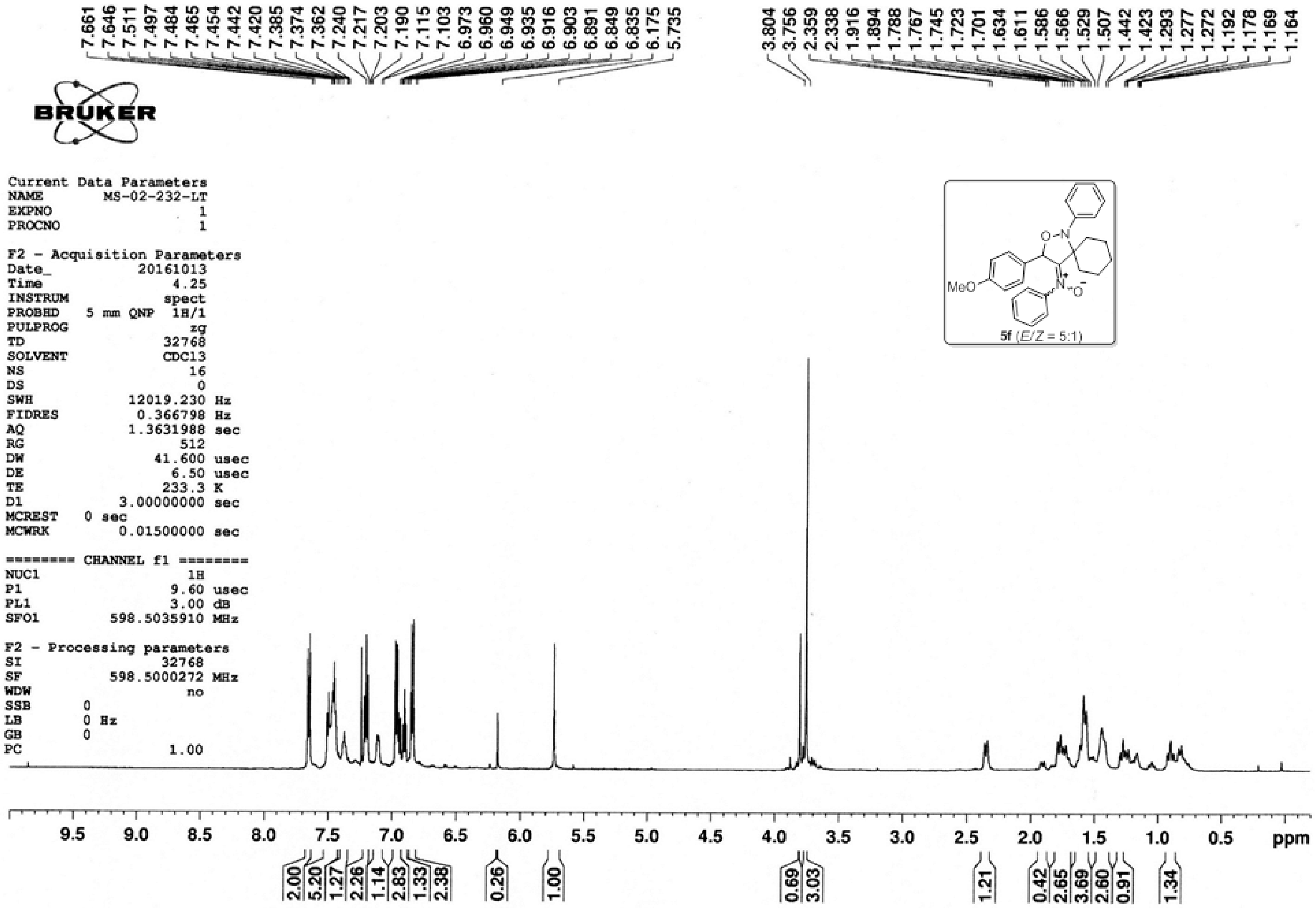
7.661 7.646 7.511 7.497 7.484 7.465 7.454 7.442 7.420 7.385 7.374 7.362 7.240 7.217 7.203 7.190 7.115 7.103 6.973 6.960 6.949 6.935 6.916 6.903 6.891 6.849 6.835 6.175 5.735
3.804 3.756 2.359 2.338 1.916 1.894 1.788 1.767 1.745 1.723 1.701 1.634 1.611 1.586 1.566 1.529 1.507 1.442 1.423 1.293 1.277 1.272 1.192 1.178 1.169 1.164
BRUKER
Current Data Parameters
NAME MS-02-232-LT
EXPNO 1
PROCNO 1
F2 - Acquisition Parameters
Date_ 20161013
Time 4.25
INSTRUM spect
PROBHD 5 mm QNP 1H/1
PULPROG zg
TD 32768
SOLVENT CDCl3
NS 16
DS 0
SWH 12019.230 Hz
FIDRES 0.366798 Hz
AQ 1.3631988 sec
RG 512
DW 41.600 usec
DE 6.50 usec
TE 233.3 K
D1 3.00000000 sec
MCREST 0 sec
MCWRK 0.01500000 sec
======== CHANNEL f1 ========
NUC1 1H
P1 9.60 usec
PL1 3.00 dB
SFO1 598.5035910 MHz
F2 - Processing parameters
SI 32768
SF 598.5000272 MHz
WDW no
SSB 0
LB 0 Hz
GB 0
PC 1.00
MeO
O
N
5f (E/Z = 5:1)
9.5 9.0 8.5 8.0 7.5 7.0 6.5 6.0 5.5 5.0 4.5 4.0 3.5 3.0 2.5 2.0 1.5 1.0 0.5 ppm
2.00 5.20 1.27 2.26 1.14 2.83 1.33 2.38 0.26 1.00 0.69 3.03 1.21 0.42 2.65 3.69 2.60 0.91 1.34

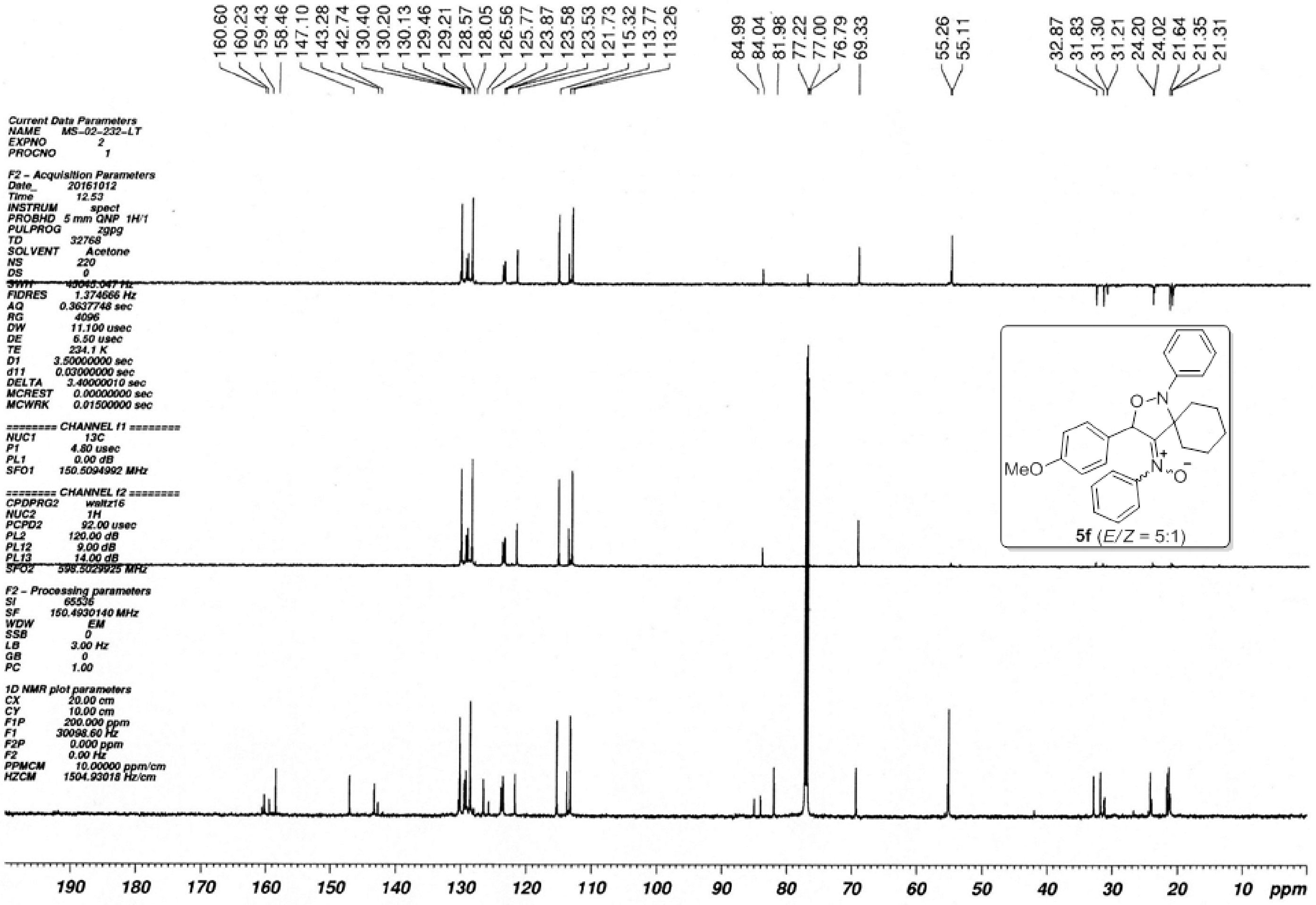
160.60 160.23 159.43 158.46 147.10 143.28 142.74 130.40 130.20 130.13 129.46 129.21 128.57 128.05 126.56 125.77 123.87 123.58 123.53 121.73 115.32 113.77 113.26
84.99 84.04 81.98 77.22 77.00 76.79 69.33
55.26 55.11
32.87 31.83 31.30 31.21 24.20 24.02 21.64 21.35 21.31
MeO
O
N
5f (E/Z = 5:1)
190 180 170 160 150 140 130 120 110 100 90 80 70 60 50 40 30 20 10 ppm

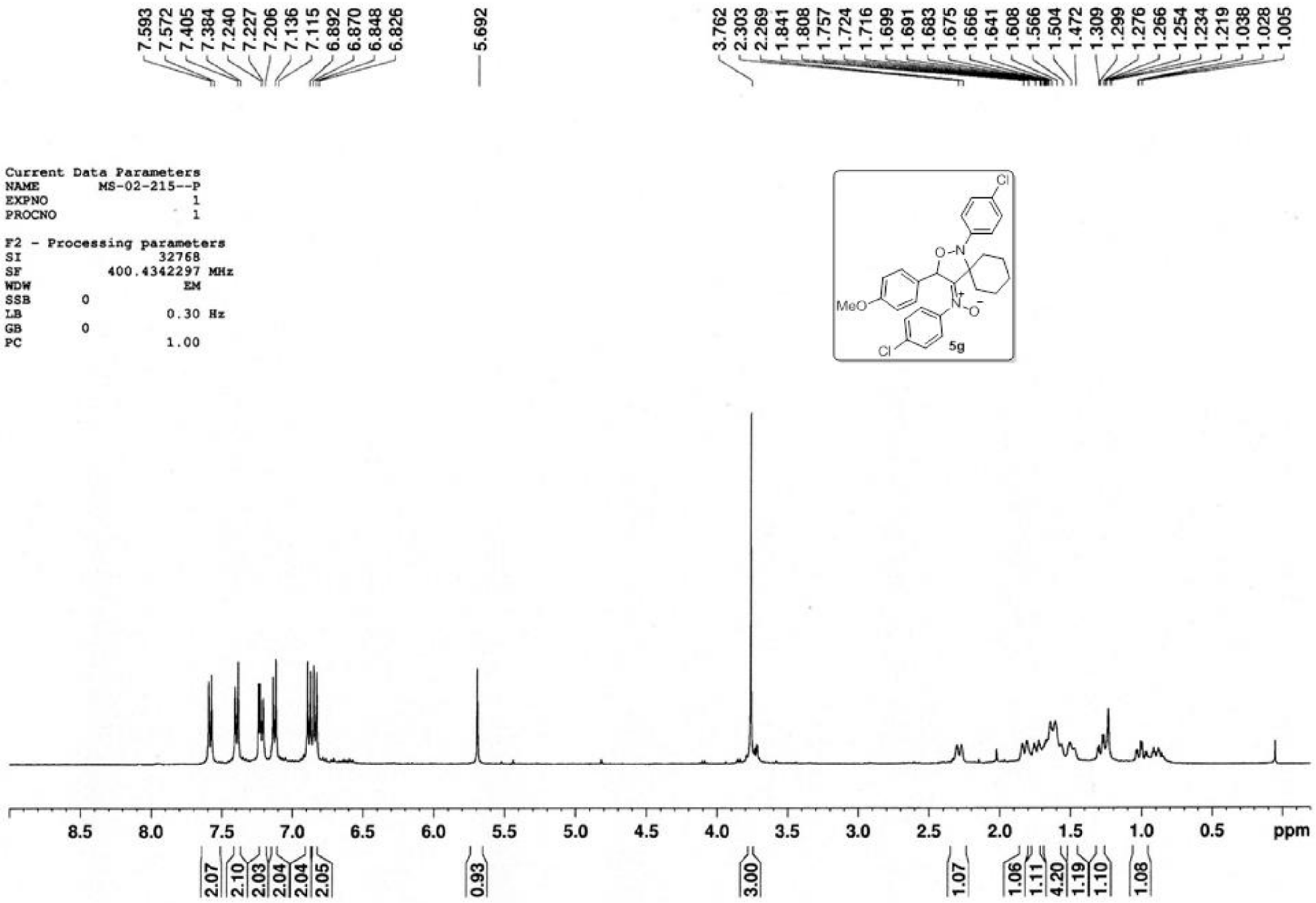
7.593
7.572
7.405
7.384
7.240
7.227
7.206
7.136
7.115
6.892
6.870
6.848
6.826
5.692
3.762
2.303
2.269
1.841
1.808
1.757
1.724
1.716
1.699
1.691
1.683
1.675
1.666
1.641
1.608
1.566
1.504
1.472
1.309
1.299
1.276
1.266
1.254
1.234
1.219
1.038
1.028
1.005
Current Data Parameters
NAME MS-02-215--P
EXPNO 1
PROCNO 1
F2 - Processing parameters
SI 32768
SF 400.4342297 MHz
WDW EM
SSB 0
LB 0.30 Hz
GB 0
PC 1.00
Cl
O
N
MeO
N+
O-
Cl
5g
8.5
8.0
7.5
7.0
6.5
6.0
5.5
5.0
4.5
4.0
3.5
3.0
2.5
2.0
1.5
1.0
0.5
ppm
2.07
2.10
2.03
2.04
2.04
2.05
0.93
3.00
1.07
1.06
1.11
4.20
1.19
1.10
1.08

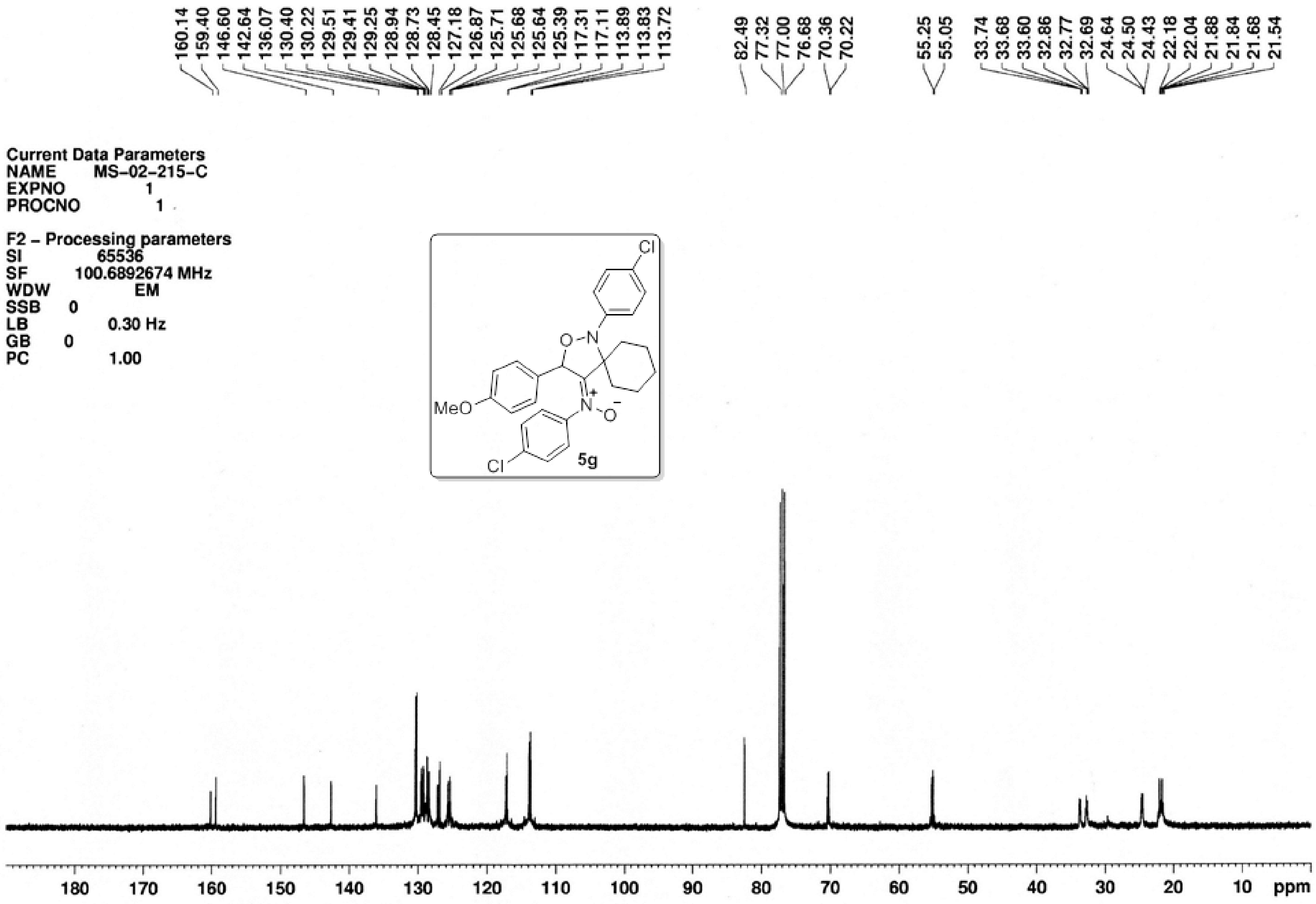
160.14
159.40
146.60
142.64
136.07
130.40
130.22
129.51
129.41
129.25
128.94
128.73
128.45
127.18
126.87
125.71
125.68
125.64
125.39
117.31
117.11
113.89
113.83
113.72
82.49
77.32
77.00
76.68
70.36
70.22
55.25
55.05
33.74
33.68
33.60
32.86
32.77
32.69
24.64
24.50
24.43
22.18
22.04
21.88
21.84
21.68
21.54
Current Data Parameters
NAME MS-02-215-C
EXPNO 1
PROCNO 1
F2 - Processing parameters
SI 65536
SF 100.6892674 MHz
WDW EM
SSB 0
LB 0.30 Hz
GB 0
PC 1.00
Cl
O-N
MeO
N+
O-
Cl
5g
180
170
160
150
140
130
120
110
100
90
80
70
60
50
40
30
20
10
ppm

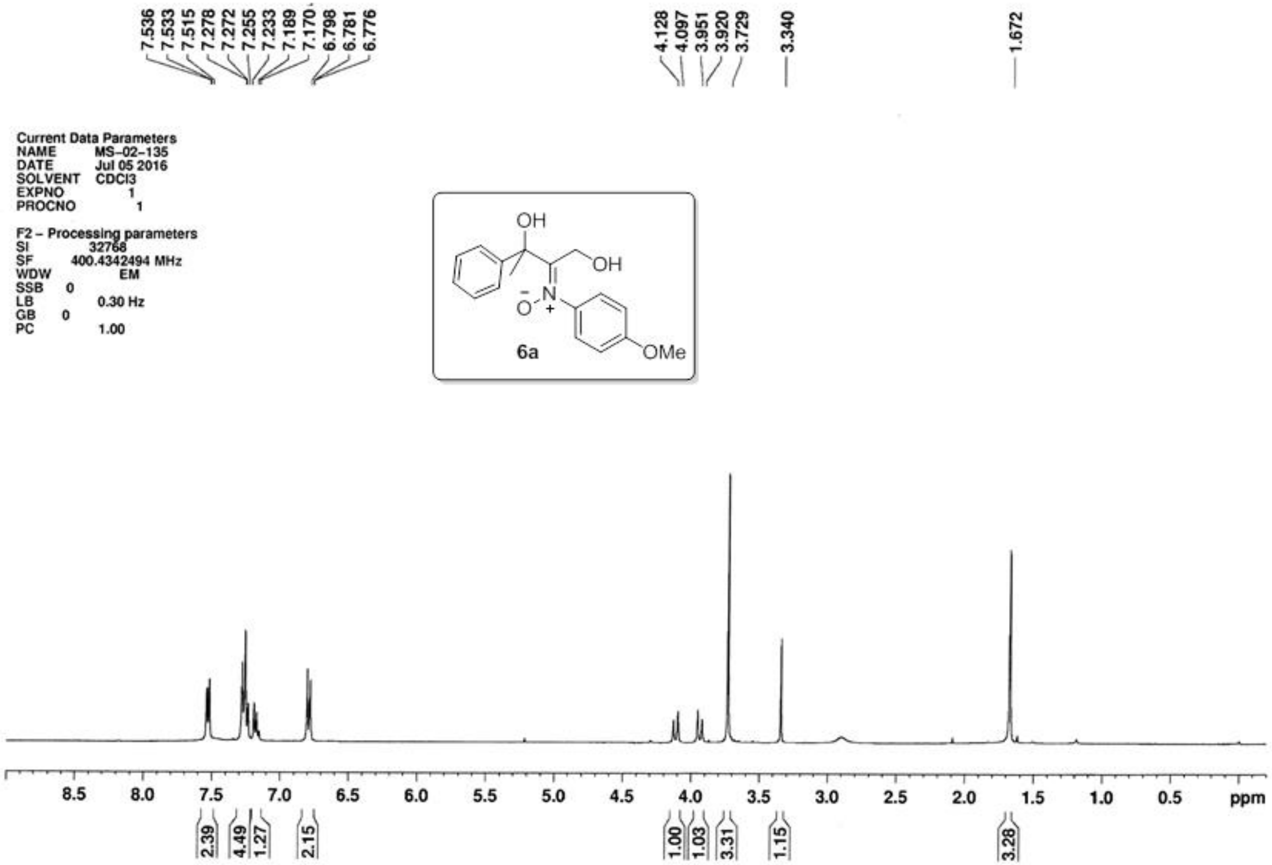
7.536
7.533
7.515
7.278
7.272
7.255
7.233
7.189
7.170
6.798
6.781
6.776
4.128
4.097
3.951
3.920
3.729
3.340
1.672
Current Data Parameters
NAME MS–02–135
DATE Jul 05 2016
SOLVENT CDCl3
EXPNO 1
PROCNO 1
F2 – Processing parameters
SI 32768
SF 400.4342494 MHz
WDW EM
SSB 0
LB 0.30 Hz
GB 0
PC 1.00
OH
OH
O
N
OMe
6a
8.5 8.0 7.5 7.0 6.5 6.0 5.5 5.0 4.5 4.0 3.5 3.0 2.5 2.0 1.5 1.0 0.5 ppm
2.39
4.49
1.27
2.15
1.00
1.03
3.31
1.15
3.28

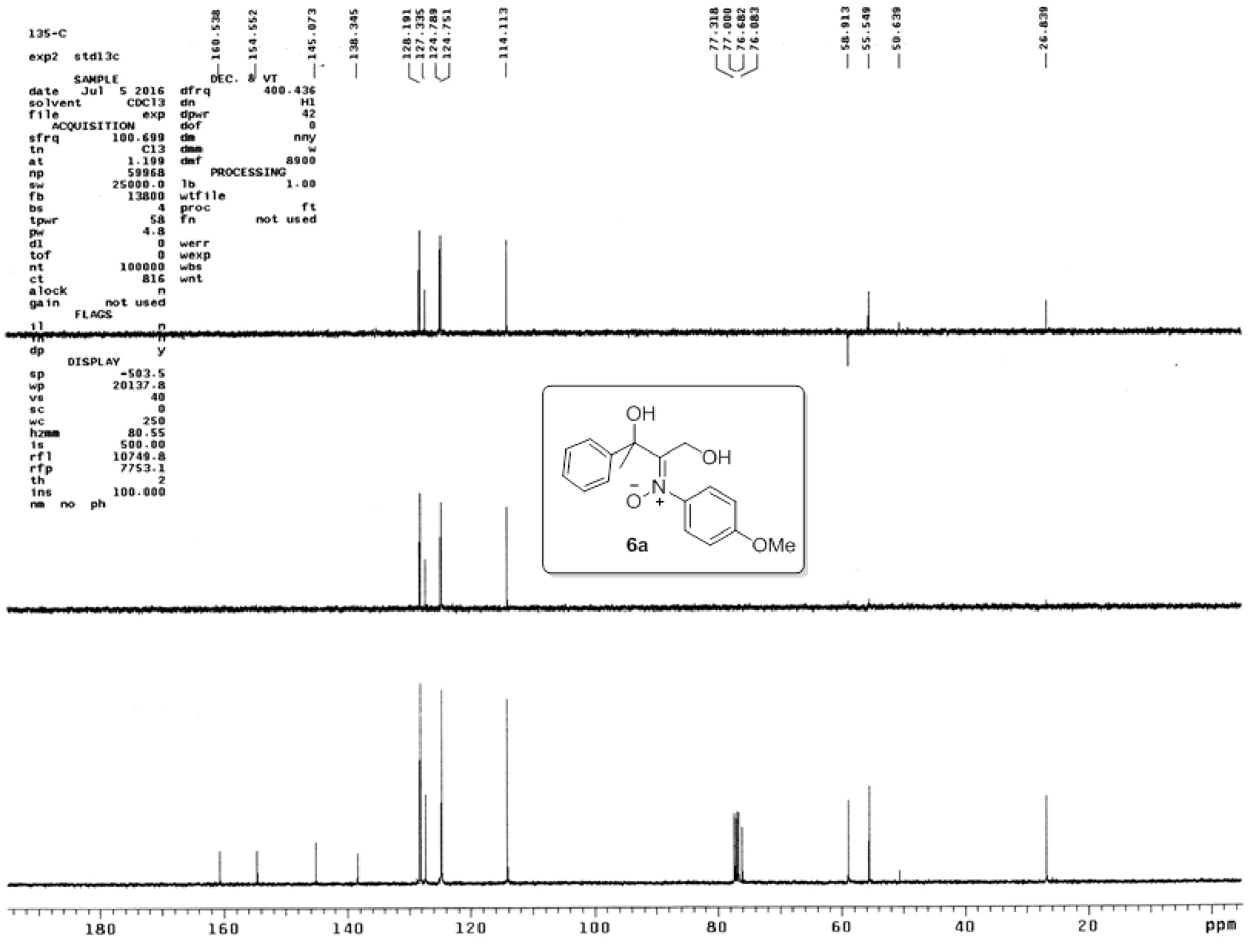

135-C
exp2 std13c
SAMPLE
date Jul 5 2016
solvent CDCl3
file exp
ACQUISITION
sfrq 100.699
tn C13
at 1.199
np 59968
sw 25000.0
fb 13800
bs 4
tpwr 58
pw 4.8
d1 0
tof 0
nt 100000
ct 816
alock n
gain not used
FLAGS
il n
in n
dp y
DISPLAY
sp -503.5
wp 20137.8
vs 40
sc 0
wc 250
hzmm 80.55
is 500.00
rfl 10749.8
rfp 7753.1
th 2
ins 100.000
nm no ph
DEC. & VT
dfrq 400.436
dn H1
dpwr 42
dof 0
dm nny
dmm w
dmf 8900
PROCESSING
lb 1.00
wtfile
proc ft
fn not used
werr
wexp
wbs
wnt
160.538
154.552
145.073
138.345
128.191
127.335
124.789
124.751
114.113
77.318
77.000
76.682
76.083
58.913
55.549
50.639
26.839
OH
OH
N
O
OMe
6a
180
160
140
120
100
80
60
40
20
ppm

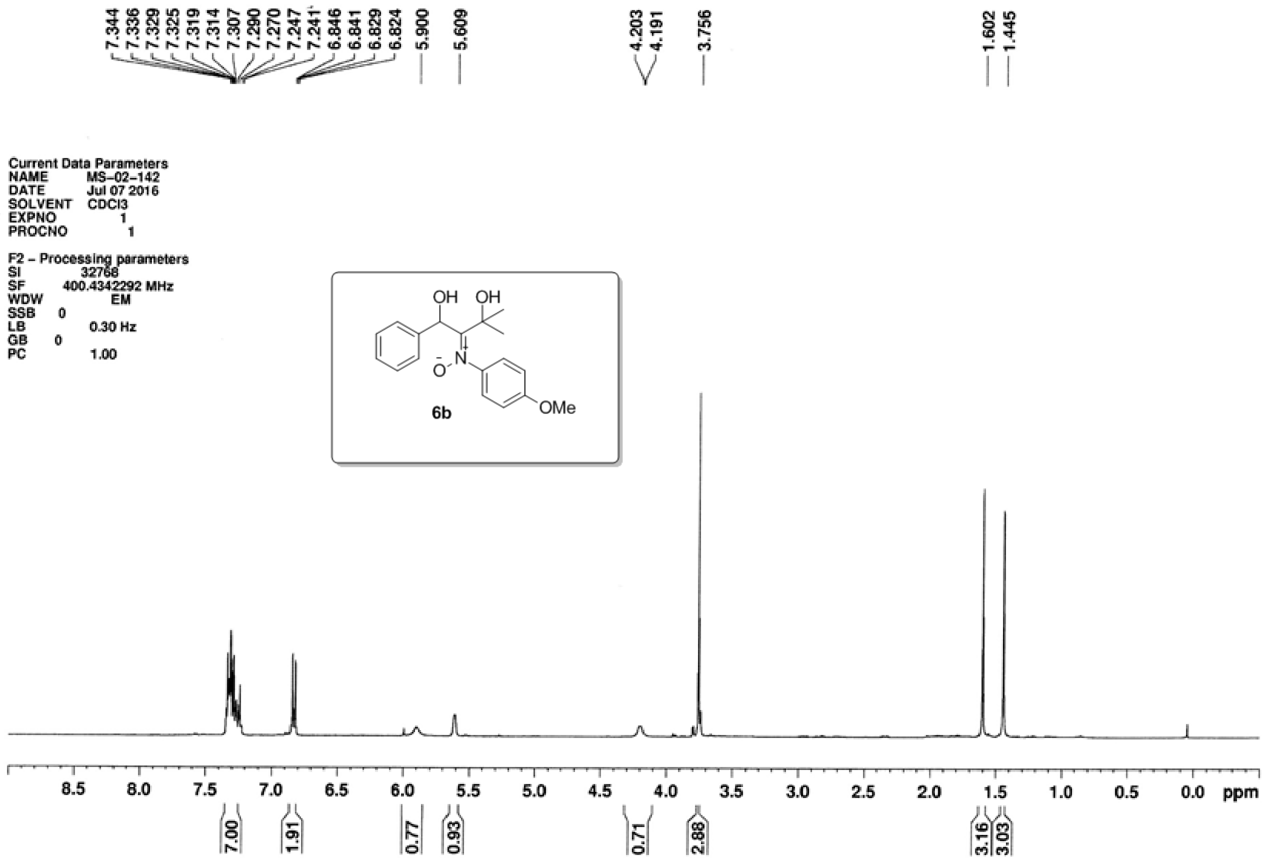
7.344
7.336
7.329
7.325
7.319
7.314
7.307
7.290
7.270
7.247
7.241
6.846
6.841
6.829
6.824
5.900
5.609
4.203
4.191
3.756
1.602
1.445
Current Data Parameters
NAME MS-02-142
DATE Jul 07 2016
SOLVENT CDCl3
EXPNO 1
PROCNO 1
F2 - Processing parameters
SI 32768
SF 400.4342292 MHz
WDW EM
SSB 0
LB 0.30 Hz
GB 0
PC 1.00
OH
OH
OMe
6b
8.5
8.0
7.5
7.0
6.5
6.0
5.5
5.0
4.5
4.0
3.5
3.0
2.5
2.0
1.5
1.0
0.5
0.0
ppm
7.00
1.91
0.77
0.93
0.71
2.88
3.16
3.03

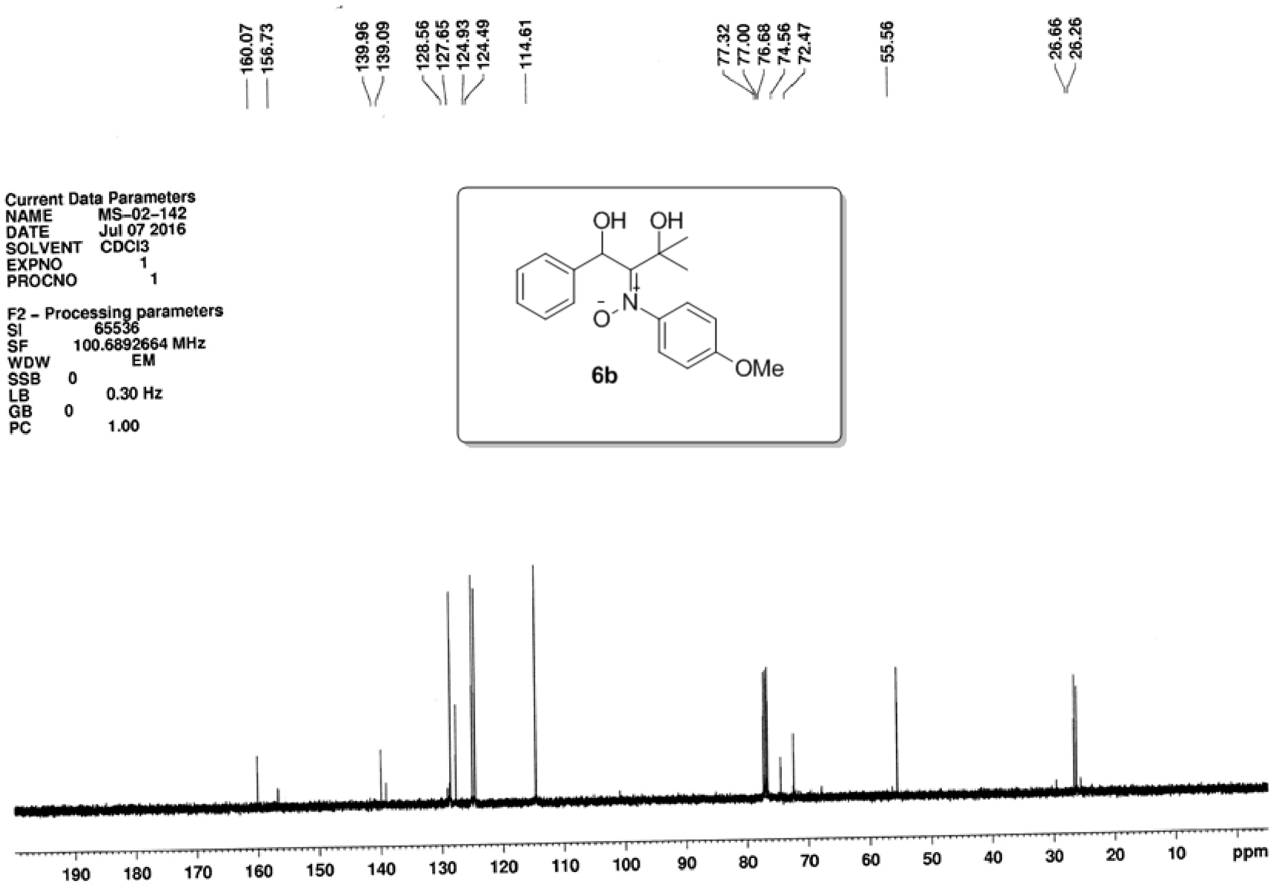
160.07
156.73
139.96
139.09
128.56
127.65
124.93
124.49
114.61
77.32
77.00
76.68
74.56
72.47
55.56
26.66
26.26
Current Data Parameters
NAME MS–02–142
DATE Jul 07 2016
SOLVENT CDCl3
EXPNO 1
PROCNO 1
F2 – Processing parameters
SI 65536
SF 100.6892664 MHz
WDW EM
SSB 0
LB 0.30 Hz
GB 0
PC 1.00
OH OH
N
O
OMe
6b
190 180 170 160 150 140 130 120 110 100 90 80 70 60 50 40 30 20 10 ppm

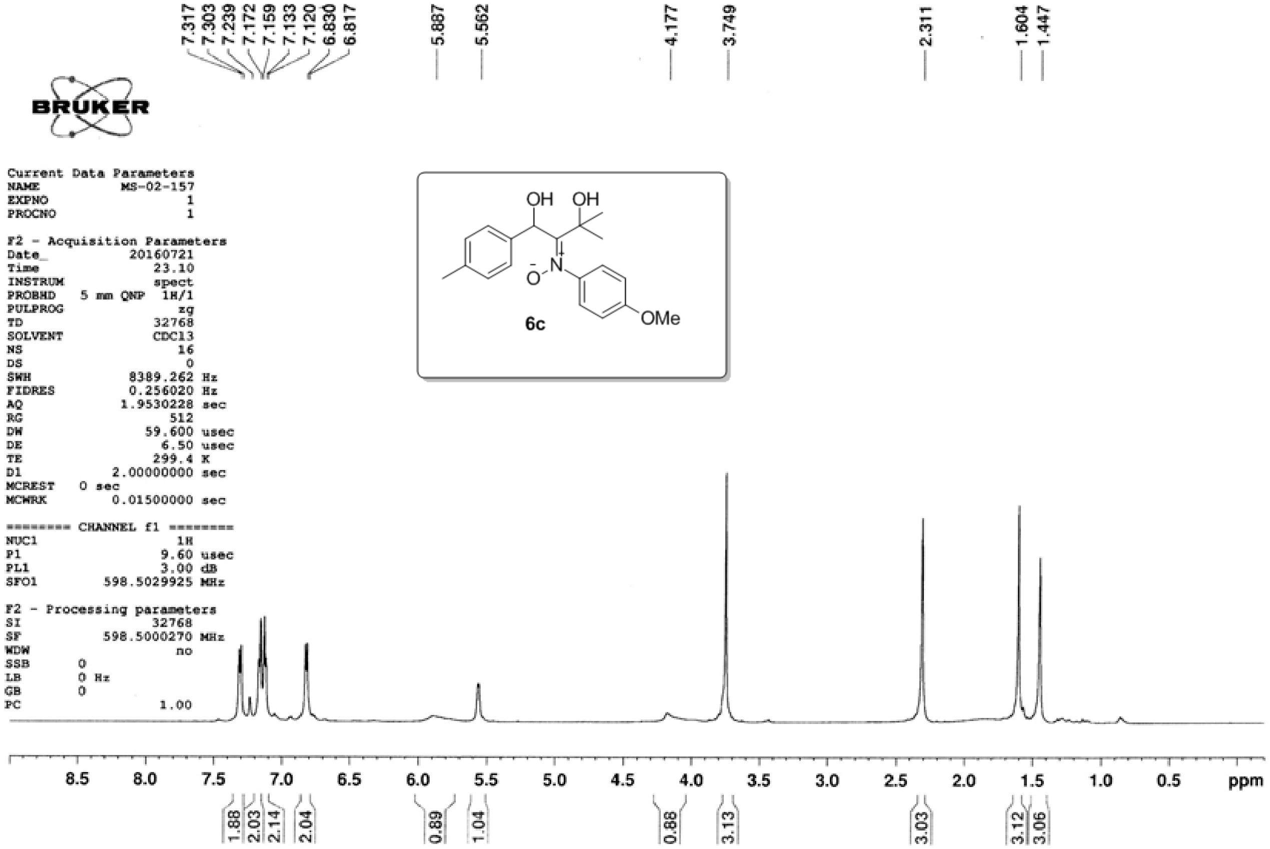
BRUKER
Current Data Parameters
NAME MS-02-157
EXPNO 1
PROCNO 1
F2 - Acquisition Parameters
Date_ 20160721
Time 23.10
INSTRUM spect
PROBHD 5 mm QNP 1H/1
PULPROG zg
TD 32768
SOLVENT CDCl3
NS 16
DS 0
SWH 8389.262 Hz
FIDRES 0.256020 Hz
AQ 1.9530228 sec
RG 512
DW 59.600 usec
DE 6.50 usec
TE 299.4 K
D1 2.00000000 sec
MCREST 0 sec
MCWRK 0.01500000 sec
======== CHANNEL f1 ========
NUC1 1H
P1 9.60 usec
PL1 3.00 dB
SFO1 598.5029925 MHz
F2 - Processing parameters
SI 32768
SF 598.5000270 MHz
WDW no
SSB 0
LB 0 Hz
GB 0
PC 1.00
7.317 7.303 7.239 7.172 7.159 7.133 7.120 6.830 6.817 5.887 5.562 4.177 3.749 2.311 1.604 1.447
OH OH OMe
6c
8.5 8.0 7.5 7.0 6.5 6.0 5.5 5.0 4.5 4.0 3.5 3.0 2.5 2.0 1.5 1.0 0.5 ppm
1.88 2.03 2.14 2.04 0.89 1.04 0.88 3.13 3.03 3.12 3.06

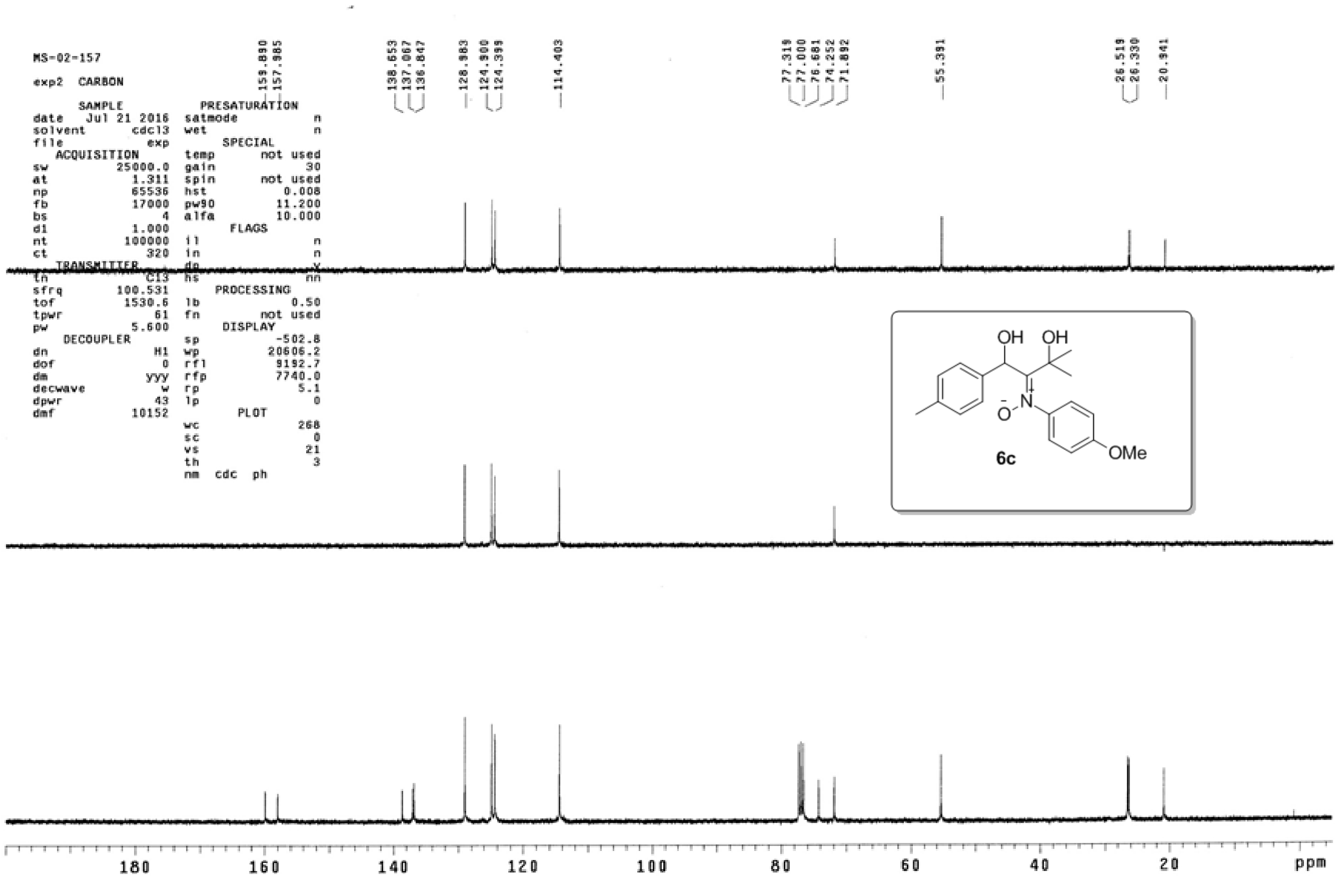
MS-02-157
exp2 CARBON
SAMPLE
date Jul 21 2016
solvent cdcl3
file exp
ACQUISITION
sw 25000.0
at 1.311
np 65536
fb 17000
bs 4
d1 1.000
nt 100000
ct 320
TRANSMITTER
tn C13
sfrq 100.531
tof 1530.6
tpwr 61
pw 5.600
DECOUPLER
dn H1
dof 0
dm yyy
decwave w
dpwr 43
dmf 10152
PRESATURATION
satmode n
wet n
SPECIAL
temp not used
gain 30
spin not used
hst 0.008
pw90 11.200
alfa 10.000
FLAGS
il n
in n
dp y
hs nn
PROCESSING
lb 0.50
fn not used
DISPLAY
sp -502.8
wp 20606.2
rfl 9192.7
rfp 7740.0
rp 5.1
lp 0
PLOT
wc 268
sc 0
vs 21
th 3
nm cdc ph
159.890
157.985
138.653
137.067
136.847
128.983
124.900
124.399
114.403
77.319
77.000
76.681
74.252
71.892
55.391
26.519
26.330
20.941
OH
OH
N
O
OMe
6c
180
160
140
120
100
80
60
40
20
ppm

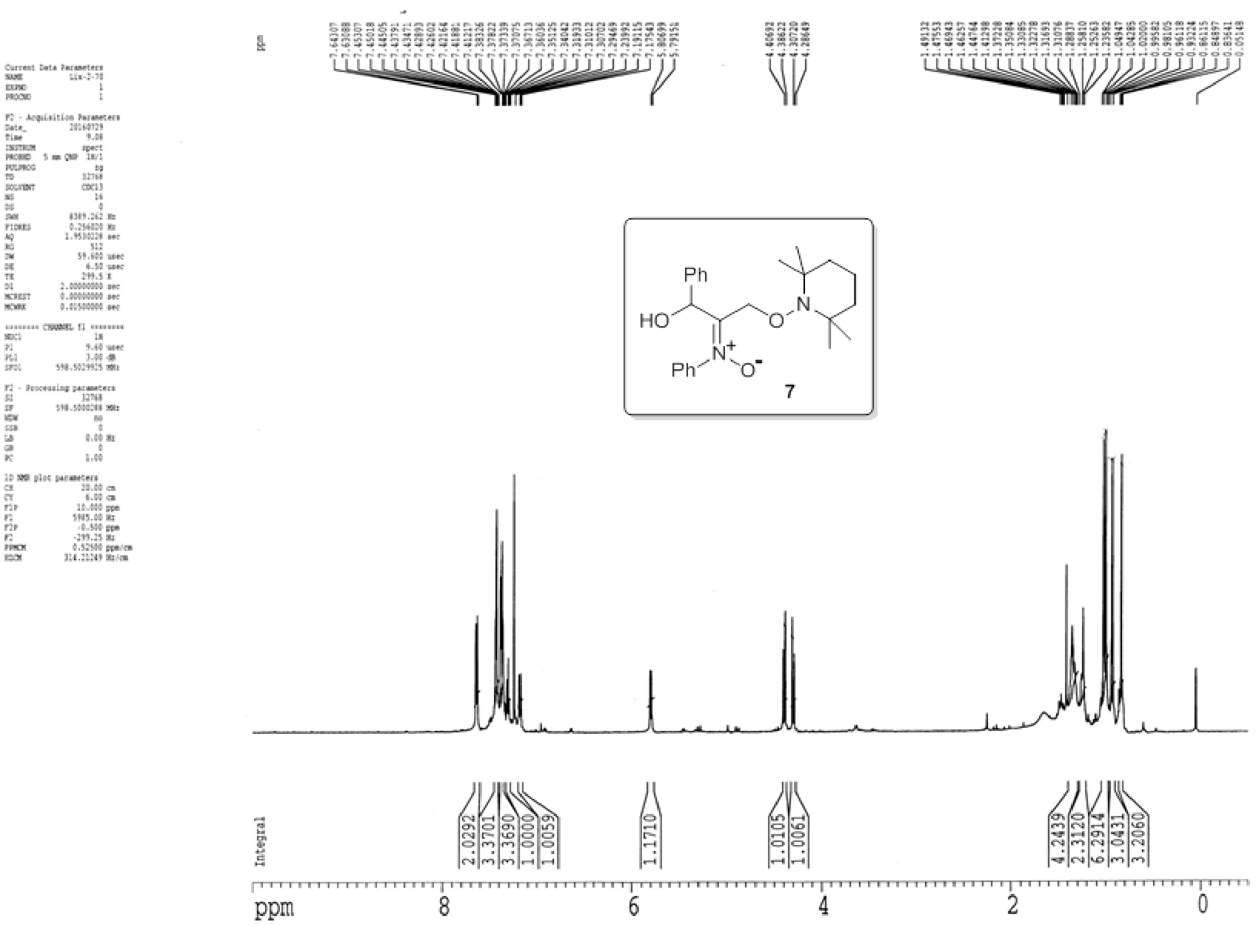
Ph
HO
O
N
N+
O-
Ph
7
Integral
ppm
8
6
4
2
0

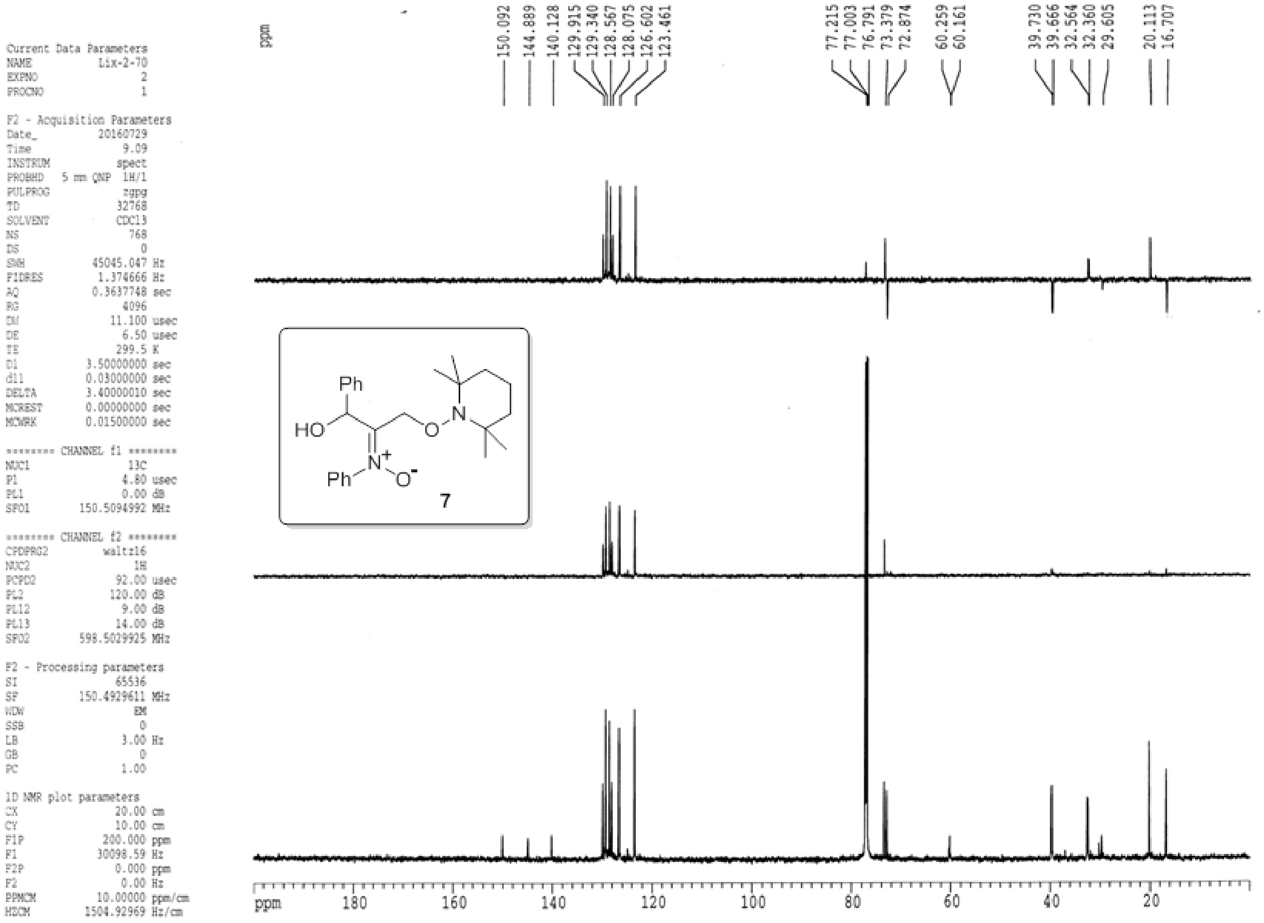
Current Data Parameters
NAME Lix-2-70
EXPNO 2
PROCNO 1
F2 - Acquisition Parameters
Date_ 20160729
Time 9.09
INSTRUM spect
PROBHD 5 mm QNP 1H/1
PULPROG zgpg
TD 32768
SOLVENT CDCl3
NS 768
DS 0
SWH 45045.047 Hz
FIDRES 1.374666 Hz
AQ 0.3637748 sec
RG 4096
DW 11.100 usec
DE 6.50 usec
TE 299.5 K
D1 3.50000000 sec
d11 0.03000000 sec
DELTA 3.40000010 sec
MCREST 0.00000000 sec
MCWRK 0.01500000 sec
======== CHANNEL f1 ========
NUC1 13C
P1 4.80 usec
PL1 0.00 dB
SFO1 150.5094992 MHz
======== CHANNEL f2 ========
CPDPRG2 waltz16
NUC2 1H
PCPD2 92.00 usec
PL2 120.00 dB
PL12 9.00 dB
PL13 14.00 dB
SFO2 598.5029925 MHz
F2 - Processing parameters
SI 65536
SF 150.4929611 MHz
WDW EM
SSB 0
LB 3.00 Hz
GB 0
PC 1.00
1D NMR plot parameters
CX 20.00 cm
CY 10.00 cm
F1P 200.000 ppm
F1 30098.59 Hz
F2P 0.000 ppm
F2 0.00 Hz
PPMCM 10.00000 ppm/cm
HZCM 1504.92969 Hz/cm
ppm
150.092
144.889
140.128
129.915
129.340
128.567
128.075
126.602
123.461
77.215
77.003
76.791
73.379
72.874
60.259
60.161
39.730
39.666
32.564
32.360
29.605
20.113
16.707
Ph
HO
N
O
N+
O-
Ph
7
ppm 180 160 140 120 100 80 60 40 20
